# Harmine Derivatives as Anticancer Agents Endowed With Potent and Selective Antileukemia Activity: Synthesis, Biological Evaluation, Proapoptotic and Genotoxic Activity

**DOI:** 10.1002/ardp.70197

**Published:** 2026-02-10

**Authors:** Abdul Aziz Timbilla, Filip Pidany, Eliska Kohelova, Jana Kroustkova, Karel Kralovec, Jan Rataj, Martina Ceckova, Negar Maafi, Víctor Lopez, Cristina Moliner Langa, Stefan Kosturko, Jaroslav Jenco, Darina Muthna, Darja Koutova, Martina Rezacova, Lucie Cahlikova, Jakub Chlebek, Radim Havelek

**Affiliations:** ^1^ Department of Medical Biochemistry, Faculty of Medicine in Hradec Kralove Charles University Hradec Kralove Czech Republic; ^2^ Department of Pharmacognosy and Pharmaceutical Botany, Faculty of Pharmacy Charles University Hradec Kralove Czech Republic; ^3^ Department of Biological and Biochemical Sciences, Faculty of Chemical Technology University of Pardubice Pardubice Czech Republic; ^4^ Department of Pharmacology and Toxicology, Faculty of Pharmacy in Hradec Kralove Charles University Hradec Kralove Czech Republic; ^5^ Faculty of Health Sciences Universidad San Jorge Zaragoza Spain; ^6^ Department of Analytical Chemistry, Faculty of Pharmacy in Hradec Kralove Charles University Hradec Kralove Czech Republic

**Keywords:** cancer, cell cycle, cytotoxicity, DNA damage, harmine, semisynthetic derivatives

## Abstract

β‐Carboline alkaloids, such as harmine (**1**), have demonstrated notable anticancer properties, making them attractive candidates for anticancer drug development. This study evaluated the antiproliferative activity of compound **1** and thirty‐three *N* ^9^‐substituted derivatives across a panel of cancer cell lines representing various histotypes. Among these, derivative **6**, a harmine analog bearing a 3,5‐dimethylbenzyl substituent, was the most potent, showing enhanced cytotoxicity and selectivity toward cancer cells. Compound **6** exhibited IC_50_ values below 10 µM in all tested cancer cell lines, while its IC_50_ in non‐cancerous cells exceeded 100 µM. Viability assays and xCELLigence real‐time monitoring confirmed a concentration‐dependent inhibition of cancer cell growth with minimal effects on non‐malignant cells. Flow cytometry demonstrated G1 phase arrest in MOLT‐4 cells, supported by Western blot data showing reduced phosphorylated Rb and increased p27 protein levels. Apoptosis induction was confirmed through Annexin V/PI staining, TUNEL assays, and caspase activation studies. These revealed the involvement of both intrinsic (caspase‐9) and extrinsic (caspase‐8) apoptotic pathways, along with activation of caspases 3/7. Western blot analysis also showed a concentration‐dependent increase in the Bax/Bcl‐2 ratio. Immunofluorescence microscopy visualization indicated DNA damage through elevated levels of PAR and γH2AX, consistent with single‐ and double‐strand DNA breaks. Importantly, compound **6** exhibited low inhibitory activity against monoamine oxidase A (MAO‐A) and did not promote reactive oxygen species (ROS) generation, minimizing potential off‐target effects. Together, these findings support the potential of compound **6** as a selective and effective candidate for antileukemia therapy.

## Graphical abstract

We identified a novel N9‐substituted harmine analog, compound **6**, that selectively suppresses leukemic cell growth, triggers DNA damage, and activates apoptosis while sparing healthy fibroblasts. This discovery highlights N9‐modified β‐carbolines as a powerful new class of selective antileukemic agents, bridging natural product chemistry with next‐generation cancer therapeutics.

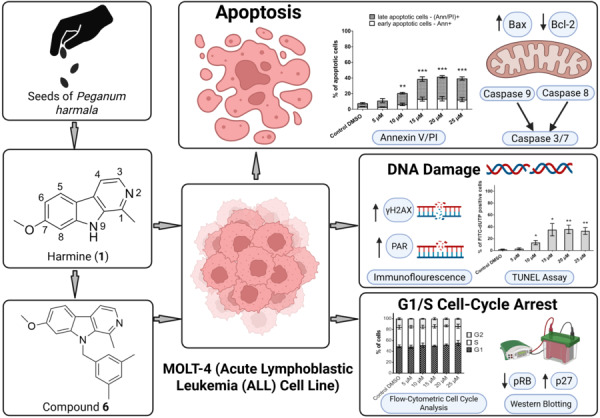

**Abbreviations**
AChE: acetylcholinesterase
ADME: absorption, distribution, metabolism, excretion
AKT: protein kinase B
ALL: acute lymphoblastic leukemia
AML: acute myeloid leukemia
Bax: Bcl‐2‐associated X protein
BBB: blood–brain barrier
Bcl‐2: B cell lymphoma 2
CDK: cyclin‐dependent kinase
CI: cell index
CNS: central nervous system
COX‐2: cyclooxygenase‐2
DAPI: 4′,6‐diamidino‐2‐phenylindole
DMF: dimethylformamide
DMSO: dimethyl sulfoxide
DNA: deoxyribonucleic acid
DNMTs: DNA methyltransferase
DPBS: Dulbecco's phosphate‐buffered saline
DSB: double‐strand break
DYRK1A: dual‐specificity tyrosine‐phosphorylation‐regulated kinase 1A
EMT: epithelial–mesenchymal transition
ERK: extracellular signal‐regulated kinase
ESI‐HRMS: electrospray ionization high‐resolution mass spectrometry
ESOL: estimated solubility
FDA: Food and Drug Administration
FITC‐dUTP: fluorescein isothiocyanate‐labeled deoxyuridine
FLT3: FMS‐like tyrosine kinase 3
FLT3‐ITD: FLT3‐internal tandem duplication
GHS: globally harmonized system
GI: gastrointestinal
GP: growth percentage
GSK‐3β: glycogen synthase kinase‐3β
H2AX: H2A histone family member X
HBA: hydrogen bond acceptor
HBD: hydrogen bong donor
HPLC: high‐performance liquid chromatography
ITD: internal tandem duplicates
LC: liquid chromatography
LD_50_
: lethal dose for 50% of the tested population
MAO‐A: monoamine oxidase‐A
MAPK: mitogen‐activated protein kinase
MMP: matrix metalloproteinase
MOMP: mitochondrial outer membrane permeabilization
MS: mass spectrometry
mTOR: mechanistic target of rapamycin
Mw: molecular weight
NIH: National Institutes of Health
NMR: nuclear magnetic resonance
NO: nitric oxide
NSCLC: non‐small cell lung cancer
PAR: poly(ADP‐ribose)
PARP: poly(ADP‐ribose) polymerase
P‐gp: P‐glycoprotein
PI: propidium iodide
PI3K: phosphoinositide 3‐kinase
Q‐TOF: quadrupole‐time‐of‐flight
ROS: reactive oxygen species
RTCA: real‐time cell analysis
RTCA: real‐time cell analysis
SAPK/JNK: stress‐activated protein kinases/c‐Jun N‐terminal kinases
SD: standard deviation
SMILES: simplified molecular input line entry system
SSB: single‐strand break
TBHP: tert‐butyl hydroperoxide
TBS: Tris‐buffered saline
THF: tetrahydrofuran
TLC: thin‐layer chromatography
TPSA: total polar surface area
TRAIL: TNF‐related apoptosis‐inducing ligand
TUNEL: terminal deoxynucleotidyl transferase dUTP nick‐end labeling
UHPLC: ultra‐high‐performance liquid chromatography
VEGF: vascular endothelial growth factor
VNMR: varian nuclear magnetic resonance spectrometer

## 1 Introduction

The discovery and development of novel anticancer agents derived from natural sources has been growing in recent years. Harmine (**1**), a β‐carboline alkaloid identified in various plants such as *Peganum harmala* L., *Peganum nigellastrum* Bunge [1] or *Banistereopsis caapi* (Spruce ex Griseb.) C.V. Morton [2, 3], represents a particularly promising natural compound. Traditionally, **1**‐containing plants have been utilized in the Middle East, Central Asia, and North Africa for their diverse therapeutic properties [4, 5, 6]. Alkaloid **1** was first isolated in the 1840s from the seeds and roots of *P. harmala*, and it quickly gained recognition for its broad spectrum of pharmacological properties [7]. The targets of **1** and its semisynthetic derivatives are various, encompassing antibacterial, antimalarial, antiviral, anti‐inflammatory, neuroprotective, epigenetic modulation, and antitumor activities [8, 9]. These phytocompounds demonstrated broad‐spectrum bioactivity, including antimicrobial effects against a range of Gram‐positive and Gram‐negative bacterial strains [10], antiplasmodial activity through disruption of the *Plasmodium* life cycle [11], antiviral effects via inhibition of viral replication [12], and modulation of key host targets such as pro‐inflammatory signaling pathways, acetylcholinesterase (AChE) [13], glycogen synthase kinase‐3β (GSK‐3β) [14], dual‐specificity tyrosine‐phosphorylation‐regulated kinase 1A (DYRK1A) [15], and DNA methyltransferases (DNMTs) [16]. As outlined in our earlier review, the parent **1** and some of its analogs exhibited promising anticancer potential, acting through mechanisms mirroring the modes of action of some clinically validated chemotherapeutic agents [3].

Notably, **1** demonstrates marked cytotoxic and antiproliferative activity across various solid or hematological cancer cell lines [7, 17, 18]. Of particular interest is the presence of an indole moiety within its structure, as indole‐based heterocycles represent a well‐established pharmacophore in anticancer drug development. Several Food and Drug Administration (FDA)‐approved and clinically investigated anticancer agents, including FLT3 inhibitors such as midostaurin, sunitinib, crenolanib, and lestaurtinib, incorporate indole scaffolds, highlighting the importance of this structural motif in targeting blood malignancies such as acute myeloid leukemia (AML) [19]. FLT3 is among the most frequently mutated genes in AML, with activating internal tandem duplications (ITDs) occurring in approximately 24% of patients and being strongly associated with adverse prognosis [20]. To further evaluate the therapeutic relevance of **1** and its derivatives, it is essential to compare their β‐carboline (9*H*‐pyrido[3,4‐*b*]indole) core with the structural frameworks of these clinically validated kinase inhibitors. These agents typically feature a planar, nitrogen‐containing heterocyclic scaffold, such as indoles or quinolines, which facilitates effective binding within the ATP‐binding cleft of kinases, thereby enabling potent inhibition of oncogenic targets like FLT3 [19]. A recent study has shown that β‐carboline‐based derivatives exhibit selective anti‐AML effects through modulation of key oncogenic pathways, including PI3K/AKT and MAPK/ERK, positioning this scaffold as a relevant modulator of kinase signaling in AML [21]. Given the structural resemblance between the β‐carboline scaffold of **1** and the indole‐based heterocycles present in clinically validated FLT3 inhibitors, we investigated the selectivity of compound **6** by evaluating its antiproliferative activity in human AML cell lines harboring the FLT3‐ITD mutation (MOLM‐13 and MV4‐11) in comparison with the FLT3 wild‐type THP‐1 cell line.

Abnormal cell proliferation is a hallmark of cancer, and overexpression of DYRK1A has been shown to enhance cancer cell resistance to proapoptotic stimuli. DYRK1A signaling has been increasingly linked to the acquisition of multidrug resistance, in part through the promotion of cancer stem‑like traits, positioning also this kinase as an attractive therapeutic target for counteracting treatment resistance across multiple cancer types [22]. Originally identified as a potent ATP‐competitive inhibitor of DYRK1A by Bain et al. in 2007 [23], **1** has since been shown to also target additional members of the CMGC kinase family, including DYRK1B, DYRK2, and DYRK4, highlighting its broader kinase‐inhibitory profile [24]. In this regard, the β‐carboline backbone of **1** holds structural similarities with indole‐based kinase inhibitors, suggesting its potential to interfere with oncogenic signaling pathways beyond DYRK1A [25].

Mechanistically, **1** exerts its anticancer activity through multiple, interconnected pathways. A primary mode of action involves direct interaction with DNA. Compound **1** preferentially intercalates into guanine‐cytosine base pairs rather than adenine‐thymine base pairs. This interferes with the synthesis and replication of DNA as well as impedes DNA topoisomerase I activity [26, 27, 28]. The formation of **1**‐DNA complexes is driven by exothermic reactions involving van der Waals forces, hydrophobic interactions, and hydrogen bonding, collectively inducing significant DNA damage that triggers cellular stress responses [29]. Beyond DNA intercalation, **1** exhibits multiple anticancer mechanisms, including induction of DNA damage, enhancement of ROS production, modulation of cell cycle progression, inhibition of angiogenesis, induction of apoptosis, regulation of autophagy, and activation of p53, identifying it as a multifaceted therapeutic agent [3]. At the molecular signaling level, **1** modulates crucial pathways including the phosphatidylinositol 3‐kinase (PI3K)/protein kinase B (AKT) signaling cascade, leading to upregulation of the epithelial marker E‐cadherin and the downregulation of mesenchymal markers such as vimentin and N‐cadherin [30]. In addition, **1** inhibits DNA methyltransferases (DNMTs), resulting in G1 phase cell cycle arrest and consequent growth inhibition [7]. A significant mechanism of **1** involves the induction of apoptosis and subsequent elimination of cancer cells through modulation of the balance between proapoptotic and antiapoptotic proteins, notably Bcl‐2 and Bax. This shift disrupts mitochondrial function and triggers apoptotic signaling pathways. Specifically, treatment with **1** leads to upregulation of Bax concomitant with downregulation of Bcl‐2 [31]. The **1** has demonstrated proapoptotic effects in chemotherapy‐resistant cancer cell models and has shown potential to overcome resistance to anticancer agents [3]. In this context, the application of **1** induces nuclear fragmentation and the formation of apoptotic bodies in melanoma cells. This proapoptotic activity is associated with downregulation of the antiapoptotic protein Bcl‐2 and upregulation of proapoptotic markers, including Bax, caspase‐3, caspase‐8, caspase‐9, and Bid [32]. In a study by Uhl et al. [33], dose‐dependent treatment of breast cancer cells with **1** resulted in increased Bax expression, while levels of the protein Bcl‐2, as well as phosphorylated Erk (p‐Erk), mTOR (p‐mTOR), and AKT (p‐AKT), were decreased. Notably, total levels of AKT, mTOR, and Erk remained unchanged. These findings suggest that **1** induces apoptosis in breast cancer cells, at least in part, through inhibition of the PI3K/AKT/mTOR signaling pathway. Other reported mechanisms contributing to the anticancer potential of **1** include the induction of DNA damage, which leads to the transcriptional upregulation of p53 target genes such as p21, resulting in cell cycle arrest, senescence, or apoptosis. Moreover, **1**‐induced genotoxic stress is associated with increased levels of DNA damage and repair markers, such as γH2AX, which was significantly elevated in MCF‐7 breast cancer cells following 24‐h exposure [34].

Despite these promising anticancer properties, the clinical application of **1** as part of the anticancer drug portfolio is hindered by its neurotoxicity, primarily resulting from off‐target inhibition of monoamine oxidase A (MAO‐A) observed in animal models. Preclinical studies in animal models have demonstrated that acute administration of **1** induces tremorgenic and hypothermic effects, leading to movement impairment [15, 17]. Furthermore, long‐term treatment with **1** has been associated with significant weight loss, locomotor deficits, and transient tremors in socially defeated rats, collectively limiting its dosing potential for in vivo purposes [35]. These neurotoxic effects substantially narrow the therapeutic window and underscore the need for structural optimization to enhance safety without compromising efficacy [32].

In addition, in the pursuit of developing **1** as a molecule with anticancer potential, multiple structural modifications were proposed to improve the drug‐likeness, enhance its therapeutic efficacy, introduce novel anticancer‐related biological activities, minimize toxicity, and increase bioavailability. Among these, semisynthetic modifications concentrated at the C1, C3, *N* ^*2*^, and *N* ^9^ positions of the **1** core scaffold demonstrated an improved balance between physicochemical properties and enhanced candidate anticancer activity [8, 36]. Notably, the *N* ^9^ position has emerged as a critical site for structural optimization within the β‐carboline scaffold, as exemplified by antitumor agent‐49 (CAS No: 2763914‐21‐0). This derivative exhibited significantly enhanced antiproliferative activity against HepG2 cells (IC_50_ = 1.79 ± 0.16 μM), representing a marked improvement over its parent compound (IC_50_ = 16.8 ± 2.33 μM) [37]. Moreover, recent advances have highlighted *N* ^9^‐position modifications as a promising strategy to enhance the antitumor efficacy of **1** while simultaneously reducing its acute toxicity and neurotoxic effects [7].

Considering this background, we rationally designed and synthesized a library of *N* ^9^‐substituted **1** derivatives (compounds **2**–**34**), incorporating various aromatic and aliphatic ligands onto the β‐carboline scaffold. Fourteen (**3**, **5**, **6**, **7**, **8**, **9**, **10**, **12**, **18**, **19**, **20**, **21**, **22**, and **23**) of these tricyclic 9H‐pyrido[3,4‐*b*]indole derivatives were newly prepared in this study. The rationale for *N* ^9^ substitution stems from structure–activity relationship studies indicating that short alkyl or aryl substitutions at *N* ^9^ can enhance cytotoxic potency and potentially reduce MAO‐A inhibition, thereby mitigating neurotoxicity [17, 18]. Their antiproliferative activity was subsequently evaluated across a panel of tumor cell lines representing different tissue origins. Given that the cytotoxicity screening identified the 3,5‐dimethylbenzyl analog **6** as the most potent compound, we proceeded to investigate, for the first time, the effects of compound **6** on the survival and proliferation of acute lymphoblastic leukemia (ALL) MOLT‐4 cells. Simultaneously, we determined proliferation and viability against non‐cancer lung fibroblast MRC‐5 cells to verify the potential of this molecule as a candidate for prospecting novel antitumor drugs. To further explore the antileukemic potential of compound **6**, we assessed its activity against AML cell lines with varying FLT3 mutation statuses. Moreover, we evaluated the ability of the most potent derivative, **6**, to inhibit MAO‐A, promote intracellular ROS production, induce DNA damage, and activate intracellular signaling pathways leading to cell cycle arrest and apoptosis. In summary, DNA‐damaging activity, DNA fragmentation, proapoptotic activity, and cell cycle progression perturbing activity indicate that compound **6** is a promising chemotherapeutic candidate. Moreover, the findings of this study demonstrate that compound **6** exhibits significant anti‐leukemic activity against ALL, providing the first evidence of the therapeutic potential of *N* ^9^‐substituted **1**‐derived compounds in the treatment of this most prevalent pediatric malignancy.

## 2 Results and Discussion

### 2.1 Extraction and Isolation of Harmine (**1**)

Compound **1** was isolated in sufficient quantity (2.1 g) for the preparation of semi‐synthetic harmine derivatives from *P. harmala* seeds using subsequently a combination of Soxhlet extraction, LLE, Flash chromatography, and preparative TLC. Its structure was confirmed by LC‐MS, ^1^H NMR, and ^13^C NMR experiments, with the obtained data aligning well with previously reported data [38].

During the extraction process, we obtained an alkaloid yield of 6.12%, which is consistent with 6.63% yield previously reported by Madah et al. [39]. This comparable yield indicates both richness of the plant material in alkaloids and the effectiveness of the employed separation methods. Alkaloid **1** is typically isolated from plant extracts using various chromatographic techniques, including column chromatography, preparative HPLC, preparative TLC, and pH‐zone‐refining counter‐current chromatography [3]. In this study, however, we report the use of Flash chromatography for **1** isolation for the first time. Although originally developed for the rapid purification of synthetic compounds, Flash chromatography has recently gained traction for the separation of complex natural product mixtures, including alkaloids [40, 41]. Silica gel is the most commonly used adsorbent for isolating plant metabolites due to its effectiveness and versatility. However, aluminum oxide is also frequently employed in alkaloid separations. Its unique adsorption behavior stems from the positive electric field of Al^3+^ ions, and basic sites on the surface enhance interaction with nitrogen atoms of **1**, resulting in improved separation selectivity and resolution compared to silica gel [42].

### 2.2 Preparation of **1** Derivatives

Previous investigations have found that β‐carboline compounds such as **1** can be highly cytotoxic and effectively suppress tumor cell proliferation through proapoptotic mechanism [17]. The anticancer action of β‐carboline alkaloids, such as **1**, can be enhanced via structural modification while concurrently reducing their toxic or other confounding off‐target effects [43, 44]. The strategic modification at the *N* ^9^ position had emerged as a pivotal approach to enhance its antitumor selectivity and minimize off‐target monoamine oxidase (MAO) inhibition. Substitution at *N* ^9^ with bromo alkyl chains, such as 5‐bromopentyl and 6‐bromohexyl, has been reported to significantly enhance cytotoxicity across diverse cancer cell lines, with IC_50_ values ranging from 0.45 to 9.34 µM in HepG2, MCF‐7, and A549 cells while reducing MAO‐A/MAO‐B binding affinity by introducing steric hindrance [45]. Importantly, these structural changes maintained fundamental antitumor mechanisms of **1**, including DNA intercalation and apoptosis induction through Bcl‐2 suppression but decoupled these effects from neurotoxic MAO interactions [46]. The resulting derivatives exhibited >100‐fold selectivity for malignant over non‐cancer cells, underscoring the therapeutic potential of *N* ^9^‐functionalized **1** analogs. Notably, Chen et al. [17] described compound **8**, which features an ethyl formate substitution at C3 and a benzyl group at *N* ^9^, demonstrating superior potency with IC_50_ values as low as 0.011–0.021 µM in HepG2 cells. This finding highlights the critical role of *N* ^9^ substitution in enhancing the antitumor potential of **1** while reducing toxicity [17].

**Scheme 1:**
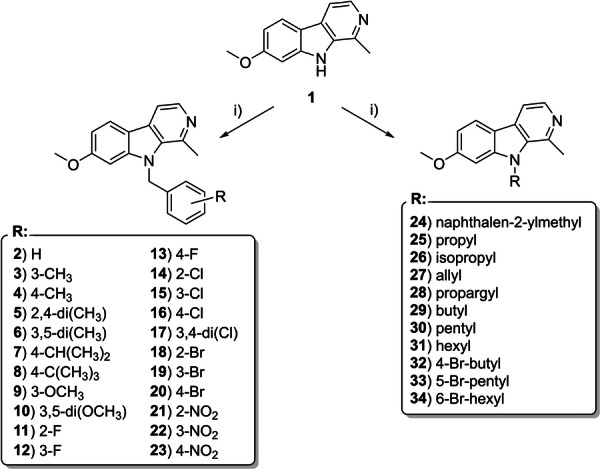
Synthesis of N^9^‐substituted harmine derivatives (**2**–**34**) with phenyl and naphthyl skeletons featuring various phenyl modifications (**2**–**24**) and aliphatic modifications (**25**–**34**). Reagents and conditions: (i) NaH, dry DMF/THF 1:1 (v/v), 0°C, 10 min; then RT, corresponding aryl or alkyl bromide, 24 h; yields 81%–95% (aromatic derivatives) and 58%–82% (aliphatic derivatives).

Based on this rationale, to optimize therapeutic efficacy, a library of *N* ^9^‐substituted derivatives of compound **1**, encompassing compounds **2**–**34**, was synthesized through a streamlined and efficient one‐pot reaction. The library comprised two primary categories: aromatic derivatives (compounds **2**–**24**; Scheme 1) and aliphatic derivatives (compounds **25**–**34**; Scheme 1). The synthetic strategy involved selective deprotonation of **1** at the *N* ^9^‐position using sodium hydride (NaH) in a dry DMF/THF (1:1, v/v) mixture at 0°C, followed by nucleophilic substitution with the corresponding alkyl or aryl bromides at room temperature. This method enabled the preparation of structurally diverse derivatives in moderate to excellent yields following a 24‐h reaction period.

The aromatic derivatives **2**–**24** were designed to investigate steric and electronic effects by introducing a variety of substituents into the benzyl moiety, while keeping the naphthyl moiety unmodified. The simplest member in the series, benzyl‐substituted derivative **2** (Scheme 1), served as the foundation for further modifications. Initial optimization of compound **2** focused on introducing methyl groups, including monosubstitution at the *m*‐ and *p*‐positions (compounds **3** and **4**) and disubstitution at the 2,4‐ and 3,5‐positions (compounds **5** and **6**, respectively). Subsequent structural exploration introduced bulkier substituents, such as isopropyl and *tert*‐butyl groups at the *p*‐position, yielding derivatives **7** and **8**. Further diversification involved electron‐donating methoxy groups, resulting in the *m*‐substituted derivative **9** and the 3,5‐dimethoxy derivative **10**. Electron‐withdrawing groups were also thoroughly investigated. Halogenated benzyl derivatives (compounds **11**–**16** and **18**–**20**) incorporated fluorine, chlorine, and bromine as monohalogenated substituents at the *o*‐, *m*‐, and *p*‐positions. Additionally, compound **17** featured a 3,4‐dichlorophenyl substitution. Derivatives containing strongly electron‐withdrawing nitro groups (compounds **21**–**23**) were synthesized to cover all positional isomers, further broadening the range of substituents. To expand structural diversity, a naphthyl‐substituted derivative **24** was introduced, incorporating a larger aromatic system into the series.

For the aliphatic derivatives (compounds **25**–**34**; Scheme 1), the focus was on diversifying the aliphatic backbone by incorporating a range of saturated, unsaturated, straight, and branched alkyl groups. Straight‐chain alkyl groups, including propyl (**25**), butyl (**29**), pentyl (**30**), and hexyl (**31**), represented the simplest modifications. Branched alkyl substituents, such as isopropyl (**26**), were included to probe steric effects. Unsaturated derivatives, such as allyl (**27**) and propargyl (**28**), were synthesized to explore the impact of double and triple bonds. Finally, halogen‐substituted aliphatic chains, including 4‐bromobutyl (**32**), 5‐bromopentyl (**33**), and 6‐bromohexyl (**34**), were incorporated to further broaden the series and enhance structural complexity. ^1^H NMR, ^13^C NMR, ESI‐HRMS spectra, and LC‐UV chromatograms of prepared compounds are shown in the supporting material (Figure S1).

### 2.3 Cytotoxic Activity Screening of 1 and Semisynthetic Derivatives (2–34)

We assessed the cytotoxic and antiproliferative effects of **1** and thirty‐three derivatives, including fourteen novel compounds, across multiple cancer cell lines and the non‐cancerous MRC‐5 cell line as a control. Structural modifications with various alkyl and aryl groups yielded several promising candidates for anticancer drug development. The antiproliferative potential of the synthesized harmine derivatives (**2**–**34**) was evaluated against a panel of human tumor and non‐tumor cell lines derived from various tissues, namely Jurkat (acute T cell leukemia), MOLT‐4 (acute lymphoblastic leukemia), A549 (lung carcinoma), HT‐29 (colorectal adenocarcinoma), PANC‐1 (pancreas epithelioid carcinoma), A2780 (ovarian carcinoma), MCF‐7 (breast adenocarcinoma), SAOS‐2 (osteosarcoma), and MRC‐5 (non‐cancer lung fibroblasts) were used.  Compounds were compared to **1** and the reference drug, doxorubicin. Each compound was tested for cytotoxicity by treating cells with a 10 µM dose for 48 h. The inhibitory results against determined human cells are summarized in Table 1, Figure S2, Table 2, and Figure S3. These data demonstrated that compound **6** exerts the most significant inhibitory effects with a mean growth percentage (GP) value of 60%. Also, certain aliphatic derivatives, incorporating prop‐2‐en‐1‐yl (**27**) and propyl (**25**) substituents at position *N* ^9^ of the β‐carboline nucleus, exhibited notable growth inhibitory activity of 66% and 67%, respectively. However, compound **6** emerged as the most active agent, demonstrating notable cytotoxicity against Jurkat, MOLT‐4, and A2780 cell lines, with maximum cytotoxic effects of 5%, 20%, and 40%, respectively. The data indicated that compound **6** exerted the most potent cytotoxic effect among the tested compounds, displaying notable effects after 48 h of exposure at a 10 µM dose. Given the pronounced inhibitory effects of compound **6** on Jurkat, MOLT‐4, and A2780 cells, IC_50_ values were determined. The negative impact of compound **6** against Jurkat, MOLT‐4, and A2780 cells is illustrated in Table 3, with IC_50_ values of 2.90 ± 1.05, 7.68 ± 1.03, and 6.43 ± 1.13 µM, respectively. In our experiments, compound **6** demonstrated selective inhibitory activity, exhibiting no obvious cytotoxicity (IC_50_ = 106.50 ± 2.27 µM) against non‐cancerous lung fibroblasts MRC‐5. We subsequently calculated the selectivity index (SI) values, which represent the ratio of the IC_50_ value for the non‐cancerous MRC‐5 cell line to the IC_50_ value for the cancerous cell lines Jurkat, MOLT‐4, and A2780 after 48 h of treatment. The SI values for Jurkat, MOLT‐4, and A2780 were found to be 36.72, 13.87, and 16.56, respectively. Given that an SI value greater than 2 [38] indicates selective cytotoxicity of compound **6** toward cancer cells while only marginally affecting the viability of non‐cancerous lung fibroblasts (MRC‐5), these results suggest a high specificity for the semisynthetic compound **6** prepared by modification at the *N* ^9^ position of **1** with 3,5‐dimethylbenzyl group. The enhanced potential of this derivative could be because the lipophilic benzyl moiety included in this chemical is known to enhance metabolic stability and membrane permeability while preserving crucial interactions inside kinase ATP‐binding sites [47]. Compared to **1**, derivative **6** demonstrated superior antiproliferative activity across almost all tested cancer cell histotypes. It was observed that compound **6** effectively inhibited Jurkat and MOLT‐4 ALL cell growth. In particular, derivative **6** displayed interesting inhibitory activity toward A2780 ovarian carcinoma cells. Furthermore, the minimal cytotoxicity observed in MRC‐5 cells, with SI values exceeding 10, highlighted the enhanced therapeutic potential of compound **6** for further investigation. Our findings are consistent with previous studies demonstrating that the introduction of aromatic, short alkyl, or electron‐withdrawing groups at the *N* ^9^ position of **1** can significantly enhance its anticancer activity. The presence of bulky groups such as phenyl derivatives at the *N* ^9^ usually enhances activity against several potential targets used for cancer therapy, including PI3K/AKT/mTOR, ERK/JNK/p38, or p53/p21 pathway [7, 36]. Previous studies have also demonstrated that the presence of methyl groups in *N* ^9^ substitution of **1** derivative is associated with increased cytotoxic activity [48].

**Table 1:** The antiproliferative effect of each compound across all cell lines was calculated following treatment with a 10 µM concentration, using a 48‐h incubation period, and assessed via the WST‐1 method relative to negative control cells (0.1% DMSO‐treated, set at 100% proliferation).

	Jurkat	MOLT‐4	A549	HT‐29	PANC‐1	A2780	MCF‐7	SAOS‐2	MRC‐5
**Harmine (1)**	89	73	77	70	83	75	80	78	87
**2**	60	81	84	90	92	69	89	90	88
**3**	40	77	93	104	94	65	93	98	98
**4**	63	86	84	80	89	70	89	95	83
**5**	56	78	91	84	90	69	94	95	93
**6**	5	20	78	81	77	40	71	59	111
**7**	67	93	59	96	92	56	117	98	117
**8**	74	104	44	77	78	65	106	89	92
**9**	47	74	96	104	89	71	96	97	100
**10**	8	44	95	92	87	45	78	80	107
**11**	78	92	86	89	82	90	86	88	101
**12**	117	83	96	122	115	143	112	108	114
**13**	68	89	88	91	96	92	94	93	106
**14**	36	84	86	88	94	76	91	91	95
**15**	119	88	115	140	112	157	117	115	122
**16**	74	77	86	96	105	72	100	101	75
**17**	84	78	85	112	109	78	106	106	107
**18**	63	72	73	85	95	66	93	92	66
**19**	81	72	104	128	119	150	112	111	122
**20**	58	77	79	80	90	79	89	94	79
**21**	16	63	67	73	87	54	70	70	78
**22**	147	98	111	118	114	145	112	102	108
**23**	77	81	81	84	91	80	80	90	111
**24**	95	84	86	106	112	96	115	104	106
**25**	62	67	61	64	80	49	84	68	72
**26**	74	84	78	80	105	70	87	87	91
**27**	55	74	47	65	81	56	84	70	64
**28**	70	84	66	67	84	60	85	76	80
**29**	59	68	66	64	93	51	87	86	73
**30**	62	65	70	62	88	56	89	87	79
**31**	57	60	73	67	87	55	90	81	84
**32**	73	50	73	68	81	72	84	78	90
**33**	63	26	72	74	74	65	98	79	88
**34**	107	117	94	96	103	129	98	93	93
**DOX**	2	1	12	36	60	10	31	13	43
**0%–25%**	**26%–50%**	**51%–75%**							

*Note:* The values displayed indicate the percentage of viable cells at the end of the experiment. Each value is the mean of three independent experiments. Values from the intervals 0%–25%, 26%–50%, and 51%–75% are counterpointed with different colors. Doxorubicin at 1 μM was used as a reference drug.

**Table 2:** The table provides an overview of the mean growth percentage (GP), along with its minimum and maximum values, and highlights the three most responsive cell lines.

Compound	Mean GP	Range of GP	Three most sensitive cell lines
**1**	79	70–89	HT‐29, MOLT‐4, A2780
**2**	83	60–92	Jurkat, A2780, MOLT‐4
**3**	85	40–104	Jurkat, A2780, MOLT‐4
**4**	82	63–95	Jurkat, A2780, HT‐29
**5**	83	56–95	Jurkat, A2780, MOLT‐4
**6**	60	5–111	Jurkat, MOLT‐4, A2780
**7**	88	56–117	A2780, A549, Jurkat
**8**	81	44–106	A549, A2780, Jurkat
**9**	86	47–104	Jurkat, A2780, MOLT‐4
**10**	71	8–107	Jurkat, MOLT‐4, A2780
**11**	88	78–101	Jurkat, PANC‐1, A549
**12**	112	83–143	MOLT‐4, A549, SAOS‐2
**13**	91	68–106	Jurkat, A549, MOLT‐4
**14**	82	36–95	Jurkat, A2780, MOLT‐4
**15**	121	88–157	MOLT‐4, PANC‐1, A549
**16**	87	72–105	A278, Jurkat, MRC‐5
**17**	96	78–112	MOLT‐4, A2780, Jurkat
**18**	78	63–95	Jurkat, A2780, MRC‐5
**19**	111	72–150	MOLT‐4, Jurkat, A549
**20**	81	58–94	Jurkat, MOLT‐4, A549
**21**	64	16–87	Jurkat, A2780, MOLT‐4
**22**	117	98–147	MOLT‐4, SAOS‐2, MRC‐5
**23**	84	77–111	Jurkat, A2780, MCF‐7
**24**	100	84–115	MOLT‐4, A549, Jurkat
**25**	67	49–84	A2780, A549, Jurkat
**26**	83	70–105	A2780, Jurkat, A549
**27**	66	47–84	A549, Jurkat, A2780
**28**	75	60–85	A2780, A549, HT‐29
**29**	72	51–93	A2780, Jurkat, HT‐29
**30**	73	56–89	A2780, Jurkat, HT‐29
**31**	73	55–90	A2780, Jurkat, MOLT‐4
**32**	73	50–90	MOLT‐4, A2780, Jurkat
**33**	71	26–98	MOLT‐4, Jurkat, A2780
**34**	103	93–129	SAOS‐2, A549, HT‐29
Doxorubicin	23	1–60	MOLT‐4, Jurkat, A549

*Note:* The GP value of each compound represents the average proliferation after the application of a 10 μM dose within a 48‐h treatment time interval on nine cell lines. Data were calculated from three independent experiments and are expressed as a percentage of the proliferation of 0.1% DMSO mock‐treated control cells (100%).

**Table 3:** The IC_50_ values of compound 6 in different human cancer and non‐cancer cell lines after 48 h of treatment.^a^ ^,^ ^b^

Cell line	Compound 6
Jurkat	2.90 ± 1.05
MOLT‐4	7.68 ± 1.03
A2780	6.43 ± 1.13
MRC‐5	106.50 ± 2.27

^a^ Results are expressed in µM.

^b^ All experiments were conducted in triplicate, and the data are presented as mean values ± SD of at least three independent replications.

The antiproliferative structure–activity relationship (SAR) analysis revealed that the inhibitory activity of **1** was significantly enhanced by specific substituents. Notably, the introduction of 3‐methylbenzyl and 3‐methoxybenzyl substituents significantly enhanced cytotoxicity compared to **1** (compounds **3** and **9**), with further improvement upon adding a second methyl or methoxy group at the benzyl 5‐position (compounds **6** and **10**). Conversely, electronegative substituents at the benzyl 3‐position reduced activity, yielding lower potency than **1** (compounds **12**, **15**, **19,** and **22**). In contrast, electronegative groups at the 2‐position produced comparable or greater cytotoxic effects (compounds **11** and **18**), with chloro‐ and nitro‐substituted derivatives (compounds **14** and **21**) showing notably increased efficacy against the Jurkat cell line. Among the aliphatic **1** derivatives (compounds **25**–**34**), only modest improvements in activity were observed relative to **1**. Interestingly, compounds bearing 4‐bromopentyl (**33**) and 6‐bromohexyl (**34**) substituents did not exhibit the strong cytotoxic potential previously reported by Du et al. [46].

In the context of halogenated alkyl derivatives, their potential chemical reactivity introduces an important consideration in the interpretation of biological activity. Halogenated alkyl groups are known to be susceptible to nucleophilic substitution, particularly in the presence of biological nucleophiles such as alcohols, thiols, and amines. While there is currently no direct experimental literature evidence demonstrating that these aliphatic **1** derivatives undergo significant transformation by such nucleophiles under in vitro cell culture conditions, this hypothesis cannot be excluded. It may, in part, account for the discrepancies observed between our findings and those reported by [46]. Interestingly, the *N* ^9^‐haloalkyl **1** derivatives demonstrated variable antiproliferative activity across the tested cancer cell lines, with IC_50_ values ranging from the nanomolar in MCF‐7 breast cancer cells to the micromolar concentrations in SGC‐7901 gastric cancer and SMMC‐7721 liver cancer cells. Notably, all *N* ^9^‐haloalkyl compounds exhibited greater potency in inhibiting cancer cell growth compared to their corresponding *N* ^9^‐alkyl analogs. The cytotoxic potency of *N* ^9^‐substituted derivatives was found to correlate with the length of the halogenated alkyl chain, following the decreasing order: 6‐bromohexyl > 5‐bromopentyl > 4‐bromobutyl. Moreover, *N* ^9^‐substitution with a 4‐chlorobutyl group resulted in lower cytotoxic activity compared to the 4‐bromobutyl analog in MCF‐7 and SMMC‐7721 cell lines, whereas the opposite trend was observed in SGC‐7901 and A549 cells, where the chlorinated derivative exhibited greater activity. In contrast to the other determined cell lines (SGC‐7901, SMMC‐7721, and A549), MCF‐7 cells exhibited sensitivity exclusively to *N* ^9^‐haloalkyl‐substituted derivatives, while demonstrating resistance (IC_50_ > 50 µM) to their corresponding simple aliphatic alkyl analogs [46, 49].

Based on the findings of cytotoxicity screening, MOLT‐4 ALL cells were selected as a model for further investigation, as they exhibited an optimal balance between resistance and responsiveness to compound **6** treatment, along with pronounced selectivity toward malignant cells. Bearing in mind that p53 is a nuclear transcription factor that accumulates in response to cellular stress, including DNA damage and oncogene activation. This triggers transcriptional transactivation of p53 target genes such as p21, Bax, leading to cell cycle arrest, senescence, and/or apoptosis [32]. Since **1** was identified as an activator of the p53 pathway, which is also able to modulate follow‐up downstream signaling [50], we decided to preferably use in mechanistic experiments ALL MOLT‐4 cells, which are p53 wild‐type, over ALL Jurkat cells, which are p53 mutant E6.1.

### 2.4 Evaluation of the Inhibitory Potential of Compound 6 on FLT3‐ITD

Given that the indole scaffold is recognized as an important pharmacophore in FLT3 inhibitors, we evaluated the inhibitory effects of derivative **6** on AML cell lines harboring FLT3‐ITD mutations (MV4‐11 and MOLM‐13) as well as on wild‐type FLT3 cells (THP‐1), to evaluate its potential as a therapeutic strategy for targeting AML. Compound **6** shows cytotoxic activity to AML cells with the IC_50_ values ranging from 2.17 µM to 3.53 µM as obtained in three AML cell lines (Table 4). Since the cytotoxic effect in THP‐1 cells does not differ from that in the cell lines bearing the ITD mutation of the FLT3 gene (MV4‐11 and MOLM‐13), we suggest that another mechanism of action than inhibition of FLT3‐ITD is responsible for the cytotoxicity of this compound **6** analog.

**Table 4:** The IC_50_ values of compound 6 in FLT3‐ITD (MV4‐11 and MOLM‐13) and FLT3 wildtype (THP‐1) AML cell lines after 48 h of treatment.^a^ ^,^ ^b^

Cell line	Compound 6	Specificity ratio	
MV4‐11	3.53 ± 0.20	IC_50_ (THP‐1)/IC_50_ (MV4‐11)	IC_50_ (THP‐1)/IC_50_ (MOLM‐13)
MOLM‐13	2.98 ± 0.13		
THP‐1	2.17 ± 0.35	0.62	0.73

^a^ Results are expressed in µM.

^b^ All experiments were conducted in triplicate, and the data are presented as mean values ± SD of at least three independent replications.

### 2.5 Compound 6 Dose‐Dependently Inhibited Proliferation of Solid Tumor Cell Lines With Minimal Impact on the Non‐Cancer MRC‐5 Fibroblasts During Real‐Time xCELLigence Monitoring

In the next step, we evaluated the effects of **1** and compound **6** on cell adhesion, morphology, viability, and the proliferation of A549, MCF‐7, A2780, and MRC‐5 cells using the xCELLigence RTCA system. This approach offers the advantage of continuously monitoring cellular responses without requiring exogenous labeling.

**Figure 1:**
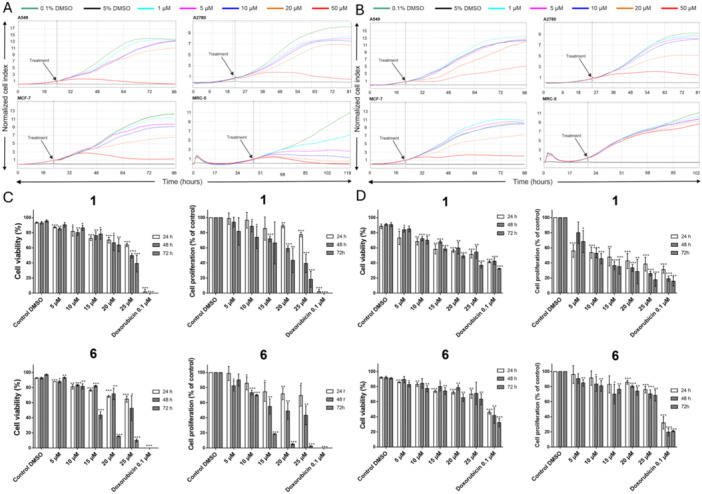
Effects of compounds **1** and **6** on the viability and proliferation. Real‐time cell proliferation of A549, MCF‐7, A2780, and MRC‐5 cells following exposure to (A) **1** and (B) **6** was monitored using the xCELLigence RTCA electrical impedance system. Cells treated with 0.1% DMSO served as vehicle control, while 5% DMSO‐treated cells were used as the positive control for cytotoxicity. The plots shown are representative of at least three independent replicate experiments. (C) MOLT‐4 ALL and (D) MRC‐5 lung fibroblast cells were incubated for 24, 48, and 72 h in the absence (control) or presence of different concentrations of 5–25 µM of **1** or compound **6**. Following treatments, viability and proliferation were determined by cell counting using the Trypan blue exclusion. At least three independent experiments were carried out. Cells treated with 0.1 μM doxorubicin and 0.1% DMSO were used as the positive and negative controls, respectively. Proliferation is depicted as the percentage of proliferating cells with respect to those determined in the 0.1% DMSO vehicle controls, which was considered to be 100%. Data are expressed as mean ± SD of experiments performed in triplicate (*n* = 3). Statistical significance compared to the negative control was determined using a *t*‐test. Significant differences were considered when *p < 0.1, **p < 0.01, ***p < 0.001.

From the proliferation curves shown in Figure 1A, it can be observed that **1** affects the proliferation of all the determined cell lines in a less or more graduate concentration‐dependent manner. As illustrated in Figure 1B, incubating cells with 20 and 50 µM of compound **6** for 72 h resulted in complete growth inhibition in A2780 cells, partial inhibition in MCF‐7 and A549 cells, but had no considerable effect on MRC‐5 cells. Therefore, based on the xCELLigence RTCA analysis results, we can further corroborate that derivative **6** exhibits a certain degree of selective cytotoxicity toward cancer cells. This selective growth‐inhibitory activity shows that compound **6** has better selectivity for malignant cells while sparing non‐cancerous cells. In addition, the results indicate that **1** has much more effect on the proliferation of the non‐cancerous MRC‐5 cell line than compound **6**. These findings are in accordance with our findings from the cytotoxic activity screening and IC_50_ values, confirming the selective cytotoxicity of compound **6** and promise as a targeted anticancer drug and its therapeutic potential.

### 2.6 Compound 6 Exhibits Enhanced Antiproliferative Activity Against MOLT‐4 Cells, While Exerting a Lesser Impact on the Viability and Proliferation of MRC‐5 Cells

Furthermore, the response of MOLT‐4 and MRC‐5 cells to **1** and compound **6** was assessed by determining the viability and proliferation at 24, 48, and 72 h after treatment with doses ranging from 5 to 25 µM. The inhibitory effect of the treatment was assessed using Trypan blue staining, which relies on the integrity of the cell membrane. Viable cells exclude this diazo dye, while nonviable cells take up the dye, are stained, and appear blue under the light microscope. Viability and percentage proliferation versus controls were determined by optical microscopy by counting the number of stained (dead) and unstained (live) cells.

The cell viability evaluation revealed that both **1** and compound **6** showed a dose‐ and time‐dependent inhibitory impact on MOLT‐4 cells. Derivative **6** showed higher antiproliferative activity than its parent compound **1**. Notably, the 72 h proliferation rates of **1** were 67% at 15 μM, 43% at 20 μM, and 18% at 25 μM, respectively. In contrast, derivative **6** exhibited stronger antiproliferative activity in the longer 72 h interval of exposure, where MOLT‐4 proliferation rates decreased to 19%, 5%, and 3% at the dosage of 15, 20, and 25 µM, respectively. This time‐dependent inhibitory effect on ALL cells was also evident in cell survival at 72 h following **1** exposure, with MOLT‐4 viability decreasing to 78% at 15 μM, 64% at 20 μM, and 39% at 25 μM, consistent with the proliferation data. However, compound **6**‐treated MOLT‐4 cells exhibited a higher reduction in viability within 72 h, reaching 44% (15 μM), 16% (20 μM), and 10% (25 μM) (Figure 1C). Compared to its effects on MOLT‐4 cells, compound **6** exhibited significantly lower cytotoxicity toward MRC‐5 cells relative to **1**. After 72 h of treatment, at the concentration of 25 µM, **1** decreased MRC‐5 cell viability to 37%, whereas derivative **6** induced a more moderate reduction, maintaining viability at 63% at the same concentration. The disparity was more pronounced in terms of cell proliferation, particularly at higher concentrations of 15 µM, 20 µM, and 25 µM after 72 h. Alkaloid **1** decreased cell proliferation to 35% (15 µM), 29% (20 µM), and 18% (25 µM), respectively, while compound **6** led to a comparatively mild reduction to 76% (15 µM), 74% (20 µM), and 68% (25 µM) at the same concentrations (Figure 1D).

### 2.7 Compound 6 Induces Cell Cycle Accumulation in G1 Phase

**Figure 2:**
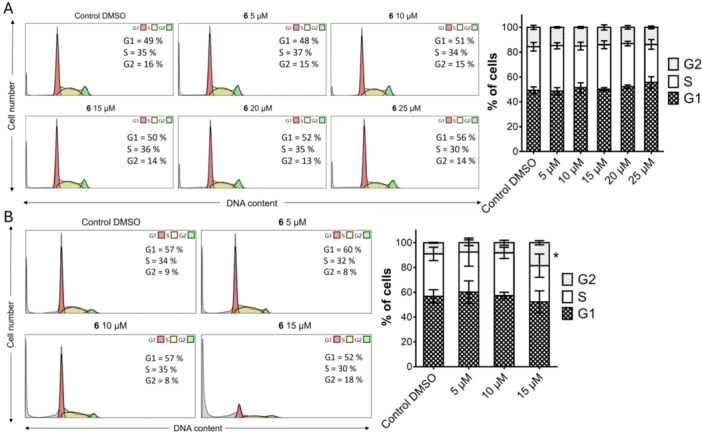
Effects of compound **6** on cell cycle progression. MOLT‐4 cells were treated with compound **6** (5–25 µM) or 0.1% DMSO as a control and incubated for (A) 24 h and (B) 48 h, followed by analysis of cell cycle distribution. Representative histograms from one of the independent experiments demonstrate the mean (%) distribution of cells across the G1, S, and G2 phases. Bar graphs present the cumulative percentages of MOLT‐4 cells in each phase of the cell cycle. Results are expressed as mean ± SD from at least three independent experiments. Statistical significance compared to the negative control was determined using a *t*‐test and is denoted as *p < 0.1, **p < 0.01, ***p < 0.001.

One of the strategies of treating cancer with chemotherapeutic agents is to reduce tumor burden by inducing cytostatic effects. Such treatments can disrupt the progression of the cell cycle, leading to the onset of cell death in cancer cells. Since the Trypan blue evaluation revealed that compound **6** showed a more pronounced inhibitory impact on MOLT‐4 ALL cells, we determined the distribution of the cell population into cell cycle phases. After exposure to compound **6** at different concentrations and time, the MOLT‐4 cells showed an altered distribution of cells at different stages of the cell cycle compared to the distribution of 0.1% DMSO vehicle control. After 24 h of incubation with derivative **6**, there was an increase in the percentage of cells in the G1 phase, accompanied by a decrease in cells in the S phase, following a dose‐dependent response. Upon treatment with 5, 10, 15, 20, and 25 μM of compound **6** for 24 h, the population of MOLT‐4 cells in G1 phase changed from 49% (0.1% DMSO control cells) to 48%, 51%, 50%, 52%, and 56%, respectively (Figure 2A). Comparable findings were observed at 48 h, where exposure to 5, 10, and 15 µM of compound **6** resulted in G1 phase cell populations of 60%, 57%, and 52%, compared to 57% in the 0.1% DMSO control cells group. Furthermore, treatment with compound **6** at concentrations of 20 and 25 µM resulted in an insufficient number of cycling MOLT‐4 cells for further analysis. This finding correlates with the increased sub‐G1 cell population and the results of the Annexin V/PI apoptosis assay (Figure 2B). Consistent with cytotoxicity screening results, IC_50_ values, xCELLigence analysis, and Trypan blue staining, treatment with compound **6** did not alter the distribution of MRC‐5 cells across the different phases of the cell cycle (Figure S4).

Parent alkaloid **1** has been shown to impair proliferation and induce apoptosis in diverse cancer cell models; however, scanty information is reported on anti‐ALL activity of **1** or its semisynthetic derivatives. Shen et al. (2018) [51] reported that **1** induces G1/S phase cell cycle arrest in A549 non‐small cell lung cancer (NSCLC) cells. Their flow cytometry analysis demonstrated a dose‐dependent accumulation of cells in the G1 phase. Building on these observations, is the reason why we utilized flow cytometry to investigate the effects of compound **6** on the cell cycle in MOLT‐4 cells. As shown, our results revealed that after 24 h of treatment, there was an increased proportion of cells in the G1 phase, accompanied by a reduction in the S phase population. After 48 h, these effects persisted, along with a notable rise in the sub‐G1 population, suggesting the potential of the derivative to induce apoptosis.

### 2.8 Compound 6 Triggers Apoptosis With DNA Fragmentation and Caspase Activation in MOLT‐4 Cells

**Figure 3:**
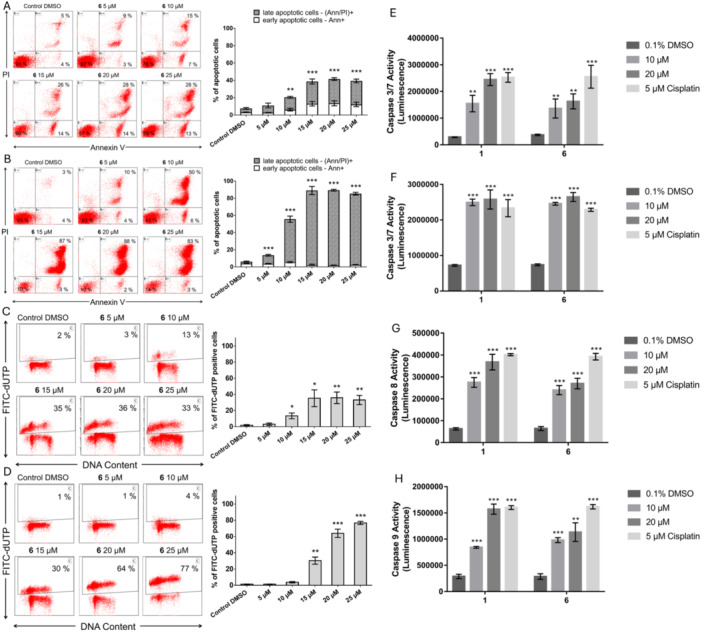
Effect of compound **6** exposure on apoptosis induction. The induction of apoptosis was evaluated using Annexin V/PI staining at (A) 24 and (B) 48 h post‐treatment. Flow cytometry histograms, representative of one of the independent experiments, display the mean percentage of cells in various quadrants based on the incorporation of Annexin V and/or propidium iodide (PI). Bar graphs present the cumulative data on the percentages of MOLT‐4 cells at various stages of apoptosis (mean ± SD, *n* = 3). The effect of compound **6** on the DNA fragmentation in MOLT‐4 leukemic cells at (C) 24 h and (D) 48 h after treatment with doses ranging from 5 to 25 μM. The quantitative data from flow cytometry are presented as the top panel of histograms, which show the percentage of cells with a DNA strand breaks terminal deoxynucleotidyl transferase‐mediated FITC‐dUTP nick end‐labeling. Representative dot plots of one of three independent measurements are shown. The bar graph displays the percentage of cells that are TUNEL positive (mean ± SD, *n* = 3). (E) Caspase‐3/7 activity in MOLT‐4 cells following 24‐h treatment with **1** and compound **6** at concentrations of 10 and 20 µM. (F) Caspase‐3/7 activity after 48‐h treatment. (G) Caspase‐8 activity in MOLT‐4 cells after 24‐h exposure to **1** and compound **6** at 10 and 20 µM. (H) Caspase‐9 activity in MOLT‐4 cells following 24‐h treatment with **1** and compound **6** at 10 and 20 µM. Cells treated with 5 µM cisplatin and 0.1% DMSO served as positive and negative controls, respectively. Data represent the mean ± SD from at least three independent experiments. Significance levels were denoted as *p < 0.05, **p < 0.01, and ***p < 0.001, relative to values observed in the respective DMSO vehicle‐treated control group.

The results demonstrated a significant decline in MOLT‐4 cell viability after compound **6** treatment. Following this observation, we used flow cytometry to assess apoptosis induction in MOLT‐4 cells through Annexin V/PI staining at 24 and 48 h post‐treatment. As shown in Figure 3A, flow cytometric analysis indicated that treatment with compound **6** increased the fraction of Annexin V‐positive and PI‐negative cells, particularly at higher concentrations (15, 20, and 25 µM) and at the shorter 24 h interval of exposure. After 48 h of treatment with 10–25 µM of **6**, a notable increase in the percentage of late apoptotic cells (Annexin V‐positive, PI‐positive) was observed, along with a significant reduction in MOLT‐4 cell viability (Figure 3B).

Moreover, compound **6**‐induced apoptotic DNA fragmentation in MOLT‐4 cells was further measured by TUNEL assay. To explore whether apoptosis activation was associated with DNA fragmentation, we treated MOLT‐4 cells with compound **6** at concentrations ranging from 5 to 25 µM for 24 and 48 h, followed by TUNEL flow cytometry analysis. While control cells showed no considerable FITC‐dUTP labeling, after compound **6** treatment, a strong TUNEL dUTP fluorescence signal was detected. The results indicate that treatment with derivative **6** at concentrations of 15, 20, and 25 µM considerably induced DNA fragmentation and apoptosis in a time‐dependent manner. Therewith, the MOLT‐4 cells did not respond in a dose‐dependent manner, showing approximately 35% of FITC‐dUTP positive cells across all these tested concentrations (15, 20, and 25 µM) of compound **6** after a 24‐h treatment period (Figure 3C). In contrast, at a 48‐h interval of exposure, the level of cells with DNA fragmentation and consequently apoptosis reaches 64% and 77% for 20 and 25 µM of compound **6**, respectively (Figure 3D). Taken together, the results found in the DNA fragmentation TUNEL experiments parallel the previous Annexin V/PI findings, further supporting the apoptosis‐inducing activity of compound **6**. Previous studies reported that compound **1** can induce significant structural DNA damage by binding to and interacting with DNA [3]. Spectrometric analyses, molecular simulations, and the comet assay have demonstrated that **1** intercalates into DNA base pairs, leading to DNA fragmentation [52]. Our findings demonstrated a time‐dependent increase in DNA fragmentation following treatment with compound **6**, with 48 h of exposure at 25 µM resulting in up to 77% of cells testing positive for FITC‐dUTP incorporation. These findings align with the late apoptotic events observed in Annexin V/PI staining, reinforcing the hypothesis that compound **6** promotes apoptosis by inducing DNA breaks.

Earlier research has extensively established the ability of **1** to activate apoptotic pathways, as shown by Hamsa and Kuttan (2011) [32]. Their research showed that **1** downregulated Bcl‐2 expression in B16F‐10 melanoma cells while promoting apoptosis through the upregulation and activation of Bax, caspase‐3, ‐8, ‐9, and Bid. Additionally, nuclear staining revealed morphological features characteristic of apoptosis, including membrane blebbing, chromatin condensation, DNA fragmentation, and the formation of apoptotic bodies [32]. To explore the proapoptotic effects of **1** and compound **6** on MOLT‐4 cells, we assessed caspase activity. Extrinsic signaling pathways, initiated at the plasma membrane, are characterized by the activation of caspase‐8, whereas the intrinsic pathway, which is mitochondrial‐dependent, leads to the activation of caspase‐9. Effector caspases‐3 and ‐7 are activated downstream of the apical initiator caspases and serve as central mediators in both the extrinsic and intrinsic apoptotic pathways. To identify the pathways activated by **1** and compound **6** (10 and 20 µM), we assessed the activity of caspases‐8, ‐9, and ‐3/7 at a 24‐h interval post‐treatment. To assess whether the increase in caspase activity persists or increases over time, we also evaluated caspase‐3/7 activity 48 h post‐treatment. The findings demonstrated that both **1** and compound **6** significantly increased caspase‐3/7 activity in a dose‐dependent manner within 24 h of treatment. Treatment with 10 µM of **1** or **6** for 24 h led to a substantial increase in caspase activity compared to the negative control 0.1% DMSO. Notably, at a concentration of 20 µM, **1** induced caspase‐3/7 activation comparable to that observed in 5 µM cisplatin‐treated cells (Figure 3E). Extending the treatment period to 48 h revealed sustained caspase‐3/7 activity in both **1** and compound **6** treatment groups, with compound **6** exhibiting caspase activity comparable to **1**. This prolonged activation indicates that **1** and derivative **6** not only initiate apoptosis but also maintain apoptotic signaling pathways. The persistence of caspase activity suggests that compound **6** has the potential to induce prolonged cell death in MOLT‐4 leukemic cells (Figure 3F). Following 24 h of treatment with compound **6** and **1**, caspase‐8 activity increased considerably (*p* < 0.001) in a dose‐dependent manner. This observation is especially significant since it implies that both parent **1** and compound **6** can trigger the extrinsic apoptotic pathway (Figure 3G). After 24 h of treatment, both **1** and compound **6** markedly elevated caspase‐9 activity, highlighting the involvement of the intrinsic apoptotic pathway. Interestingly, the increase in caspase‐9 activity induced by **1** was more dose‐dependent compared to that observed with compound **6** (Figure 3H). To sum up, the ability of **1** and compound **6** to activate both the extrinsic (caspase‐8) and intrinsic (caspase‐9) pathways highlights their strong proapoptotic properties. While **1** demonstrated higher initial efficacy in triggering apoptotic pathways, compound **6** exhibited comparable caspase‐3/7 activity to **1** after 48 h, underscoring its sustained proapoptotic effects. Furthermore, derivative **6** proved more effective than **1** in reducing cell viability and suppressing proliferation in our studies. These findings suggest that compound **6** may exert its inhibitory effects on cancer cells through additional mechanisms beyond apoptosis, such as decelerating cell cycle progression.

### 2.9 Both Compound 6 and Parent 1 Elevate the Formation of DNA Damage Markers PAR and γH2AX

**Figure 4:**
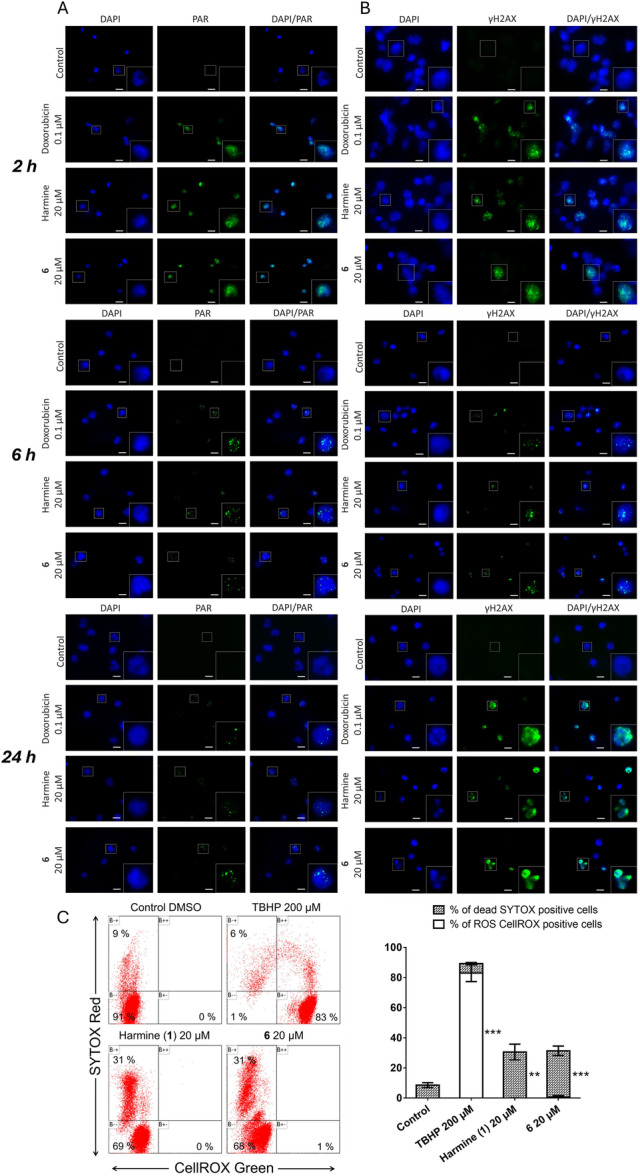
Effects of **1** and compound **6** on DNA damage in MOLT‐4 cells. (A) Immunofluorescence images showing the formation of poly(ADP‐ribose) (PAR) in cells treated with 0.1% DMSO vehicle control, 0.1 μM doxorubicin, 20 μM of **1**, or 20 μM of compound **6**, detected 2, 6, and 24 h after exposure. The left panels display blue DAPI fluorescence of the nucleus, the middle panels show green Alexa Fluor 488 fluorescence labeling PAR, and the right panels present an overlay of DAPI and PAR. (B) Immunofluorescence γH2AX staining showing γH2AX foci formation 2, 6, and 24 h after exposure to 0.1% DMSO vehicle control, **1**, or compound **6** at 20 μM compared with 0.1 μM doxorubicin‐treated positive control. In the left panel of images, cell nuclei are labeled with DAPI; in the middle, γH2AX is stained with immunofluorescence using Alexa Fluor 488; and in the right images, the DAPI and γH2AX are merged. Higher magnifications of selected cell nuclei are shown in the bottom right corner. The images presented are representative of experiments independently repeated at least three times. Scale bar: 10 μm. (C) ROS generation in MOLT‐4 cells was assessed 24 h after treatment with 20 µM of **1** or **6**. As a positive control, 200 µM tert‐butyl hydroperoxide (TBHP), a well‐established ROS inducer, was used. Representative flow cytometry histograms illustrate fluorescence signals from CellROX Green and SYTOX Red Dead Cell Stain, accompanied by bar graphs summarizing cumulative data on the percentage of ROS (CellROX)‐positive cells and dead (SYTOX)‐positive cells. Quantitative data represent the mean ± SD from at least three independent experiments. Statistical significance was assessed using a *t*‐test, with *p < 0.05, **p < 0.01, and ***p < 0.001.

Previous studies have shown that **1** inhibits DNA topoisomerase I activity [53], leading to DNA damage that ultimately results in suppressed proliferation and death of cancer cells in vitro [52]. Both topoisomerase I and topoisomerase II serve as enzyme targets for clinically used chemotherapeutic agents in the treatment of many cancer types or desirable molecular target for new drug discovery efforts. Type I topoisomerase inhibitors stabilize the DNA single‐strand breaks (SSBs) initially induced by topoisomerase I, whereas type II topoisomerase inhibitors stabilize the DNA double‐strand breaks (DSBs) introduced by topoisomerase II [54]. To investigate whether **1** and compound **6** promote inhibitory effects against leukemic cells by introducing SSBs and DSBs in nuclear DNA, the immunofluorescence visualization of poly(ADP‐ribose) (PAR) and γH2AX was carried out after 2, 6, and 24 h of exposure. In DNA‐damaged cells, binding and dimerization of poly(ADP‐ribose) polymerases (PARPs) at sites of SSBs triggers elongation and transfer of long branched chains of PAR onto various nuclear acceptors [55]. Therefore, to investigate PARP activation, we assessed PAR polymer formation through immunofluorescence analysis. As demonstrated by epi‐fluorescence imaging in Figure 4A, the level of PAR formation within the nuclei of MOLT‐4 cells was considerably higher following 2‐h treatment with 20 µM of **1** or compound **6** compared to the sham control containing 0.1% DMSO. Consistent with previous findings [56], we observed PARylation following doxorubicin treatment at the 2 h interval. In certain cell nuclei, a distinct PAR signal remains detectable at both 6 and 24 h post‐treatment. Phosphorylated histone H2AX at serine 139 (γH2AX) serves as a sensitive indicator of DSBs, with its levels positively correlating with the extent and kinetics of DNA DSBs formation and repair [57]. As illustrated in Figure 4B, cells exposed to 20 µM of **1** or compound **6** for 2 h exhibited dispersed, pan‐nuclear marks of staining with γH2AX green immunofluorescence, typically associated with extensive DNA damage, in contrast to the negative control group. At a later time point, 6 h post‐treatment, distinct, accumulated, persisting nuclear γH2AX foci became evident. Following an extended 24‐h incubation with **1** or compound **6**, fragmented, blue‐stained nuclei were observed, along with a secondary increase in γH2AX staining associated with apoptotic cells. Notably, parallel treatment with 0.1 μM doxorubicin used as a positive control and targeting topoisomerase II resulted in an anticipated increase in the frequency of γH2AX foci within the nuclei of MOLT‐4 cells.

Consistent with our observations, a study done by Zhao and Wink (2013) [34] demonstrated that treatment with **1** led to an increase in γH2AX levels in MCF‐7 breast cancer cells, indicating the presence of DSBs. Given that DNA damage is a key mechanism of action for many clinically established antileukemic drugs, such as doxorubicin, we conducted immunofluorescence analysis of genotoxic stress markers after a 2, 6, and 24‐h treatment period. Specifically, we examined poly(ADP‐ribose) (PAR) and γH2AX levels to assess whether **1** and its derivative, **6**, induce DNA damage in leukemic cells, including both SSBs and DSBs. Our immunofluorescence data showed a substantial increase in nuclear PAR production in MOLT‐4 cells treated for 2 h with 20 µM of either **1** or **6**. This rise in PAR levels suggests increased PARP activity, which implies the existence of SSBs. The phosphorylation of histone H2AX at serine 139, resulting in γH2AX, acts as a sensitive marker for DSBs, with accumulation corresponding with the degree of DNA damage [58]. Treatment with 20 µM of compound **1** or derivative **6** resulted in marked γH2AX staining in MOLT‐4 cells at the 2‐h time point, with a further increase observed after 24 h, indicating the generation of DSBs. These findings indicate that **1** and compound **6** can efficiently produce DNA damage in leukemic cells, activating repair pathways involved with SSBs and DSBs. The observed rise in PAR and γH2AX levels highlights the potential for these chemicals to damage DNA integrity, leading to their cytotoxic actions in cancer cells.

ROS has a counterintuitive role in anticancer therapy. Certain chemotherapy drugs, such as doxorubicin, stimulate ROS production, significantly enhancing their lethal impact on cancer cells. On the other side, elevated ROS levels can support a proproliferative, antiapoptotic, and metastatic phenotype of cancer cells [59]. Thus, ROS formation was analyzed by flow cytometry using the CellROX Green probe, which is nonfluorescent in its reduced state but emits a strong fluorescent signal upon oxidation. Simultaneously, SYTOX Red dead cell stain was applied to differentiate dead cells from those oxidatively stressed or cells remaining unstressed. As shown in Figure 4C, the results showed no increase in CellROX fluorescence after 24 h of treatment with 20 µM **1** or **6**. Consistent with previous cytotoxicity findings, the percentage of SYTOX‐positive cells increased to 31% following treatment with **1** or compound **6**, respectively. As expected, treatment with TBHP (a ROS inducer) for 24 h resulted in a significant increase (83%) in ROS‐associated fluorescence.

These findings indicate that compounds **1** and **6** induced DNA damage in MOLT‐4 cells without depending on increased ROS generation.

### 2.10 Compound 6 Targets the DNA Damage, Cell Cycle Checkpoint, and p38‐MAPK Signaling Pathway in MOLT‐4 Cells

**Figure 5:**
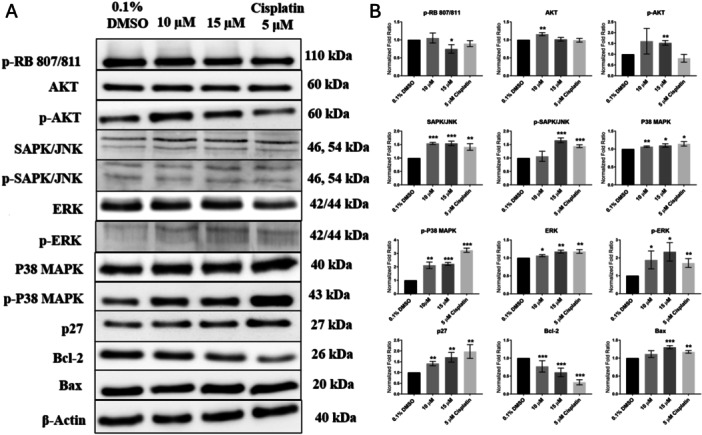
Effect of compound **6** treatment on the cell cycle regulatory and apoptosis‐related protein levels. (A) Western blot analysis of protein expression levels in MOLT‐4 cells was conducted 4 h following treatment with compound **6** at 10 and 15 µM. Cells treated with 0.1% DMSO were used as a negative control, while those treated with 5 µM cisplatin served as a positive control. (B) Quantitative densitometry was performed using ImageJ. Quantification plots are presented as mean ± SD from three independent experiments for each condition. Statistical significance was evaluated using a *t*‐test, with *p < 0.05, **p < 0.01, and ***p < 0.001.

Considering the proapoptotic and cell cycle‐perturbing effects of compound **6**, we employed Western blot analysis to assess its impact on key regulatory proteins involved in cell cycle progression and apoptosis. The Western blot analysis was conducted on lysed MOLT‐4 cells treated with 10 and 15 µM of compound **6** for 4 h to examine the levels of apoptosis‐related and cell cycle regulatory proteins. Cells treated with 0.1% DMSO served as negative controls, whereas cells treated with 5 µM cisplatin were used as positive controls to confirm the proapoptotic effect. As depicted in Figure 5, compound **6** treatment led to a considerable decrease in phosphorylated retinoblastoma protein (pRb phosphorylated at Ser807/811) levels compared to the 0.1% DMSO vehicle control. The reduction was most pronounced (*p* < 0.05) at the 15 µM concentration, indicating a notable suppression of cell cycle progression, likely occurring in the G1 phase. The retinoblastoma protein (pRb) functions as a cell cycle regulator by controlling G1 to S phase transition and is also involved in crucial roles involving tumor suppression [60]. Our findings demonstrated a reduction in phosphorylated retinoblastoma (p‐Rb) protein levels compared to the negative control. Since p‐Rb plays a critical role in promoting cell cycle transition from the G1 to S phase, its downregulation suggests that compound **6** induces G1/S phase cell cycle arrest. Concurrently, a dose‐dependent significant (*p* < 0.01) increase in the protein levels of p27, a cyclin‐dependent kinase inhibitor, was observed. The upregulation of p27 reinforces the finding that compound **6** disrupts cell cycle progression, as p27 inhibits cyclin‐CDK complexes, thereby blocking progression through the cell cycle. Similarly, in a study conducted by Liu et al. (2016) [31], **1** was shown to upregulate p27, which sequentially boosted its binding to cyclin‐CDK complexes, causing an inhibition of their activity and leading to G1 phase cell cycle arrest in human breast cancer MCF‐7 cells. Collectively, these results provide compelling evidence that compound **6** exerts its effects by inducing G1 phase arrest, which may contribute to its observed antiproliferative properties. AKT signaling is a key regulator of cell proliferation [61], and **1** has been shown to inhibit p‐Akt (Thr308) level in human colorectal carcinoma SW620 cells [31]. Currently, treatment with compound **6** for 4 h at both 10 and 15 µM did not result in any significant change in AKT protein levels in MOLT‐4 cells. In contrast, the levels of phosphorylated AKT at Thr308 (pAKT) exhibited a dose‐dependent biphasic response; at 10 µM, pAKT levels were higher compared to the negative control, but a notable decrease was observed at the 15 µM concentration. This bimodal activity suggests that lower doses of compound **6** may enhance survival signaling through the AKT pathway, whereas higher concentrations may inhibit this signaling, leading to reduced cell survival and proliferation. The MAPK signaling pathway plays a critical role in regulating various cellular processes, such as cell proliferation and apoptosis. Within the MAPK family, p38 and SAPK/JNK are recognized for their role in tumor suppression by promoting apoptotic cell death in cancer cells. Our findings demonstrated an upregulation of phosphorylated p38 MAPK (p‐p38) and SAPK/JNK following compound **6** treatment. The phosphorylation levels of p‐p38 were comparable at both 10 and 15 µM concentrations, while SAPK/JNK showed a significant increase at 15 µM of compound **6**, indicating a strong activation of stress response signaling. Total ERK levels exhibited a slight increase; however, phosphorylated ERK (p‐ERK) was more prominently upregulated at both 10 and 15 µM of compound **6**, potentially reflecting a compensatory survival mechanism, as ERK signaling is associated with cell survival and proliferation. Given that ERK activation is commonly linked to cell growth and survival, while p38 MAPK and SAPK/JNK pathways predominantly mediate proapoptotic responses, the balance of these signaling cascades may ultimately dictate the fate of compound **6**‐treated cells. Consistent with our findings, Ock et al. (2021) [62] reported that **1** hydrochloride interferes with the cell cycle progression of breast cancer cells by modulating MAPK and AKT signaling pathways, suggesting that the inhibitory effect of **1** may be mediated through these pathways. These results indicate that both compounds **6** and **1** may promote apoptotic cell death via activation of stress‐responsive MAPK pathways. Furthermore, **1** has been reported to significantly upregulate the level of proapoptotic protein Bax while suppressing the anti‐apoptotic protein Bcl‐2 in colon cancer Caco‐2 cells [63]. Our Western blot analysis similarly revealed that compound **6** significantly modulates apoptotic regulators, inducing a dose‐dependent decrease in Bcl‐2 expression while upregulating Bax, reinforcing its proapoptotic role. Moreover, we noticed that compound **6** significantly activated caspase‐9, a key mediator of intrinsic apoptosis induced by DNA damage. Bax acts as a stress sensor, promoting mitochondrial outer membrane permeabilization (MOMP) and releasing proapoptotic factors like cytochrome c into the cytosol. Compared to the control group, compound **6** treatment upregulated (*p* < 0.001) the proapoptotic protein Bax while dose‐dependently reducing (*p* < 0.001) the levels of the anti‐apoptotic protein Bcl‐2. This change in the Bcl‐2/Bax ratio toward proapoptotic signaling highlights the enhanced potential of the harmine derivative to drive cells toward apoptosis.

### 2.11 Evaluation of the Inhibitory Activity of Compound 6 on Monoamine Oxidase

Previous studies have indicated that the anticancer potential of **1** may be attenuated by its off‐target inhibition of MAO‐A, an enzyme responsible for the degradation and reuptake of monoamines, including serotonin and norepinephrine, thereby affecting multiple signaling pathways within the nervous system [64, 65]. Inhibition of MAO‐A is not fundamentally negative, as selective MAO‐A inhibitors, such as phenelzine and isocarboxazid, are clinically approved and used for depression and other neuropsychiatric disorders. Nonetheless, its suppression may lead to an excessive accumulation of synaptic monoamines, potentially causing manifestations such as agitation, flushing, tachycardia, palpitations, muscular twitching, hyperreflexia, and even seizures [66]. Moreover, MAO‐A inhibition constitutes an important safety concern when evaluating these compounds as anticancer agents, especially in patients undergoing treatment with multiple serotonergic or sympathomimetic medications, due to an increased likelihood of serotonin toxicity when used in conjunction with other serotonergic drugs [67]. Since the neurotoxicity of compound **1**, resulting from undesirable competitive inhibition of MAO‐A, has been a major obstacle to its possible clinical use in anticancer therapy, we assessed the in vitro inhibitory potential of the newly synthesized and most potent molecule, derivative **6**, against human MAO‐A. The parent compound, **1**, exhibited potent MAO‐A inhibitory activity, with an IC_50_ value of 0.08 ± 0.01 µM. In contrast, compound **6** demonstrated only mild MAO‐A inhibition, with an IC_50_ value of 254.40 ± 33.89 µM (Table 5). Considering that the IC_50_ values for cytotoxic activity in the MOLT‐4 ALL model were in the low micromolar range, while the MAO‐A inhibitory IC_50_ value of compound **6** was in the high micromolar range, these results suggest a favorable therapeutic window, potentially reducing the likelihood of dose‐limiting neurotoxicity.

**Table 5:** The IC_50_ values of compounds 1 and 6 against human MAO‐A.^a^

Compound	MAO‐A
**1**	0.08 ± 0.01 µM
**6**	254.40 ± 33.89 µM

^a^ All experiments were conducted in triplicate, and the data are presented as mean values ± SD of at least three independent replications.

Aiming at potential as an anticancer candidate, compound **6** showed low off‐target affinity for MAO‐A, which overcomes the disadvantages of **1**‐induced neurotoxicity. This is consistent with previous reports showing that substitution at the *N* ^9^ position of **1** modulates its inhibitory activity against MAO‐A. Structure‐based studies by Bálint and colleagues demonstrated that increasing the size of the substituent leads to a marked reduction in MAO‐A inhibitory activity, with the benzyl derivative being among the least potent [45]. Accordingly, computational analyses confirmed that benzyl substitution at the *N* ^9^ position retains strong antitumor activity while exhibiting low neurotoxicity [68]. Although comprehensive studies directly evaluating the effects of **1** in ALL models are currently lacking, **1** has been recognized for its anticancer activity in other leukemia cell lineages. In the context of AML, **1** has been shown to downregulate DNA methyltransferase 1 (DNMT1) expression in NB4 leukemia cells, resulting in the reactivation of tumor suppressor genes and subsequent inhibition of leukemic cell proliferation [69]. Furthermore, **1** has demonstrated cytotoxic activity against acute promyelocytic HL‐60 and chronic myelogenous K‐562 leukemia cell lines [70]. Evidently, these findings suggest that **1** may hold therapeutic potential for leukemia treatment. In our study, both compound **1** and its derivative **6** showed potent anticancer activity against MOLT‐4 cells, with compound **6** additionally exhibiting a reduced inhibitory effect on non‐cancerous MRC‐5 cells, suggesting selective cytotoxicity.

### 2.12 ADME Prediction, Drug‐Likeness, and Toxicity

In the context of drug discovery, an optimal drug candidate should integrate potent and selective pharmacological activity with favorable absorption, distribution, metabolism, and excretion (ADME) properties that support clinical potential. To further characterize drug‐like properties of **1** and its derivatives that demonstrated antiproliferative activity between 0% and 50% (**1**, **3**, **6**, **8**, **9**, **10**, **14**, **21**, **25**, **27**, **32**, and **33**), an *in silico* pharmacokinetic assessment was conducted using the SwissADME, which is a freely accessible online tool [71]. Table 6 provides a summary of the ADME and physicochemical parameters, including molecular weight (Mw), rotatable bonds (rot. bonds), hydrogen bond donor (HDD) and hydrogen bond acceptor (HBA) count, partition coefficient octanol‐water (cLog*P*_*O*/W_), topological polar surface area (TPSA), and water solubility (solubility ESOL). The analysis showed that compound **1** and all other evaluated derivatives, apart from compound **8**, met Lipinski's rule of five, indicating favorable drug‐like characteristics. In addition, the rules of Veber are followed by all the compounds, with less than 10 rotatable bonds and a topological polar surface area (TPSA) below 140 Å^2^. These properties are predictive of good gastrointestinal absorption and suggest suitability for oral administration [72]. Water solubility, a key parameter in the pharmacokinetic profiling of preclinical drug candidates, was evaluated using estimated aqueous solubility (ESOL) predictions. All compounds except compound **8** were classified as soluble or moderately soluble. Furthermore, all the compounds other than **8** have a balanced hydrophilic‐lipophilic profile, according to our data, with a consensus log octanol/water partition coefficient (cLogP_O/W_) less than 5. The compounds were also assessed for their potential interaction with P‐glycoprotein (P‐gp), an efflux transporter known to affect drug bioavailability by promoting excretion. Most compounds were predicted to be substrates of P‐gp, although three (**1**, **21**, and **27**) were identified as not susceptible to P‐gp‐mediated efflux, suggesting potentially improved bioavailability. Importantly, all tested compounds were predicted to cross the blood–brain barrier (BBB), indicating possible effects within the central nervous system or related side effects. To evaluate the safety profile, the compounds were further analyzed using ProTox 3.0 [73]. With the exception of compounds **14** and **21**, all tested molecules were classified within toxicity class 4, indicating that they are neither acutely toxic nor lethal, but may still present a moderate oral toxicity risk. Their predicted LD_50_ values, ranging from 300 to 2000 mg/kg, suggest that further pharmaceutical technology optimization will be necessary to further enhance their safety profiles for potential clinical application.

**Table 6:** Predicted drug‐like, physicochemical, and toxicity properties for compound with antiproliferative effects (0%–50%) against carcinoma cell lines using predictive models ADME Swiss and ProTox‐3.0.

Code	Mw	Lipinskis rule of five				Drug‐likeness	GI absorption		BBB permeant	Solubility ESOL		P‐gp	Predicted toxicity	
		Rot. bonds	HBA	HBD	cLog*P*_*O*/W_	LP violation	Class	TPSA		Class	mg/L		LD_50_ (mg/kg)	Class
**1**	212.25	1	2	1	2.78	Yes; 0	High	37.91	Yes	Moderately	19.0	No	500	4
**3**	316.40	3	2	0	4.18	Yes; 0	High	27.05	Yes	Moderately	2.59	Yes	850	4
**6**	330.42	3	2	0	4.52	Yes; 0	High	27.05	Yes	Moderately	1.38	Yes	850	4
**8**	358.48	4	2	0	5.07	Yes; 0	High	27.05	Yes	Poorly	0.325	Yes	900	4
**9**	332.40	4	3	0	3.84	Yes; 0	High	36.28	Yes	Moderately	4.68	Yes	700	4
**10**	362.42	5	4	0	3.82	Yes; 0	High	45.51	Yes	Moderately	4.45	Yes	850	4
**14**	336.81	3	2	0	4.32	Yes; 0	High	27.05	Yes	Moderately	1.41	Yes	907	2
**21**	347.37	4	4	0	3.05	Yes; 0	High	72.87	Yes	Moderately	5.16	No	230	3
**25**	254.33	3	2	0	3.25	Yes; 0	High	27.05	Yes	Soluble	31.2	Yes	500	4
**27**	252.31	3	2	0	3.15	Yes; 0	High	27.05	Yes	Soluble	36.3	No	500	4
**32**	347.25	5	2	0	3.88	Yes; 0	High	27.05	Yes	Moderately	7.38	Yes	787	4
**33**	361.28	6	2	0	4.17	Yes; 0	High	27.05	Yes	Moderately	4.55	Yes	1000	4
**Required**	≤ 500	≤ 10	≤ 10	≤ 5	≤ 5	—	—	≤ 140	—	—	—	—	—	—

*Note:* Calculated using SwissADME online server: Mw = molecular weight; rot. bonds = number of rotatable bonds, HBA = number of hydrogen acceptors; HBD = number of hydrogen donors; cLog P_O/W_ = consensus log octanol/water partition coefficient; LP violation = Lipinski's violation; GI absorption = gastrointestinal absorption; TPSA = total polar surface area in in Å^2^; BBB = blood–brain barrier permeation; solubility ESOL = estimated aqueous solubility; P‐gp = P‐glycoprotein substrate. Calculated using ProTox‐3.0 online server for oral toxicity prediction: LD_50_ = Lethal Dose for 50% of the tested population; Class = toxicity class.

Our physicochemical and pharmacokinetic *in silico* studies using SwissADME showed that compound **6** follows Lipinski's rule of five [74], has a good solubility of 1.38 mg/L, and has high gastrointestinal absorption. These findings indicate that compound **6** has the potential for enteral administration and may be developed as an oral drug candidate. It was also shown to possess the ability to cross the BBB, suggesting its potential to exert pharmacological effects within the central nervous system (CNS). Additionally, our findings suggest that this CNS activity can be achieved without off‐target inhibition of MAO‐A. The toxicity of compound **6** was also assessed using ToxPro 3.0, which predicted that derivative **6** has an LD_50_ dosage of 850 mg/kg and was placed under toxicity class 4. Class 4 toxicity corresponds to compounds with an LD_50_ between 300 and 2000 mg/kg, indicating potential harm if ingested, though not acutely lethal [73]. In a study by Mosaad et al. (2017) [75], Adriamycin (doxorubicin) had an LD_50_ of 56.875 mg/kg. This places it in Class 3 toxicity, but it is still routinely used in clinical practice, meaning compound **6,** which is in the toxicity Class 4, falls under the acceptably toxicity range to be acceptable for drug development and usage. The balanced pharmacokinetic and toxicity profile of compound **6** shows its potential as a pharmacological candidate, since it matches the desirable physicochemical features required for effective drug development.

## 3 Conclusions

In conclusion, this study brings new clues and original observations regarding the potential of β‐carboline alkaloids, particularly their newly prepared derivative **6**, as promising anti‐ALL agents. Compound **6**, modified at the *N* ^9^ position with a 3,5‐dimethylbenzyl group, demonstrated selective cytotoxicity, significantly reducing cancer cell growth while sparing non‐cancerous MRC‐5 cells. At a concentration of 10 µM, compound **6** exhibited pronounced cytotoxicity against Jurkat, MOLT‐4, and A2780 cell lines, resulting in an average cell growth of 60%, compared to 79% observed with compound **1**. IC_50_ values confirmed its selectivity, with cancer cell IC_50_ values below 10 µM compared to > 100 µM for MRC‐5 cells. Exposure of model MOLT‐4 ALL cells to compound **6** redistributed the cell cycle, increasing G1 phase populations and decreasing S phase cells, while also activating apoptosis pathways. Western blot analysis showed reduced phosphorylated retinoblastoma protein, increased p27 levels, and upregulation of proapoptotic Bax while suppressing anti‐apoptotic Bcl‐2. Additionally, derivative **6** caused DNA damage, as evidenced by increased poly(ADP‐ribose) (PAR) and γH2AX levels, indicating strand breaks and a negative impact on MOLT‐4 genomic DNA integrity. It should also be emphasized that compound **6** exhibited much lower inhibitory activity against MAO‐A compared to **1** and did not induce ROS production, both of which are associated with minimizing the risk of adverse effects. The integration of these properties positions compound **6** as a promising candidate for antileukemic therapy. However, further investigations are still necessary to validate its therapeutic potential and support its advancement toward application in medicine.

## 4 Experimental

### 4.1 Isolation and Chemical Synthesis

#### 4.1.1 General Experimental Procedures

All solvents were treated using standard laboratory techniques before use. Reagents (highest purity grade) were acquired from Merck (Czech Republic). Chromatography‐grade solvents were obtained from Penta Chemicals Unlimited (Czech Republic). The NMR spectra were recorded in CDCl₃ at ambient temperature using a VNMR S500 spectrometer (Varian) (500 MHz for ¹H, 126 MHz for ¹³C) and a JNM‐ECZ600R spectrometer (Jeol) (600 MHz for ¹H, 151 MHz for ¹³C). Chemical shifts (δ) are reported in parts per million (ppm) and were referenced indirectly to tetramethylsilane (TMS) using the residual solvent signal of chloroform‐d₁ (CDCl₃: δ 7.26 for ¹H, 77.0 for ¹³C). Coupling constants (J) are given in Hz. Chromatographic analysis and purity assessment were done with Waters Autopurification HPLC‐PDA‐MS system (Waters Corporation, Milford, Massachusetts, USA) in analytical mode. The system consisted of the Waters Sample Manager 2767, System Fluidics organizer, two Waters 515 HPLC pumps, Waters 2545 Binary Gradient module, Waters 2998 Photodiode array detector, and Waters Acquity qDa detector. All samples (0.2‐0.5 mg) were dissolved in 1 mL of MS grade methanol prior to analysis. As a mobile phase, water with 0.1% formic acid (solvent A) and methanol with 0.1% formic acid (solvent B) were employed. Analysis was done at ambient temperature using XSelect CSH C18 OBD reverse phase column (100 mm × 4.6 mm i.d., 5 µm) (Waters Corporation, Milford, Massachusetts, USA). The flow rate of the mobile phase was set to 0,8 mL/min. Linear gradient from 5% up to 100% of methanol (v/v) in 8,5 min, followed by 2 min of 100% methanol and 1.5 min at initial conditions for re‐equilibration was used. The detection range for the analysis of peak purity by UV was set from 190 to 450 nm with a sampling rate of 20 Hz. The optimum values of the ESI‐MS parameters were: capillary voltage – 0.8 kV; Probe temperature −600°C; Cone Voltage ‐ 15 V. LC/MS mass spectra were recorded across the range 100–800 m/z. LC ESI‐MS analyses were carried out in the positive ion mode. Analysis confirmed the purity of all the compounds > 95% (PDA detection, UV–vis, uncalibrated). ESI‐HRMS were obtained with a Waters Synapt G2‐Si hybrid mass analyzer of a quadrupole‐time‐of‐flight (Q‐TOF) type, coupled to a Waters Acquity I‐Class UHPLC system (Waters, Millford, MA, USA). Preparative separations were performed on a Sepacore Flash chromatography system X10 (Büchi Labortechnik, Flawil, Switzerland) consisting of two C‐605 pump modules, a C‐620 control unit, a C‐640 UV detector, and a C‐660 fraction collector. The system was controlled by the software SepacoreControl 1.4. Flash chromatography separation was performed on a glass column (49 × 460 mm, Büchi, Switzerland) packed with neutral alumina (32–63 μm, Alfa Aesar, ThermoFisher, Czech Republic). Solid introduction was performed by means of a polypropylene cartridge (40 × 75 mm, Büchi, Switzerland) connected to the top of the column. The extract was adsorbed on neutral alumina prior to introduction. Analytical TLC was carried out on Merck plates precoated with silica gel 60 F254, and preparative TLC was carried out on glass plates (15 × 15 cm) precoated with silica gel 60 GF254 (Merck, Darmstadt, Germany). Compounds on the plates were visualized under UV light (254 and 366 nm) and by spraying with Dragendorff's reagent.

#### 4.1.2 Plant material

*P. harmala* seeds (294 g) were purchased in a local market in Markazi province, Arak city (Iran) in June 2019. Pharmacognostical identification was performed by Prof. RNDr. Lubomír Opletal, CSc. A voucher specimen has been deposited at the Department of Pharmacognosy and Pharmaceutical Botany, Faculty of Pharmacy in Hradec Králové under the following code number AL‐692.

#### 4.1.3 Extraction and Isolation of **1**

Ground seeds of *P. harmala* were Soxhlet‐extracted successively with petrol ether and MeOH for 2 h. The petrol ether extract was discarded, and the MeOH extract was concentrated using a rotary vacuum evaporator at 40°C to a brown syrupy residue. The residue was dissolved in 5% HCl (2 L) and diluted with H_2_O to 4 L. The acidic aqueous solution was filtered through a Celite 535 layer, then subsequently alkalized with 10% Na_2_CO_3_ to pH 9–10 and extracted with CHCl_3_ (4 × 2 L). The organic phase was evaporated to obtain a summary alkaloid extract (26 g). The alkaloid extract was purified by LLE again, similarly as mentioned above, but the acidic aqueous layer was defatted by Et_2_O. After alkalization, the CHCl_3_ layer was dried using anhydrous Na_2_SO_4_ and evaporated under reduced pressure to obtain 18 g of the purified alkaloid extract.

The extract was fractionated by Flash chromatography on the glass column packed with neutral alumina (900 g) using a mobile phase containing solvent A (CH_2_Cl_2_:NH_4_OH; 100:0.2%) and solvent B (MeOH:NH_4_OH; 100:0.2%). First, the column was equilibrated with solvent A. The extract was separated using a 400 min program, starting with isocratic elution with 100% A (30 min), then linearly increased to 100% B (30–210 min), and 100% B held for 30 min. The flow rate was set at 50 mL/min, and individual fractions were collected by monitoring the eluting analytes at 254 nm, 274 nm, 321 nm, and 376 nm. Totally, 87 fractions were collected, which were combined into 5 fractions (A–E), based on analytical TLC. Subsequent preparative TLC of fractions A (982 mg) and B (1.88 g) using CH_2_Cl_2_:MeOH:NH_4_OH (60:30:0.2, 2×) and EtOAc:MeOH:NH_3_ (100:8:3, 2×) gave **1** (2.1 g).

#### 4.1.4 Procedure for Preparation of **1** Derivatives

Harmine **1** (1.0 eq) was dissolved in 2 mL of a 1:1 (v/v) mixture of anhydrous dimethylformamide (DMF) and tetrahydrofuran (THF) under an inert argon atmosphere. Sodium hydride (NaH, 1.05 eq; or 1.5 eq in selected cases, 60% suspension in mineral oil) was carefully added in portions to the solution, maintaining the reaction mixture in an ice bath at 0°C. The mixture was stirred at this temperature for 10 min. Subsequently, the alkylating agent (1.05 eq) was added, and the reaction was stirred at ambient temperature overnight. The progress of the reaction was monitored using thin‐layer chromatography (TLC). Upon completion, the reaction mixture was concentrated under reduced pressure, and the resulting residue was purified by preparative TLC to obtain **2**–**34**.

9‐Benzyl‐7‐methoxy‐1‐methyl‐9*H*‐pyrido[3,4‐*b*]indole (**2**): Harmine **1** (40 mg; 0.188 mmol), NaH (8 mg; 0.197 mmol), benzyl bromide (23 μL; 0.197 mmol), DMF/THF (v/v = 1:1, 2 mL). The residue was purified by preparative TLC using mobile phase EtOAc/MeOH/28% aq. sol. NH_4_OH (100:4:1.5) to obtain pure product **2** as a white solid. Yield: 95%. ^1^H NMR (600 MHz, CDCl_3_) *δ* 8.30 (d, *J* = 5.3 Hz, 1H, Ar*H*), 8.00 (d, *J* = 8.6 Hz, 1H, Ar*H*), 7.77 (d, *J* = 5.3 Hz, 1H, Ar*H*), 7.29–7.21 (m, 3H, 3 × Ar*H*), 7.01–6.98 (m, 2H, 2 × Ar*H*), 6.90 (dd, *J* = 8.6, *J* = 2.2 Hz, 1H, Ar*H*), 6.75 (d, *J* = 2.2 Hz, 1H, Ar*H*), 5.71 (s, 2H, Ar‐C*H*_*2*_N), 3.84 (s, 3H, Ar‐OC*H*_3_), 2.83 (s, 3H, Ar‐C*H*_3_). ^13^C NMR (151 MHz, CDCl_3_) *δ* 161.1 (Ar*C*‐OCH_3_), 143.5 (Ar*C*‐N), 141.0 (Ar*C*‐N), 138.6 (Ar*C*H‐N), 137.9 (Ar*C*), 135.7 (Ar*C‐*N), 129.4 (Ar*C*), 129.0 (2 × Ar*C*H), 127.5 (Ar*C*H), 125.4 (2 × Ar*C*H), 122.4 (Ar*C*H), 115.2 (Ar*C*), 112.3 (Ar*C*H), 109.2 (Ar*C*H), 93.3 (Ar*C*H), 55.6 (Ar‐O*C*H_3_), 48.2 (Ar‐*C*H_2_N), 23.0 (Ar‐*C*H_3_). ESI‐HRMS *m/z* calcd for C_20_H_19_N_2_O^+^ [M + H]^+^ 303.1492, found 303.1499; 98.5% purity.

7‐Methoxy‐1‐methyl‐9‐[(3‐methylphenyl)methyl]‐9*H*‐pyrido[3,4‐*b*]indole (**3**): Harmine **1** (40 mg; 0.188 mmol), NaH (8 mg; 0.197 mmol), 3‐methylbenzyl bromide (37 mg; 0.197 mmol), DMF/THF (v/v = 1:1, 2 mL). The residue was purified by preparative TLC using mobile phase cHx/EtOAc/28% aq. sol. NH_4_OH (60:30:0.2), developed twice, to obtain pure product **3** as a white solid. Yield: 90%. ^1^H NMR (600 MHz, CDCl_3_) *δ* 8.30 (d, *J* = 5.2 Hz, 1H, Ar*H*), 8.00 (d, *J* = 8.6 Hz, 1H, Ar*H*), 7.77 (d, *J* = 5.2 Hz, 1H, Ar*H*), 7.15 (t, *J* = 7.6 Hz, 1H, Ar*H*), 7.05 (d, *J* = 7.6 Hz, 1H, Ar*H*), 6.89 (dd, *J* = 8.6 Hz, *J* = 2.1 Hz, 1H, Ar*H*), 6.85 (s, 1H, Ar*H*), 6.78 (d, *J* = 7.6 Hz, 1H, Ar*H*), 6.75 (d, *J* = 2.1 Hz, 1H, Ar*H*), 5.67 (s, 2H, Ar‐C*H*_*2*_N), 3.84 (s, 3H, Ar‐OC*H*_3_), 2.84 (s, 3H, Ar‐C*H*_3_), 2.25 (s, 3H, Ar‐C*H*_3_). ^13^C NMR (151 MHz, CDCl_3_) *δ* 161.0 (Ar*C*‐OCH_3_), 143.6 (Ar*C*‐N), 141.1 (Ar*C*‐N), 138.8 (Ar*C*), 138.7 (Ar*C*H‐N), 137.9 (Ar*C*), 135.8 (Ar*C*‐N), 129.4 (Ar*C*), 128.9 (Ar*C*H), 128.3 (Ar*C*H), 126.0 (Ar*C*H), 122.5 (Ar*C*H), 122.4 (Ar*C*H), 115.2 (Ar*C*), 112.3 (Ar*C*H), 109.2 (Ar*C*H), 93.4 (Ar*C*H), 55.6 6 (Ar‐O*C*H_3_), 48.2 (Ar‐*C*H_2_N), 23.1 (Ar‐*C*H_3_), 21.4 (Ar‐*C*H_3_). ESI‐HRMS *m/z* calcd for C_21_H_21_N_2_O^+^ [M + H]^+^ 317.1648, found 317.1654; 99.4% purity.

7‐Methoxy‐1‐methyl‐9‐[(4‐methylphenyl)methyl]‐9*H*‐pyrido[3,4‐b]indole (**4**): Harmine **1** (40 mg; 0.188 mmol), NaH (8 mg; 0.197 mmol), 4‐methylbenzyl bromide (49 mg; 0.197 mmol), DMF/THF (v/v = 1:1, 2 mL). The residue was purified by preparative TLC using mobile phase cHx/EtOAc/28% aq. sol. NH_4_OH (60:30:0.2), developed twice, to obtain pure product **4** as a white solid. Yield: 93%. ^1^H NMR (600 MHz, CDCl_3_) *δ* 8.30 (d, *J* = 5.2 Hz, 1H, Ar*H*), 8.00 (d, *J* = 8.6 Hz, 1H, Ar*H*), 7.76 (d, *J* = 5.2 Hz, 1H, Ar*H*), 7.08–7.06 (m, 2H, 2 × Ar*H*), 6.90–6.88 (m, 3H, 3 × Ar*H*), 6.75 (d, *J* = 2.2 Hz, 1H, Ar*H*), 5.67 (s, 2H, Ar‐ C*H*_*2*_N), 3.84 (s, 3H, Ar*‐*OC*H*_3_), 2.84 (s, 3H, Ar*‐*C*H*_3_), 2.29 (s, 3H, Ar*‐*C*H*_3_). ^13^C NMR (151 MHz, CDCl_3_) *δ* 161.0 (Ar*C*‐OCH_3_), 143.5 (Ar*C*‐N), 141.0 (Ar*C*‐N), 138.7 (Ar*C*H‐N), 137.2 (Ar*C*), 135.8 (Ar*C*‐N), 134.9 (Ar*C*), 129.7 (3 × Ar*CH*), 129.4 (2 × Ar*C*H), 125.4 (Ar*C*H), 122.3 (Ar*C*H), 115.2 (Ar*C*), 112.3 (Ar*C*H), 109.2 (Ar*C*H), 93.4 (Ar*C*H), 55.6 (Ar‐O*C*H_3_), 48.0 (Ar‐*C*H_2_N), 23.1 (Ar‐*C*H_3_), 21.0 (Ar‐*C*H_3_). ESI‐HRMS *m/z* calcd for C_21_H_21_N_2_O^+^ [M + H]^+^ 317.1648, found 317.1652; 99.3% purity.

9‐[(2,4‐Dimethylphenyl)methyl]‐7‐methoxy‐1‐methyl‐9*H*‐pyrido[3,4‐*b*]indole (**5**): Harmine **1** (40 mg; 0.188 mmol), NaH (8 mg; 0.197 mmol), 2,4‐dimethylbenzyl bromide (50 μL; 0.197 mmol), DMF/THF (v/v = 1:1, 2 mL). The residue was purified by preparative TLC using mobile phase cHx/EtOAc/28% aq. sol. NH_4_OH (60:30:0.2), developed twice, to obtain pure product **5** as a white solid. Yield: 84%. ^1^H NMR (600 MHz, CDCl_3_) *δ* 8.30 (d, *J* = 5.2 Hz, 1H, Ar*H*), 8.01 (d, *J* = 8.6 Hz, 1H, Ar*H*), 7.77 (d, *J* = 5.2 Hz, 1H, Ar*H*), 7.07 (bs, 1H, Ar*H*), 6.90 (dd, *J* = 8.6 Hz, *J* = 2.2 Hz, 1H, Ar*H*), 6.74 (d, *J* = 7.9 Hz, 1H, Ar*H*), 6.66 (d, *J* = 2.2 Hz, 1H, Ar*H*), 6.22 (d, *J* = 7.9 Hz, 1H, Ar*H*), 5.51 (s, 2H, Ar‐C*H*_*2*_N), 3.83 (s, 3H, Ar*‐*OC*H*_3_), 2.72 (s, 3H, Ar*‐*C*H*_3_), 2.44 (s, 3H, Ar*‐*C*H*_3_), 2.25 (s, 3H, Ar*‐*C*H*_3_). ^13^C NMR (151 MHz, CDCl_3_) *δ* 161.1 (Ar*C*‐OCH_3_), 143.6 (Ar*C*‐N), 141.1 (Ar*C*‐N), 138.5 (Ar*C*H‐N), 136.9 (Ar*C*), 135.7 (Ar*C*‐N), 133.5 (Ar*C*), 132.6 (Ar*C*), 131.2 (Ar*C*H), 129.3 (Ar*C*), 127.2 (Ar*C*H), 124.8 (Ar*C*H), 122.4 (Ar*C*H), 115.1 (Ar*C*), 112.2 (Ar*C*H), 109.2 (Ar*C*H), 93.1 (Ar*C*H), 55.6 (Ar‐O*C*H_3_), 46.1 (Ar‐*C*H_2_N), 22.4 (Ar‐*C*H_3_), 20.9 (Ar‐*C*H_3_), 19.0 (Ar‐*C*H_3_). ESI‐HRMS *m/z* calcd for C_22_H_23_N_2_O^+^ [M + H]^+^ 331.1805, found 333.1810; 98.9% purity.

9‐[(3,5‐Dimethylphenyl)methyl]‐7‐methoxy‐1‐methyl‐3*H*,4*H*,9*H*‐pyrido[3,4‐*b*]indole (**6**): Harmine **1** (40 mg; 0.188 mmol), NaH (8 mg; 0.197 mmol), 3,5‐dimethylbenzyl bromide (62 mg; 0.197 mmol), DMF/THF (v/v = 1:1, 2 mL). The residue was purified by preparative TLC using mobile phase cHx/EtOAc/28% aq. sol. NH_4_OH (60:30:0.2), developed twice, to obtain pure product **6** as a white solid. Yield: 85%. ^1^H NMR (600 MHz, CDCl_3_) *δ* 8.30 (d, *J* = 5.2 Hz, 1H, Ar*H*), 8.01 (d, *J* = 8.6 Hz, 1H, Ar*H*), 7.77 (d, *J* = 5.2 Hz, 1H, Ar*H*), 6.90 (dd, *J* = 8.6 Hz, *J* = 2.2 Hz, 1H, Ar*H*), 6.87 (s, 1H, Ar*H*), 6.75 (d, *J* = 2.2 Hz, 1H, Ar*H*), 6.63 (s, 2H, 2 × Ar*H*), 5.63 (s, 2H Ar‐C*H*_*2*_N), 3.85 (s, 3H, Ar*‐*OC*H*_3_), 2.84 (s, 3H, Ar*‐*C*H*_3_), 2.20 (s, 6H, 2 × Ar*‐*C*H*_3_). ^13^C NMR (151 MHz, CDCl_3_) *δ* 161.0 (Ar*C*‐OCH_3_), 143.6 (Ar*C*‐N), 141.1 (Ar*C*‐N), 138.7 (2 × Ar*C*), 138.6 (Ar*C*H‐N), 137.9 (Ar*C*), 135.9 (Ar*C*‐N), 129.3 (Ar*C*), 129.2 (Ar*C*H), 123.2 (2 × Ar*C*H), 122.3 (Ar*C*H), 115.2 (Ar*C*), 112.3 (Ar*C*H), 109.1 (Ar*C*H), 93.4 (Ar*C*H), 55.6 (Ar‐O*C*H_3_), 48.3 (Ar‐*C*H_2_N), 23.1 (Ar‐*C*H_3_), 21.3 (2 × Ar‐*C*H_3_). ESI‐HRMS *m/z* calcd for C_22_H_23_N_2_O^+^ [M + H]^+^ 331.1805, found 331.1808; 99.1% purity.

7‐Methoxy‐1‐methyl‐9‐{[4‐(propan‐2‐yl)phenyl]methyl}‐9*H*‐pyrido[3,4‐*b*]indole (**7**): Harmine **1** (40 mg; 0.188 mmol), NaH (8 mg; 0.197 mmol), 4‐isopropylbenzyl bromide (33 μL; 0.197 mmol), DMF/THF (v/v = 1:1, 2 mL). The residue was purified by preparative TLC using mobile phase EtOAc/MeOH/28% aq. sol. NH_4_OH (100:4:1.5) to obtain pure product **7** as a white solid. Yield: 88%. ^1^H NMR (600 MHz, CDCl_3_) *δ* 8.29 (d, *J* = 5.2 Hz, 1H, Ar*H*), 8.00 (d, *J* = 8.6 Hz, 1H, Ar*H*), 7.76 (d, *J* = 5.2 Hz, 1H, Ar*H*), 7.13–7.10 (m, 2H, 2 × Ar*H*), 6.94–6.91 (m, 2H, 2 × Ar*H*), 6.89 (dd, *J* = 8.6 Hz, *J* = 2.1 Hz, 1H, Ar*H*), 6.78 (d, *J* = 2.1 Hz, 1H, Ar*H*), 5.68 (s, 2H, Ar‐C*H*_*2*_N), 3.85 (s, 3H, Ar*‐*OC*H*_3_), 2.88–2.81 (m, 1H, overlap, Ar‐C*H*(CH_3_)_2_) 2.85 (s, 3H, overlap, Ar‐C*H*_3_), 1.19 (d, *J* = 6.9 Hz, 6H, CH(C*H*_3_)_2_). ^13^C NMR (151 MHz, CDCl_3_) *δ* 161.0 (Ar*C*‐OCH_3_), 148.2 (Ar*C*‐iPro), 143.6 (Ar*C*‐N), 141.1 (Ar*C*‐N), 138.6 (Ar*C*H‐N), 135.8 (Ar*C*‐N), 135.2 (Ar*C*), 129.4 (Ar*C*), 127.0 (2 × Ar*C*H), 125.5 (2 × Ar*C*H), 122.4 (Ar*C*H), 115.2 (Ar*C*), 112.3 (Ar*C*H), 109.2 (Ar*C*H), 93.4 (Ar*C*H), 55.6 (Ar‐O*C*H_3_), 48.1 (Ar‐*C*H_2_N), 33.7 (Ar‐*C*H(CH_3_)_2_), 23.9 (2 × Ar‐*C*H_3_), 23.1 (Ar‐*C*H_3_). ESI‐HRMS *m/z* calcd for C_23_H_25_N_2_O^+^ [M + H]^+^ 345.1961, found 345.1968; 98.9% purity.

9‐[(4‐*tert*‐Butylphenyl)methyl]‐7‐methoxy‐1‐methyl‐9*H*‐pyrido[3,4‐*b*]indole (**8**): Harmine **1** (40 mg; 0.188 mmol), NaH (8 mg; 0.197 mmol), 4‐*tert*‐butylbenzyl bromide (36 μL; 0.197 mmol), DMF/THF (v/v = 1:1, 2 mL). The residue was purified by preparative TLC using mobile phase EtOAc/MeOH/28% aq. sol. NH_4_OH (100:4:1.5) to obtain pure product **8** as a white solid. Yield: 87%. ^1^H NMR (600 MHz, CDCl_3_) *δ* 8.29 (d, *J* = 5.2 Hz, 1H, Ar*H*), 8.00 (d, *J* = 8.6 Hz, 1H, Ar*H*), 7.76 (d, *J* = 5.2 Hz, 1H, Ar*H*), 7.29–7.26 (m, 2H, 2 × Ar*H*), 6.94–6.91 (m, 2H, 2 × Ar*H*), 6.89 (dd, *J* = 8.6 Hz, *J* = 2.2 Hz, 1H, Ar*H*), 6.78 (d, *J* = 2.2 Hz, 1H, Ar*H*), 5.69 (s, 2H, Ar‐C*H*_*2*_N), 3.85 (s, 3H, Ar*‐*OC*H*_3_), 2.85 (s, 3H, Ar‐C*H*_3_), 1.26 (s, 9H, 3 × C*H*_3_). ^13^C NMR (151 MHz, CDCl_3_) *δ* 161.0 (Ar*C*‐OCH_3_), 150.5 (Ar*C*‐iBu), 143.6 (Ar*C*‐N), 141.1 (Ar*C*‐N), 138.6 (Ar*C*H‐N), 135.8 (Ar*C*‐N), 134.9 (Ar*C*), 129.4 (Ar*C*), 125.9 (2 × Ar*C*H), 125.2 (2 × Ar*C*H), 122.4 (Ar*C*H), 115.2 (Ar*C*), 112.3 (Ar*C*H), 109.2 (Ar*C*H), 93.4 (Ar*C*H), 55.6 (Ar‐O*C*H_3_), 48.0 (Ar‐*C*H_2_N), 34.5 (Ar‐*C*(CH_3_)_3_), 31.3 (Ar‐C(*C*H_3_)_3_), 23.1 (Ar‐*C*H_3_). ESI‐HRMS *m/z* calcd for C_24_H_27_N_2_O^+^ [M + H]^+^ 359.2118, found 359.2124; 96.5% purity.

7‐Methoxy‐9‐[(3‐methoxyphenyl)methyl]‐1‐methyl‐9*H*‐pyrido[3,4‐*b*]indole (**9**): Harmine **1** (40 mg; 0.188 mmol), NaH (8 mg; 0.197 mmol), 3‐methoxybenzyl bromide (28 μL; 0.197 mmol), DMF/THF (v/v = 1:1, 2 mL). The residue was purified by preparative TLC using mobile phase EtOAc/MeOH/28% aq. sol. NH_4_OH (100:4:1.5) to obtain pure product **9** as a white solid. Yield: 91%. ^1^H NMR (600 MHz, CDCl_3_) *δ* 8.29 (d, *J* = 5.3 Hz, 1H, Ar*H*), 7.99 (d, *J* = 8.7 Hz, 1H, Ar*H*), 7.75 (d, *J* = 5.3 Hz, 1H, Ar*H*), 7.19 (t, *J* = 7.8 Hz, 1H, Ar*H*), 6.89 (dd, *J* = 8.7 Hz, *J* = 2.1 Hz, 1H, Ar*H*), 6.79–6.73 (m, 2H, 2 × Ar*H*), 6.58 (d, *J* = 7.8 Hz, 1H, Ar*H*), 6.54 (s, 1H, Ar*H*), 5.67 (s, 2H, Ar‐C*H*_*2*_N), 3.84 (s, 3H, Ar*‐*OC*H*_3_), 3.68 (s, 3H, Ar*‐*OC*H*_3_), 2.83 (s, 3H, Ar*‐*C*H*_3_). ^13^C NMR (151 MHz, CDCl_3_) *δ* 161.1 (Ar*C*‐OCH_3_), 160.2 (Ar*C*‐OCH_3_), 143.5 (Ar*C*‐N), 141.0 (Ar*C*‐N), 139.6 (Ar*C*‐N), 138.7 (Ar*C*H), 135.8 (Ar*C*‐N), 130.1 (Ar*C*H), 129.4 (Ar*C*), 122.4 (Ar*C*H), 117.7 (Ar*C*H), 115.2 (Ar*C*), 112.3 (Ar*C*H), 112.3 (Ar*C*H), 111.6 (Ar*C*H), 109.2 (Ar*C*H), 93.3 (Ar*C*H), 55.6 (Ar‐O*C*H_3_), 55.1 (Ar‐O*C*H_3_), 48.2 (Ar‐*C*H_2_N), 23.0 (Ar‐*C*H_3_). ESI‐HRMS *m/z* calcd for C_21_H_21_N_2_O^+^ [M + H]^+^ 333.1598, found 333.1611; 99.4% purity.

9‐[(3,5‐Dimethoxyphenyl)methyl]‐7‐methoxy‐1‐methyl‐9*H*‐pyrido[3,4‐*b*]indole (**10**): Harmine **1** (40 mg; 0.188 mmol), NaH (8 mg; 0.197 mmol), 3,5‐dimethoxybenzyl bromide (46 mg; 0.197 mmol), DMF/THF (v/v = 1:1, 2 mL). The residue was purified by preparative TLC using mobile phase EtOAc/MeOH/28% aq. sol. NH_4_OH (100:4:1.5) to obtain pure product **10** as a white solid. Yield: 82%. ^1^H NMR (600 MHz, CDCl_3_) *δ* 8.29 (d, *J* = 5.3 Hz, 1H, Ar*H*), 7.99 (d, *J* = 8.6 Hz, 1H, Ar*H*), 7.75 (d, *J* = 5.3 Hz, 1H, Ar*H*), 6.89 (dd, *J* = 8.6 Hz, *J* = 2.2 Hz, 1H, Ar*H*), 6.75 (d, *J* = 2.2 Hz, 1H, Ar*H*), 6.32 (t, *J* = 2.2 Hz, 1H, Ar*H*), 6.15–6.14 (m, 2H, 2 × Ar*H*), 5.63 (s, 2H, Ar‐C*H*_*2*_N), 3.85 (s, 3H, Ar*‐*OC*H*_3_), 3.67 (s, 6H, 2 × Ar*‐*OC*H*_3_), 2.84 (s, 3H, Ar*‐*C*H*_3_). ^13^C NMR (151 MHz, CDCl_3_) *δ* 161.4 (2 × Ar*C*‐OCH_3_), 161.1 (Ar*C*‐OCH_3_), 143.6 (Ar*C*‐N), 141.1 (Ar*C*‐N), 140.5 (Ar*C*‐N), 138.8 (Ar*C*H), 135.8 (Ar*C*‐N), 129.4 (Ar*C*), 122.4 (Ar*C*H), 115.2 (Ar*C*), 112.3 (Ar*C*H), 109.2 (Ar*C*H), 103.7 (2 × Ar*C*H), 98.7 (Ar*C*H), 93.4 (Ar*C*H), 55.6 (Ar‐O*C*H_3_), 55.3 (2 × Ar‐O*C*H_3_), 48.3 (Ar‐*C*H_2_N), 23.0 (Ar‐*C*H_3_). ESI‐HRMS *m/z* calcd for C_22_H_23_N_2_O_3_^+^ [M + H]^+^ 363.1703, found 363.1707; 96.8% purity.

9‐[(2‐Fluorophenyl)methyl]‐7‐methoxy‐1‐methyl‐9*H*‐pyrido[3,4‐*b*]indole (**11**): Harmine **1** (40 mg; 0.188 mmol), NaH (8 mg; 0.197 mmol), 2‐fluorobenzyl bromide (21 μL; 0.197 mmol), DMF/THF (v/v = 1:1, 2 mL). The residue was purified by preparative TLC using mobile phase EtOAc/MeOH/28% aq. sol. NH_4_OH (100:4:1.5) to obtain pure product **11** as a white solid. Yield: 85%. ^1^H NMR (600 MHz, CDCl_3_) *δ* 8.31 (d, *J* = 5.2 Hz, 1H, Ar*H*), 8.01 (d, *J* = 8.6 Hz, 1H, Ar*H*), 7.77 (d, *J* = 5.2 Hz, 1H, Ar*H*), 7.24–7.21 (m, 1H, Ar*H*), 7.16–7.11 (m, 1H, Ar*H*), 6.93–6.88 (m, 2H, 2 × Ar*H*), 6.76 (d, *J* = 2.1 Hz, 1H, Ar*H*), 6.51–6.47 (m, 1H, Ar*H*), 5.77 (s, 2H, Ar‐C*H*_*2*_N), 3.86 (s, 3H, Ar*‐*OC*H*_3_), 2.82 (s, 3H, Ar*‐*C*H*_3_). ^13^C NMR (151 MHz, CDCl_3_) *δ* 161.2 (Ar*C*‐OCH_3_), 159.5 (d, *J* = 245.3 Hz, Ar*C*‐F), 143.4 (Ar*C*‐N), 141.0 (Ar*C*‐N), 138.9 (Ar*C*H‐N), 135.7 (Ar*C*‐N), 129.6 (Ar*C*), 129.2 (d, *J* = 8.0 Hz, Ar*C*H), 127.1 (d, *J* = 4.0 Hz, Ar*C*H), 125.0 (d, *J* = 13.8 Hz, Ar*C*), 124.8 (d, *J* = 3.5 Hz, Ar*C*H), 122.5 (Ar*C*H), 115.4 (d, *J* = 20.8 Hz, Ar*C*H), 115.3 (Ar*C*), 112.4 (Ar*C*H), 109.4 (Ar*C*H), 93.1 (Ar*C*H), 55.7 (Ar‐O*C*H_3_), 42.3 (d, *J* = 5.7 Hz, Ar‐*C*H_2_N), 22.8 (Ar‐*C*H_3_). ESI‐HRMS *m/z* calcd for C_20_H_18_FN_2_O^+^ [M + H]^+^ 321.1398, found 321.1406; 95.0% purity.

9‐[(3‐Fluorophenyl)methyl]‐7‐methoxy‐1‐methyl‐9*H*‐pyrido[3,4‐*b*]indole (**12**): Harmine **1** (40 mg; 0.188 mmol), NaH (8 mg; 0.197 mmol), 3‐fluorobenzyl bromide (24 μL; 0.197 mmol), DMF/THF (v/v = 1:1, 2 mL). The residue was purified by preparative TLC using mobile phase EtOAc/MeOH/28% aq. sol. NH_4_OH (100:4:1.5) to obtain pure product **12** as a white solid. Yield: 88%. ^1^H NMR (600 MHz, CDCl_3_) *δ* 8.31 (d, *J* = 5.3 Hz, 1H, Ar*H*), 8.01 (d, *J* = 8.6 Hz, 1H, Ar*H*), 7.77 (d, *J* = 5.3 Hz, 1H, Ar*H*), 7.28–7.21 (m, 1H, Ar*H*), 6.95–6.93 (m, 1H, Ar*H*), 6.91 (dd, *J* = 8.6 Hz, *J* = 2.2 Hz, 1H, Ar*H*), 6.81–6.78 (m, 1H, Ar*H*), 6.72 (d, *J* = 2.2 Hz, 1H, Ar*H*), 6.70–6.67 (m, 1H, Ar*H*), 5.70 (s, 2H, Ar‐C*H*_*2*_N), 3.85 (s, 3H, Ar*‐*OC*H*_3_), 2.82 (s, 3H, Ar*‐*C*H*_3_). ^13^C NMR (151 MHz, CDCl_3_) *δ* 163.3 (d, *J* = 247.2 Hz, Ar*C*‐F), 161.2 (Ar*C*‐OCH_3_), 143.4 (Ar*C*‐N), 140.8 (Ar*C*‐N), 140.6 (d, *J* = 6.9 Hz, Ar*C*), 138.8 (Ar*C*H‐N, ArC), 135.6 (Ar*C*‐N), 130.7 (d, *J* = 8.4 Hz), 129.6 (Ar*C*H), 122.5 (Ar*C*H), 121.0 (d, *J* = 2.9 Hz, Ar*C*H), 115.2 (Ar*C*), 114.6 (d, *J* = 21.1 Hz, Ar*C*H), 112.6 (d, *J* = 22.4 Hz, Ar*C*H), 112.4 (Ar*C*H), 109.3 (Ar*C*H), 93.3 (Ar*C*H), 55.6 (Ar‐O*C*H_3_), 47.8 (Ar‐*C*H_2_N), 22.9 (Ar‐*C*H_3_). ESI‐HRMS *m/z* calcd for C_20_H_18_FN_2_O^+^ [M + H]^+^ 321.1398, found 321.1411; 97.5% purity.

9‐[(4‐Fluorophenyl)methyl]‐7‐methoxy‐1‐methyl‐9*H*‐pyrido[3,4‐*b*]indole (**13**): Harmine **1** (40 mg; 0.188 mmol), NaH (8 mg; 0.197 mmol), 4‐fluorobenzyl bromide (25 μL; 0.197 mmol), DMF/THF (v/v = 1:1, 2 mL). The residue was purified by preparative TLC using mobile phase EtOAc/MeOH/28% aq. sol. NH_4_OH (100:4:1.5) to obtain pure product **13** as a white solid. Yield: 86%. ^1^H NMR (600 MHz, CDCl_3_) *δ* 8.30 (d, *J* = 5.2 Hz, 1H, Ar*H*), 8.00 (d, *J* = 8.6 Hz, 1H, Ar*H*), 7.76 (d, *J* = 5.2 Hz, 1H, Ar*H*), 6.98–6.94 (m, 4H, 4 × Ar*H*), 6.90 (dd, *J* = 8.6 Hz, *J* = 2.2 Hz, 1H, Ar*H*), 6.72 (d, *J* = 2.2 Hz, 1H, Ar*H*), 5.67 (s, 2H, Ar‐C*H*_*2*_N), 3.84 (s, 3H, Ar*‐*OC*H*_3_), 2.82 (s, 3H, Ar*‐*C*H*_3_). ^13^C NMR (151 MHz, CDCl_3_) *δ* 162.1 (d, *J* = 246.0 Hz, Ar*C*‐F), 161.1 (Ar*C*‐OCH_3_), 143.4 (Ar*C*‐N), 140.9 (Ar*C*‐N), 138.8 (Ar*C*H‐N), 135.6 (Ar*C*‐N), 133.5 (Ar*C*), 129.5 (Ar*C*), 127.1 (d, *J* = 8.0 Hz, 2 × Ar*C*H), 122.5 (Ar*C*H), 115.9 (d, *J* = 21.7 Hz, 2 × Ar*C*H), 115.2 (Ar*C*), 112.3 (Ar*C*H), 109.2 (Ar*C*H), 93.4 (Ar*C*H), 55.6 (Ar‐O*C*H_3_), 47.6 (Ar‐*C*H_2_N), 23.0 (Ar‐*C*H_3_). ESI‐HRMS *m/z* calcd for C_20_H_18_FN_2_O^+^ [M + H]^+^ 321.1398, found 321.1402; 99.4% purity.

9‐[(2‐Chlorophenyl)methyl]‐7‐methoxy‐1‐methyl‐9*H*‐pyrido[3,4‐*b*]indole (**14**): Harmine **1** (40 mg; 0.188 mmol), NaH (8 mg; 0.197 mmol), 2‐chlorobenzyl bromide (41 mg; 0.197 mmol), DMF/THF (v/v = 1:1, 2 mL). The residue was purified by preparative TLC using mobile phase cHx/EtOAc/28% aq. sol. NH_4_OH (60:30:0.2), developed twice, to obtain pure product **14** as a white solid. Yield: 89%. ^1^H NMR (600 MHz, CDCl_3_) *δ* 8.31 (d, *J* = 5.2 Hz, 1H, Ar*H*), 8.01 (d, *J* = 8.6 Hz, 1H, Ar*H*), 7.77 (d, *J* = 5.2 Hz, 1H, Ar*H*), 7.46 (dd, *J* = 7.8 Hz, *J* = 1.3 Hz, 1H, Ar*H*), 7.20 (td, *J* = 7.8 Hz, *J* = 1.3 Hz, 1H, Ar*H*), 7.00 (td, *J* = 7.8 Hz, *J* = 1.3 Hz, 1H, Ar*H*), 6.91 (dd, *J* = 8.6 Hz, *J* = 2.1 Hz, 1H, Ar*H*), 6.70 (d, *J* = 2.1 Hz, 1H, Ar*H*), 6.40 (dd, *J* = 7.8 Hz, *J* = 1.3 Hz, 1H, Ar*H*), 5.72 (s, 2H, Ar‐C*H*_*2*_N), 3.85 (s, 3H, Ar*‐*OC*H*_3_), 2.75 (s, 3H, Ar*‐*C*H*_3_). ^13^C NMR (151 MHz, CDCl_3_) *δ* 161.2 (Ar*C*‐OCH_3_), 143.3 (Ar*C*‐N), 141.1 (Ar*C*‐N), 138.9 (Ar*C*H‐N), 135.6 (Ar*C*‐N), 135.2 (Ar*C*), 131.5 (Ar*C*), 129.6 (Ar*C*H), 129.5 (Ar*C*), 128.8 (Ar*C*H), 127.5 (Ar*C*H), 126.8 (Ar*C*H), 122.5 (Ar*C*H), 115.2 (Ar*C*), 112.3 (Ar*C*H), 109.5 (Ar*C*H), 93.1 (Ar*C*H), 55.7 (Ar‐O*C*H_3_), 46.3 (Ar‐*C*H_2_N), 22.6 (Ar‐*C*H_3_),. ESI‐HRMS *m/z* calcd for C_20_H_18_ClN_2_O^+^ [M + H]^+^ 337.1102, found 337.1108; 99.3% purity.

9‐[(3‐Chlorophenyl)methyl]‐7‐methoxy‐1‐methyl‐9*H*‐pyrido[3,4‐*b*]indole (**15**): Harmine **1** (40 mg; 0.188 mmol), NaH (8 mg; 0.197 mmol), 3‐chlorobenzyl bromide (41 mg; 0.197 mmol), DMF/THF (v/v = 1:1, 2 mL). The residue was purified by preparative TLC using mobile phase cHx/EtOAc/28% aq. sol. NH_4_OH (60:30:0.2), developed twice, to obtain pure product **15** as a white solid. Yield: 90%. ^1^H NMR (600 MHz, CDCl_3_) *δ* 8.31 (d, *J* = 5.2 Hz, 1H, Ar*H*), 8.01 (d, *J* = 8.6 Hz, 1H, Ar*H*), 7.77 (d, *J* = 5.2 Hz, 1H, Ar*H*), 7.24–7.21 (m, 1H, Ar*H*), 7.19 (t, *J* = 7.6 Hz, 1H, Ar*H*), 7.04 (bs, 1H, Ar*H*), 6.91 (dd, *J* = 8.6 Hz, *J* = 2.2 Hz, 1H, Ar*H*), 6.83 (d, *J* = 7.6 Hz, 1H, Ar*H*), 6.71 (d, *J* = 2.2 Hz, 1H, Ar*H*), 5.68 (s, 2H, Ar‐C*H*_*2*_N), 3.85 (s, 3H, Ar*‐*OC*H*_3_), 2.82 (s, 3H, Ar*‐*C*H*_3_). ^13^C NMR (151 MHz, CDCl_3_) *δ* 161.2 (Ar*C*‐OCH_3_), 143.3 (Ar*C*‐N), 140.8 (Ar*C*‐N), 140.0 (Ar*C*‐N), 139.0 (Ar*C*H‐N), 135.6 (Ar*C*), 135.1 (Ar*C*), 130.4 (Ar*C*H), 129.5 (Ar*C*), 127.9 (Ar*C*H), 125.6 (Ar*C*H), 123.6 (Ar*C*H), 122.5 (Ar*C*H), 115.3 (Ar*C*), 112.4 (Ar*C*H), 109.3 (Ar*C*H), 93.3 (Ar*C*H), 55.7 (Ar‐O*C*H_3_), 47.8, 23.0 (Ar‐*C*H_3_). ESI‐HRMS *m/z* calcd for C_20_H_18_ClN_2_O^+^ [M + H]^+^ 337.1102, found 337.1107; 99.2% purity.

9‐[(4‐Chlorophenyl)methyl]‐7‐methoxy‐1‐methyl‐9*H*‐pyrido[3,4‐*b*]indole (**16**): Harmine **1** (40 mg; 0.188 mmol), NaH (8 mg; 0.197 mmol), 4‐chlorobenzyl bromide (41 mg; 0.197 mmol), DMF/THF (v/v = 1:1, 2 mL). The residue was purified by preparative TLC using mobile phase cHx/EtOAc/28% aq. sol. NH_4_OH (60:30:0.2), developed twice, to obtain pure product **16** as a white solid. Yield: 94%. ^1^H NMR (600 MHz, CDCl_3_) *δ* 8.31 (d, *J* = 5.2 Hz, 1H, Ar*H*), 8.01 (d, *J* = 8.6 Hz, 1H, Ar*H*), 7.77 (d, *J* = 5.2 Hz, 1H, Ar*H*), 7.26–7.23 (m, 2H, 2 × Ar*H*), 6.94–6.92 (m, 2H, 2 × Ar*H*), 6.90 (dd, *J* = 8.6 Hz, *J* = 2.1 Hz, 1H, Ar*H*), 6.71 (d, *J* = 2.1 Hz, 1H, Ar*H*), 5.67 (s, 2H, Ar‐C*H*_*2*_N), 3.84 (s, 3H, Ar*‐*OC*H*_3_), 2.82 (s, 3H, Ar*‐*C*H*_3_). ^13^C NMR (151 MHz, CDCl_3_) *δ* 161.2 (Ar*C*‐OCH_3_), 143.4 (Ar*C*‐N), 140.8 (Ar*C*‐N), 138.9 (Ar*C*H‐N), 136.3 (Ar*C*), 135.6 (Ar*C*‐N), 133.4 (Ar*C*), 129.5 (Ar*C*), 129.2 (2 × Ar*C*H), 126.9 (2 × Ar*C*H), 122.5 (Ar*C*H), 115.3 (Ar*C*), 112.4 (Ar*C*H), 109.3 (Ar*C*H), 93.3 (Ar*C*H), 55.6 (Ar‐O*C*H_3_), 47.7 (Ar‐*C*H_2_N), 23.0 (Ar‐*C*H_3_). ESI‐HRMS *m/z* calcd for C_20_H_18_ClN_2_O^+^ [M + H]^+^ 337.1102, found 337.1109; 99.5% purity.

9‐[(3,4‐Dichlorophenyl)methyl]‐7‐methoxy‐1‐methyl‐9*H*‐pyrido[3,4‐*b*]indole (**17**): Harmine **1** (40 mg; 0.188 mmol), NaH (8 mg; 0.197 mmol), 3,4‐dichlorobenzyl bromide (29 μL; 0.197 mmol), DMF/THF (v/v = 1:1, 2 mL). The residue was purified by preparative TLC using mobile phase EtOAc/MeOH/28% aq. sol. NH_4_OH (100:4:1.5) to obtain pure product **17** as a white solid. Yield: 88%. ^1^H NMR (600 MHz, CDCl_3_) *δ* 8.32 (d, *J* = 5.3 Hz, 1H, Ar*H*), 8.01 (d, *J* = 8.6 Hz, 1H, Ar*H*), 7.77 (d, *J* = 5.3 Hz, 1H, Ar*H*), 7.33 (d, *J* = 8.3 Hz, 1H, Ar*H*), 7.16–7.15 (m, 1H, Ar*H*), 6.92 (dd, *J* = 8.6 Hz, *J* = 2.1 Hz, 1H, Ar*H*), 6.80–6.77 (m, 1H, Ar*H*), 6.68 (d, *J* = 2.1 Hz, 1H, Ar*H*), 5.65 (s, 2H, Ar‐C*H*_*2*_N), 3.86 (s, 3H, Ar*‐*OC*H*_3_), 2.82 (s, 3H, Ar*‐*C*H*_3_). ^13^C NMR (151 MHz, CDCl_3_) *δ* 161.2 (Ar*C*‐OCH_3_), 143.2 (Ar*C*‐N), 140.7 (Ar*C*‐N), 139.1 (Ar*C*H‐N), 138.2 (Ar*C*), 135.5 (Ar*C*‐N), 133.4 (Ar*C*), 131.8 (Ar*C*), 131.1 (Ar*C*H), 129.6 (Ar*C*), 127.5 (Ar*C*H), 124.8 (Ar*C*H), 122.6 (Ar*C*H), 115.3 (Ar*C*), 112.4 (Ar*C*H), 109.3 (Ar*C*H), 93.3 (Ar*C*H), 55.7 (Ar‐O*C*H_3_), 47.3 (Ar‐*C*H_2_N), 23.0 (Ar‐*C*H_3_). ESI‐HRMS *m/z* calcd for C_20_H_17_Cl_2_N_2_O^+^ [M + H]^+^ 371.0712, found 371.0717; 97.0% purity.

9‐[(2‐Bromophenyl)methyl]‐7‐methoxy‐1‐methyl‐9*H*‐pyrido[3,4‐*b*]indole (**18**): Harmine **1** (40 mg; 0.188 mmol), NaH (8 mg; 0.197 mmol), 2‐bromobenzyl bromide (49 mg; 0.197 mmol), DMF/THF (v/v = 1:1, 2 mL). The residue was purified by preparative TLC using mobile phase EtOAc/MeOH/28% aq. sol. NH_4_OH (100:4:1.5) to obtain pure product **18** as a white solid. Yield: 85%. ^1^H NMR (600 MHz, CDCl_3_) *δ* 8.31 (d, *J* = 5.2 Hz, 1H, Ar*H*), 8.01 (d, *J* = 8.6 Hz, 1H, Ar*H*), 7.77 (d, *J* = 5.2 Hz, 1H, Ar*H*), 7.64 (d, *J* = 7.8 Hz, 1H, Ar*H*), 7.12 (t, *J* = 7.8 Hz, 1H, Ar*H*), 7.05 (t, *J* = 7.8 Hz, 1H, Ar*H*), 6.91 (dd, *J* = 8.6 Hz, *J* = 2.1 Hz, 1H, Ar*H*), 6.70 (d, *J* = 2.1 Hz, 1H, Ar*H*), 6.38 (d, *J* = 7.8 Hz, 1H, Ar*H*), 5.67 (s, 2H, Ar‐C*H*_*2*_N), 3.85 (s, 3H, Ar*‐*OC*H*_3_), 2.74 (s, 3H Ar*‐*C*H*_3_). ^13^C NMR (151 MHz, CDCl_3_) *δ* 161.2 (Ar*C*‐OCH_3_), 143.3 (Ar*C*‐N), 141.1 (Ar*C*‐N), 139.0 (Ar*C*H‐N), 136.7 (Ar*C*), 135.5 (Ar*C*‐N), 132.9 (Ar*C*H), 129.5 (Ar*C*), 129.1 (Ar*C*H), 128.1 (Ar*C*H), 127.0 (Ar*C*H), 122.5 (Ar*C*H), 121.3 (Ar*C*), 115.2 (Ar*C*), 112.3 (Ar*C*H), 109.5 (Ar*C*H), 93.1 (Ar*C*H), 55.7 (Ar‐O*C*H_3_), 48.8 (Ar‐*C*H_2_N), 22.5 (Ar‐*C*H_3_). ESI‐HRMS *m/z* calcd for C_20_H_18_BrN_2_O^+^ [M + H]^+^ 381.0597, found 381.0601; 98.6% purity.

9‐[(3‐Bromophenyl)methyl]‐7‐methoxy‐1‐methyl‐9*H*‐pyrido[3,4‐*b*]indole (**19**): Harmine **1** (40 mg; 0.188 mmol), NaH (8 mg; 0.197 mmol), 3‐bromobenzyl bromide (49 mg; 0.197 mmol), DMF/THF (v/v = 1:1, 2 mL). The residue was purified by preparative TLC using mobile phase EtOAc/MeOH/28% aq. sol. NH_4_OH (100:4:1.5) to obtain pure product **19** as a white solid. Yield: 92%. ^1^H NMR (600 MHz, CDCl_3_) *δ* 8.31 (d, *J* = 5.2 Hz, 1H, Ar*H*), 8.01 (d, *J* = 8.6 Hz, 1H, Ar*H*), 7.77 (d, *J* = 5.2 Hz, 1H, Ar*H*), 7.38 (d, *J* = 7.8 Hz, 1H, Ar*H*), 7.23 (bs, 1H, Ar*H*), 7.12 (t, *J* = 7.8 Hz, 1H, Ar*H*), 6.91 (dd, *J* = 8.6 Hz, *J* = 2.2 Hz, 1H, Ar*H*), 6.85 (d, *J* = 7.8 Hz, 1H, Ar*H*), 6.70 (d, *J* = 2.2 Hz, 1H, Ar*H*), 5.67 (s, 2H, Ar‐C*H*_*2*_N), 3.85 (s, 3H, Ar*‐*OC*H*_3_), 2.82 (s, 3H, Ar*‐*C*H*_3_). ^13^C NMR (151 MHz, CDCl_3_) *δ* 161.2 (Ar*C*‐OCH_3_), 143.3 (Ar*C*‐N), 140.8 (Ar*C*‐N), 140.3 (Ar*C*), 139.0 (Ar*C*H‐N), 135.6 (Ar*C*‐N), 130.8 (Ar*C*H), 130.7 (Ar*C*H), 129.5 (Ar*C*), 128.5 (Ar*C*H), 124.1 (Ar*C*H), 123.2 (Ar*C*), 122.5 (Ar*C*H), 115.3 (Ar*C*), 112.4 (Ar*C*H), 109.3 (Ar*C*H), 93.3 (Ar*C*H), 55.7 (Ar‐O*C*H_3_), 47.7 (Ar‐*C*H_2_N), 23.0 (Ar‐*C*H_3_). ESI‐HRMS *m/z* calcd for C_20_H_18_BrN_2_O^+^ [M + H]^+^ 381.0597, found 381.0602; 98.1% purity.

9‐[(4‐Bromophenyl)methyl]‐7‐methoxy‐1‐methyl‐9*H*‐pyrido[3,4‐*b*]indole (**20**): Harmine **1** (40 mg; 0.188 mmol), NaH (8 mg; 0.197 mmol), 4‐bromobenzyl bromide (49 mg; 0.197 mmol), DMF/THF (v/v = 1:1, 2 mL). The residue was purified by preparative TLC using mobile phase EtOAc/MeOH/28% aq. sol. NH_4_OH (100:4:1.5) to obtain pure product **20** as a white solid. Yield: 95%. ^1^H NMR (600 MHz, CDCl_3_) *δ* 8.31 (d, *J* = 5.2 Hz, 1H, Ar*H*), 8.00 (d, *J* = 8.5 Hz, 1H, Ar*H*), 7.76 (d, *J* = 5.2 Hz, 1H, Ar*H*), 7.41–7.38 (m, 2H, 2 × Ar*H*), 6.90 (dd, *J* = 8.5 Hz, *J* = 2.2 Hz, 1H, Ar*H*), 6.89–6.86 (m, 2H, 2 × Ar*H*), 6.70 (d, *J* = 2.2 Hz, 1H, Ar*H*), 5.65 (s, 2H, Ar‐C*H*_*2*_N), 3.84 (s, 3H, Ar*‐*OC*H*_3_), 2.81 (s, 3H, Ar*‐*C*H*_3_). ^13^C NMR (151 MHz, CDCl_3_) *δ* 161.1 (Ar*C*‐OCH_3_), 143.3 (Ar*C*‐N), 140.8 (Ar*C*‐N), 138.9 (Ar*C*H‐N), 136.9 (Ar*C*), 135.6 (Ar*C*‐N), 132.2 (2 × Ar*C*H), 129.5 (Ar*C*), 127.2 (2 × Ar*C*H), 122.5 (Ar*C*H), 121.4 (Ar*C*), 115.3 (Ar*C*), 112.4 (Ar*C*H), 109.3 (Ar*C*H), 93.3 (Ar*C*H), 55.6 (Ar‐O*C*H_3_), 47.8 (Ar‐*C*H_2_N), 23.0 (Ar‐*C*H_3_). ESI‐HRMS *m/z* calcd for C_20_H_18_BrN_2_O^+^ [M + H]^+^ 381.0597, found 381.0602; 98.9% purity.

7‐Methoxy‐1‐methyl‐9‐[(2‐nitrophenyl)methyl]‐9*H*‐pyrido[3,4‐*b*]indole (**21**): Harmine **1** (40 mg; 0.188 mmol), NaH (8 mg; 0.197 mmol), 2‐nitrobenzyl bromide (43 mg; 0.197 mmol), DMF/THF (v/v = 1:1, 2 mL). The residue was purified by preparative TLC using mobile phase cHx/EtOAc/28% aq. sol. NH_4_OH (60:30:0.2), developed twice, to obtain pure product **21** as a white solid. Yield: 90%. ^1^H NMR (600 MHz, CDCl_3_) *δ* 8.32 (d, *J* = 5.2 Hz, 1H, Ar*H*), 8.27 (dd, *J* = 7.8 Hz, *J* = 1.4 Hz, 1H, Ar*H*), 8.04 (d, *J* = 8.6 Hz, 1H, Ar*H*), 7.79 (d, *J* = 5.2 Hz, 1H, Ar*H*), 7.45 (td, *J* = 7.8 Hz, *J* = 1.4 Hz, 1H, Ar*H*), 7.38 (td, *J* = 7.8 Hz, *J* = 1.4 Hz, 1H, Ar*H*), 6.93 (dd, *J* = 8.6 Hz, *J* = 2.1 Hz, 1H, Ar*H*), 6.65 (d, *J* = 2.1 Hz, 1H, Ar*H*), 6.54 (dd, *J* = 7.8 Hz, *J* = 1.4 Hz, 1H, Ar*H*), 6.10 (s, 2H, Ar‐C*H*_*2*_N), 3.84 (s, 3H, Ar*‐*OC*H*_3_), 2.69 (s, 3H, Ar*‐*C*H*_3_). ^13^C NMR (151 MHz, CDCl_3_) *δ* 161.3 (Ar*C*‐OCH_3_), 146.7 (Ar*C*‐NO_2_), 143.2 (Ar*C*‐N), 140.9 (Ar*C*‐N), 139.2 (Ar*C*H‐N), 135.4 (Ar*C*‐N), 134.7 (Ar*C*H), 134.2 (Ar*C*), 129.7 (Ar*C*), 128.6 (Ar*C*H), 127.7 (Ar*C*H), 125.7 (Ar*C*H), 122.7 (Ar*C*H), 115.3 (Ar*C*), 112.4 (Ar*C*H), 109.7 (Ar*C*H), 93.0 (Ar*C*H), 55.7 (Ar‐O*C*H_3_), 46.5 (Ar‐*C*H_2_N), 22.7 (Ar‐*C*H_3_). ESI‐HRMS *m/z* calcd for C_20_H_18_N_3_O_3_^+^ [M + H]^+^ 348.1343, found 348.1349; 98.6% purity.

7‐Methoxy‐1‐methyl‐9‐[(3‐nitrophenyl)methyl]‐9*H*‐pyrido[3,4‐*b*]indole (**22**): Harmine **1** (40 mg; 0.188 mmol), NaH (8 mg; 0.197 mmol), 3‐nitrobenzyl bromide (43 mg; 0.197 mmol), DMF/THF (v/v = 1:1, 2 mL). The residue was purified by preparative TLC using mobile phase cHx/EtOAc/28% aq. sol. NH_4_OH (60:30:0.2), developed twice, to obtain pure product **22** as a white solid. Yield: 88%. ^1^H NMR (600 MHz, CDCl_3_) *δ* 8.33 (d, *J* = 5.2 Hz, 1H, Ar*H*), 8.12 (dd, *J* = 7.8 Hz, *J* = 1.2 Hz, 1H, Ar*H*), 8.03 (d, *J* = 8.6 Hz, 1H, overlap, Ar*H*), 8.03–8.01 (m, 1H, overlap, Ar*H*), 7.79 (d, *J* = 5.2 Hz, 1H, Ar*H*), 7.43 (t, *J* = 7.8 Hz, 1H, Ar*H*), 7.19 (d, *J* = 7.8 Hz, 1H, Ar*H*), 6.93 (dd, *J* = 8.6 Hz, *J* = 2.1 Hz, 1H, Ar*H*), 6.69 (d, *J* = 2.1 Hz, 1H, Ar*H*), 5.81 (s, 2H, Ar‐C*H*_*2*_N), 3.85 (s, 3H, Ar*‐*OC*H*_3_), 2.81 (s, 3H, Ar*‐*C*H*_3_). ^13^C NMR (151 MHz, CDCl_3_) *δ* 161.3 (Ar*C*‐OCH_3_), 148.8 (Ar*C*‐NO_2_), 143.2 (Ar*C*‐N), 140.6 (Ar*C*‐N), 140.2 (Ar*C*), 139.3 (Ar*C*H‐N), 135.5 (Ar*C*‐N), 131.4 (Ar*C*H), 130.3 (Ar*C*H), 129.8 (Ar*C*), 122.8 (Ar*C*H), 122.7 (Ar*C*H), 120.6 (Ar*C*H), 115.5 (Ar*C*), 112.5 (Ar*C*H), 109.4 (Ar*C*H), 93.3 (Ar*C*H), 55.7 (Ar‐O*C*H_3_), 47.7 (Ar‐*C*H_2_N), 23.1 (Ar‐*C*H_3_). ESI‐HRMS *m/z* calcd for C_20_H_18_N_3_O_3_^+^ [M + H]^+^ 348.1343, found 348.1347; 99.7% purity.

7‐Methoxy‐1‐methyl‐9‐[(4‐nitrophenyl)methyl]‐9*H*‐pyrido[3,4‐*b*]indole (**23**): Harmine **1** (40 mg; 0.188 mmol), NaH (8 mg; 0.197 mmol), 4‐nitrobenzyl bromide (43 mg; 0.197 mmol), DMF/THF (v/v = 1:1, 2 mL). The residue was purified by preparative TLC using mobile phase cHx/EtOAc/28% aq. sol. NH_4_OH (60:30:0.2), developed twice, to obtain pure product **23** as a pale yellow solid. Yield: 86%. ^1^H NMR (600 MHz, CDCl_3_) *δ* 8.33 (d, *J* = 5.2 Hz, 1H, Ar*H*), 8.17–8.13 (m, 2H, 2 × Ar*H*), 8.04 (d, *J* = 8. Hz, 1H, Ar*H*), 7.79 (d, *J* = 5.2 Hz, 1H, Ar*H*), 7.18–7.16 (m, 2H, 2 × Ar*H*), 6.93 (dd, *J* 6 = 8.6 Hz, *J* = 2.1 Hz, 1H, Ar*H*), 6.68 (d, *J* = 2.1 Hz, 1H, Ar*H*), 5.81 (s, 2H, Ar‐C*H*_*2*_N), 3.85 (s, 3H, Ar*‐*OC*H*_3_), 2.80 (s, 3H, Ar*‐*C*H*_3_). ^13^C NMR (151 MHz, CDCl_3_) *δ* 161.3 (Ar*C*‐OCH_3_), 145.2 (Ar*C*‐NO_2_, Ar*C*), 143.2 (Ar*C*‐N), 140.6 (Ar*C*‐N), 139.3 (Ar*C*H‐N), 135.5 (Ar*C*‐N), 129.8 (Ar*C*), 126.4 (2 × Ar*C*H), 124.4 (2 × Ar*C*H), 122.7 (Ar*C*H), 115.4 (Ar*C*), 112.5 (Ar*C*H), 109.4 (Ar*C*H), 93.3 (Ar*C*H), 55.7 (Ar‐O*C*H_3_), 47.9 (Ar‐*C*H_2_N), 23.1 (Ar‐*C*H_3_). ESI‐HRMS *m/z* calcd for C_20_H_18_N_3_O_3_^+^ [M + H]^+^ 348.1343, found 348.1348; 98.1% purity.

7‐Methoxy‐1‐methyl‐9‐[(naphthalen‐2‐yl)methyl]‐9*H*‐pyrido[3,4‐*b*]indole (**24**): Harmine **1** (40 mg; 0.188 mmol), NaH (8 mg; 0.197 mmol), 2‐naphthylmethyl bromide (44 mg; 0.197 mmol), DMF/THF (v/v = 1:1, 2 mL). The residue was purified by preparative TLC using mobile phase EtOAc/MeOH/28% aq. sol. NH_4_OH (100:4:1.5) to obtain pure product **24** as a beige solid. Yield: 81%. ^1^H NMR (600 MHz, CDCl_3_) *δ* 8.32 (d, *J* = 5.3 Hz, 1H, Ar*H*), 8.04 (d, *J* = 8.6 Hz, 1H, Ar*H*), 7.82–7.79 (m, 3H, 3 × Ar*H*), 7.61 (dd, *J* = 7.7 Hz, *J* = 1.3 Hz, 1H, Ar*H*), 7.45–7.39 (m, 2H, 2 × Ar*H*), 7.34 (bs, 1H, Ar*H*), 7.25 (dd, *J* = 8.4 Hz, *J* = 1.7 Hz, 1H, Ar*H*), 6.91 (dd, *J* = 8.6 Hz, *J* = 2.1 Hz, 1H, Ar*H*), 6.78 (d, *J* = 2.1 Hz, 1H, Ar*H*), 5.88 (s, 2H, Ar‐C*H*_*2*_N), 3.82 (s, 3H, Ar*‐*OC*H*_3_), 2.85 (s, 3H, Ar*‐*C*H*_3_). ^13^C NMR (151 MHz, CDCl_3_) *δ* 161.1 (Ar*C*‐OCH_3_), 143.6 (Ar*C*‐N), 141.1 (Ar*C*‐N), 138.8 (Ar*C*H‐N), 135.9 (Ar*C*‐N), 135.4 (Ar*C*), 133.5 (Ar*C*), 132.8 (Ar*C*), 129.5 (Ar*C*), 129.0 (Ar*C*H), 127.8 (Ar*C*H), 127.7 (Ar*C*H), 126.4 (Ar*C*H), 126.0 (Ar*C*H), 124.1 (Ar*C*H), 123.5 (Ar*C*H), 122.5 (Ar*C*H), 115.3 (Ar*C*), 112.4 (Ar*C*H), 109.3 (Ar*C*H), 93.4 (Ar*C*H), 55.6 (Ar‐O*C*H_3_), 48.5 (Ar‐*C*H_2_N), 23.0 (Ar‐*C*H_3_). ESI‐HRMS *m/z* calcd for C_24_H_21_N_2_O_3_^+^ [M + H]^+^ 353.1648, found 353.1656; 99.7% purity.

7‐Methoxy‐1‐methyl‐9‐propyl‐9*H*‐pyrido[3,4‐*b*]indole (**25**): Harmine **1** (40 mg; 0.188 mmol), NaH (8 mg; 0.197 mmol), propyl bromide (18 μL; 0.197 mmol), DMF/THF (v/v = 1:1, 2 mL). The residue was purified by preparative TLC using mobile phase EtOAc/MeOH/28% aq. sol. NH_4_OH (100:4:1.5) to obtain pure product **25** as a white solid. Yield: 82%. ^1^H NMR (600 MHz, CDCl_3_) *δ* 8.27 (d, *J* = 5.3 Hz, 1H, Ar*H*), 7.96 (d, *J* = 8.5 Hz, 1H, Ar*H*), 7.72 (d, *J* = 5.3 Hz, 1H, Ar*H*), 6.87 (dd, *J* = 8.5 Hz, *J* = 2.2 Hz, 1H, Ar*H*), 6.85 (d, *J* = 2.2 Hz, 1H, Ar*H*), 4.43–4.40 (m, 2H, N‐C*H*_*2*_), 3.94 (s, 3H, Ar*‐*OC*H*_3_), 3.00 (s, 3H, Ar*‐*C*H*_3_), 1.90–1.83 (m, 2H, CH_2_), 1.00 (t, *J* = 7.4 Hz, 3H, CH_3_). ^13^C NMR (151 MHz, CDCl_3_) *δ* 160.8 (Ar*C*‐OCH_3_), 143.1 (Ar*C*‐N), 140.6 (Ar*C*‐N), 138.2 (Ar*C*H‐N), 135.4 (Ar*C*‐N), 129.3 (Ar*C*), 122.3 (Ar*C*H), 115.2 (Ar*C*), 112.2 (Ar*C*H), 108.5 (Ar*C*H), 93.5 (Ar*C*H), 55.7 (Ar‐O*C*H_3_), 46.4 (N‐*C*H_2_), 23.9 (*C*H_2_), 23.4 (Ar‐*C*H_3_), 11.2 (*C*H_3_). ESI‐HRMS *m/z* calcd for C_16_H_19_N_2_O^+^ [M + H]^+^ 255.1492, found 255.1501; 99.9% purity.

7‐Methoxy‐1‐methyl‐9‐(propan‐2‐yl)‐9*H*‐pyrido[3,4‐*b*]indole (**26**): Harmine **1** (40 mg; 0.188 mmol), NaH (8 mg; 0.197 mmol), isopropyl bromide (17 μL; 0.197 mmol), DMF/THF (v/v = 1:1, 2 mL). The residue was purified by preparative TLC using mobile phase EtOAc/MeOH/28% aq. sol. NH_4_OH (100:4:1.5) to obtain pure product **26** as a white solid. Yield: 58%. ^1^H NMR (600 MHz, CDCl_3_) *δ* 8.26 (d, *J* = 5.2 Hz, 1H, Ar*H*), 7.98 (d, *J* = 8.6 Hz, 1H, Ar*H*), 7.71 (d, *J* = 5.2 Hz, 1H, Ar*H*), 7.12 (d, *J* = 2.1 Hz, 1H, Ar*H*), 6.87 (dd, *J* = 8.6 Hz, *J* = 2.1 Hz, 1H, Ar*H*), 5.54–5.48 (m, 1H, N‐C*H*), 3.94 (s, 3H, Ar*‐*OC*H*_3_), 3.01 (s, 3H, Ar*‐*C*H*_3_), 1.72 (d, *J* = 7.0 Hz, 6H, CH(C*H*_3_)_2_). ^13^C NMR (151 MHz, CDCl_3_) *δ* 159.8 (Ar*C*‐OCH_3_), 141.2 (Ar*C*‐N), 140.4 (Ar*C*‐N), 138.1 (Ar*C*H‐N), 136.0 (Ar*C*‐N), 129.2 (Ar*C*), 122.3 (Ar*C*H), 116.7 (Ar*C*), 112.0 (Ar*C*H), 107.9 (Ar*C*H), 97.4 (Ar*C*H), 55.7 (Ar‐O*C*H_3_), 48.0 (N‐*C*H), 24.8 (Ar‐*C*H_3_), 21.2 (CH(*C*H_3_)_2_). ESI‐HRMS *m/z* calcd for C_16_H_19_N_2_O^+^ [M + H]^+^ 255.1492, found 255.1502; 99.7% purity.

7‐Methoxy‐1‐methyl‐9‐(prop‐2‐en‐1‐yl)‐9*H*‐pyrido[3,4‐*b*]indole (**27**): Harmine **1** (40 mg; 0.188 mmol), NaH (8 mg; 0.197 mmol), allyl bromide (17 μL; 0.197 mmol), DMF/THF (v/v = 1:1, 2 mL). The residue was purified by preparative TLC using mobile phase EtOAc/MeOH/28% aq. sol. NH_4_OH (100:4:1.5) to obtain pure product **27** as a white solid. Yield: 63%. ^1^H NMR (600 MHz, CDCl_3_) *δ* 8.28 (d, *J* = 5.3 Hz, 1H, Ar*H*), 8.00 (d, *J* = 8.6 Hz, 1H, Ar*H*), 7.83 (d, *J* = 5.3 Hz, 1H, Ar*H*), 6.94 (dd, *J* = 8.6 Hz, *J* = 2.1 Hz, 1H, Ar*H*), 6.80 (d, *J* = 2.1 Hz, 1H, Ar*H*), 6.08 (ddt, *J* = 17.2 Hz, *J* = 10.5 Hz, *J* = 4.0 Hz, 1H, C*H* = CH_2_), 5.21 (d, *J* = 10.5 Hz, 1H, CH = C*H*_*2*_), 5.12–5.10 (m, 2H, N‐C*H*_*2*_), 4.78 (d, *J* = 17.2 Hz, 1H, CH = C*H*_*2*_), 3.93 (s, 3H, Ar*‐*OC*H*_3_), 3.08 (s, 3H, Ar*‐*C*H*_3_). ^13^C NMR (151 MHz, CDCl_3_) *δ* 161.9 (Ar*C*‐OCH_3_), 144.3 (Ar*C*‐N), 139.5 (Ar*C*‐N), 135.6 (Ar*C*H‐N), 135.1 (Ar*C*‐N), 132.8 (Ar*C*H), 130.8 (Ar*C*), 122.9 (Ar*C*H), 116.8 (Ar*C*H), 114.6 (Ar*C*), 112.7 (Ar*C*H), 110.3 (Ar*C*H), 93.2 (Ar*C*H), 55.7 (Ar‐O*C*H_3_), 47.0 (N‐*C*H_2_), 21.0 (Ar‐*C*H_3_). ESI‐HRMS *m/z* calcd for C_16_H_17_N_2_O^+^ [M + H]^+^ 253.1335, found 253.1339; 98.6% purity.

7‐Methoxy‐1‐methyl‐9‐(prop‐2‐yn‐1‐yl)‐9*H*‐pyrido[3,4‐*b*]indole (**28**): Harmine **1** (40 mg; 0.188 mmol), NaH (8 mg; 0.197 mmol), propargyl bromide (18 μL; 0.197 mmol), DMF/THF (v/v = 1:1, 2 mL). The residue was purified by preparative TLC using mobile phase EtOAc/MeOH/28% aq. sol. NH_4_OH (100:4:1.5) to obtain pure product **28** as a white solid. Yield: 65%. ^1^H NMR (600 MHz, CDCl_3_) *δ* 8.30 (d, *J* = 5.2 Hz, 1H, Ar*H*), 7.95 (d, *J* = 9.1 Hz, 1H, Ar*H*), 7.70 (d, *J* = 5.2 Hz, 1H, Ar*H*), 6.90 (dd, *J* = 9.1 Hz, *J* = 2.2 Hz, 1H, overlap, Ar*H*), 6.90 (d, *J* = 2.2 Hz, 1H, overlap, Ar*H*), 5.18 (d, *J* = 2.5 Hz, 2H, N‐C*H*_*2*_), 3.95 (s, 3H, Ar*‐*OC*H*_3_), 3.09 (s, 3H, Ar*‐*C*H*_3_), 2.34 (t, *J* = 2.5 Hz, 1H, C≡C*H*). ^13^C NMR (151 MHz, CDCl_3_) *δ* 161.1 (Ar*C*‐OCH_3_), 142.7 (Ar*C*‐N), 141.0 (Ar*C*‐N), 139.1 (Ar*C*H‐N), 135.1 (Ar*C*‐N), 129.8 (Ar*C*), 122.5 (Ar*C*H), 115.5 (Ar*C*), 112.3 (Ar*C*H), 109.5 (Ar*C*H), 93.3 (Ar*C*H), 78.5 (*C*≡CH), 73.5 (C≡*C*H), 55.7 (Ar‐O*C*H_3_), 34.7 (N‐*C*H_2_), 23.0 (Ar‐*C*H_3_). ESI‐HRMS *m/z* calcd for C_16_H_15_N_2_O^+^ [M + H]^+^ 251.1179, found 251.1188; 99.8% purity.

9‐Butyl‐7‐methoxy‐1‐methyl‐9*H*‐pyrido[3,4‐*b*]indole (**29**): Harmine **1** (40 mg; 0.188 mmol), NaH (8 mg; 0.197 mmol), butyl bromide (21 μL; 0.197 mmol), DMF/THF (v/v = 1:1, 2 mL). The residue was purified by preparative TLC using mobile phase EtOAc/MeOH/28% aq. sol. NH_4_OH (100:4:1.5) to obtain pure product **29** as a white solid. Yield: 70%. ^1^H NMR (600 MHz, CDCl_3_) *δ* 8.27 (d, *J* = 5.2 Hz, 1H, Ar*H*), 7.96 (d, *J* = 8.5 Hz, 1H, Ar*H*), 7.72 (d, *J* = 5.2 Hz, 1H, Ar*H*), 6.87 (dd, *J* = 8.5 Hz, *J* = 2.2 Hz, 1H, Ar*H*), 6.85 (d, *J* = 2.2 Hz, 1H, Ar*H*), 4.47–4.43 (m, 2H, N‐C*H*_*2*_), 3.94 (s, 3H, Ar*‐*OC*H*_3_), 3.01 (s, 3H, Ar*‐*C*H*_3_), 1.84–1.78 (m, 2H, C*H*_2_), 1.48–1.41 (m, 2H, C*H*_2_), 0.98 (t, *J* = 7.4 Hz, 3H, C*H*_3_). ^13^C NMR (151 MHz, CDCl_3_) *δ* 160.8 (Ar*C*‐OCH_3_), 143.1 (Ar*C*‐N), 140.6 (Ar*C*‐N), 138.3 (Ar*C*H‐N), 135.3 (Ar*C*‐N), 129.3 (Ar*C*), 122.3 (Ar*C*H), 115.3 (Ar*C*), 112.2 (Ar*C*H), 108.5 (Ar*C*H), 93.5 (Ar*C*H), 55.7 (Ar‐O*C*H_3_), 44.7 (N‐*C*H_2_), 32.7 (*C*H_2_), 23.4 (Ar‐*C*H_3_), 20.2 (*C*H_2_), 13.9 (*C*H_3_). ESI‐HRMS *m/z* calcd for C_17_H_21_N_2_O^+^ [M + H]^+^ 269.1648, found 269.1659; 99.2% purity.

7‐Methoxy‐1‐methyl‐9‐pentyl‐9*H*‐pyrido[3,4‐*b*]indole (**30**): Harmine **1** (40 mg; 0.188 mmol), NaH (8 mg; 0.197 mmol), pentyl bromide (35 μL; 0.282 mmol), DMF/THF (v/v = 1:1, 2 mL). The residue was purified by preparative TLC using mobile phase EtOAc/MeOH/28% aq. sol. NH_4_OH (100:4:1.5) to obtain pure product **30** as a white solid. Yield: 78%. ^1^H NMR (600 MHz, CDCl_3_) *δ* 8.27 (d, *J* = 5.3 Hz, 1H, Ar*H*), 7.97 (d, *J* = 8.6 Hz, 1H, Ar*H*), 7.76 (d, *J* = 5.3 Hz, 1H, Ar*H*), 6.90 (dd, *J* = 8.6 Hz, *J* = 2.2 Hz, 1H, Ar*H*), 6.85 (d, *J* = 2.2 Hz, 1H, Ar*H*), 4.47–4.41 (m, 2H, N‐C*H*_*2*_), 3.94 (s, 3H, Ar*‐*OC*H*_3_), 3.06 (s, 3H, Ar*‐*C*H*_3_), 1.85–1.79 (m, 2H, C*H*_2_), 1.42–1.35 (m, 4H, 2 × C*H*_2_), 0.90 (t, *J* = 7.0 Hz, 3H, C*H*_3_). ^13^C NMR (151 MHz, CDCl_3_) *δ* 161.2 (Ar*C*‐OCH_3_), 143.5 (Ar*C*‐N), 139.9 (Ar*C*‐N), 136.9 (Ar*C*H‐N), 135.1 (Ar*C*‐N), 130.0 (Ar*C*), 122.6 (Ar*C*H), 115.0 (Ar*C*), 112.4 (Ar*C*H), 109.1 (Ar*C*H), 93.4 (Ar*C*H), 55.7 (Ar‐O*C*H_3_), 45.0 (N‐*C*H_2_), 30.3 (*C*H_2_), 29.0 (*C*H_2_), 22.5 (Ar‐*C*H_3_), 22.4 (*C*H_2_), 13.9 (*C*H_3_). ESI‐HRMS *m/z* calcd for C_18_H_23_N_2_O^+^ [M + H]^+^ 283.1805, found 283.1812; 99.3% purity.

9‐Hexyl‐7‐methoxy‐1‐methyl‐9*H*‐pyrido[3,4‐*b*]indole (**31**): Harmine **1** (40 mg; 0.188 mmol), NaH (8 mg; 0.197 mmol), hexyl bromide (40 μL; 0.282 mmol), DMF/THF (v/v = 1:1, 2 mL). The residue was purified by preparative TLC using mobile phase cHx/EtOAc/28% aq. sol. NH_4_OH (60:30:0.2), developed twice, to obtain pure product **31** as a white solid. Yield: 82%. ^1^H NMR (600 MHz, CDCl_3_) *δ* 8.26 (d, *J* = 5.4 Hz, 1H, Ar*H*), 7.97 (d, *J* = 8.6 Hz, 1H, Ar*H*), 7.77 (d, *J* = 5.4 Hz, 1H, Ar*H*), 6.90 (dd, *J* = 8.6 Hz, *J* = 2.2 Hz, 1H, Ar*H*), 6.84 (d, *J* = 2.2 Hz, 1H, Ar*H*), 4.46–4.42 (m, 2H, N‐C*H*_*2*_), 3.94 (s, 3H, Ar*‐*OC*H*_3_), 3.07 (s, 3H, Ar*‐*C*H*_3_), 1.85–1.77 (m, 2H, C*H*_2_), 1.45–1.39 (m, 2H, C*H*_2_), 1.36–1.28 (m, 4H, 2 × C*H*_2_), 0.87 (t, *J* = 7.2 Hz, 3H, C*H*_3_). ^13^C NMR (151 MHz, CDCl_3_) *δ* 161.4 (Ar*C*‐OCH_3_), 143.7 (Ar*C*‐N), 139.7 (Ar*C*‐N), 136.4 (Ar*C*H‐N), 135.0 (Ar*C*‐N), 130.2 (Ar*C*H), 122.6 (Ar*C*H), 114.9 (Ar*C*), 112.5 (Ar*C*H), 109.3 (Ar*C*H), 93.4 (Ar*C*H), 55.7 (Ar‐O*C*H_3_), 45.0 (N‐*C*H_2_), 31.5 (*C*H_2_), 30.6 (*C*H_2_), 26.5 (*C*H_2_), 22.5 (*C*H_2_), 22.3 (Ar‐*C*H_3_), 13.9 (*C*H_3_). ESI‐HRMS *m/z* calcd for C_19_H_25_N_2_O^+^ [M + H]^+^ 297.1961, found 297.1964; 99.7% purity.

9‐(4‐Bromobutyl)‐7‐methoxy‐1‐methyl‐9*H*‐pyrido[3,4‐*b*]indole (**32**): Harmine **1** (40 mg; 0.188 mmol), NaH (8 mg; 0.197 mmol), 1,4‐dibromobutane (33 μL; 0.282 mmol), DMF/THF (v/v = 1:1, 2 mL). The residue was purified by preparative TLC using mobile phase cHx/EtOAc/28% aq. sol. NH_4_OH (60:30:0.2), developed twice, to obtain pure product **32** as a white solid. Yield: 76%. ^1^H NMR (600 MHz, CDCl_3_) *δ* 8.29 (d, *J* = 5.3 Hz, 1H, Ar*H*), 7.98 (d, *J* = 8.6 Hz, 1H, Ar*H*), 7.76 (d, *J* = 5.3 Hz, 1H, Ar*H*), 6.90 (dd, *J* = 8.6 Hz, *J* = 2.2 Hz, 1H, Ar*H*), 6.87 (d, *J* = 2.2 Hz, 1H, Ar*H*), 4.54–4.50 (m, 2H, N‐C*H*_*2*_), 3.95 (s, 3H, Ar*‐*OC*H*_3_), 3.57 (t, *J* = 6.3 Hz, 2H, C*H*_2_), 3.05 (s, 3H, Ar*‐*C*H*_3_), 2.05–1.99 (m, 2H, C*H*_2_), 1.92–1.86 (m, 2H,C*H*_2_). ^13^C NMR (151 MHz, CDCl_3_) *δ* 161.1 (Ar*C*‐OCH_3_), 143.1 (Ar*C*‐N), 140.2 (Ar*C*‐N), 138.1 (Ar*C*H‐N), 135.2 (Ar*C*‐N), 129.7 (Ar*C*), 122.5 (Ar*C*H), 115.2 (Ar*C*), 112.4 (Ar*C*H), 109.0 (Ar*C*H), 93.3 (Ar*C*H), 55.7 (Ar‐O*C*H_3_), 44.0 (N‐*C*H_2_), 32.8 (*C*H_2_), 29.7 (*C*H_2_), 29.0 (*C*H_2_), 23.2 (Ar‐*C*H_3_). ESI‐HRMS *m/z* calcd for C_17_H_20_BrN_2_O^+^ [M + H]^+^ 347.0754, found 347.0759; 96.0% purity.

9‐(5‐Bromopentyl)‐7‐methoxy‐1‐methyl‐9*H*‐pyrido[3,4‐*b*]indole (**33**): Harmine **1** (40 mg; 0.188 mmol), NaH (8 mg; 0.197 mmol), 1,5‐dibromopentane (39 μL; 0.282 mmol), DMF/THF (v/v = 1:1, 2 mL). The residue was purified by preparative TLC using mobile phase cHx/EtOAc/28% aq. sol. NH_4_OH (60:30:0.2), developed twice, to obtain pure product **33** as a white solid. Yield: 75%. ^1^H NMR (600 MHz, CDCl_3_) *δ* 8.28 (d, *J* = 5.2 Hz, 1H, Ar*H*), 7.97 (d, *J* = 8.6 Hz, 1H, Ar*H*), 7.74 (d, *J* = 5.2 Hz, 1H, Ar*H*), 6.89 (dd, *J* = 8.6 Hz, *J* = 2.2 Hz, 1H, Ar*H*), 6.84 (d, *J* = 2.2 Hz, 1H, Ar*H*), 4.50–4.45 (m, 2H, N‐C*H*_*2*_), 3.95 (s, 3H Ar*‐*OC*H*_3_), 3.39 (t, *J* = 6.6 Hz, 2H, C*H*_2_), 3.02 (s, 3H, Ar*‐*C*H*_3_), 1.93–1.80 (m, 4H, 2 × C*H*_2_), 1.61–1.56 (m, 2H, C*H*_2_). ^13^C NMR (151 MHz, CDCl_3_) *δ* 160.9 (Ar*C*‐OCH_3_), 143.0 (Ar*C*‐N), 140.5 (Ar*C*‐N), 138.4 (Ar*C*H‐N), 135.2 (Ar*C*‐N), 129.4 (Ar*C*), 122.4 (Ar*C*H), 115.3 (Ar*C*), 112.3 (Ar*C*H), 108.7 (Ar*C*H), 93.4 (Ar*C*H), 55.7 (Ar‐O*C*H_3_), 44.7 (N‐*C*H_2_), 33.2 (*C*H_2_), 32.2 (*C*H_2_), 29.7 (*C*H_2_), 25.4 (*C*H_2_), 23.4 (Ar‐*C*H_3_). ESI‐HRMS *m/z* calcd for C_18_H_22_BrN_2_O^+^ [M + H]^+^ 361.0910, found 361.0922; 98.6% purity.

9‐(6‐Bromohexyl)‐7‐methoxy‐1‐methyl‐9*H*‐pyrido[3,4‐*b*]indole (**34**): Harmine **1** (40 mg; 0.188 mmol), NaH (8 mg; 0.197 mmol), 1,6‐dibromohexane (43 μL; 0.282 mmol), DMF/THF (v/v = 1:1, 2 mL). The residue was purified by preparative TLC using mobile phase cHx/EtOAc/28% aq. sol. NH_4_OH (60:30:0.2), developed twice, to obtain pure product **34** as a white solid. Yield: 77%. ^1^H NMR (600 MHz, CDCl_3_) *δ* 8.28 (d, *J* = 5.3 Hz, 1H, Ar*H*), 7.98 (d, *J* = 8.6 Hz, 1H, Ar*H*), 7.76 (d, *J* = 5.3 Hz, 1H, Ar*H*), 6.90 (dd, *J* = 8.6 Hz, *J* = 2.1 Hz, 1H, Ar*H*), 6.85 (d, *J* = 2.1 Hz, 1H, Ar*H*), 4.50–4.45 (m, 2H, N‐C*H*_*2*_), 3.95 (s, 3H, Ar*‐*OC*H*_3_), 3.51 (t, *J* = 6.6 Hz, 2H, C*H*_2_), 3.04 (s, 3H, Ar*‐*C*H*_3_), 1.88–1.82 (m, 2H, C*H*_2_), 1.79–1.74 (m, 2H, C*H*_2_), 1.54–1.42 (m, 4H, 2 × C*H*_2_). ^13^C NMR (151 MHz, CDCl_3_) *δ* 160.9 (Ar*C*‐OCH_3_), 143.1 (Ar*C*‐N), 140.4 (Ar*C*‐N), 138.2 (Ar*C*H‐N), 135.3 (Ar*C*‐N), 129.4 (Ar*C*), 122.4 (Ar*C*H), 115.2 (Ar*C*), 112.3 (Ar*C*H), 108.6 (Ar*C*H), 93.5 (Ar*C*H), 55.7 (Ar‐O*C*H_3_), 44.7 (N‐*C*H_2_), 33.5 (*C*H_2_), 32.5 (*C*H_2_), 30.4 (*C*H_2_), 27.9 (*C*H_2_), 26.1 (*C*H_2_), 23.3 (Ar‐*C*H_3_). ESI‐HRMS *m/z* calcd for C_19_H_24_BrN_2_O^+^ [M + H]^+^ 375.1067, found 375.1068; 96.4% purity.

### 4.2 In Vitro Biological Evaluation

#### 4.2.1 Screening of Antiproliferative Activity

Selected human tumor and non‐tumor cell lines Jurkat (acute T cell leukemia), MOLT‐4 (acute lymphoblastic leukemia), MOLM‐13 (acute myeloid leukemia), THP‐1 (acute monocytic leukemia), MV4‐11 (acute monocytic leukemia) A549 (lung carcinoma), HT‐29 (colorectal adenocarcinoma), PANC‐1 (pancreas epithelioid carcinoma), A2780 (ovarian carcinoma), MCF‐7 (breast adenocarcinoma), SAOS‐2 (osteosarcoma) and MRC‐5 (normal lung fibroblasts) were purchased from either ATCC (Manassas, USA), Merck Life Science (Prague, Czech Republic) or Deutsche Sammlung von Mikroorganismen und Zellkulturen (Braunschweig, Germany) and cultured according to the provider's culture method guidelines. All cell lines were maintained at 37°C in a humidified 5% carbon dioxide and 95% air incubator. Experiments were conducted using cell lines within strictly defined passage limits to ensure phenotypic stability: non‐tumor cell line (MRC‐5) was utilized within 10 passages, while tumor cell lines (Jurkat, MOLT‐4, MOLM‐13, THP‐1, MV4‐11, A549, HT‐29, PANC‐1, A2780, MCF‐7, and SAOS‐2) were maintained for a maximum of 20 passages. All assays were performed during the exponential growth phase to guarantee optimal metabolic activity and reproducibility.

#### 4.2.2 Screening for Antiproliferative Activity, Growth Percent, and IC_50_ Value Calculation

To begin the research, each cell line was seeded at a predetermined optimum density (1 × 10^3^ to 50 × 10^3^ cells per well) on a 96‐well plate (TPP, Trasadingen, Switzerland), and adherent cell lines were allowed to settle overnight. Cells were treated for 48 h with **1**, structurally related β‐carbolines, and prepared **1** derivatives at final concentrations of 10 µM (single‐dose screening) or between 0.1 and 1000 µM (IC_50_ values determination). A 1 µM concentration of doxorubicin (Sigma‐Aldrich, St. Louis, USA) served as a positive control. After the culture period, the WST‐1 proliferation assay (Merck Life Science, Prague, Czech Republic) was carried out according to the manufacturer's protocol, and the absorbance was measured using Tecan Spark (Tecan, Männedorf, Switzerland). Every value is the mean of at least three separate experiments and reflects the percentage of proliferation of 0.1% DMSO mock‐treated control cells (100%). Each compound examined had its growth percentage (GP) determined. GP reflects the average percentage decrease in proliferation of all cell lines treated with the same compound.

#### 4.2.3 Determination of Acute Myeloid Leukemia Cell Proliferation and Viability Using the MTT Assay

The experiments were performed in 96‐well plate formats. THP‐1 cells absent from FLT3 mutation were seeded at 3 × 10^4^ per well, and the twelve‐point concentration curve of the tested compound **6** ranged between 200 µM and 97.656 nM. MOLM cells (5 × 10^4^ per well) and MV4‐11 cells (3 × 10^4^ per well) were seeded in their respective RPMI and IMDM media, and the twelve‐point concentration curve of **6** ranged between 10 µM and 4.883 nM. After 48‐h incubation under the standard conditions, the MTT assay (Merck Life Science, Prague, Czech Republic) was performed according to the manufacturer's protocol. The spectrophotometric analysis was done on the Hidex Sense Microplate Reader at 570 and 720 nm wavelengths. All the experiments were performed as three biological replicates done in triplicates.

#### 4.2.4 Proliferation and Viability Measurement Using Trypan Blue Exclusion Assay

The Trypan blue exclusion assay was used to assess cell viability and proliferation. The cells were treated with different doses (5–25 µM) of **1** and **6** and cultivated for 24, 48, and 72 h. Cells treated with 0.1 µM doxorubicin served as the positive control. Cell membrane integrity was assessed using the Trypan blue exclusion method, which involved mixing 50 µL of 0.5% Trypan blue with 50 µL of cell suspension. For MRC‐5 adherent‐culture cells, floating cells were combined with adherent cells that had been trypsinized and resuspended in media prior to mixing with 0.5% Trypan blue. Cell counts were performed using a Bürker chamber and a Nikon Eclipse E200 light microscope (Nikon, Tokyo, Japan). Proliferation is represented as the percentage of the change in the number of cells relative to those reported in the 0.1% DMSO vehicle controls (negative control), which is regarded as 100%.

#### 4.2.5 Screening for Antiproliferative Activity Using the xCELLigence System

The xCELLigence system (Roche, Basel, Switzerland; ACEA Biosciences, San Diego, CA, USA) was used for monitoring cell adhesion, proliferation, and cytotoxicity. The real‐time cell analysis RTCA SP (single plate) station was connected and tested using Resistor Plate before being put in an incubator at 37°C with a 5% CO_2_ environment. First, the experiments' seeding concentrations were tuned for each cell line. The xCELLigence system evaluated cell growth, adhesion, and spreading every 30 min after seeding in 190 µL of media per well of an E‐plate 96. Approximately 24 h after seeding, cells in the log growth phase were treated with 10 µL of sterile deionized water containing **1** or compound **6** at final concentrations ranging from 1 to 50 μM. Controls got sterile deionized water + 0.1% DMSO. Cells treated with 5% DMSO were utilized as the positive control. Growth curves were standardized to the period of treatment. The evaluations were carried out using the xCELLigence 1.2.1 software (Roche, Basel, Switzerland, and ACEA Biosciences, San Diego, CA, USA).

#### 4.2.6 Monoamine Oxidase A Enzyme Inhibitory Activity

The monoamine oxidase A (MAO‐A) inhibitory activity was assessed using the following protocol [76]. Each well contained 50 µL of sample or solvent, 50 µL of a chromogenic solution (comprising 0.8 mM vanillic acid, 417 mM 4‐aminoantipyrine, and 4 U/mL horseradish peroxidase), 100 µL of 2.5 mM tyramine, and 50 µL of 8 U/mL MAO‐A. All reagents were prepared in potassium phosphate buffer (0.2 M, pH 7.6). Human MAO‐A was obtained from Sigma‐Aldrich (St. Louis, MO, USA), while other general laboratory reagents were sourced from Panreac Química S.L.U. (Barcelona, Spain). Water used in the experiments was purified using a Milli‐Q water purification system (TGI Pure Water Systems, Greenville, SC, USA). Absorbance was recorded at 490 nm every 5 min for a total duration of 30 min. The percentage of enzymatic inhibition was calculated as: Inhibition % = [1−/(*V*_S_ − *V*_C_)] × 100, where *V*_S_ is the reaction rate of the extract sample and *V*_C_ is the rate of control rate. Each assay was performed in four independent experiments. Statistical analysis was carried out using GraphPad Prism version 6.0c for Mac OS X (GraphPad Software, San Diego, CA, USA). Results were expressed as mean ± standard errors mean (SEM). The 50% inhibitory concentration (IC_50_) was estimated by means of a linear regression equation.

#### 4.2.7 Cell Cycle and DNA Fragmentation Analysis

Cells were collected by trypsinization, washed with ice‐cold Dulbecco's phosphate‐buffered saline (DPBS), fixed with 70% ethanol, and kept at 4°C for cell cycle distribution analysis. The fixed cells were then centrifuged to remove the ethanol before being rinsed twice with ice‐cold DPBS. To detect low molecular‐weight DNA fragments, cells were incubated in a buffer (192 mL 0.2 M Na_2_HPO_4_ + 8 mL of 0.1 M citric acid, pH 7.8) for 5 min at room temperature. After washing with ice‐cold DPBS, cells were labeled with propidium iodide in Vindelov's solution for 1 h at 37°C. The DNA content was measured using a Beckman Coulter CytoFLEX LX (Beckman Coulter, Miami, FL, USA) flow cytometer with an excitation wavelength of 488 nm and emission spectrum 600–620 nm. The data were analyzed using the Kaluza Analysis 2.1 software.

#### 4.2.8 Annexin V/PI Flow Cytometry Analysis of Apoptosis

Apoptosis was measured using flow cytometry with an Alexa Fluor 488 Annexin V/Dead Cell Apoptosis kit (Life Technologies, Grand Island, NY, USA) according to the manufacturer's instructions. The Alexa Fluor 488 Annexin V/Dead Cell Apoptosis kit leverages the affinity of Alexa Fluor 488‐conjugated Annexin V for phosphatidylserine exposed on the outer leaflet of apoptotic cell membranes in the presence of Ca^2+^ ions. Concurrently, the assay utilizes the capacity of propidium iodide (PI) to intercalate into DNA of cells with compromised membrane integrity. At least 20,000 events per sample were acquired on a CytoFLEX LX Flow Cytometer (Beckman Coulter, Miami, FL, USA). The list mode data were analyzed using Kaluza Analysis 2.1 software (Beckman Coulter, Miami, FL, USA).

#### 4.2.9 Activity of Caspases

The caspase‐dependent induction of programmed cell death was assessed by monitoring the activities of caspases‐3/7, caspase‐8, and caspase‐9 by Caspase‐Glo Assays (Promega, Madison, WI, USA) 24 h after treatment with 10 and 20 μM of compounds **6** and **1**. Cells treated with 5 µM of cisplatin served as positive controls. The test includes a proluminogenic substrate in an optimized buffer solution. The addition of a Caspase‐Glo Reagent causes cell lysis, caspase cleavage of the substrate, and the production of a luminous signal. Cells were seeded at 1 × 10^4^ cells per well in a white wall 96‐well plate (Sigma‐Aldrich, St. Louis, MO, USA). After treatment, 100 µL of Caspase‐Glo Assay Reagent was added to each well and incubated for 30 min. Luminescence was detected using a Tecan Spark multimode microplate reader (Tecan Group, Männedorf, Switzerland).

#### 4.2.10 Immunofluorescence Staining of Poly(ADP‐Ribose) (PAR), γH2AX and Epi‐fluorescence Microscopy

The MOLT‐4 cells in optimal seeding density were incubated in the presence of 0.1% DMSO (negative control), 0.1 μM doxorubicin (positive control), compounds **1** or **6** at 20 μM for 2, 6, and 24 h. After treatment, for each condition, 6 × 10^5^ cells were loaded on poly‐l‐lysine‐coated glass slides (Merck, Darmstadt, Germany) and were fixed with 4% freshly prepared paraformaldehyde for 10 min at room temperature, washed with DPBS, and permeabilized in 0.2% Triton X‐100/DPBS (all reagent from Merck, Darmstadt, Germany). Thereafter, MOLT‐4 cells were blocked in a solution with 7% FCS and 2% BSA and immunostained with a mouse monoclonal anti‐PAR (Enzo Life Sciences, San Diego, CA) or γH2AX primary antibody (Cell Signaling, Boston, MA, USA) overnight at 4°C. Then, the secondary donkey anti‐mouse IgG Alexa Fluor 488‐conjugated antibody (Jackson ImmunoResearch Laboratories, West Grove, PA, USA) was applied to each slide (after their preincubation with 5.0% donkey serum in DPBS for 30 min at room temperature), and incubation for 1 h in the dark was succeeded by washing (3 × 5 min) in DPBS. The nuclei were counterstained using a DAPI solution (Merck, Darmstadt, Germany). Finally, the slides were mounted with coverslips using ProLong Glass Antifade mountant (Thermo Fisher Scientific, Waltham, MA, USA). Images of all of the examined slides were obtained by a Nikon epi‐fluorescence microscope system Eclipse 80i; the exposure time and dynamic range of the camera in all of the channels were adjusted to the same values for all of the slides to portray quantitatively comparable images. The images were further processed and merged using NIS‐Elements Advanced Research 5.11.03 (instruments and software from Nikon, Tokyo, Japan).

#### 4.2.11 TUNEL Assay

To determine apoptosis‐associated DNA fragmentation by TUNEL assay, 1 × 10^6^ of MOLT‐4 cells treated for 24 or 48 h with compound **6** in contractions ranging from 5 µM to 25 µM were collected and evaluated using a DNA break labeling detection kit, APO‐DIRECT (Merck Millipore, Billerica, MA, USA) in accordance with the manufacturer's instructions. Cells were washed twice with DPBS at 4°C. Subsequently, 1 mL of fixation solution (1% formaldehyde) was added to the cell suspension for 30 min on ice. Cells were then centrifuged for 5 min at 300*g* and washed twice with 5 mL of DPBS. Cells were permeabilized in 70% ethanol for at least 24 h at −20°C. Following this, cells were washed twice with wash buffer and then incubated in a TUNEL reaction mixture (comprising deoxyuridine triphosphate—dUTP‐FITC, terminal deoxynucleotidyl transferase—TdT enzyme, TdT reaction buffer, and distilled H_2_O) for 60 min at 37°C. After incubation, the cells were washed twice more with rinse buffer and resuspended in 0.5 mL of propidium iodide/RNase A solution. Samples were analyzed on a CytoFLEX LX Flow Cytometer (Beckman Coulter, Miami, FL, USA) to measure green fluorescence (dUTP‐FITC incorporated into fragmented DNA) and red fluorescence (PI bound to DNA) in individual cell nuclei. Resulting listmode data were analyzed using Kaluza Analysis 2.1 software (Beckman Coulter, Miami, FL, USA).

#### 4.2.12 Detection of ROS in Cells

MOLT‐4 cells were seeded at a density of 4 × 10⁵ cells/mL and exposed to compounds **1** or **6** at a concentration of 20 µM for 24 h. Cells treated with tert‐butyl hydroperoxide (TBHP) at 200 μM for 1 h in the cultivation medium were used as a positive control for ROS induction. The ROS generation was subsequently quantified by adding CellROX Green reagent at a final concentration of 500 nM, following the instructions provided in the CellROX Green Flow Cytometry Assay Kit (Thermo Fisher Scientific, Waltham, MA, USA) manual. SYTOX Red Dead Cell Stain incorporated in the kit was used in combination for assessing cell viability. Fluorescence was measured using a CytoFLEX LX Flow Cytometer (Beckman Coulter, Miami, FL, USA). Data analysis was performed using Kaluza Analysis 2.1 software (Beckman Coulter, Miami, FL, USA).

#### 4.2.13 Western Blot Analysis

Whole‐cell lysates (Cell Lysis Buffer, Cell Signaling Technology, Danvers, MA, USA) were collected 4 h after MOLT‐4 cells were treated with 10 and 15 μM of compound **6**. Cells treated with 0.1% DMSO were used as a negative control. The cells treated with 5 μM cisplatin served as positive controls. The protein concentration was measured using the BCA assay (Sigma‐Aldrich, St. Louis, MO, USA). The lysates were loaded onto polyacrylamide gel lanes (20 µg of pure protein), and then they were electrophoresed. After electrophoresis separation, the proteins were transferred to a PVDF membrane (Bio‐Rad, Hercules, CA, USA). Non‐specific membrane bindings were blocked for 1 h in Tris‐buffered saline (TBS) with 0.05% Tween 20% and 5%, w/v non‐fat dry milk. The membranes were then rinsed in TBS. Primary antibodies against particular antigens pRb (phospho‐Rb (Ser807/811)); AKT; pAKT (phospho‐AKT (Thr308)); p38 MAPK; phospho‐p38 MAPK (phospho‐p38 (Thr180/Tyr182)); p44/42 MAPK (Erk1/2); phospho‐p44/42 MAPK (Erk1/2, (Thr202/Tyr204)); SAPK/JNK; p‐SAPK/JNK (Thr183/Tyr185); p27; Bcl‐2; and Bax from Cell Signaling Technology (Danvers, MA, USA) and β‐actin from Sigma‐Aldrich (St. Louis, MO, USA) were incubated overnight at 4°C. The membranes were washed five times with TBS with 0.05% Tween 20 for 10 min each, and once with TBS for 10 min. They were then incubated with a secondary antibody (DakoCytomation, Glostrup, Denmark or Cell Signaling Technology, Danvers, MA, USA) for 1 h at room temperature. A chemiluminescence detection kit (Roche, Basel, Switzerland) was used to detect the bands utilizing the GeneTools analysis system for images (Syngene Cambridge, UK). Each membrane was probed again, and β‐actin was determined in order to ensure equal protein loading. Densitometric analysis of Western blot bands was performed using ImageJ software, a Java‐based, open‐source image processing program developed by the National Institutes of Health (NIH) in the United States of America.

### 4.3 ADME Prediction of Drug‑Likeness and Toxicity

The physicochemical properties and pharmacokinetic profiles of selected compounds were assessed using SwissADME, a free online tool made by the Swiss Institute of Bioinformatics (SIB) [71]. It predicted molecular weight (MW), number of hydrogen bond donors (HBD) and acceptors (HBA), topological polar surface area (TPSA), lipophilicity (log P), solubility (ESOL), gastrointestinal (GI) absorption, blood–brain barrier (BBB) permeability, and P‐glycoprotein (P‐gp) substrate status of each compound using SMILES (Simplified Molecular Input Line Entry System) notations. ProTox‐3.0 was used to predict the toxicity of the selected compounds. It is a freely available online platform that can be used for in silico toxicity prediction [73]. It uses machine learning methods to estimate the median lethal doses (LD_50_) of compounds and place them into toxicity classes based on the Globally Harmonized System (GHS) for chemical categorization. The prediction workflow comprised two steps. First, the molecular structures of the selected compounds were generated using ChemDraw Professional 20.0 (PerkinElmer Informatics) and converted into their corresponding SMILES notation. Second, each SMILES string was submitted to the SwissADME or ProTox‐3.0, respectively.

### 4.4 Statistical Analysis of Data

The descriptive statistics of the data were computed, and the charts were created with either Microsoft Office 365 Excel (Microsoft, Redmond, WA, USA) or GraphPad Prism 7 biostatistics (GraphPad Software, La Jolla, CA, USA). Unless otherwise specified, all values in this study were given as arithmetic means with standard deviations for triplicates. Normality testing was conducted on quantitative data to determine whether parametric or nonparametric tests should be applied. For trials using parametric variables, the unpaired Student's *t*‐test was used to determine significant differences between groups. The degree of significance is indicated by *p < 0.05, **p < 0.01, and ***p < 0.001. IC_50_ values were determined from a WST‐1 proliferation assay conducted across a broad concentration range. Dose‐response curves were generated and analyzed using nonlinear regression in GraphPad Prism 7 software (GraphPad Software, San Diego, CA, USA) to calculate IC_50_ values.

## Author Contributions

**Abdul Aziz Timbilla:** conceptualization, writing – original draft, methodology, investigation, formal analysis, data curation, visualization. **Filip Pidany:** investigations, methodology, writing – review and editing, data curation, formal analysis, visualization. **Eliska Kohelova:** writing – review and editing, formal analysis, methodology, validation. **Jana Kroustkova:** investigation, data curation, writing – review and editing, validation. **Karel Kralovec:** investigation, methodology, data curation, writing – review and editing, visualization, validation. **Jan Rataj:** investigation, data curation, writing – review and editing, validation. **Martina Ceckova:** investigation, data curation, writing – review andand editing, validation. **Negar Maafi:** data curation, investigation, writing – review and editing, formal analysis, validation, visualization. **Víctor Lopez:** investigation, data curation, formal analysis, writing – review and editing, validation, visualization. **Cristina Moliner Langa:** investigation, data curation, formal analysis, writing – review and editing, validation, visualization. **Stefan Kosturko:** investigation, data curation. **Jaroslav Jenco:** investigation, data curation, formal analysis, writing – review and editing, validation. **Darina Muthna:** investigation, data curation, formal analysis, writing – review and editing, validation. **Darja Koutova:** investigation, data curation, formal analysis, writing – review and editing, validation. **Martina Rezacova:** supervision, conceptualization, writing – review and editing, validation, funding acquisition, funding acquisition, project administration. **Lucie Cahlikova:** conceptualization, writing – editing and reviewing, validation. **Jakub Chlebek:** conceptualization, supervision, writing – original draft, investigation, methodology, formal analysis, data curation, validation, visualization. **Radim Havelek:** supervision, conceptualization, writing – original draft, investigation, methodology, data curation, visualization, validation, funding acquisition.

## Conflicts of Interest

The authors declare no conflicts of interest.

## Declaration of Generative AI and AI‐Assisted Technologies in the Writing Process

During the preparation of this manuscript, the authors utilized the Grammarly tool to enhance the clarity and language of the text. All content was subsequently reviewed and revised by the authors, who take full responsibility for the final version of the published article.

## Supporting information

Supplementary materials.

ArchPharm SupplMat InChI Timbilla et al man.
