# Supplementary material for: Harmine Derivatives as Anticancer Agents Endowed With Potent and Selective Antileukemia Activity: Synthesis, Biological Evaluation, Proapoptotic and Genotoxic Activity
Source: Arch Pharm (Weinheim). 2026 Feb 10;359(2):e70197. doi: 10.1002/ardp.70197 (PMC12892019; doi:10.1002/ardp.70197)
Supplement: Supplementary file 1 — Supplementary materials. [file ARDP-359-e70197-s002.docx]

Supplementary material

Harmine derivatives as anticancer agents endowed with potent and selective antileukemia activity: Synthesis, biological evaluation, proapoptotic and genotoxic activity

Abdul Aziz Timbilla^1^, Filip Pidany^2^, Eliska Kohelova^2^, Jana Kroustkova^2^, Karel Kralovec^3^, Jan Rataj^4^, Martina Ceckova^4^, Negar Maafi^2^, Víctor Lopez^5^, Cristina Moliner Langa^5^, Stefan Kosturko^2^, Jaroslav Jenco^2^, Darina Muthna^1^, Darja Koutova^1^, Martina Rezacova^1^, Lucie Cahlikova^2^, Jakub Chlebek^2,^ *, Radim Havelek^1,^ *

^1^Department of Medical Biochemistry, Faculty of Medicine in Hradec Kralove, Charles University, Simkova 870, Hradec Kralove 500 03, Czech Republic

^2^Department of Pharmacognosy and Pharmaceutical Botany, Faculty of Pharmacy, Charles University, Akademika Heyrovskeho 1203, Hradec Kralove 500 05, Czech Republic

^3^Department of Biological and Biochemical Sciences, Faculty of Chemical Technology, University of Pardubice, Studentska 573, Pardubice 532 10, Czech Republic

^4^Department of Pharmacology and Toxicology, Faculty of Pharmacy in Hradec Kralove, Charles University, Akademika Heyrovskeho 1203, Hradec Kralove 500 05, Czech Republic

^5^Faculty of Health Sciences, Universidad San Jorge, 50830 Villanueva de Gállego (Zaragoza), Spain

**Table of contents**

[Figure S1: ^1^H NMR, ^13^C NMR, ESI-HRMS spectra and HPLC-UV chromatogram of prepared compounds 7](#_Toc219110065)

[^1^H NMR spectrum of 9‐benzyl‐7‐methoxy‐1‐methyl‐9*H*‐pyrido[3,4‐*b*]indole (2): 7](#_Toc219110066)

[^13^C NMR spectrum of 9‐benzyl‐7‐methoxy‐1‐methyl‐9*H*‐pyrido[3,4‐*b*]indole (2): 7](#_Toc219110067)

[ESI-HRMS spectrum of 9‐benzyl‐7‐methoxy‐1‐methyl‐9*H*‐pyrido[3,4‐*b*]indole (2): 8](#_Toc219110068)

[HPLC-UV chromatogram of 9‐benzyl‐7‐methoxy‐1‐methyl‐9*H*‐pyrido[3,4‐*b*]indole (2): 8](#_Toc219110069)

[^1^H NMR spectrum of 7‐methoxy‐1‐methyl‐9‐[(3‐methylphenyl)methyl]‐9*H*‐pyrido[3,4‐*b*]indole (3): 9](#_Toc219110070)

[^13^C NMR spectrum of 7‐methoxy‐1‐methyl‐9‐[(3‐methylphenyl)methyl]‐9*H*‐pyrido[3,4‐*b*]indole (3): 9](#_Toc219110071)

[ESI-HRMS spectrum of 7‐methoxy‐1‐methyl‐9‐[(3‐methylphenyl)methyl]‐9*H*‐pyrido[3,4‐*b*]indole (3): 10](#_Toc219110072)

[HPLC-UV chromatogram of 7‐methoxy‐1‐methyl‐9‐[(3‐methylphenyl)methyl]‐9*H*‐pyrido[3,4‐*b*]indole (3): 10](#_Toc219110073)

[^1^H NMR spectrum of 7‐methoxy‐1‐methyl‐9‐[(4‐methylphenyl)methyl]‐9*H*‐pyrido[3,4‐*b*]indole (4): 11](#_Toc219110074)

[^13^C NMR spectrum of 7‐methoxy‐1‐methyl‐9‐[(4‐methylphenyl)methyl]‐9*H*‐pyrido[3,4‐*b*]indole (4): 11](#_Toc219110075)

[ESI-HRMS spectrum of 7‐methoxy‐1‐methyl‐9‐[(4‐methylphenyl)methyl]‐9*H*‐pyrido[3,4‐*b*]indole (4): 12](#_Toc219110076)

[HPLC-UV chromatogram of 7‐methoxy‐1‐methyl‐9‐[(4‐methylphenyl)methyl]‐9*H*‐pyrido[3,4‐*b*]indole (4): 12](#_Toc219110077)

[^1^H NMR spectrum of 9‐[(2,4‐dimethylphenyl)methyl]‐7‐methoxy‐1‐methyl‐9*H*‐pyrido[3,4‐*b*]indole (5): 13](#_Toc219110078)

[^13^C NMR spectrum of 9‐[(2,4‐dimethylphenyl)methyl]‐7‐methoxy‐1‐methyl‐9*H*‐pyrido[3,4‐*b*]indole (5): 13](#_Toc219110079)

[ESI-HRMS spectrum of 9‐[(2,4‐dimethylphenyl)methyl]‐7‐methoxy‐1‐methyl‐9*H*‐pyrido[3,4‐*b*]indole (5): 14](#_Toc219110080)

[HPLC-UV chromatogram of 9‐[(2,4‐dimethylphenyl)methyl]‐7‐methoxy‐1‐methyl‐9*H*‐pyrido[3,4‐*b*]indole (5): 14](#_Toc219110081)

[^1^H NMR spectrum of 9‐[(3,5‐dimethylphenyl)methyl]‐7‐methoxy‐1‐methyl‐3*H*,4*H*,9*H*‐pyrido[3,4‐*b*]indole (6): 15](#_Toc219110082)

[^13^C NMR spectrum of 9‐[(3,5‐dimethylphenyl)methyl]‐7‐methoxy‐1‐methyl‐3*H*,4*H*,9*H*‐pyrido[3,4‐*b*]indole (6): 15](#_Toc219110083)

[ESI-HRMS spectrum of 9‐[(3,5‐dimethylphenyl)methyl]‐7‐methoxy‐1‐methyl‐3*H*,4*H*,9*H*‐pyrido[3,4‐*b*]indole (6): 16](#_Toc219110084)

[HPLC-UV chromatogram of 9‐[(3,5‐dimethylphenyl)methyl]‐7‐methoxy‐1‐methyl‐3*H*,4*H*,9*H*‐pyrido[3,4‐*b*]indole (6): 16](#_Toc219110085)

[^1^H NMR spectrum of 7‐methoxy‐1‐methyl‐9‐{[4‐(propan‐2‐yl)phenyl]methyl}‐9*H*‐pyrido[3,4‐*b*]indole (7): 17](#_Toc219110086)

[^13^C NMR spectrum of 7‐methoxy‐1‐methyl‐9‐{[4‐(propan‐2‐yl)phenyl]methyl}‐9*H*‐pyrido[3,4‐*b*]indole (7): 17](#_Toc219110087)

[ESI-HRMS spectrum of 7‐methoxy‐1‐methyl‐9‐{[4‐(propan‐2‐yl)phenyl]methyl}‐9*H*‐pyrido[3,4‐*b*]indole (7): 18](#_Toc219110088)

[HPLC-UV chromatogram of 7‐methoxy‐1‐methyl‐9‐{[4‐(propan‐2‐yl)phenyl]methyl}‐9*H*‐pyrido[3,4‐*b*]indole (7): 18](#_Toc219110089)

[^1^H NMR spectrum of 9‐[(4‐tert‐butylphenyl)methyl]‐7‐methoxy‐1‐methyl‐9*H*‐pyrido[3,4‐*b*]indole (8): 19](#_Toc219110090)

[^13^C NMR spectrum of 9‐[(4‐tert‐butylphenyl)methyl]‐7‐methoxy‐1‐methyl‐9*H*‐pyrido[3,4‐*b*]indole (8): 19](#_Toc219110091)

[ESI-HRMS spectrum of 9‐[(4‐tert‐butylphenyl)methyl]‐7‐methoxy‐1‐methyl‐9*H*‐pyrido[3,4‐*b*]indole (8): 20](#_Toc219110092)

[HPLC-UV chromatogram of 9‐[(4‐tert‐butylphenyl)methyl]‐7‐methoxy‐1‐methyl‐9*H*‐pyrido[3,4‐*b*]indole (8): 20](#_Toc219110093)

[^1^H NMR spectrum of 7‐methoxy‐9‐[(3‐methoxyphenyl)methyl]‐1‐methyl‐9*H*‐pyrido[3,4‐*b*]indole (9): 21](#_Toc219110094)

[^13^C NMR spectrum of 7‐methoxy‐9‐[(3‐methoxyphenyl)methyl]‐1‐methyl‐9*H*‐pyrido[3,4‐*b*]indole (9): 21](#_Toc219110095)

[ESI-HRMS spectrum of 7‐methoxy‐9‐[(3‐methoxyphenyl)methyl]‐1‐methyl‐9*H*‐pyrido[3,4‐*b*]indole (9): 22](#_Toc219110096)

[HPLC-UV chromatogram of 7‐methoxy‐9‐[(3‐methoxyphenyl)methyl]‐1‐methyl‐9*H*‐pyrido[3,4‐*b*]indole (9): 22](#_Toc219110097)

[^1^H NMR spectrum of 9‐[(3,5‐dimethoxyphenyl)methyl]‐7‐methoxy‐1‐methyl‐9*H*‐pyrido[3,4‐*b*]indole (10): 23](#_Toc219110098)

[^13^C NMR spectrum of 9‐[(3,5‐dimethoxyphenyl)methyl]‐7‐methoxy‐1‐methyl‐9*H*‐pyrido[3,4‐*b*]indole (10): 23](#_Toc219110099)

[ESI-HRMS spectrum of 9‐[(3,5‐dimethoxyphenyl)methyl]‐7‐methoxy‐1‐methyl‐9*H*‐pyrido[3,4‐*b*]indole (10): 24](#_Toc219110100)

[HPLC-UV chromatogram of 9‐[(3,5‐dimethoxyphenyl)methyl]‐7‐methoxy‐1‐methyl‐9*H*‐pyrido[3,4‐*b*]indole (10): 24](#_Toc219110101)

[^1^H NMR spectrum of 9‐[(2‐fluorophenyl)methyl]‐7‐methoxy‐1‐methyl‐9*H*‐pyrido[3,4‐*b*]indole (11): 25](#_Toc219110102)

[^13^C NMR spectrum of 9‐[(2‐fluorophenyl)methyl]‐7‐methoxy‐1‐methyl‐9*H*‐pyrido[3,4‐*b*]indole (11): 25](#_Toc219110103)

[ESI-HRMS spectrum of 9‐[(2‐fluorophenyl)methyl]‐7‐methoxy‐1‐methyl‐9*H*‐pyrido[3,4‐*b*]indole (11): 26](#_Toc219110104)

[HPLC-UV chromatogram of 9‐[(2‐fluorophenyl)methyl]‐7‐methoxy‐1‐methyl‐9*H*‐pyrido[3,4‐*b*]indole (11): 26](#_Toc219110105)

[^1^H NMR spectrum of 9‐[(3‐fluorophenyl)methyl]‐7‐methoxy‐1‐methyl‐9*H*‐pyrido[3,4‐*b*]indole (12): 27](#_Toc219110106)

[^13^C NMR spectrum of 9‐[(3‐fluorophenyl)methyl]‐7‐methoxy‐1‐methyl‐9*H*‐pyrido[3,4‐*b*]indole (12): 27](#_Toc219110107)

[ESI-HRMS spectrum of 9‐[(3‐fluorophenyl)methyl]‐7‐methoxy‐1‐methyl‐9*H*‐pyrido[3,4‐*b*]indole (12): 28](#_Toc219110108)

[HPLC-UV chromatogram of 9‐[(3‐fluorophenyl)methyl]‐7‐methoxy‐1‐methyl‐9*H*‐pyrido[3,4‐*b*]indole (12): 28](#_Toc219110109)

[^1^H NMR spectrum of 9‐[(4‐fluorophenyl)methyl]‐7‐methoxy‐1‐methyl‐9*H*‐pyrido[3,4‐*b*]indole (13): 29](#_Toc219110110)

[^13^C NMR spectrum of 9‐[(4‐fluorophenyl)methyl]‐7‐methoxy‐1‐methyl‐9*H*‐pyrido[3,4‐*b*]indole (13): 29](#_Toc219110111)

[ESI-HRMS spectrum of 9‐[(4‐fluorophenyl)methyl]‐7‐methoxy‐1‐methyl‐9*H*‐pyrido[3,4‐*b*]indole (13): 30](#_Toc219110112)

[HPLC-UV chromatogram of 9‐[(4‐fluorophenyl)methyl]‐7‐methoxy‐1‐methyl‐9*H*‐pyrido[3,4‐*b*]indole (13): 30](#_Toc219110113)

[^1^H NMR spectrum of 9‐[(2‐chlorophenyl)methyl]‐7‐methoxy‐1‐methyl‐9*H*‐pyrido[3,4‐*b*]indole (14): 31](#_Toc219110114)

[^13^C NMR spectrum of 9‐[(2‐chlorophenyl)methyl]‐7‐methoxy‐1‐methyl‐9*H*‐pyrido[3,4‐*b*]indole (14): 31](#_Toc219110115)

[ESI-HRMS spectrum of 9‐[(2‐chlorophenyl)methyl]‐7‐methoxy‐1‐methyl‐9*H*‐pyrido[3,4‐*b*]indole (14): 32](#_Toc219110116)

[HPLC-UV chromatogram of 9‐[(2‐chlorophenyl)methyl]‐7‐methoxy‐1‐methyl‐9*H*‐pyrido[3,4‐*b*]indole (14): 32](#_Toc219110117)

[^1^H NMR spectrum of 9‐[(3‐chlorophenyl)methyl]‐7‐methoxy‐1‐methyl‐9*H*‐pyrido[3,4‐*b*]indole (15): 33](#_Toc219110118)

[^13^C NMR spectrum of 9‐[(3‐chlorophenyl)methyl]‐7‐methoxy‐1‐methyl‐9*H*‐pyrido[3,4‐*b*]indole (15): 33](#_Toc219110119)

[ESI-HRMS spectrum of 9‐[(3‐chlorophenyl)methyl]‐7‐methoxy‐1‐methyl‐9*H*‐pyrido[3,4‐*b*]indole (15): 34](#_Toc219110120)

[HPLC-UV chromatogram of 9‐[(3‐chlorophenyl)methyl]‐7‐methoxy‐1‐methyl‐9*H*‐pyrido[3,4‐*b*]indole (15): 34](#_Toc219110121)

[^1^H NMR spectrum of 9‐[(4‐chlorophenyl)methyl]‐7‐methoxy‐1‐methyl‐9*H*‐pyrido[3,4‐*b*]indole (16): 35](#_Toc219110122)

[^13^C NMR spectrum of 9‐[(4‐chlorophenyl)methyl]‐7‐methoxy‐1‐methyl‐9*H*‐pyrido[3,4‐*b*]indole (16): 35](#_Toc219110123)

[ESI-HRMS spectrum of 9‐[(4‐chlorophenyl)methyl]‐7‐methoxy‐1‐methyl‐9*H*‐pyrido[3,4‐*b*]indole (16): 36](#_Toc219110124)

[HPLC-UV chromatogram of 9‐[(4‐chlorophenyl)methyl]‐7‐methoxy‐1‐methyl‐9*H*‐pyrido[3,4‐*b*]indole (16): 36](#_Toc219110125)

[^1^H NMR spectrum of 9‐[(3,4‐dichlorophenyl)methyl]‐7‐methoxy‐1‐methyl‐9*H*‐pyrido[3,4‐*b*]indole (17): 37](#_Toc219110126)

[^13^C NMR spectrum of 9‐[(3,4‐dichlorophenyl)methyl]‐7‐methoxy‐1‐methyl‐9*H*‐pyrido[3,4‐*b*]indole (17): 37](#_Toc219110127)

[ESI-HRMS spectrum of 9‐[(3,4‐dichlorophenyl)methyl]‐7‐methoxy‐1‐methyl‐9*H*‐pyrido[3,4‐*b*]indole (17): 38](#_Toc219110128)

[HPLC-UV chromatogram of 9‐[(3,4‐dichlorophenyl)methyl]‐7‐methoxy‐1‐methyl‐9*H*‐pyrido[3,4‐*b*]indole (17): 38](#_Toc219110129)

[^1^H NMR spectrum of 9‐[(2‐bromophenyl)methyl]‐7‐methoxy‐1‐methyl‐9*H*‐pyrido[3,4‐*b*]indole (18): 39](#_Toc219110130)

[^13^C NMR spectrum of 9‐[(2‐bromophenyl)methyl]‐7‐methoxy‐1‐methyl‐9*H*‐pyrido[3,4‐*b*]indole (18): 39](#_Toc219110131)

[ESI-HRMS spectrum of 9‐[(2‐bromophenyl)methyl]‐7‐methoxy‐1‐methyl‐9*H*‐pyrido[3,4‐*b*]indole (18): 40](#_Toc219110132)

[HPLC-UV chromatogram of 9‐[(2‐bromophenyl)methyl]‐7‐methoxy‐1‐methyl‐9*H*‐pyrido[3,4‐*b*]indole (18): 40](#_Toc219110133)

[^1^H NMR spectrum of 9‐[(3‐bromophenyl)methyl]‐7‐methoxy‐1‐methyl‐9*H*‐pyrido[3,4‐*b*]indole (19): 41](#_Toc219110134)

[^13^C NMR spectrum of 9‐[(3‐bromophenyl)methyl]‐7‐methoxy‐1‐methyl‐9*H*‐pyrido[3,4‐*b*]indole (19): 41](#_Toc219110135)

[ESI-HRMS spectrum of 9‐[(3‐bromophenyl)methyl]‐7‐methoxy‐1‐methyl‐9*H*‐pyrido[3,4‐*b*]indole (19): 42](#_Toc219110136)

[HPLC-UV chromatogram of 9‐[(3‐bromophenyl)methyl]‐7‐methoxy‐1‐methyl‐9*H*‐pyrido[3,4‐*b*]indole (19): 42](#_Toc219110137)

[^1^H NMR spectrum of 9‐[(4‐bromophenyl)methyl]‐7‐methoxy‐1‐methyl‐9*H*‐pyrido[3,4‐*b*]indole (20): 43](#_Toc219110138)

[^13^C NMR spectrum of 9‐[(4‐bromophenyl)methyl]‐7‐methoxy‐1‐methyl‐9*H*‐pyrido[3,4‐*b*]indole (20): 43](#_Toc219110139)

[ESI-HRMS spectrum of 9‐[(4‐bromophenyl)methyl]‐7‐methoxy‐1‐methyl‐9*H*‐pyrido[3,4‐*b*]indole (20): 44](#_Toc219110140)

[HPLC-UV chromatogram of 9‐[(4‐bromophenyl)methyl]‐7‐methoxy‐1‐methyl‐9*H*‐pyrido[3,4‐*b*]indole (20): 44](#_Toc219110141)

[^1^H NMR spectrum of 7‐methoxy‐1‐methyl‐9‐[(2‐nitrophenyl)methyl]‐9*H*‐pyrido[3,4‐*b*]indole (21): 45](#_Toc219110142)

[^13^C NMR spectrum of 7‐methoxy‐1‐methyl‐9‐[(2‐nitrophenyl)methyl]‐9*H*‐pyrido[3,4‐*b*]indole (21): 45](#_Toc219110143)

[ESI-HRMS spectrum of 7‐methoxy‐1‐methyl‐9‐[(2‐nitrophenyl)methyl]‐9*H*‐pyrido[3,4‐*b*]indole (21): 46](#_Toc219110144)

[HPLC-UV chromatogram of 7‐methoxy‐1‐methyl‐9‐[(2‐nitrophenyl)methyl]‐9*H*‐pyrido[3,4‐*b*]indole (21): 46](#_Toc219110145)

[^1^H NMR spectrum of 7‐methoxy‐1‐methyl‐9‐[(3‐nitrophenyl)methyl]‐9*H*‐pyrido[3,4‐*b*]indole (22): 47](#_Toc219110146)

[^13^C NMR spectrum of 7‐methoxy‐1‐methyl‐9‐[(3‐nitrophenyl)methyl]‐9*H*‐pyrido[3,4‐*b*]indole (22): 47](#_Toc219110147)

[ESI-HRMS spectrum of 7‐methoxy‐1‐methyl‐9‐[(3‐nitrophenyl)methyl]‐9*H*‐pyrido[3,4‐*b*]indole (22): 48](#_Toc219110148)

[HPLC-UV chromatogram of 7‐methoxy‐1‐methyl‐9‐[(3‐nitrophenyl)methyl]‐9*H*‐pyrido[3,4‐*b*]indole (22): 48](#_Toc219110149)

[^1^H NMR spectrum of 7‐methoxy‐1‐methyl‐9‐[(4‐nitrophenyl)methyl]‐9H‐pyrido[3,4‐*b*]indole (23): 49](#_Toc219110150)

[^13^C NMR spectrum of 7‐methoxy‐1‐methyl‐9‐[(4‐nitrophenyl)methyl]‐9H‐pyrido[3,4‐*b*]indole (23): 49](#_Toc219110151)

[ESI-HRMS spectrum of 7‐methoxy‐1‐methyl‐9‐[(4‐nitrophenyl)methyl]‐9H‐pyrido[3,4‐*b*]indole (23): 50](#_Toc219110152)

[HPLC-UV chromatogram of 7‐methoxy‐1‐methyl‐9‐[(4‐nitrophenyl)methyl]‐9H‐pyrido[3,4‐*b*]indole (23): 50](#_Toc219110153)

[^1^H NMR spectrum of 7‐methoxy‐1‐methyl‐9‐[(naphthalen‐2‐yl)methyl]‐9*H*‐pyrido[3,4‐*b*]indole (24): 51](#_Toc219110154)

[^13^C NMR spectrum of 7‐methoxy‐1‐methyl‐9‐[(naphthalen‐2‐yl)methyl]‐9*H*‐pyrido[3,4‐*b*]indole (24): 51](#_Toc219110155)

[ESI-HRMS spectrum of 7‐methoxy‐1‐methyl‐9‐[(naphthalen‐2‐yl)methyl]‐9*H*‐pyrido[3,4‐*b*]indole (24): 52](#_Toc219110156)

[HPLC-UV chromatogram of 7‐methoxy‐1‐methyl‐9‐[(naphthalen‐2‐yl)methyl]‐9*H*‐pyrido[3,4‐*b*]indole (24): 52](#_Toc219110157)

[^1^H NMR spectrum of 7‐methoxy‐1‐methyl‐9‐propyl‐9*H*‐pyrido[3,4‐*b*]indole (25): 53](#_Toc219110158)

[^13^C NMR spectrum of 7‐methoxy‐1‐methyl‐9‐propyl‐9*H*‐pyrido[3,4‐*b*]indole (25): 53](#_Toc219110159)

[ESI-HRMS spectrum of 7‐methoxy‐1‐methyl‐9‐propyl‐9*H*‐pyrido[3,4‐*b*]indole (25): 54](#_Toc219110160)

[HPLC-UV chromatogram of 7‐methoxy‐1‐methyl‐9‐propyl‐9*H*‐pyrido[3,4‐*b*]indole (25): 54](#_Toc219110161)

[^1^H NMR spectrum of 7‐methoxy‐1‐methyl‐9‐(propan‐2‐yl)‐9*H*‐pyrido[3,4‐*b*]indole (26): 55](#_Toc219110162)

[^13^C NMR spectrum of 7‐methoxy‐1‐methyl‐9‐(propan‐2‐yl)‐9*H*‐pyrido[3,4‐*b*]indole (26): 55](#_Toc219110163)

[ESI-HRMS spectrum of 7‐methoxy‐1‐methyl‐9‐(propan‐2‐yl)‐9*H*‐pyrido[3,4‐*b*]indole (26): 56](#_Toc219110164)

[HPLC-UV chromatogram of 7‐methoxy‐1‐methyl‐9‐(propan‐2‐yl)‐9*H*‐pyrido[3,4‐*b*]indole (26): 56](#_Toc219110165)

[^1^H NMR spectrum of 7‐methoxy‐1‐methyl‐9‐(prop‐2‐en‐1‐yl)‐9*H*‐pyrido[3,4‐*b*]indole (27): 57](#_Toc219110166)

[^13^C NMR spectrum of 7‐methoxy‐1‐methyl‐9‐(prop‐2‐en‐1‐yl)‐9*H*‐pyrido[3,4‐*b*]indole (27): 57](#_Toc219110167)

[ESI-HRMS spectrum of 7‐methoxy‐1‐methyl‐9‐(prop‐2‐en‐1‐yl)‐9*H*‐pyrido[3,4‐*b*]indole (27): 58](#_Toc219110168)

[HPLC-UV chromatogram of 7‐methoxy‐1‐methyl‐9‐(prop‐2‐en‐1‐yl)‐9*H*‐pyrido[3,4‐*b*]indole (27): 58](#_Toc219110169)

[^1^H NMR spectrum of 7‐methoxy‐1‐methyl‐9‐(prop‐2‐yn‐1‐yl)‐9*H*‐pyrido[3,4‐*b*]indole (28): 59](#_Toc219110170)

[^13^C NMR spectrum of 7‐methoxy‐1‐methyl‐9‐(prop‐2‐yn‐1‐yl)‐9*H*‐pyrido[3,4‐*b*]indole (28): 59](#_Toc219110171)

[ESI-HRMS spectrum of 7‐methoxy‐1‐methyl‐9‐(prop‐2‐yn‐1‐yl)‐9*H*‐pyrido[3,4‐*b*]indole (28): 60](#_Toc219110172)

[HPLC-UV chromatogram of 7‐methoxy‐1‐methyl‐9‐(prop‐2‐yn‐1‐yl)‐9*H*‐pyrido[3,4‐*b*]indole (28): 60](#_Toc219110173)

[^1^H NMR spectrum of 9‐butyl‐7‐methoxy‐1‐methyl‐9*H*‐pyrido[3,4‐*b*]indole (29): 61](#_Toc219110174)

[^13^C NMR spectrum of 9‐butyl‐7‐methoxy‐1‐methyl‐9*H*‐pyrido[3,4‐*b*]indole (29): 61](#_Toc219110175)

[ESI-HRMS spectrum of 9‐butyl‐7‐methoxy‐1‐methyl‐9*H*‐pyrido[3,4‐*b*]indole (29): 62](#_Toc219110176)

[HPLC-UV chromatogram of 9‐butyl‐7‐methoxy‐1‐methyl‐9*H*‐pyrido[3,4‐*b*]indole (29): 62](#_Toc219110177)

[^1^H NMR spectrum of 7‐methoxy‐1‐methyl‐9‐pentyl‐9*H*‐pyrido[3,4‐*b*]indole (30): 63](#_Toc219110178)

[^13^C NMR spectrum of 7‐methoxy‐1‐methyl‐9‐pentyl‐9*H*‐pyrido[3,4‐*b*]indole (30): 63](#_Toc219110179)

[ESI-HRMS spectrum of 7‐methoxy‐1‐methyl‐9‐pentyl‐9*H*‐pyrido[3,4‐*b*]indole (30): 64](#_Toc219110180)

[HPLC-UV chromatogram of 7‐methoxy‐1‐methyl‐9‐pentyl‐9*H*‐pyrido[3,4‐*b*]indole (30): 64](#_Toc219110181)

[^1^H NMR spectrum of 9‐hexyl‐7‐methoxy‐1‐methyl‐9*H*‐pyrido[3,4‐*b*]indole (31): 65](#_Toc219110182)

[^13^C NMR spectrum of 9‐hexyl‐7‐methoxy‐1‐methyl‐9*H*‐pyrido[3,4‐*b*]indole (31): 65](#_Toc219110183)

[ESI-HRMS spectrum of 9‐hexyl‐7‐methoxy‐1‐methyl‐9*H*‐pyrido[3,4‐*b*]indole (31): 66](#_Toc219110184)

[HPLC-UV chromatogram of 9‐hexyl‐7‐methoxy‐1‐methyl‐9*H*‐pyrido[3,4‐*b*]indole (31): 66](#_Toc219110185)

[^1^H NMR spectrum of 9‐(4‐bromobutyl)‐7‐methoxy‐1‐methyl‐9H‐pyrido[3,4‐*b*]indole (32): 67](#_Toc219110186)

[^13^C NMR spectrum of 9‐(4‐bromobutyl)‐7‐methoxy‐1‐methyl‐9H‐pyrido[3,4‐*b*]indole (32): 67](#_Toc219110187)

[ESI-HRMS spectrum of 9‐(4‐bromobutyl)‐7‐methoxy‐1‐methyl‐9H‐pyrido[3,4‐*b*]indole (32): 68](#_Toc219110188)

[HPLC-UV chromatogram of 9‐(4‐bromobutyl)‐7‐methoxy‐1‐methyl‐9H‐pyrido[3,4‐*b*]indole (32): 68](#_Toc219110189)

[^1^H NMR spectrum of 9‐(5‐bromopentyl)‐7‐methoxy‐1‐methyl‐9*H*‐pyrido[3,4‐*b*]indole (33): 69](#_Toc219110190)

[^13^C NMR spectrum of 9‐(5‐bromopentyl)‐7‐methoxy‐1‐methyl‐9*H*‐pyrido[3,4‐*b*]indole (33): 69](#_Toc219110191)

[ESI-HRMS spectrum of 9‐(5‐bromopentyl)‐7‐methoxy‐1‐methyl‐9*H*‐pyrido[3,4‐*b*]indole (33): 70](#_Toc219110192)

[HPLC-UV chromatogram of 9‐(5‐bromopentyl)‐7‐methoxy‐1‐methyl‐9*H*‐pyrido[3,4‐*b*]indole (33): 70](#_Toc219110193)

[^1^H NMR spectrum of 9‐(6‐bromohexyl)‐7‐methoxy‐1‐methyl‐9*H*‐pyrido[3,4‐*b*]indole (34): 71](#_Toc219110194)

[^13^C NMR spectrum of 9‐(6‐bromohexyl)‐7‐methoxy‐1‐methyl‐9*H*‐pyrido[3,4‐*b*]indole (34): 71](#_Toc219110195)

[ESI-HRMS spectrum of 9‐(6‐bromohexyl)‐7‐methoxy‐1‐methyl‐9*H*‐pyrido[3,4‐*b*]indole (34): 72](#_Toc219110196)

[HPLC-UV chromatogram of 9‐(6‐bromohexyl)‐7‐methoxy‐1‐methyl‐9*H*‐pyrido[3,4‐*b*]indole (34): 72](#_Toc219110197)

[Figure S2. 73](#_Toc219110198)

[Figure S3. 75](#_Toc219110199)

[Figure S4. 76](#_Toc219110200)

# Figure S1: ^1^H NMR, ^13^C NMR, ESI-HRMS spectra and HPLC-UV chromatogram **of prepared compounds**

# ^1^H NMR spectrum of 9‐benzyl‐7‐methoxy‐1‐methyl‐9*H*‐pyrido[3,4‐*b*]indole (2):


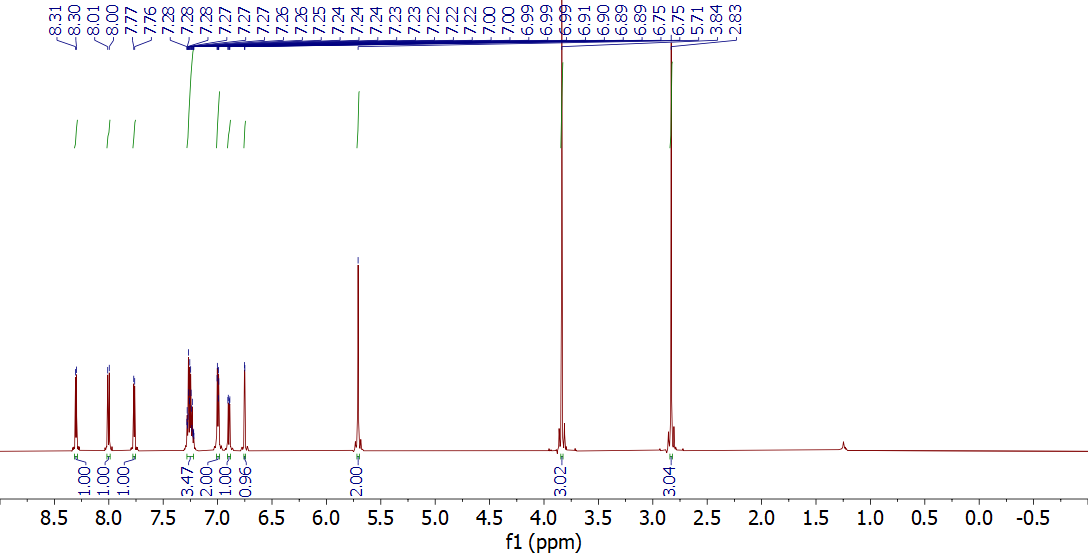


# ^13^C NMR spectrum of 9‐benzyl‐7‐methoxy‐1‐methyl‐9*H*‐pyrido[3,4‐*b*]indole (2):


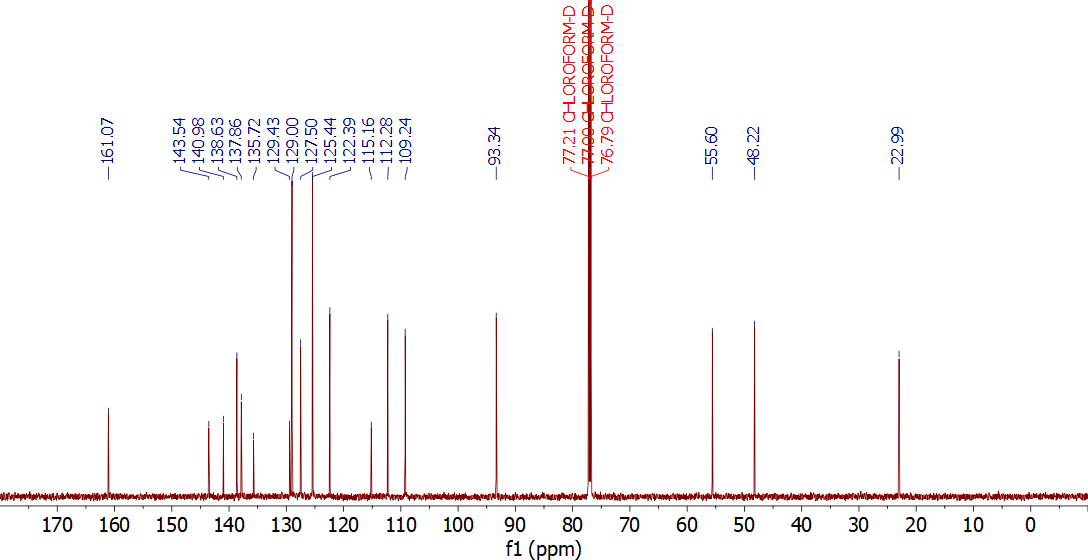


# ESI-HRMS spectrum of 9‐benzyl‐7‐methoxy‐1‐methyl‐9*H*‐pyrido[3,4‐*b*]indole (2):


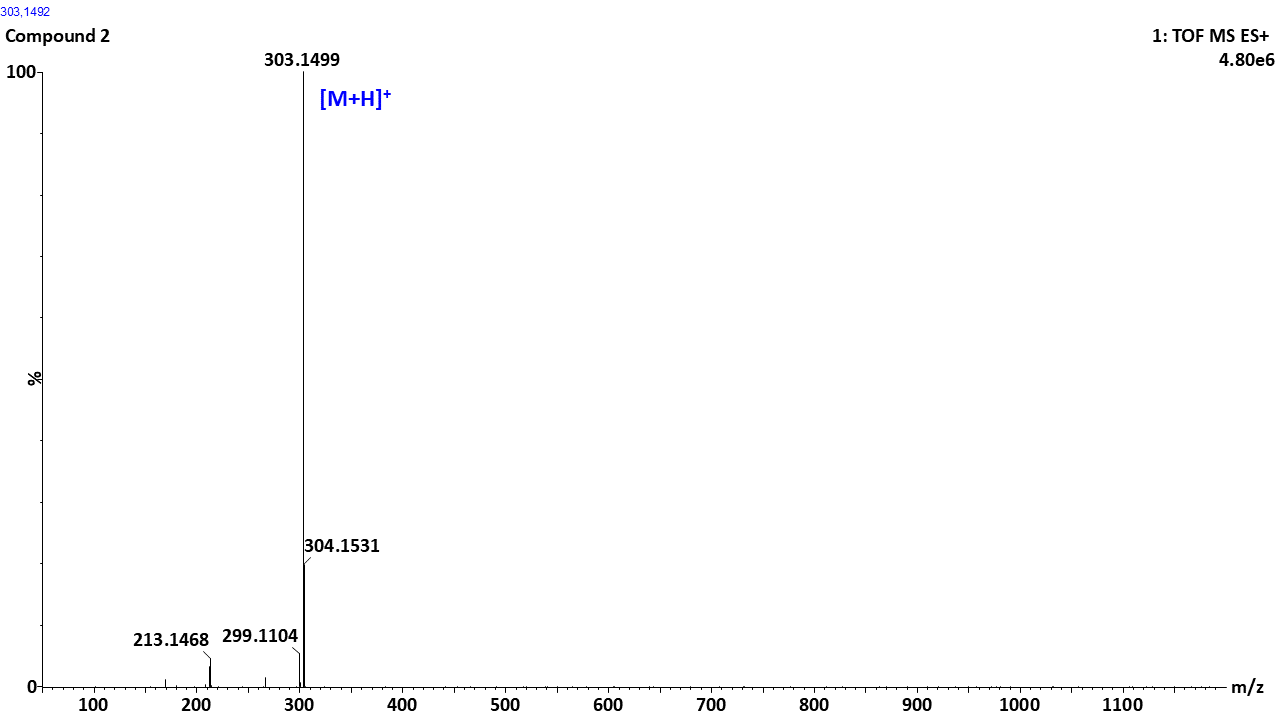


# HPLC-UV chromatogram of 9‐benzyl‐7‐methoxy‐1‐methyl‐9*H*‐pyrido[3,4‐*b*]indole (2):

# ^1^H NMR spectrum of 7‐methoxy‐1‐methyl‐9‐[(3‐methylphenyl)methyl]‐9*H*‐pyrido[3,4‐*b*]indole (3):


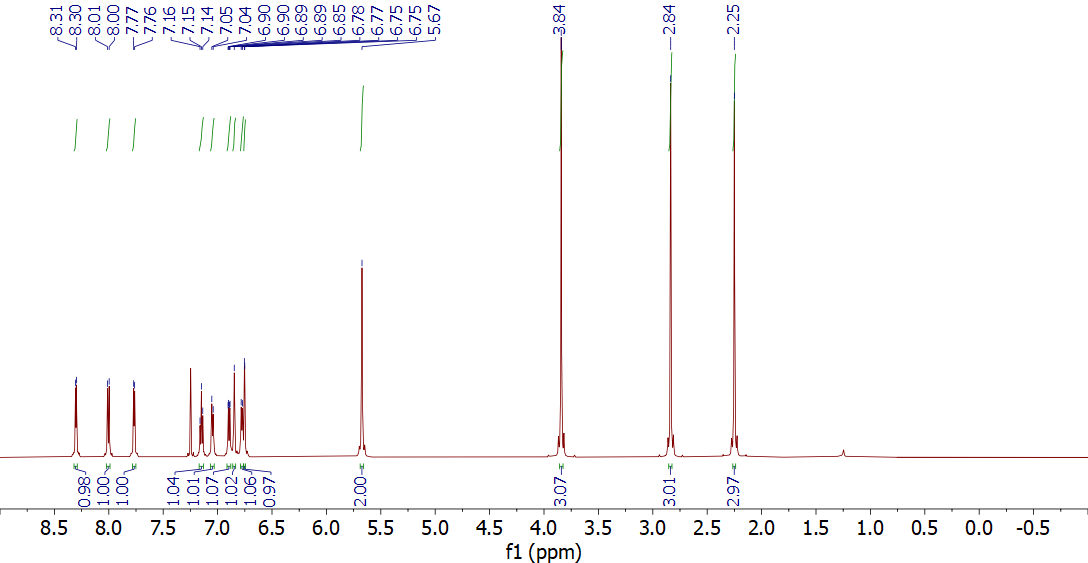


# ^13^C NMR spectrum of 7‐methoxy‐1‐methyl‐9‐[(3‐methylphenyl)methyl]‐9*H*‐pyrido[3,4‐*b*]indole (3):


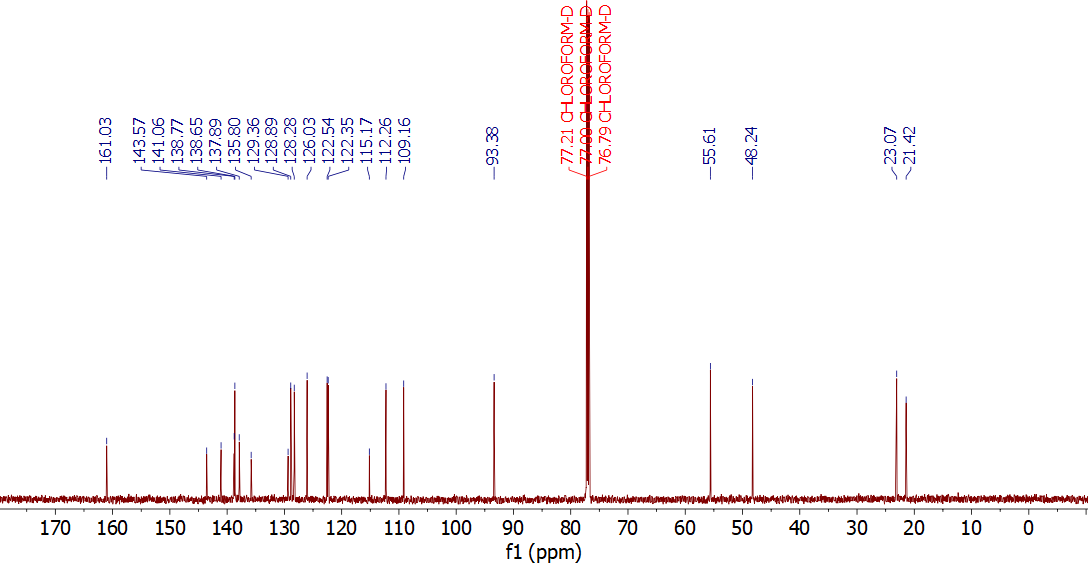


# ESI-HRMS spectrum of 7‐methoxy‐1‐methyl‐9‐[(3‐methylphenyl)methyl]‐9*H*‐pyrido[3,4‐*b*]indole (3):


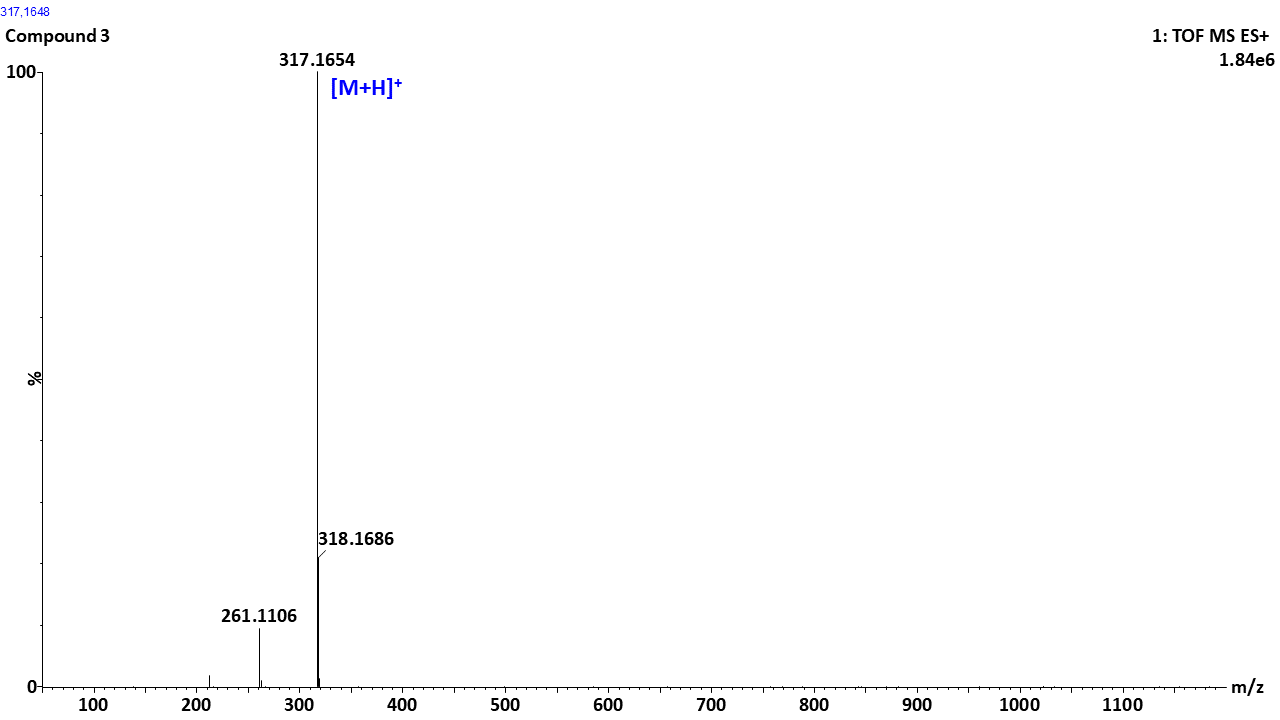


# HPLC-UV chromatogram of 7‐methoxy‐1‐methyl‐9‐[(3‐methylphenyl)methyl]‐9*H*‐pyrido[3,4‐*b*]indole (3):

# ^1^H NMR spectrum of 7‐methoxy‐1‐methyl‐9‐[(4‐methylphenyl)methyl]‐9*H*‐pyrido[3,4‐*b*]indole (4):

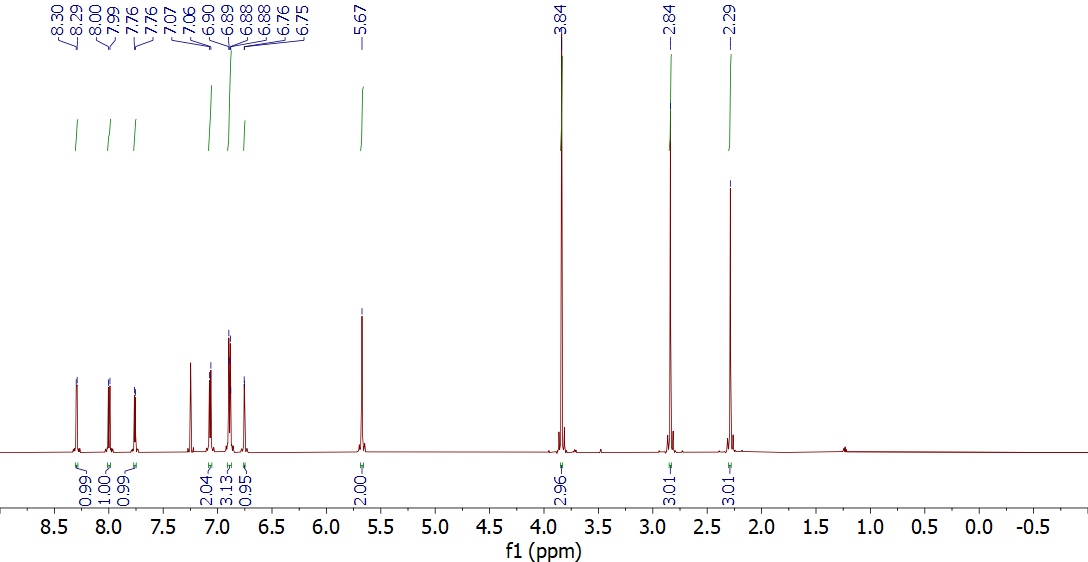


# ^13^C NMR spectrum of 7‐methoxy‐1‐methyl‐9‐[(4‐methylphenyl)methyl]‐9*H*‐pyrido[3,4‐*b*]indole (4):


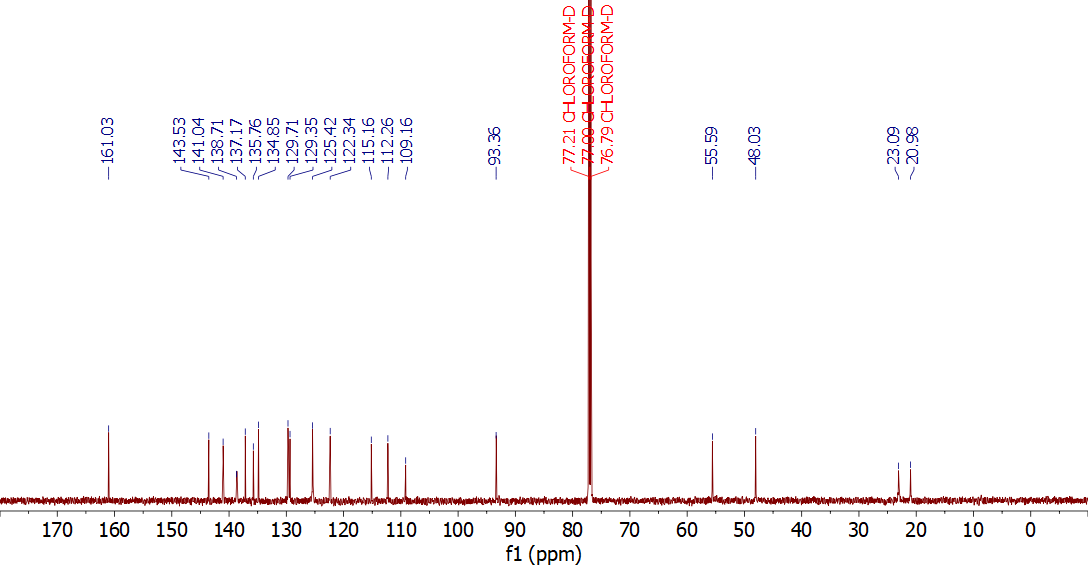


# ESI-HRMS spectrum of 7‐methoxy‐1‐methyl‐9‐[(4‐methylphenyl)methyl]‐9*H*‐pyrido[3,4‐*b*]indole (4):


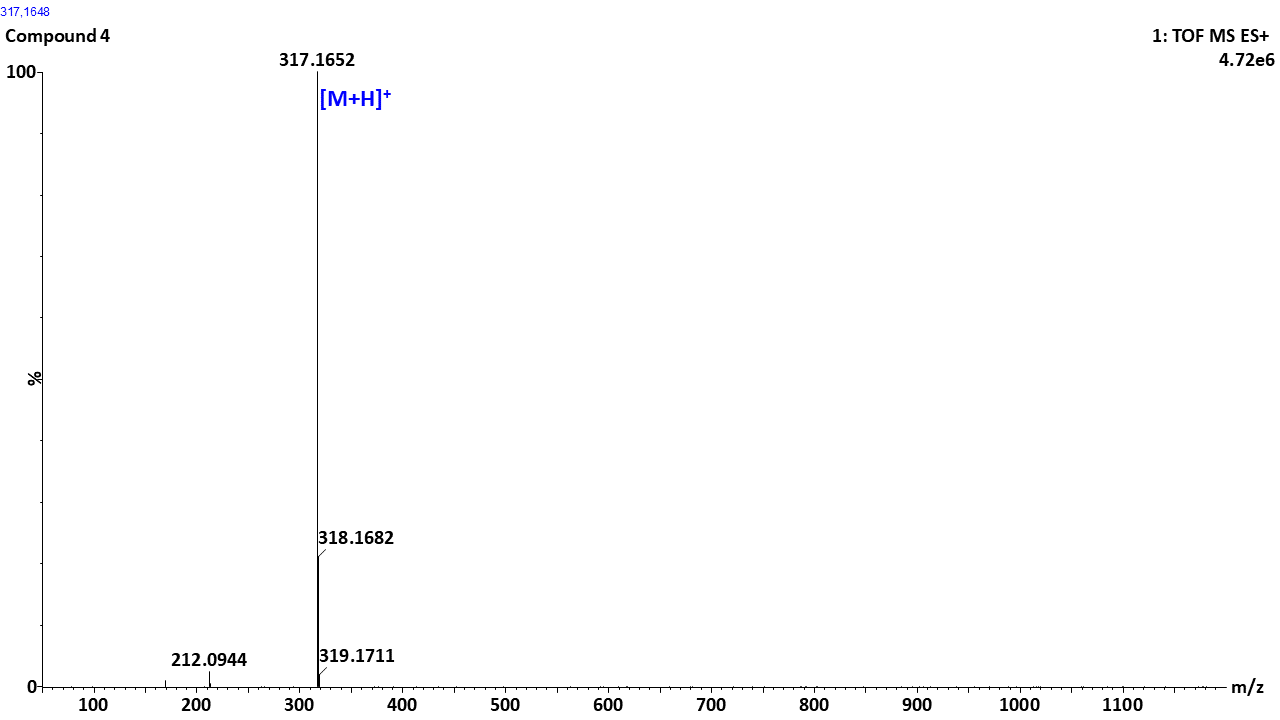


# HPLC-UV chromatogram of 7‐methoxy‐1‐methyl‐9‐[(4‐methylphenyl)methyl]‐9*H*‐pyrido[3,4‐*b*]indole (4):

# ^1^H NMR spectrum of 9‐[(2,4‐dimethylphenyl)methyl]‐7‐methoxy‐1‐methyl‐9*H*‐pyrido[3,4‐*b*]indole (5):


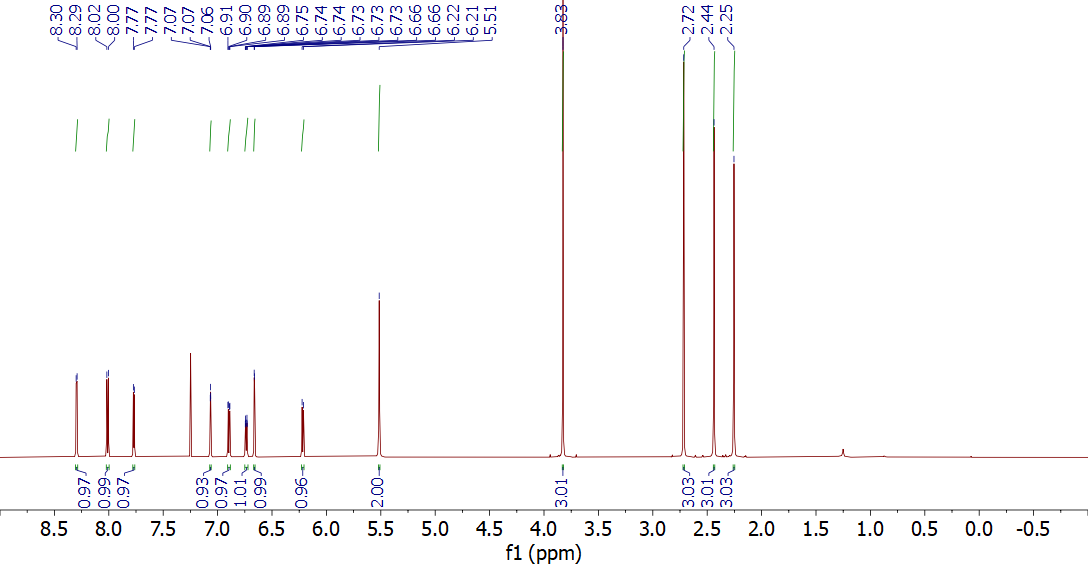


# ^13^C NMR spectrum of 9‐[(2,4‐dimethylphenyl)methyl]‐7‐methoxy‐1‐methyl‐9*H*‐pyrido[3,4‐*b*]indole (5):


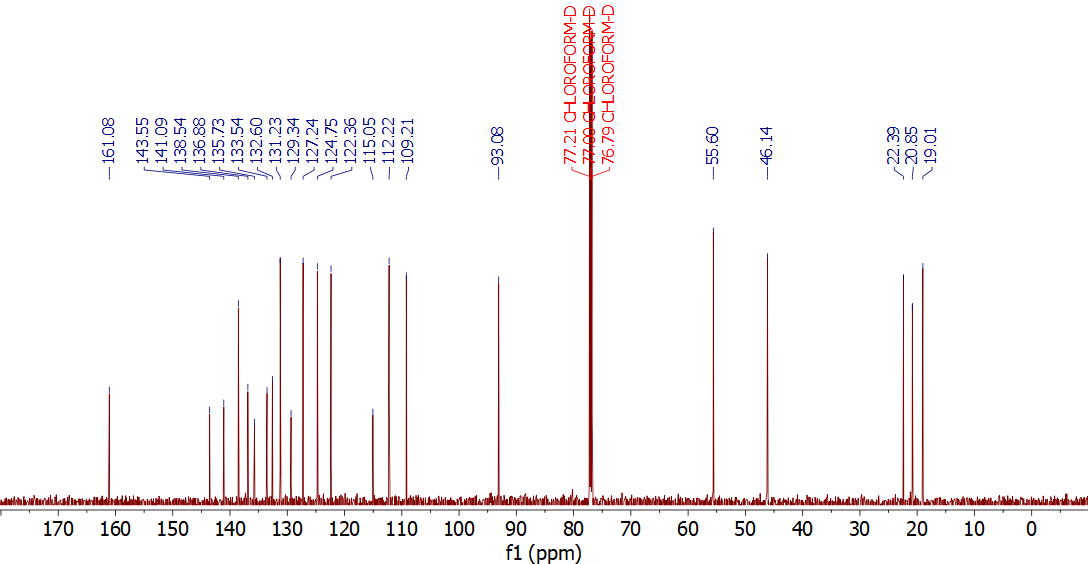


# ESI-HRMS spectrum of 9‐[(2,4‐dimethylphenyl)methyl]‐7‐methoxy‐1‐methyl‐9*H*‐pyrido[3,4‐*b*]indole (5):


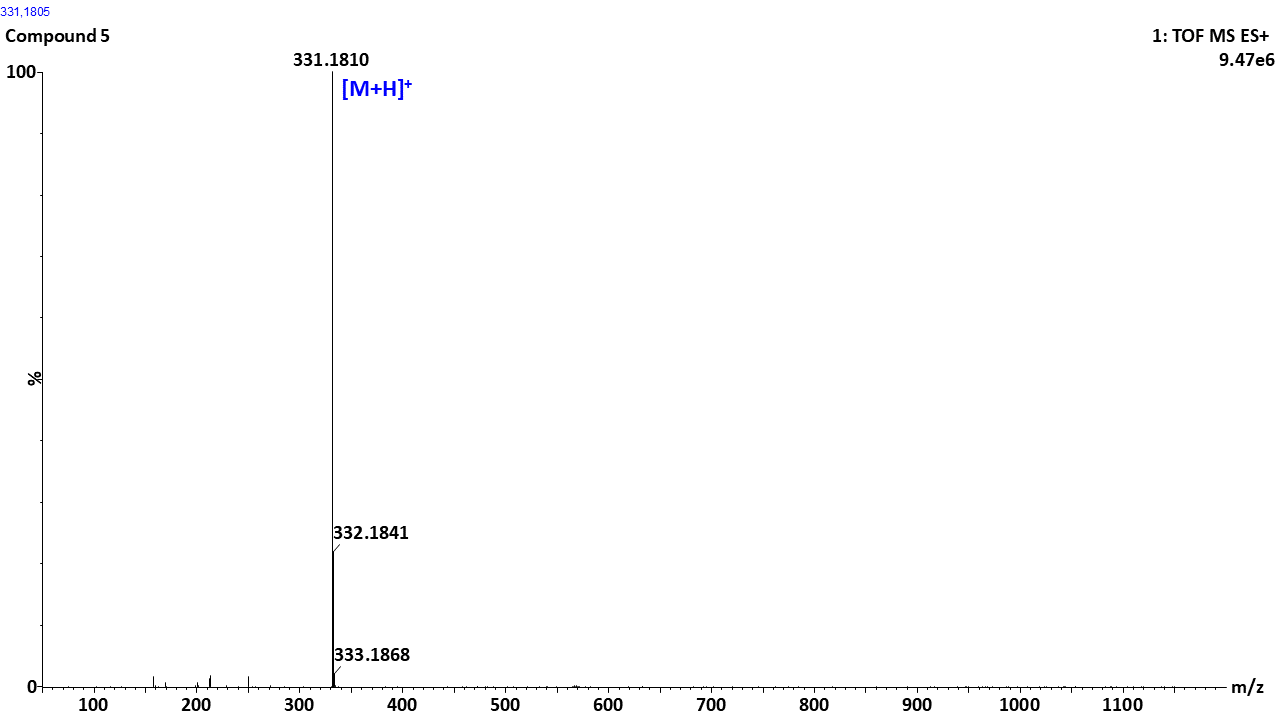


# HPLC-UV chromatogram of 9‐[(2,4‐dimethylphenyl)methyl]‐7‐methoxy‐1‐methyl‐9*H*‐pyrido[3,4‐*b*]indole (5):

# ^1^H NMR spectrum of 9‐[(3,5‐dimethylphenyl)methyl]‐7‐methoxy‐1‐methyl‐3*H*,4*H*,9*H*‐pyrido[3,4‐*b*]indole (6):

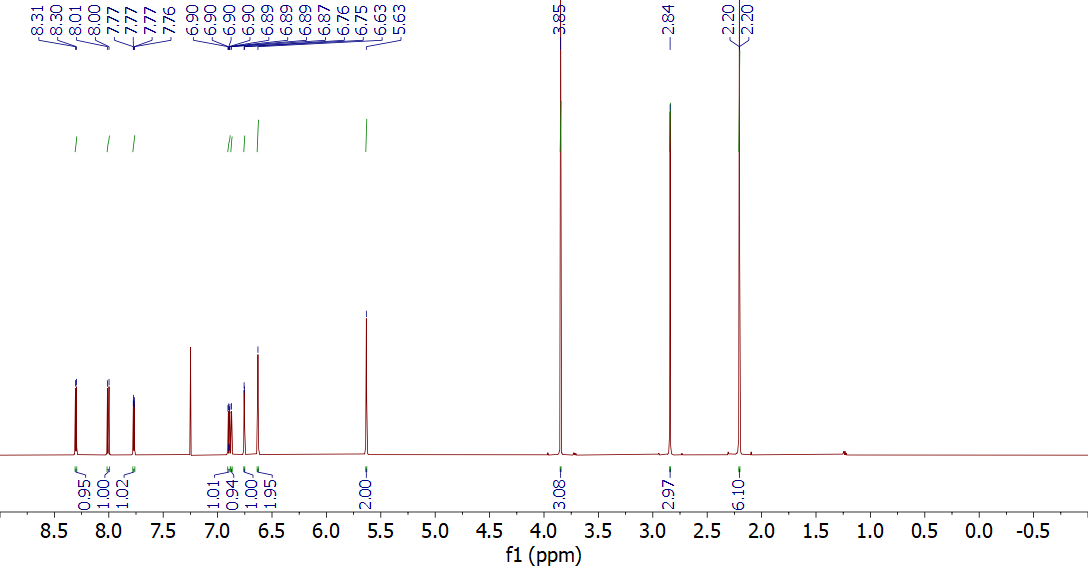


# ^13^C NMR spectrum of 9‐[(3,5‐dimethylphenyl)methyl]‐7‐methoxy‐1‐methyl‐3*H*,4*H*,9*H*‐pyrido[3,4‐*b*]indole (6):


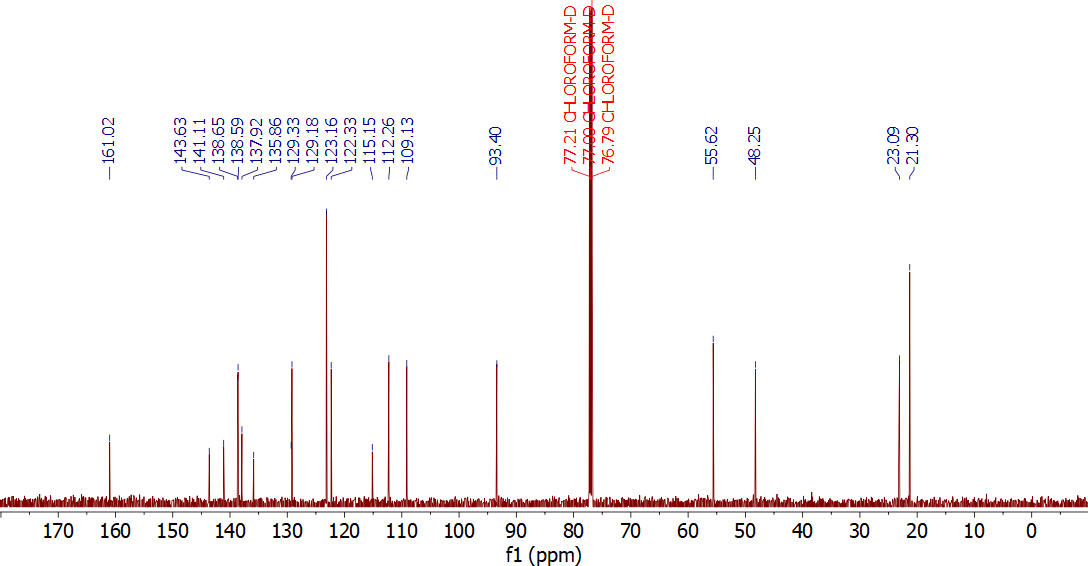


# ESI-HRMS spectrum of 9‐[(3,5‐dimethylphenyl)methyl]‐7‐methoxy‐1‐methyl‐3*H*,4*H*,9*H*‐pyrido[3,4‐*b*]indole (6):


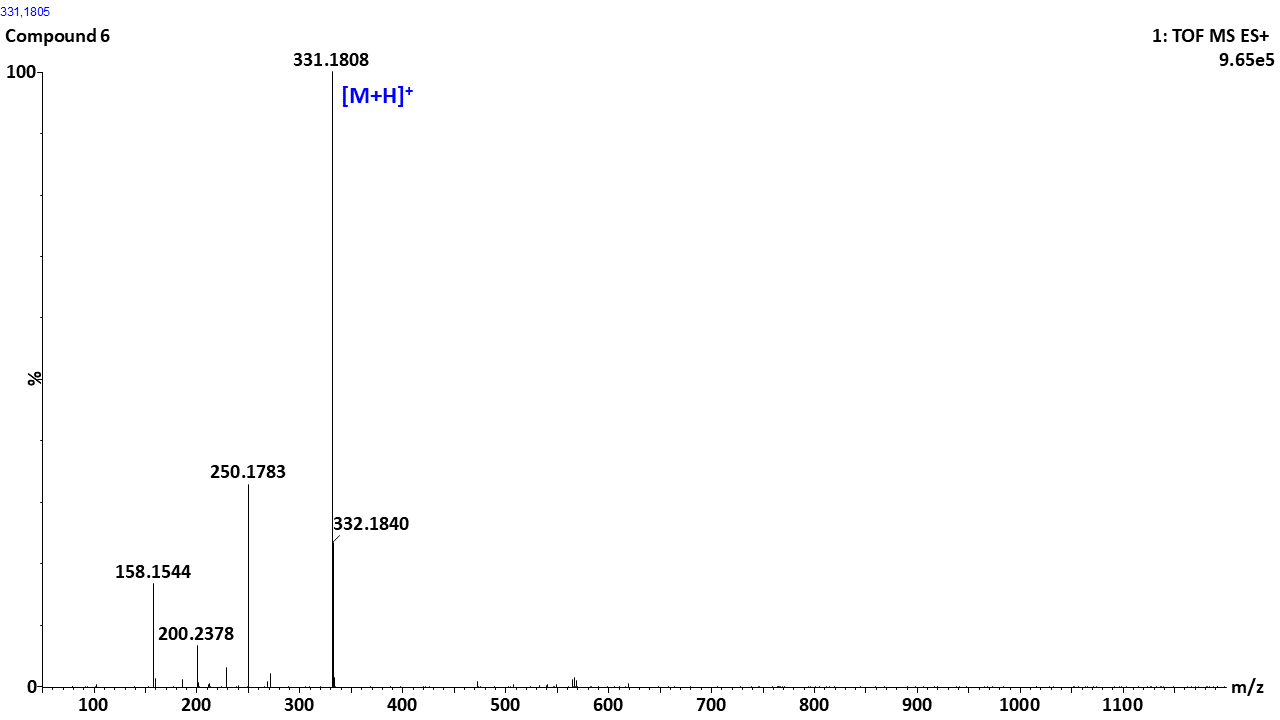


# HPLC-UV chromatogram of 9‐[(3,5‐dimethylphenyl)methyl]‐7‐methoxy‐1‐methyl‐3*H*,4*H*,9*H*‐pyrido[3,4‐*b*]indole (6):

# ^1^H NMR spectrum of 7‐methoxy‐1‐methyl‐9‐{[4‐(propan‐2‐yl)phenyl]methyl}‐9*H*‐pyrido[3,4‐*b*]indole (7):


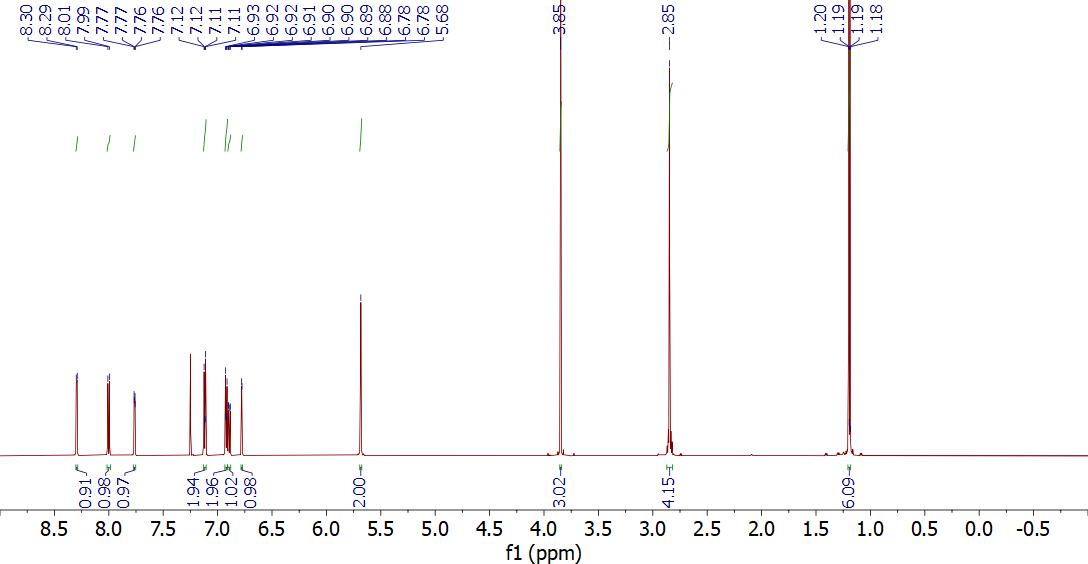


# ^13^C NMR spectrum of 7‐methoxy‐1‐methyl‐9‐{[4‐(propan‐2‐yl)phenyl]methyl}‐9*H*‐pyrido[3,4‐*b*]indole (7):


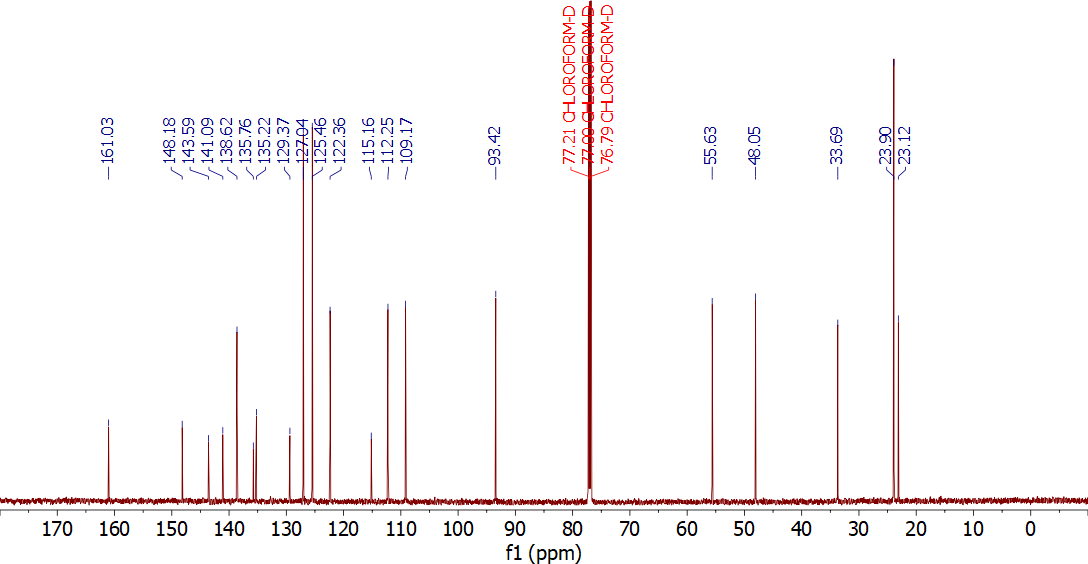


# ESI-HRMS spectrum of 7‐methoxy‐1‐methyl‐9‐{[4‐(propan‐2‐yl)phenyl]methyl}‐9*H*‐pyrido[3,4‐*b*]indole (7):


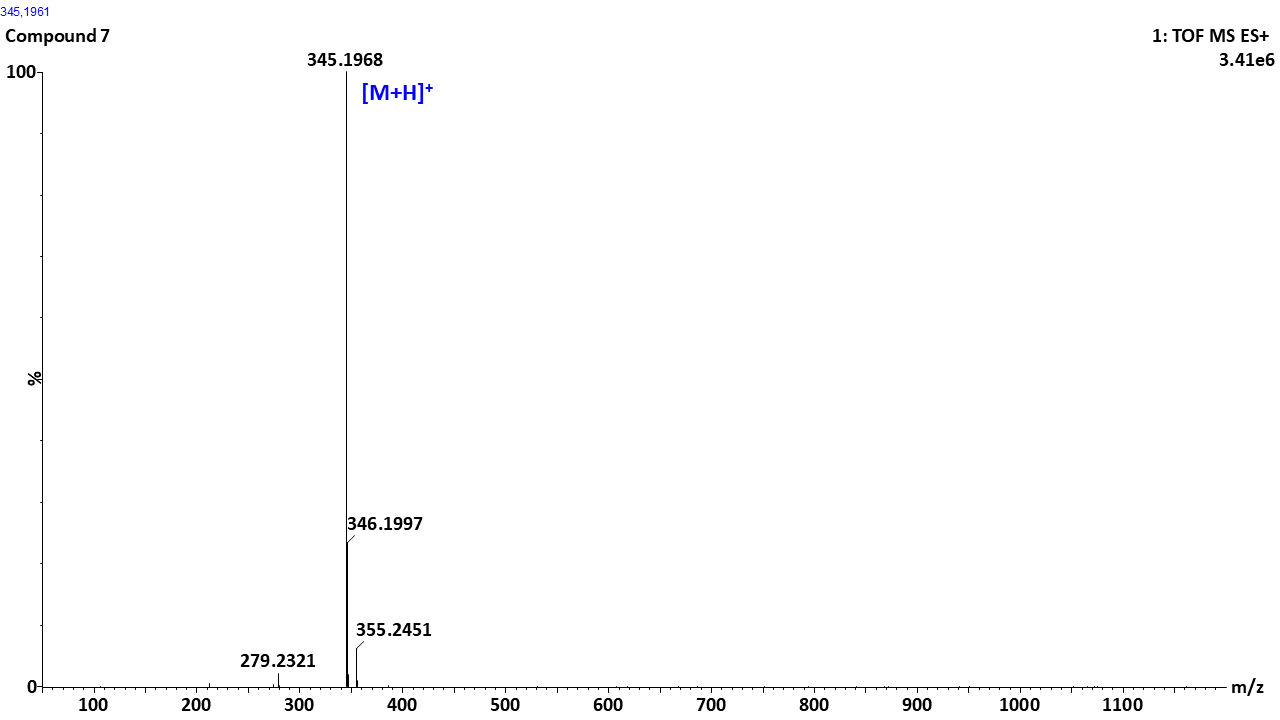


# HPLC-UV chromatogram of 7‐methoxy‐1‐methyl‐9‐{[4‐(propan‐2‐yl)phenyl]methyl}‐9*H*‐pyrido[3,4‐*b*]indole (7):

# ^1^H NMR spectrum of 9‐[(4‐tert‐butylphenyl)methyl]‐7‐methoxy‐1‐methyl‐9*H*‐pyrido[3,4‐*b*]indole (8):


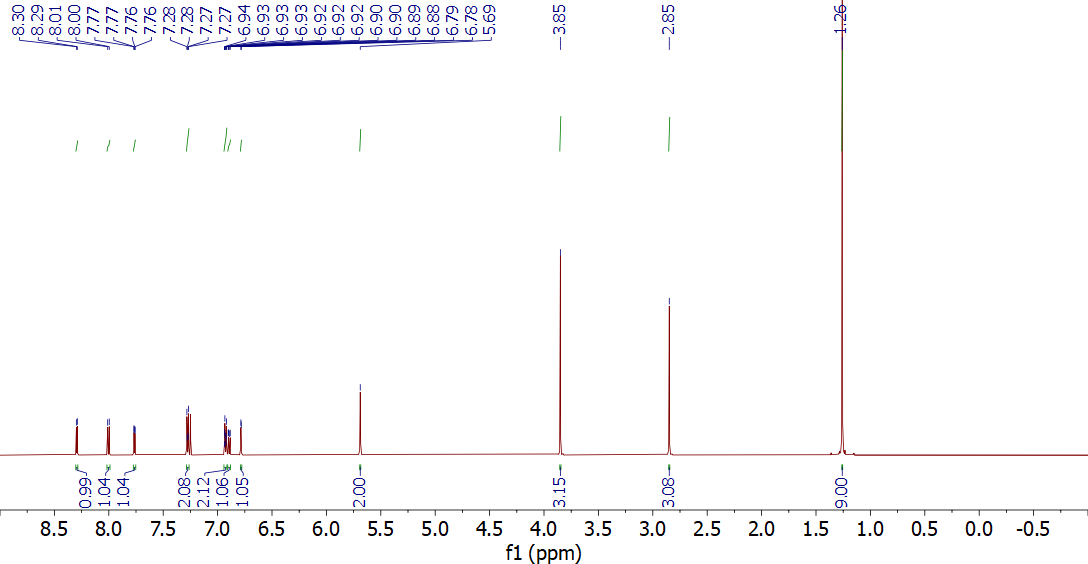


# ^13^C NMR spectrum of 9‐[(4‐tert‐butylphenyl)methyl]‐7‐methoxy‐1‐methyl‐9*H*‐pyrido[3,4‐*b*]indole (8):


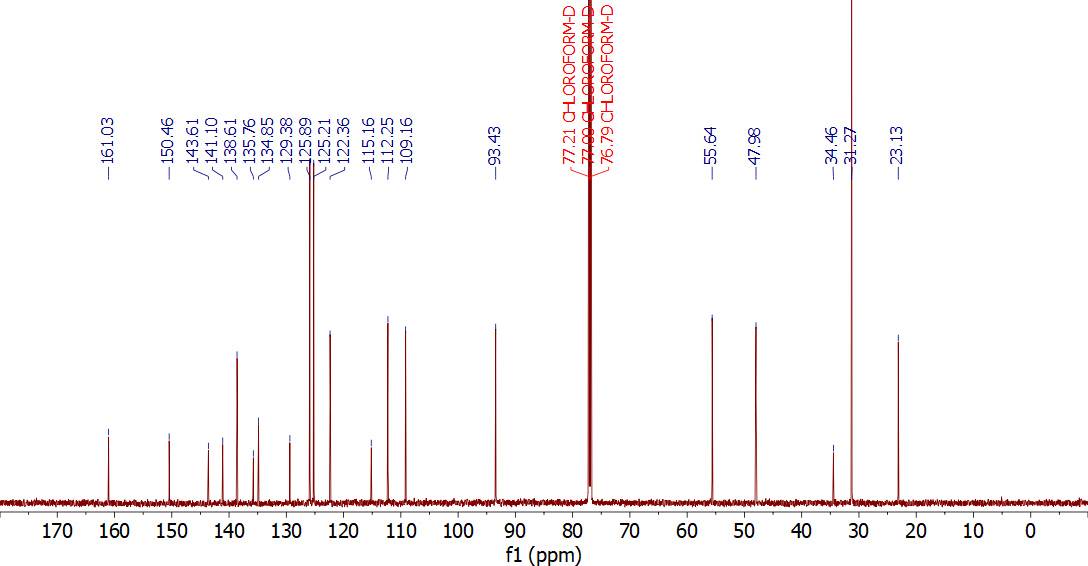


# ESI-HRMS spectrum of 9‐[(4‐tert‐butylphenyl)methyl]‐7‐methoxy‐1‐methyl‐9*H*‐pyrido[3,4‐*b*]indole (8):


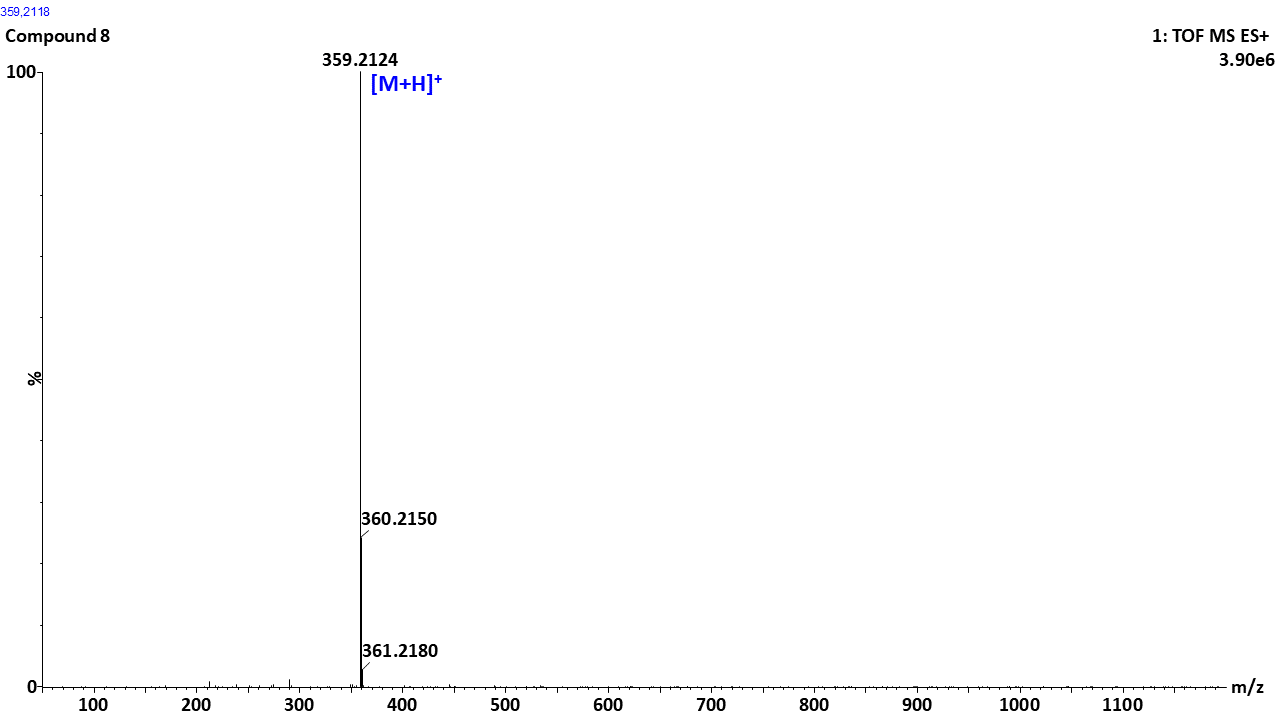


# HPLC-UV chromatogram of 9‐[(4‐tert‐butylphenyl)methyl]‐7‐methoxy‐1‐methyl‐9*H*‐pyrido[3,4‐*b*]indole (8):

# ^1^H NMR spectrum of 7‐methoxy‐9‐[(3‐methoxyphenyl)methyl]‐1‐methyl‐9*H*‐pyrido[3,4‐*b*]indole (9):


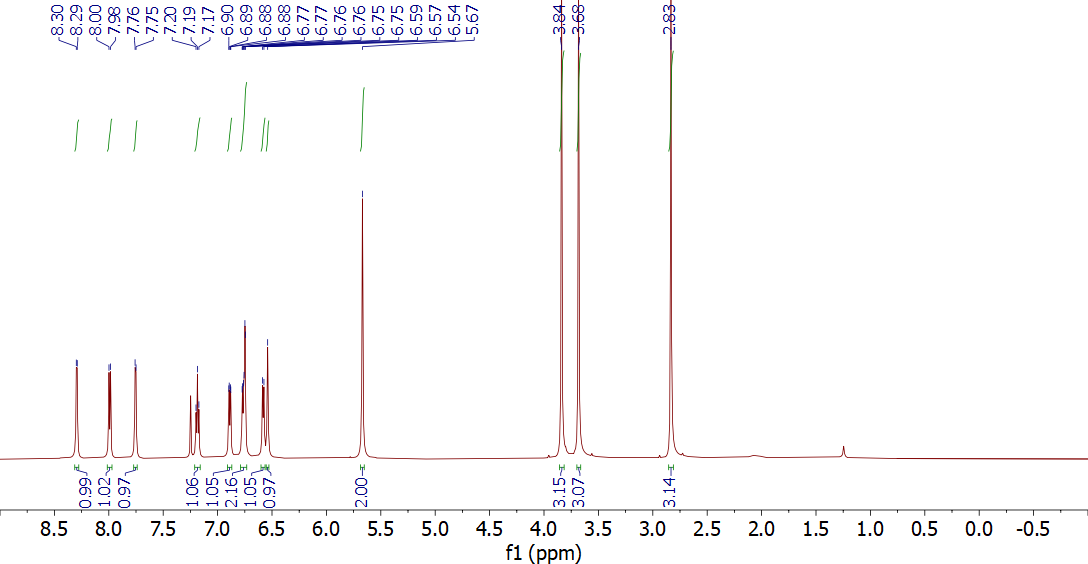


# ^13^C NMR spectrum of 7‐methoxy‐9‐[(3‐methoxyphenyl)methyl]‐1‐methyl‐9*H*‐pyrido[3,4‐*b*]indole (9):


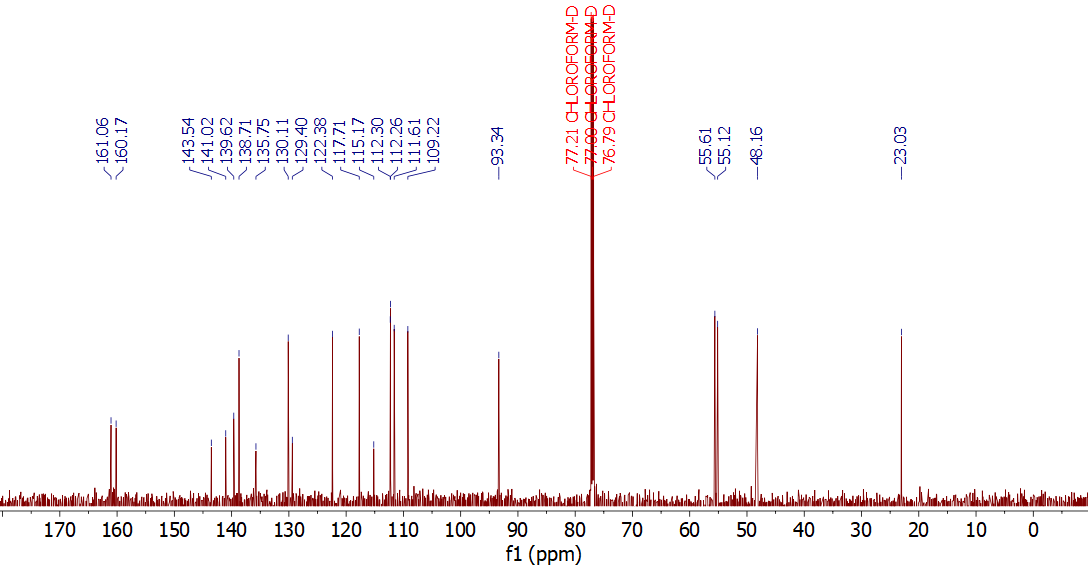


# ESI-HRMS spectrum of 7‐methoxy‐9‐[(3‐methoxyphenyl)methyl]‐1‐methyl‐9*H*‐pyrido[3,4‐*b*]indole (9):


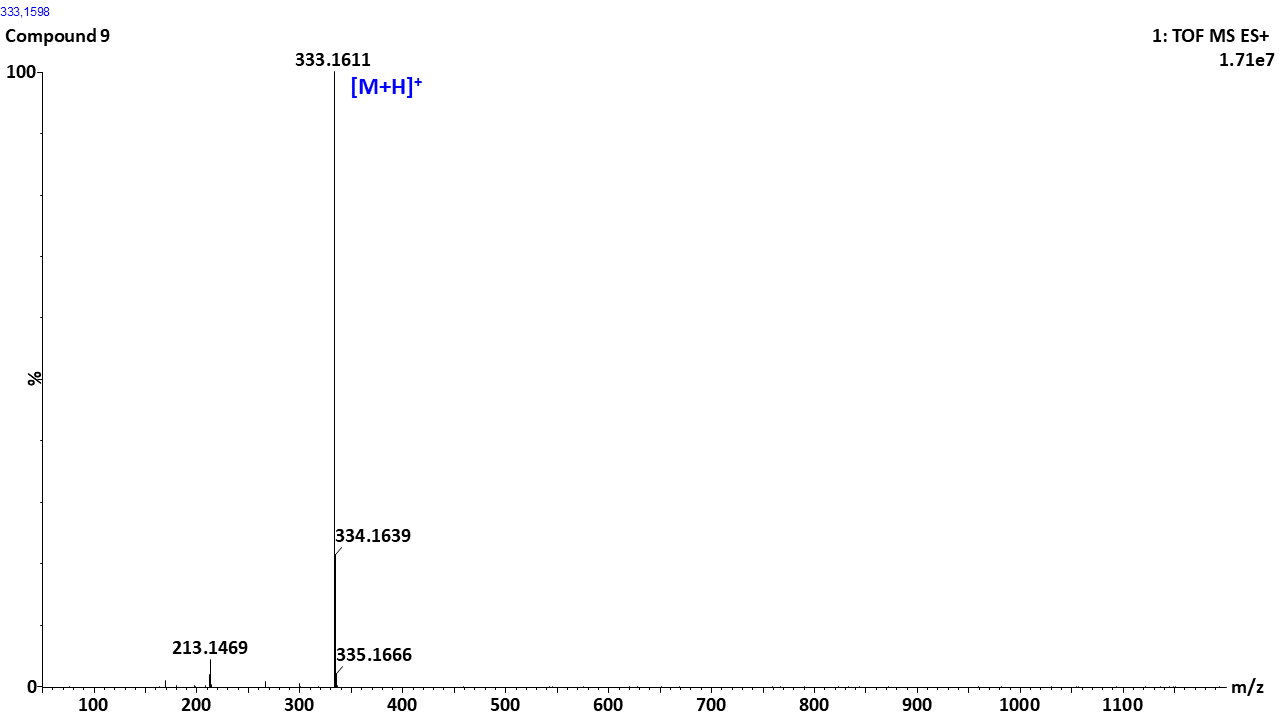


# HPLC-UV chromatogram of 7‐methoxy‐9‐[(3‐methoxyphenyl)methyl]‐1‐methyl‐9*H*‐pyrido[3,4‐*b*]indole (9):

# ^1^H NMR spectrum of 9‐[(3,5‐dimethoxyphenyl)methyl]‐7‐methoxy‐1‐methyl‐9*H*‐pyrido[3,4‐*b*]indole (10):


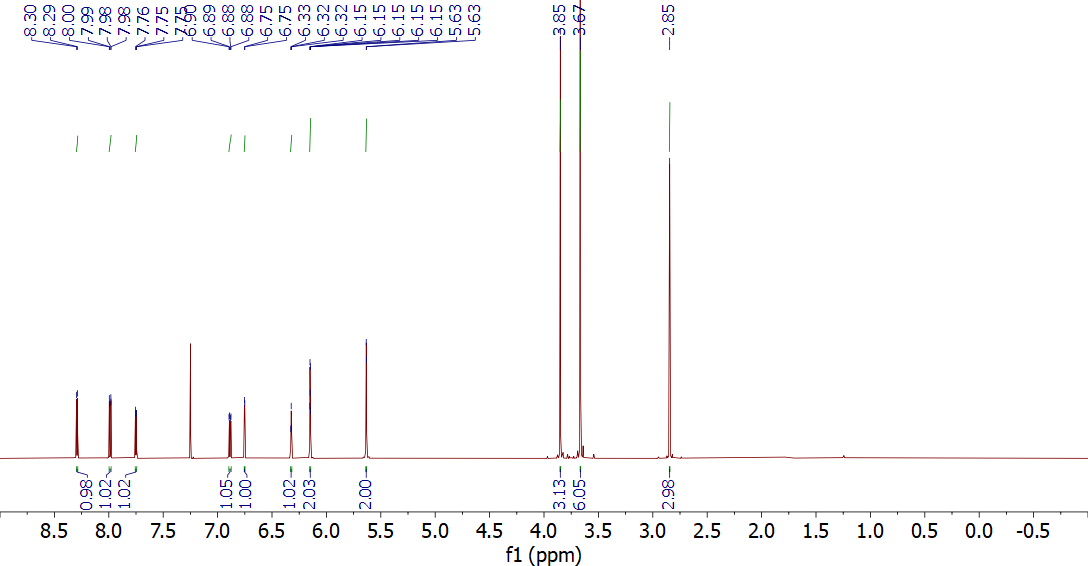


# ^13^C NMR spectrum of 9‐[(3,5‐dimethoxyphenyl)methyl]‐7‐methoxy‐1‐methyl‐9*H*‐pyrido[3,4‐*b*]indole (10):


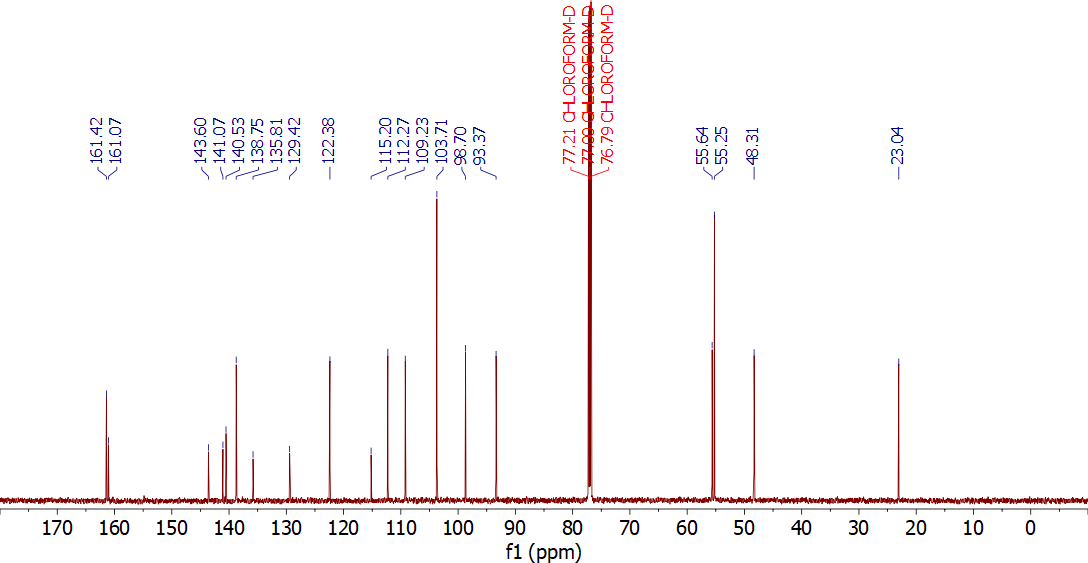


# ESI-HRMS spectrum of 9‐[(3,5‐dimethoxyphenyl)methyl]‐7‐methoxy‐1‐methyl‐9*H*‐pyrido[3,4‐*b*]indole (10):


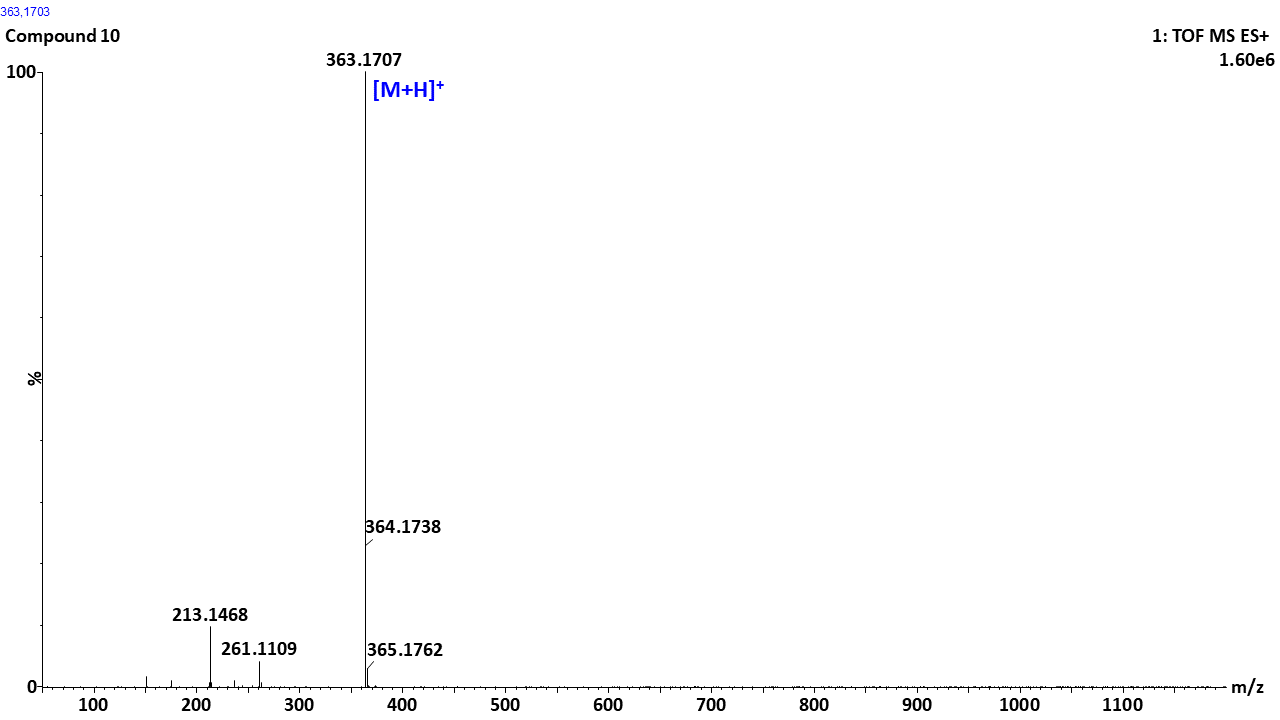


# HPLC-UV chromatogram of 9‐[(3,5‐dimethoxyphenyl)methyl]‐7‐methoxy‐1‐methyl‐9*H*‐pyrido[3,4‐*b*]indole (10):

# ^1^H NMR spectrum of 9‐[(2‐fluorophenyl)methyl]‐7‐methoxy‐1‐methyl‐9*H*‐pyrido[3,4‐*b*]indole (11):

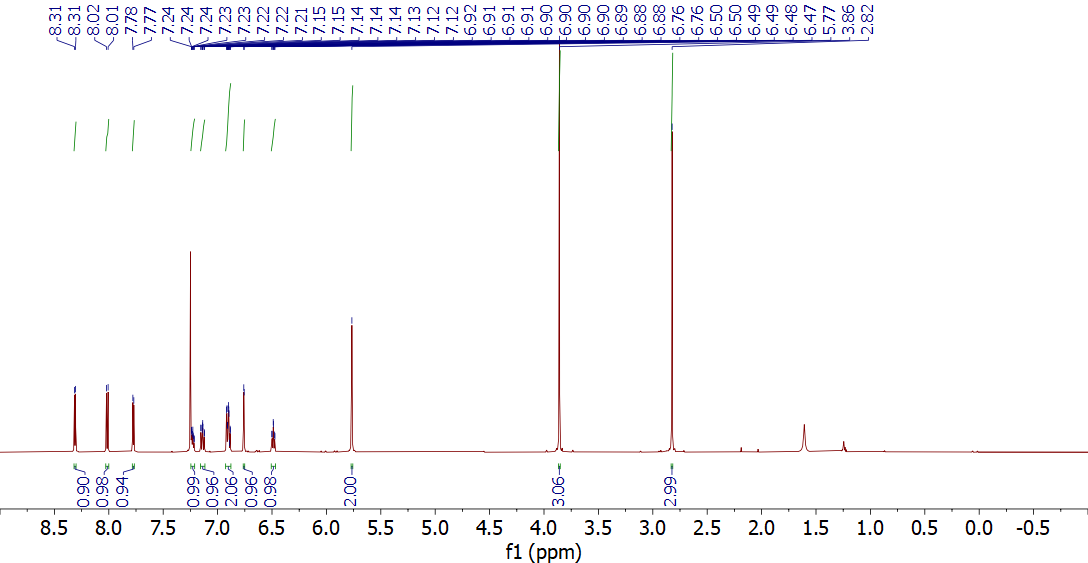


# ^13^C NMR spectrum of 9‐[(2‐fluorophenyl)methyl]‐7‐methoxy‐1‐methyl‐9*H*‐pyrido[3,4‐*b*]indole (11):


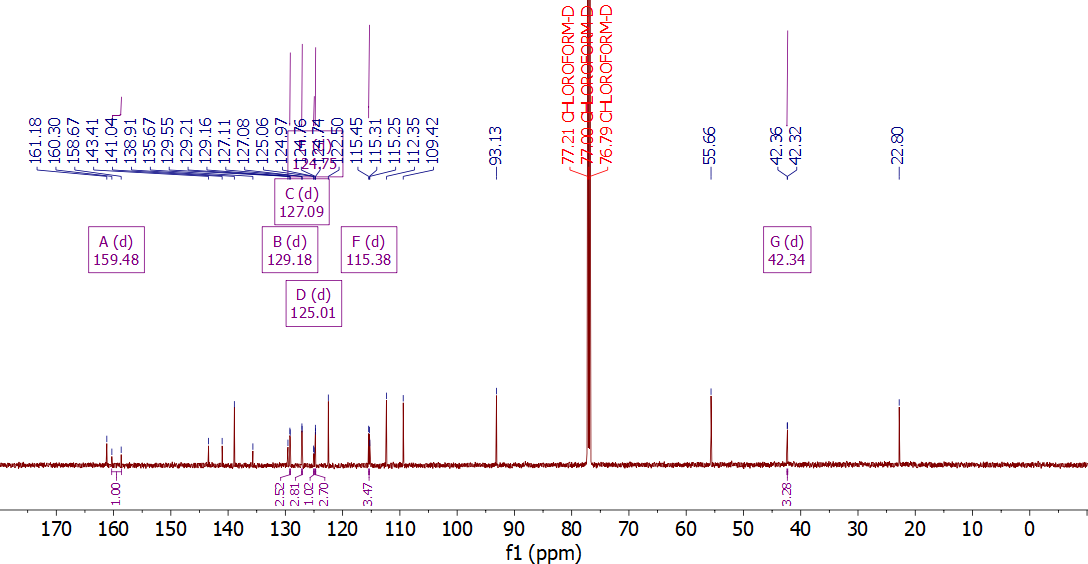


# ESI-HRMS spectrum of 9‐[(2‐fluorophenyl)methyl]‐7‐methoxy‐1‐methyl‐9*H*‐pyrido[3,4‐*b*]indole (11):


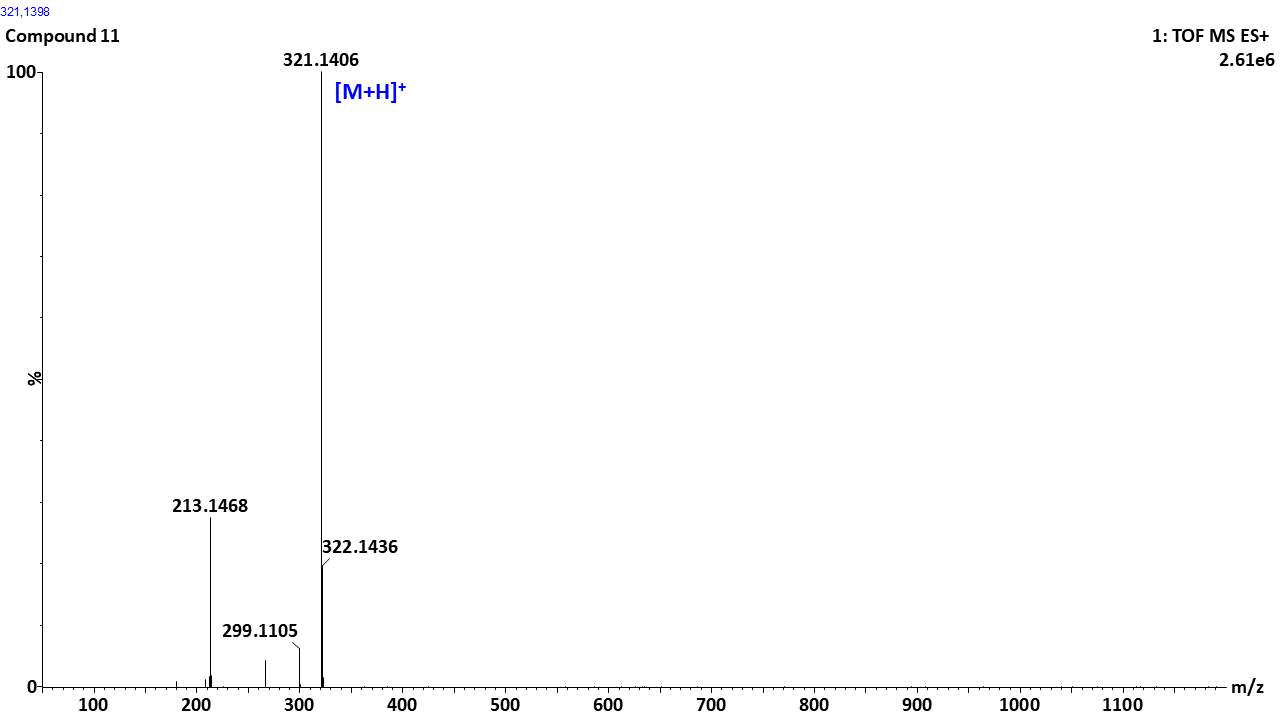


# HPLC-UV chromatogram of 9‐[(2‐fluorophenyl)methyl]‐7‐methoxy‐1‐methyl‐9*H*‐pyrido[3,4‐*b*]indole (11):

# ^1^H NMR spectrum of 9‐[(3‐fluorophenyl)methyl]‐7‐methoxy‐1‐methyl‐9*H*‐pyrido[3,4‐*b*]indole (12):


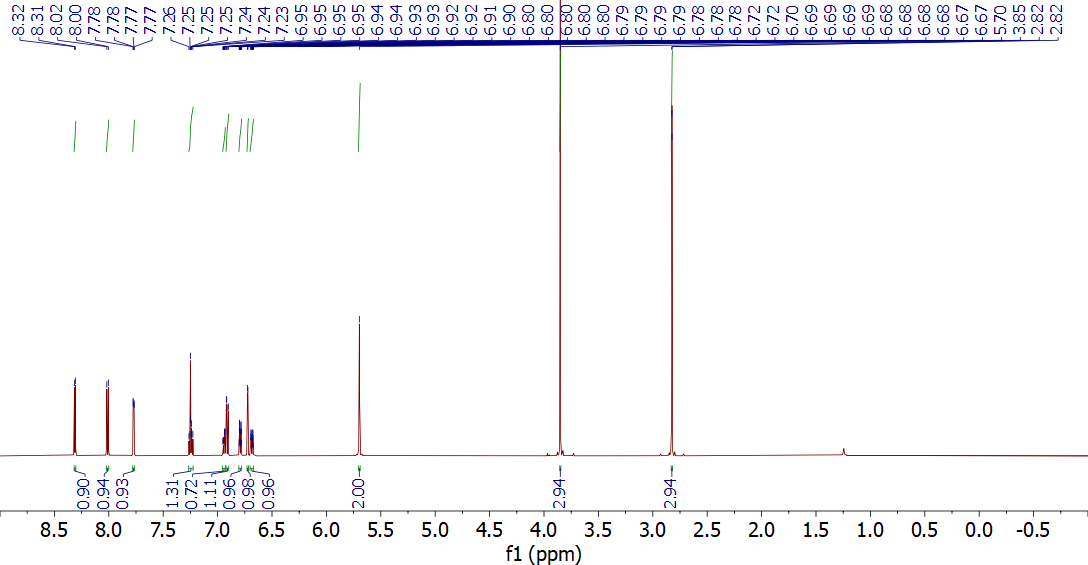


# ^13^C NMR spectrum of 9‐[(3‐fluorophenyl)methyl]‐7‐methoxy‐1‐methyl‐9*H*‐pyrido[3,4‐*b*]indole (12):


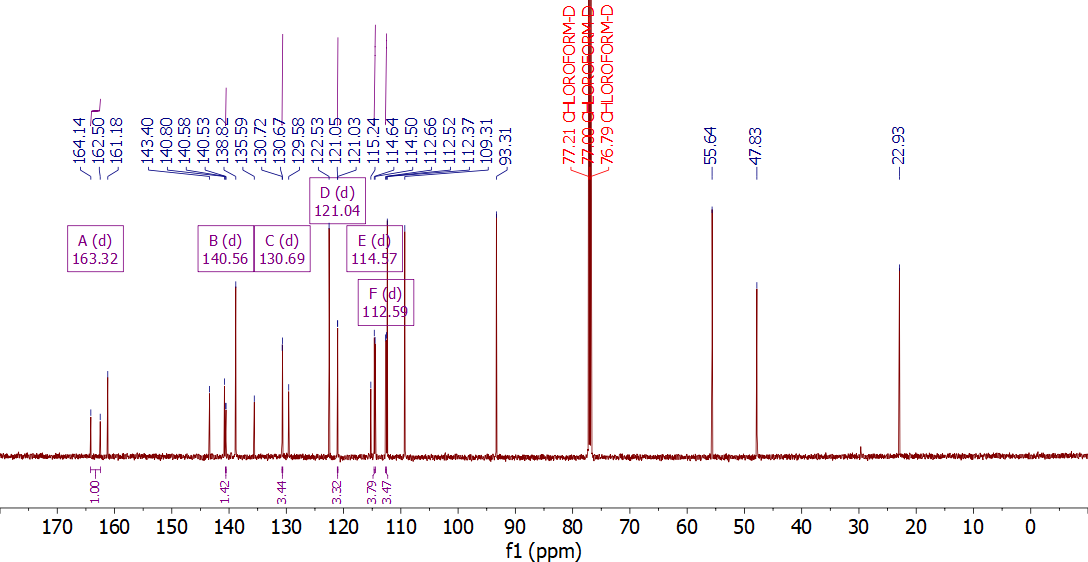


# ESI-HRMS spectrum of 9‐[(3‐fluorophenyl)methyl]‐7‐methoxy‐1‐methyl‐9*H*‐pyrido[3,4‐*b*]indole (12):


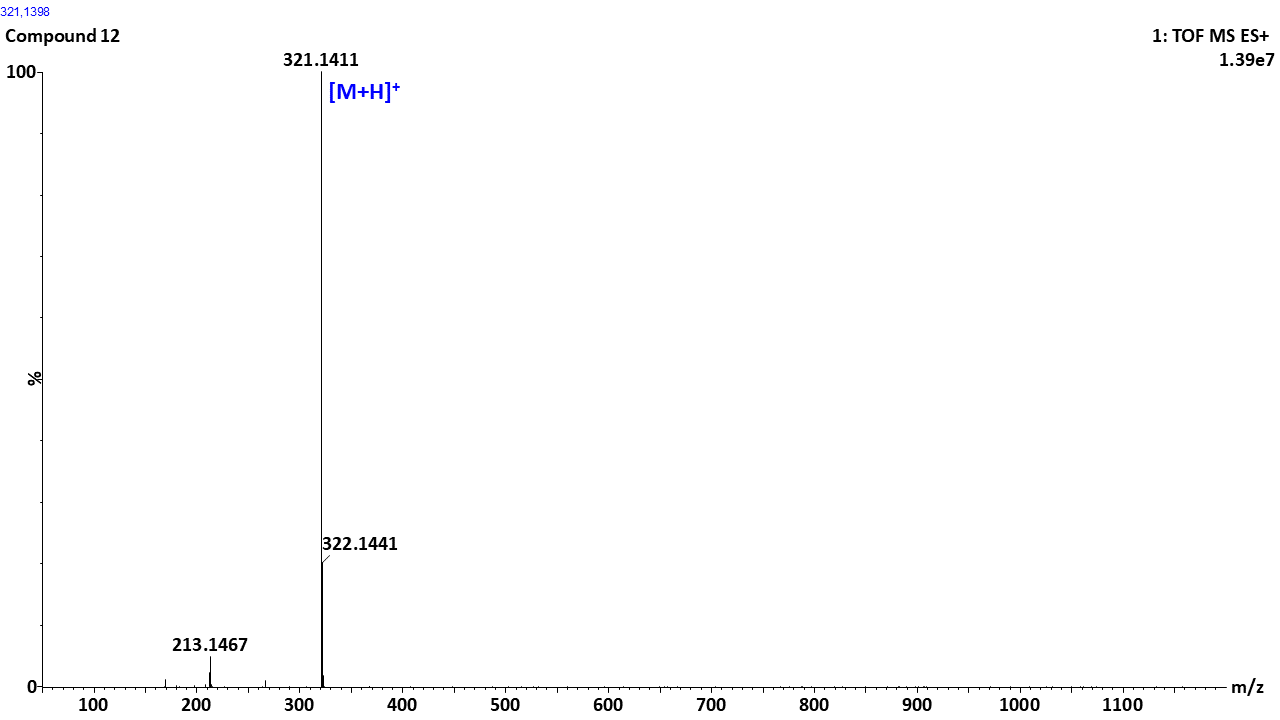


# HPLC-UV chromatogram of 9‐[(3‐fluorophenyl)methyl]‐7‐methoxy‐1‐methyl‐9*H*‐pyrido[3,4‐*b*]indole (12):

# ^1^H NMR spectrum of 9‐[(4‐fluorophenyl)methyl]‐7‐methoxy‐1‐methyl‐9*H*‐pyrido[3,4‐*b*]indole (13):

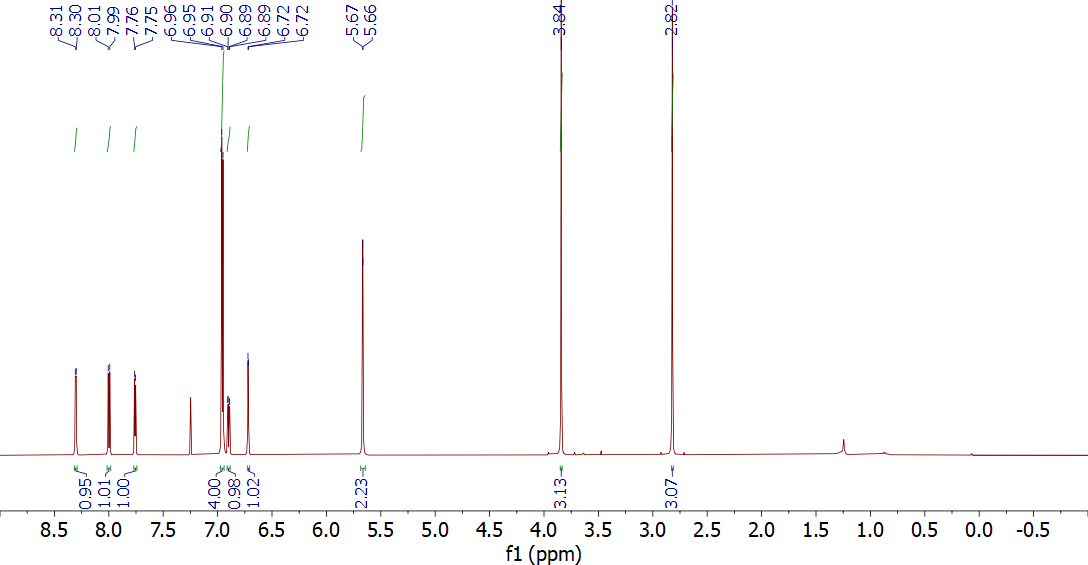


# ^13^C NMR spectrum of 9‐[(4‐fluorophenyl)methyl]‐7‐methoxy‐1‐methyl‐9*H*‐pyrido[3,4‐*b*]indole (13):


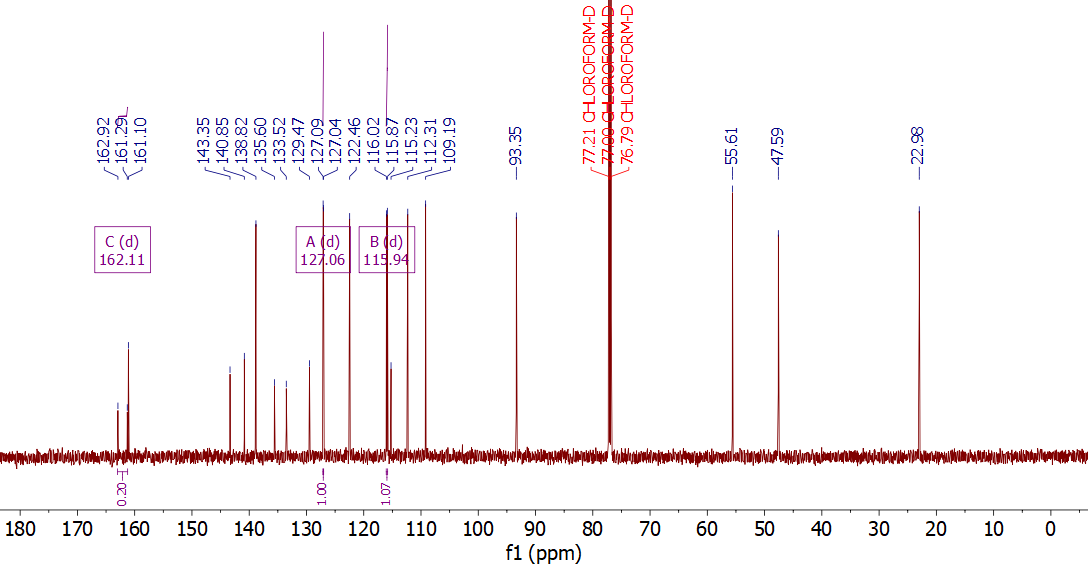


# ESI-HRMS spectrum of 9‐[(4‐fluorophenyl)methyl]‐7‐methoxy‐1‐methyl‐9*H*‐pyrido[3,4‐*b*]indole (13):


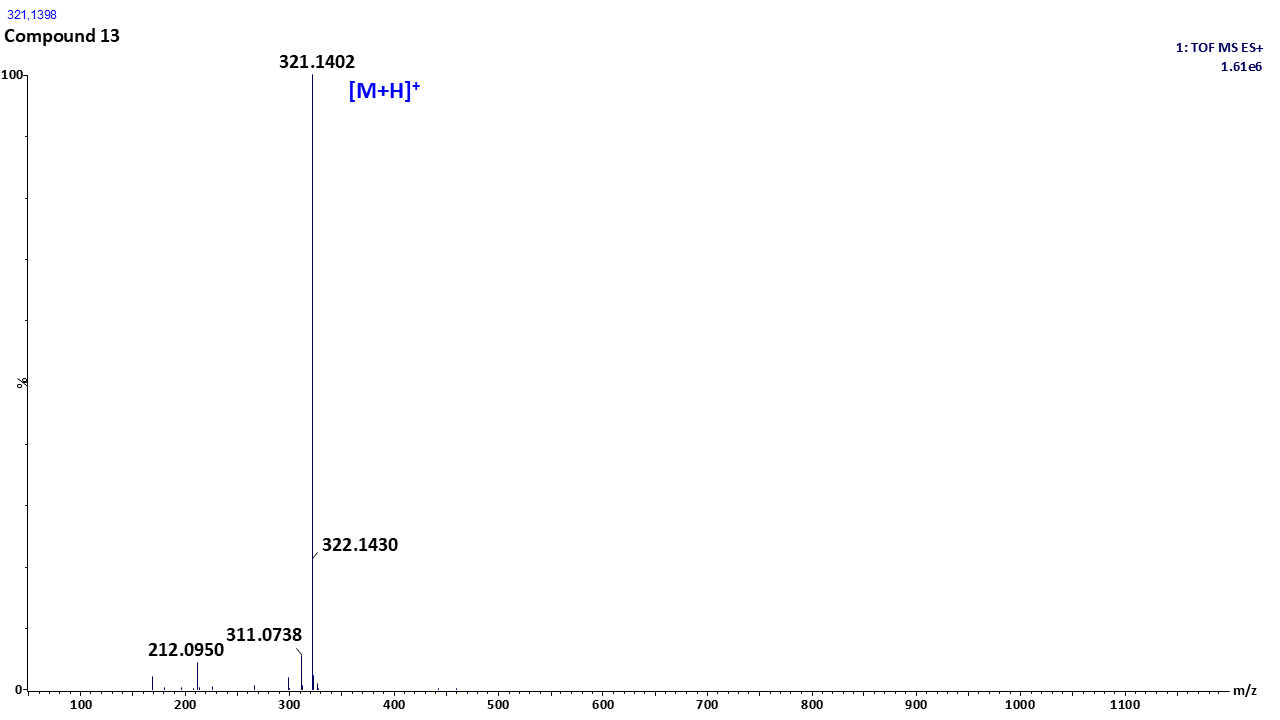


# HPLC-UV chromatogram of 9‐[(4‐fluorophenyl)methyl]‐7‐methoxy‐1‐methyl‐9*H*‐pyrido[3,4‐*b*]indole (13):

# ^1^H NMR spectrum of 9‐[(2‐chlorophenyl)methyl]‐7‐methoxy‐1‐methyl‐9*H*‐pyrido[3,4‐*b*]indole (14):


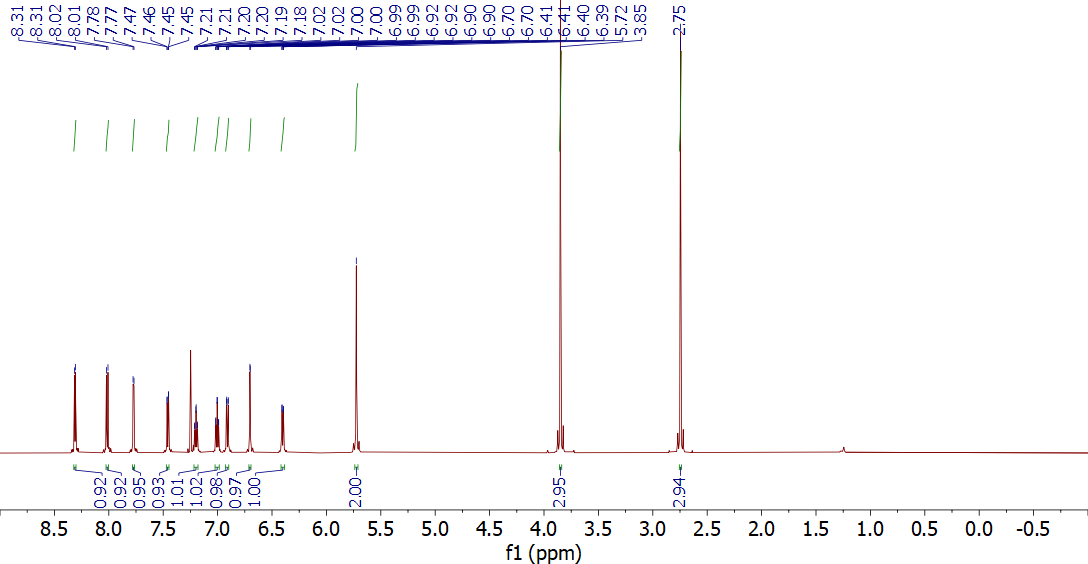


# ^13^C NMR spectrum of 9‐[(2‐chlorophenyl)methyl]‐7‐methoxy‐1‐methyl‐9*H*‐pyrido[3,4‐*b*]indole (14):


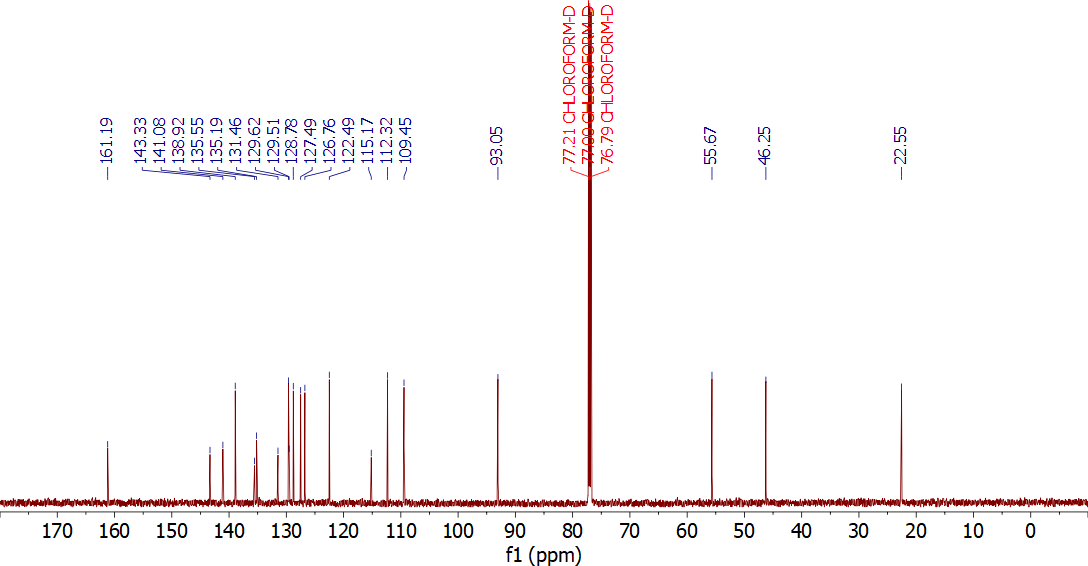


# ESI-HRMS spectrum of 9‐[(2‐chlorophenyl)methyl]‐7‐methoxy‐1‐methyl‐9*H*‐pyrido[3,4‐*b*]indole (14):


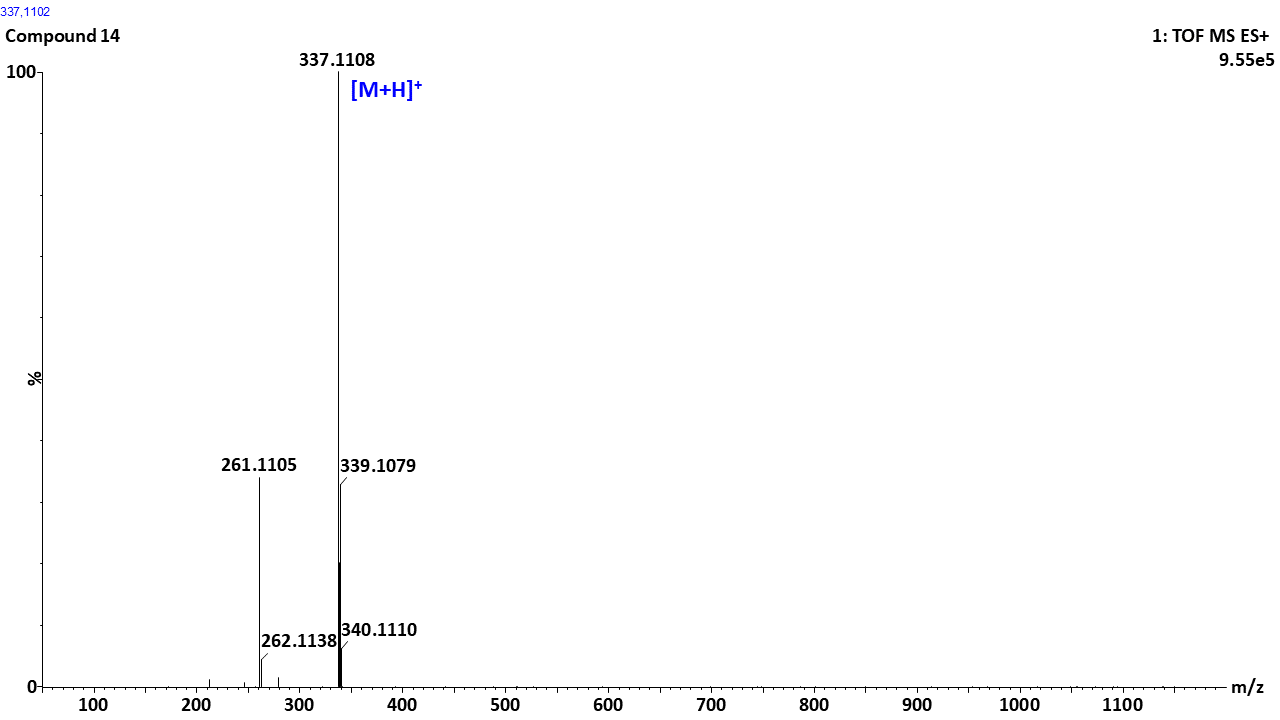


# HPLC-UV chromatogram of 9‐[(2‐chlorophenyl)methyl]‐7‐methoxy‐1‐methyl‐9*H*‐pyrido[3,4‐*b*]indole (14):

# ^1^H NMR spectrum of 9‐[(3‐chlorophenyl)methyl]‐7‐methoxy‐1‐methyl‐9*H*‐pyrido[3,4‐*b*]indole (15):

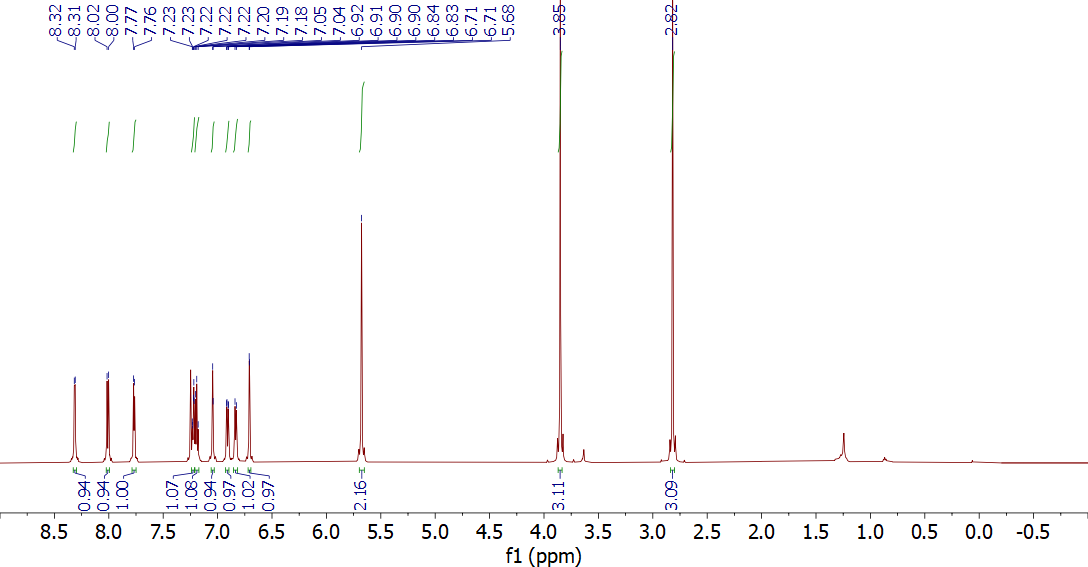


# ^13^C NMR spectrum of 9‐[(3‐chlorophenyl)methyl]‐7‐methoxy‐1‐methyl‐9*H*‐pyrido[3,4‐*b*]indole (15):


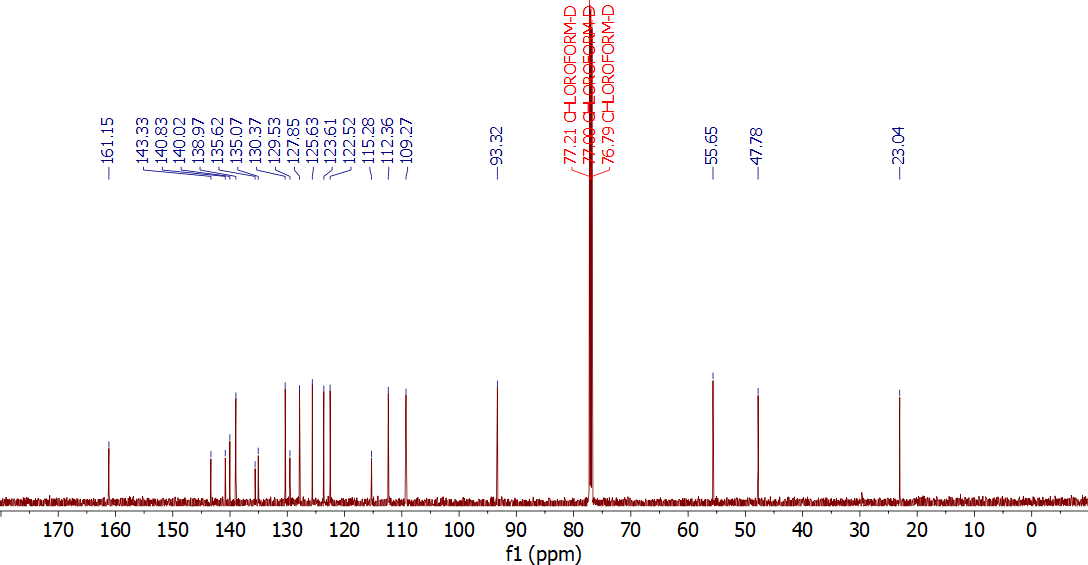


# ESI-HRMS spectrum of 9‐[(3‐chlorophenyl)methyl]‐7‐methoxy‐1‐methyl‐9*H*‐pyrido[3,4‐*b*]indole (15):


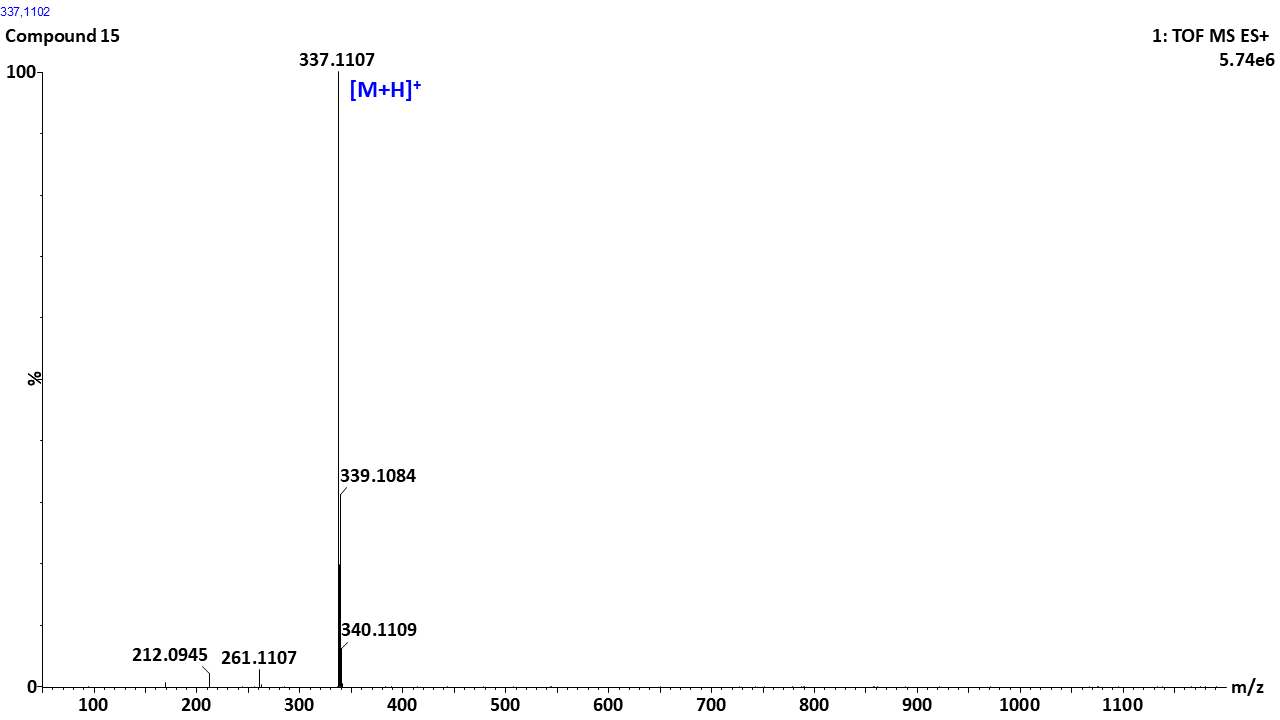


# HPLC-UV chromatogram of 9‐[(3‐chlorophenyl)methyl]‐7‐methoxy‐1‐methyl‐9*H*‐pyrido[3,4‐*b*]indole (15):

# ^1^H NMR spectrum of 9‐[(4‐chlorophenyl)methyl]‐7‐methoxy‐1‐methyl‐9*H*‐pyrido[3,4‐*b*]indole (16):


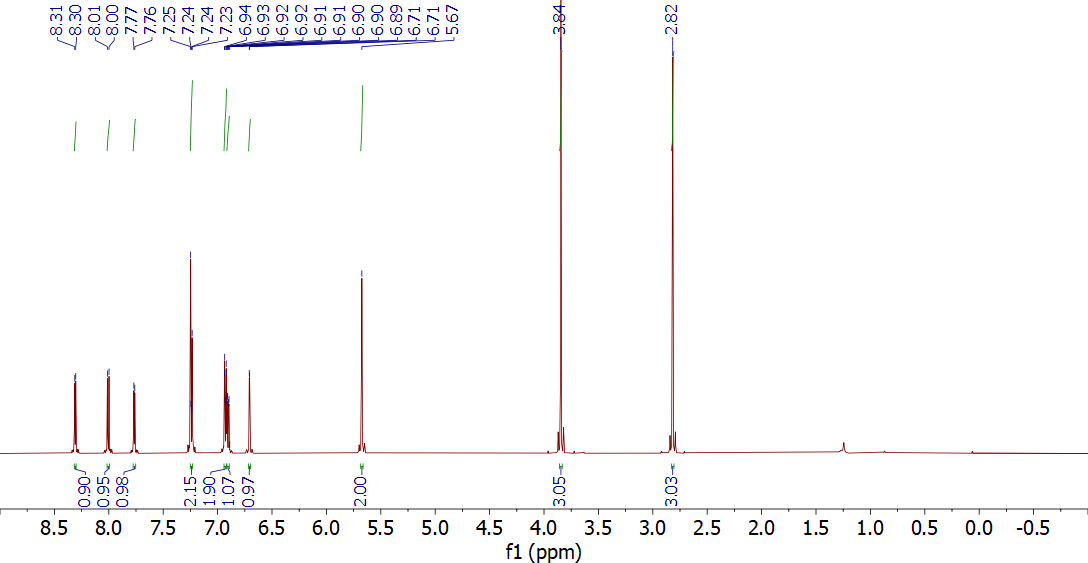


# ^13^C NMR spectrum of 9‐[(4‐chlorophenyl)methyl]‐7‐methoxy‐1‐methyl‐9*H*‐pyrido[3,4‐*b*]indole (16):


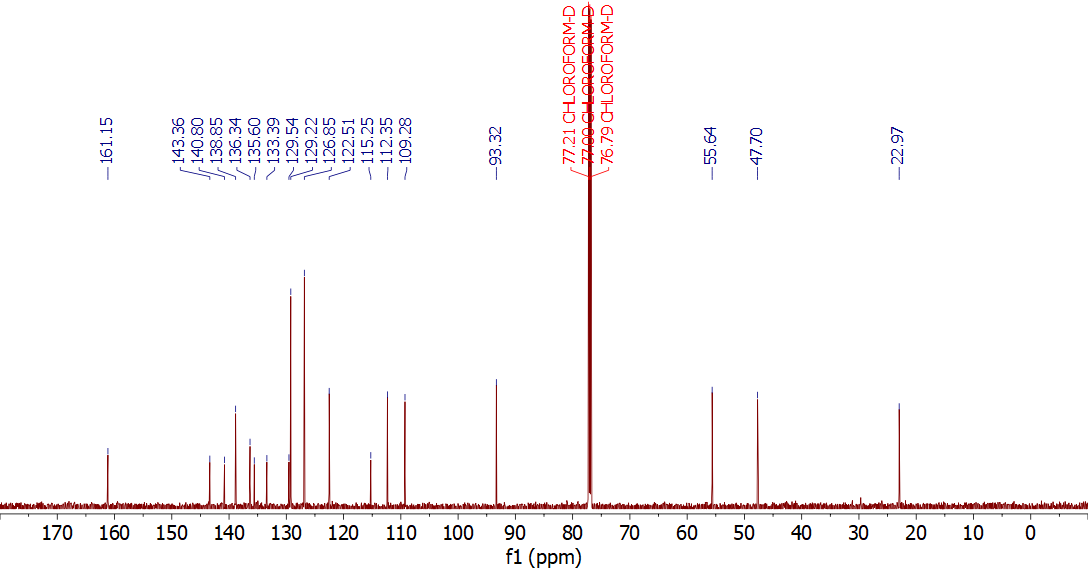


# ESI-HRMS spectrum of 9‐[(4‐chlorophenyl)methyl]‐7‐methoxy‐1‐methyl‐9*H*‐pyrido[3,4‐*b*]indole (16):


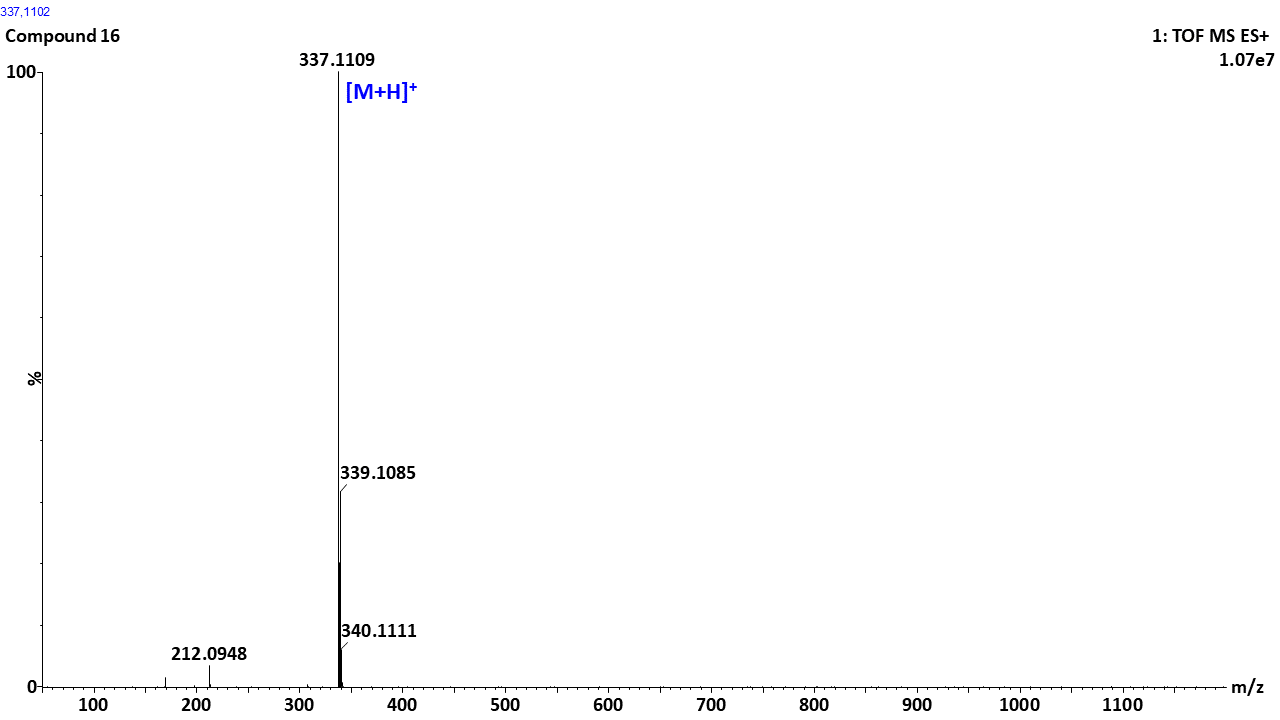


# HPLC-UV chromatogram of 9‐[(4‐chlorophenyl)methyl]‐7‐methoxy‐1‐methyl‐9*H*‐pyrido[3,4‐*b*]indole (16):

# ^1^H NMR spectrum of 9‐[(3,4‐dichlorophenyl)methyl]‐7‐methoxy‐1‐methyl‐9*H*‐pyrido[3,4‐*b*]indole (17):

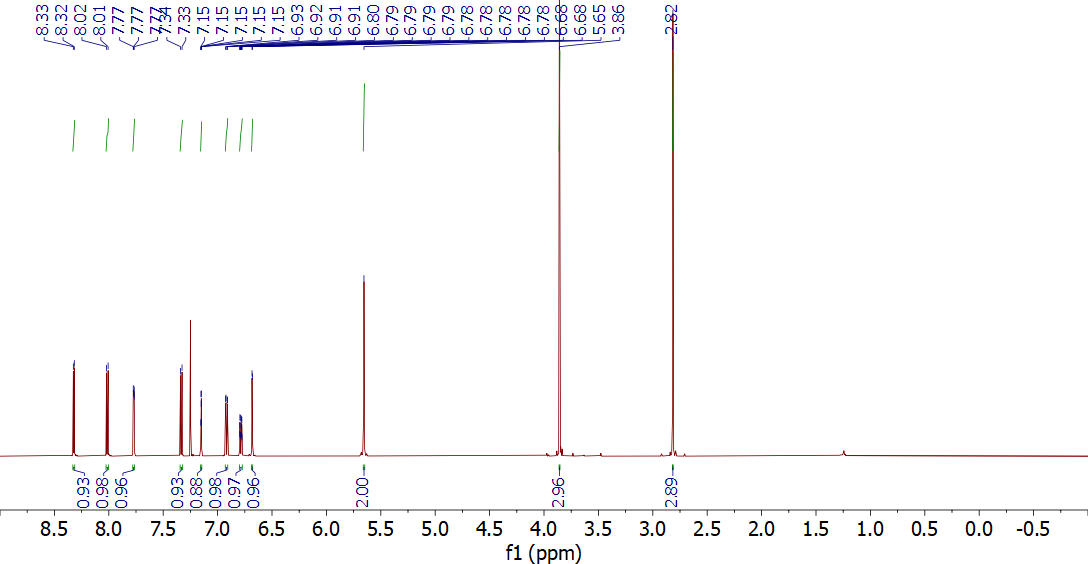


# ^13^C NMR spectrum of 9‐[(3,4‐dichlorophenyl)methyl]‐7‐methoxy‐1‐methyl‐9*H*‐pyrido[3,4‐*b*]indole (17):


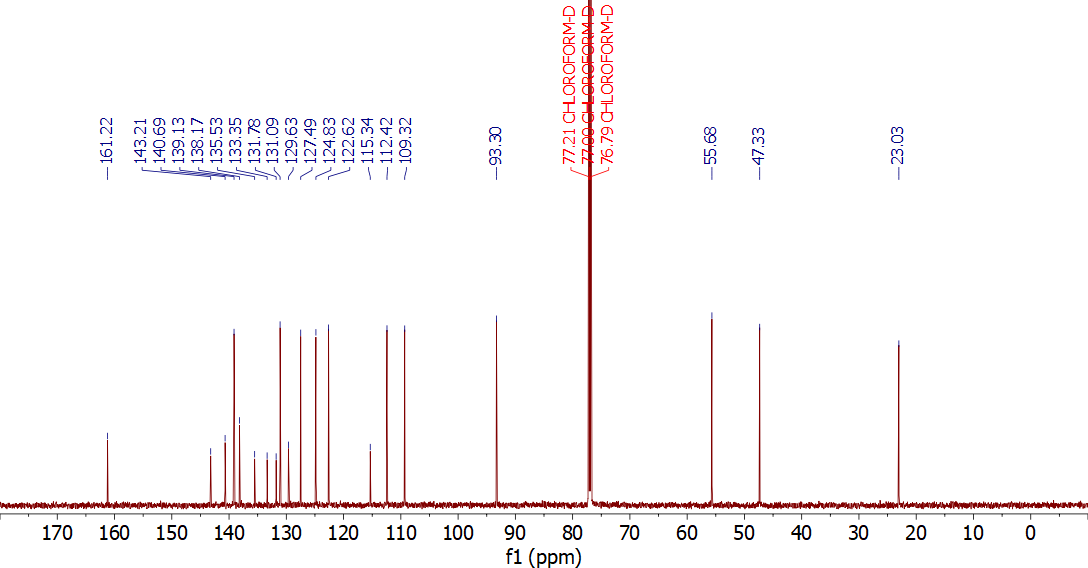


# ESI-HRMS spectrum of 9‐[(3,4‐dichlorophenyl)methyl]‐7‐methoxy‐1‐methyl‐9*H*‐pyrido[3,4‐*b*]indole (17):


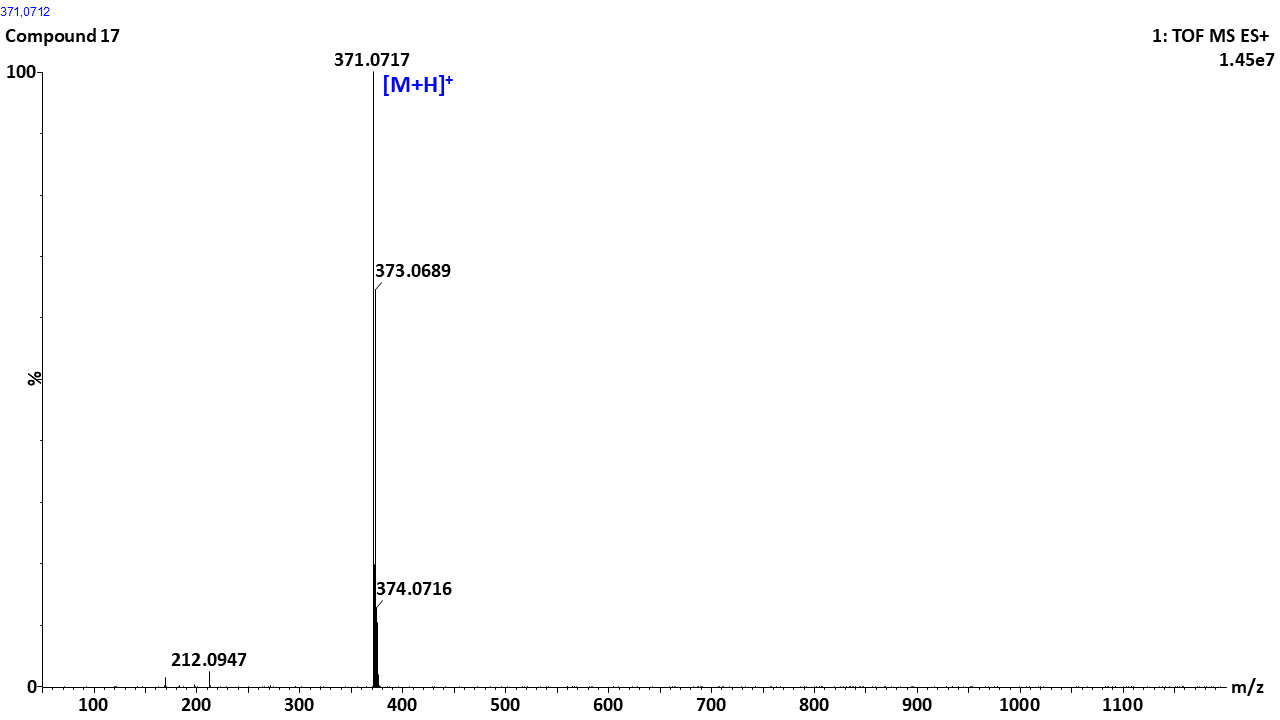


# HPLC-UV chromatogram of 9‐[(3,4‐dichlorophenyl)methyl]‐7‐methoxy‐1‐methyl‐9*H*‐pyrido[3,4‐*b*]indole (17):

# ^1^H NMR spectrum of 9‐[(2‐bromophenyl)methyl]‐7‐methoxy‐1‐methyl‐9*H*‐pyrido[3,4‐*b*]indole (18):


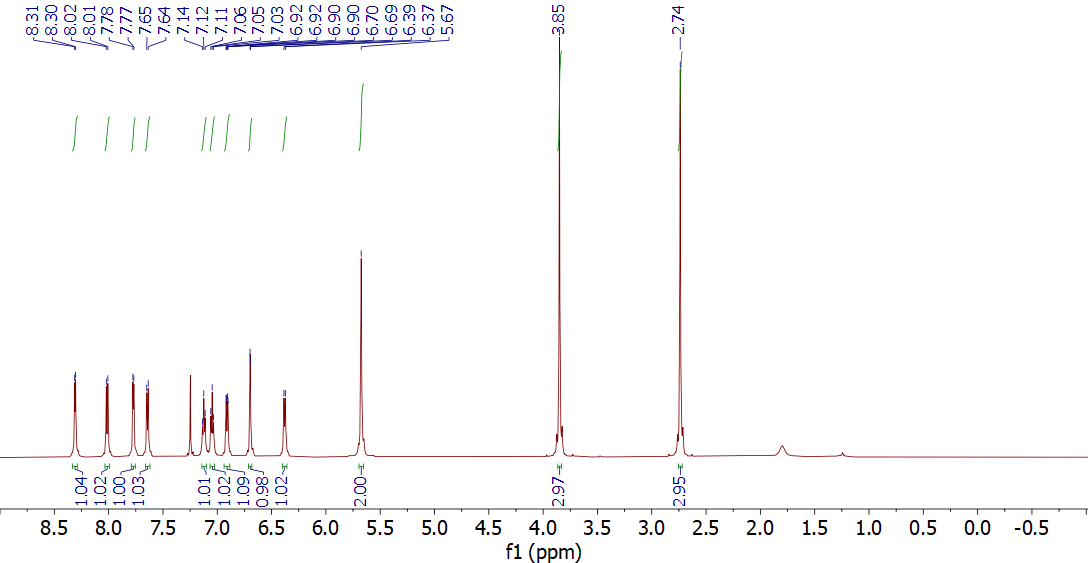


# ^13^C NMR spectrum of 9‐[(2‐bromophenyl)methyl]‐7‐methoxy‐1‐methyl‐9*H*‐pyrido[3,4‐*b*]indole (18):


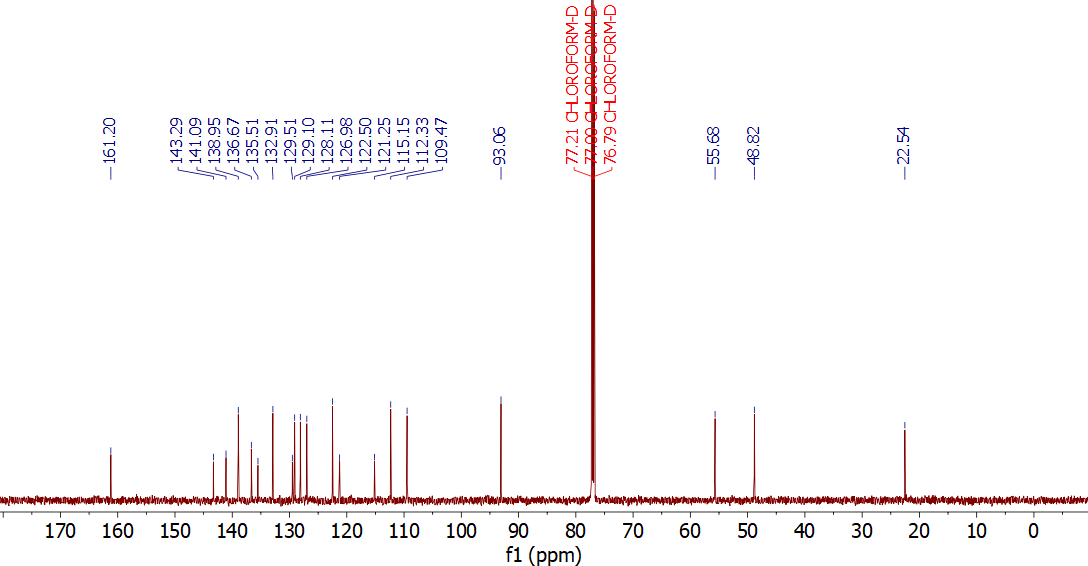


# ESI-HRMS spectrum of 9‐[(2‐bromophenyl)methyl]‐7‐methoxy‐1‐methyl‐9*H*‐pyrido[3,4‐*b*]indole (18):


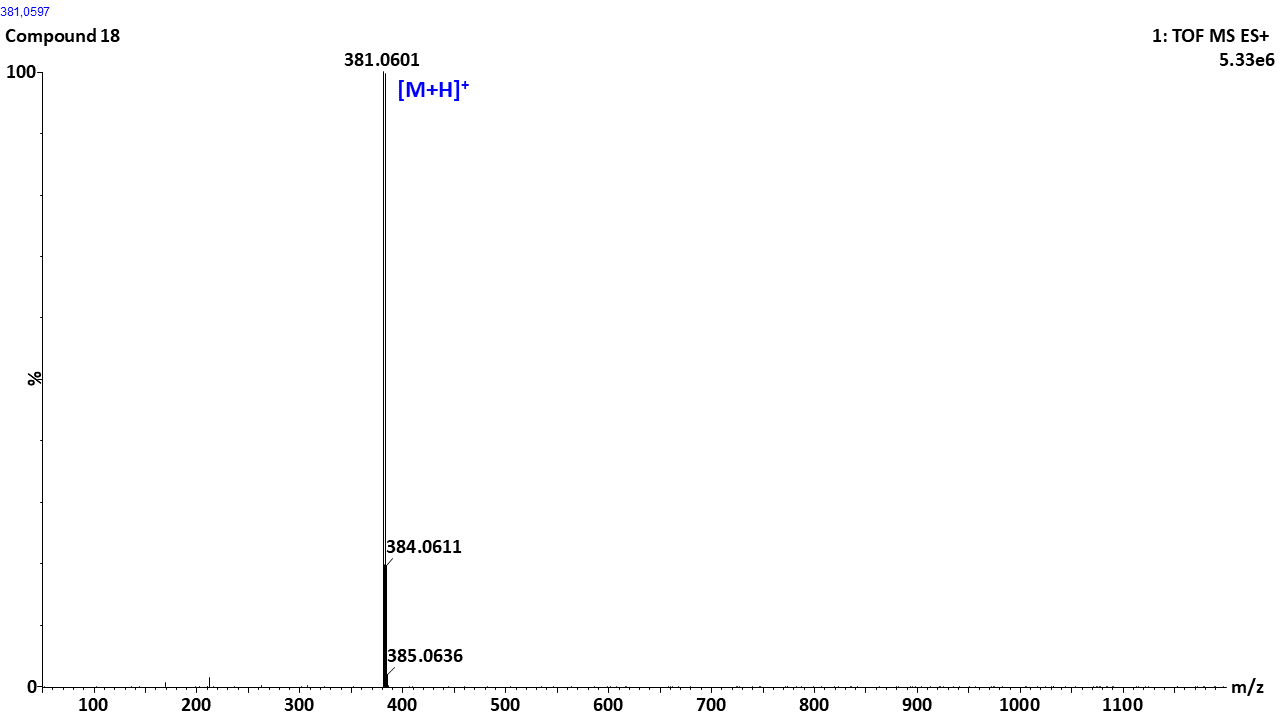


# HPLC-UV chromatogram of 9‐[(2‐bromophenyl)methyl]‐7‐methoxy‐1‐methyl‐9*H*‐pyrido[3,4‐*b*]indole (18):

# ^1^H NMR spectrum of 9‐[(3‐bromophenyl)methyl]‐7‐methoxy‐1‐methyl‐9*H*‐pyrido[3,4‐*b*]indole (19):

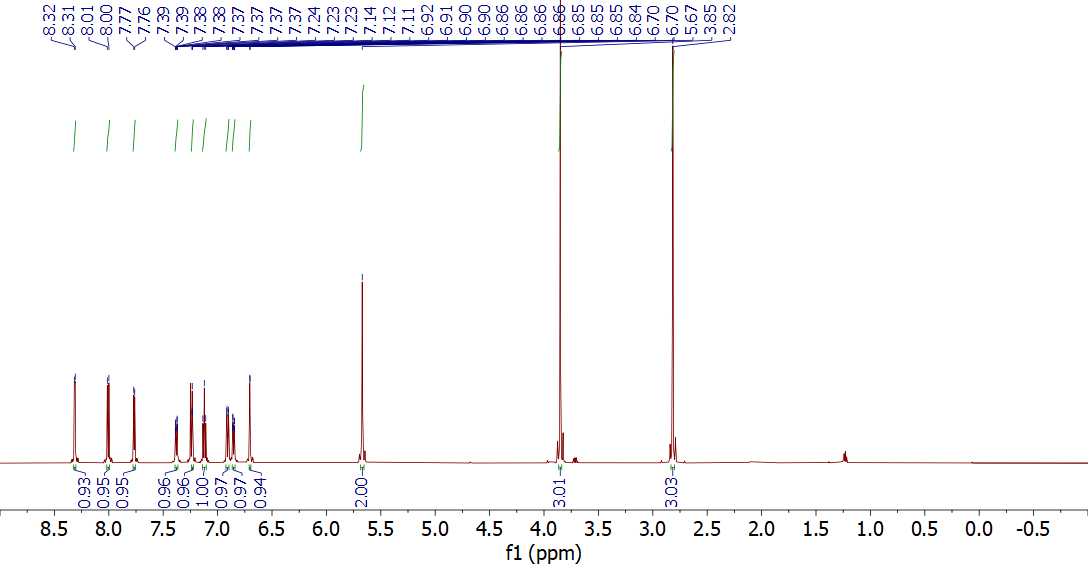


# ^13^C NMR spectrum of 9‐[(3‐bromophenyl)methyl]‐7‐methoxy‐1‐methyl‐9*H*‐pyrido[3,4‐*b*]indole (19):


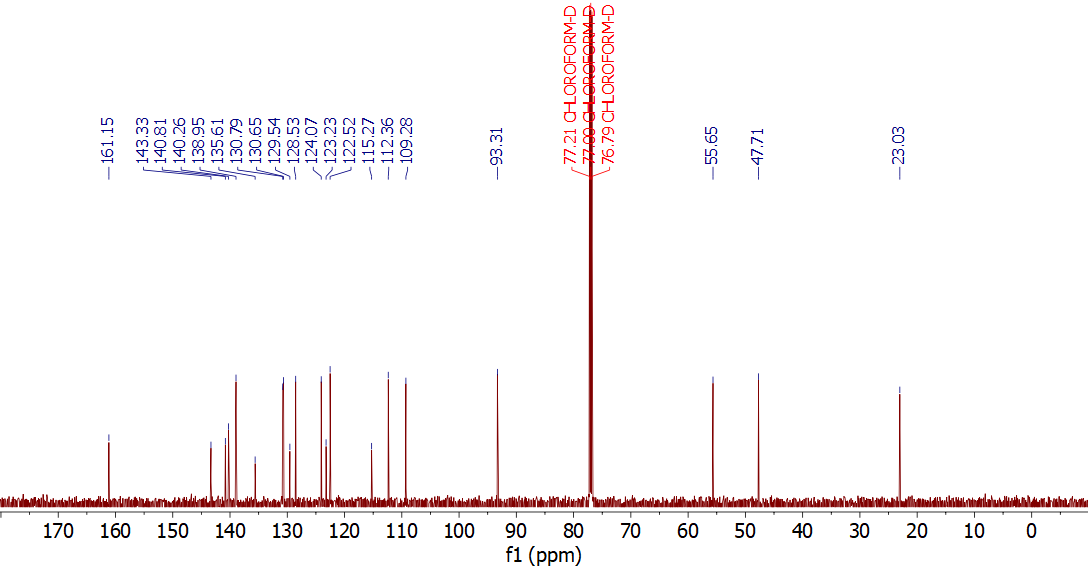


# ESI-HRMS spectrum of 9‐[(3‐bromophenyl)methyl]‐7‐methoxy‐1‐methyl‐9*H*‐pyrido[3,4‐*b*]indole (19):


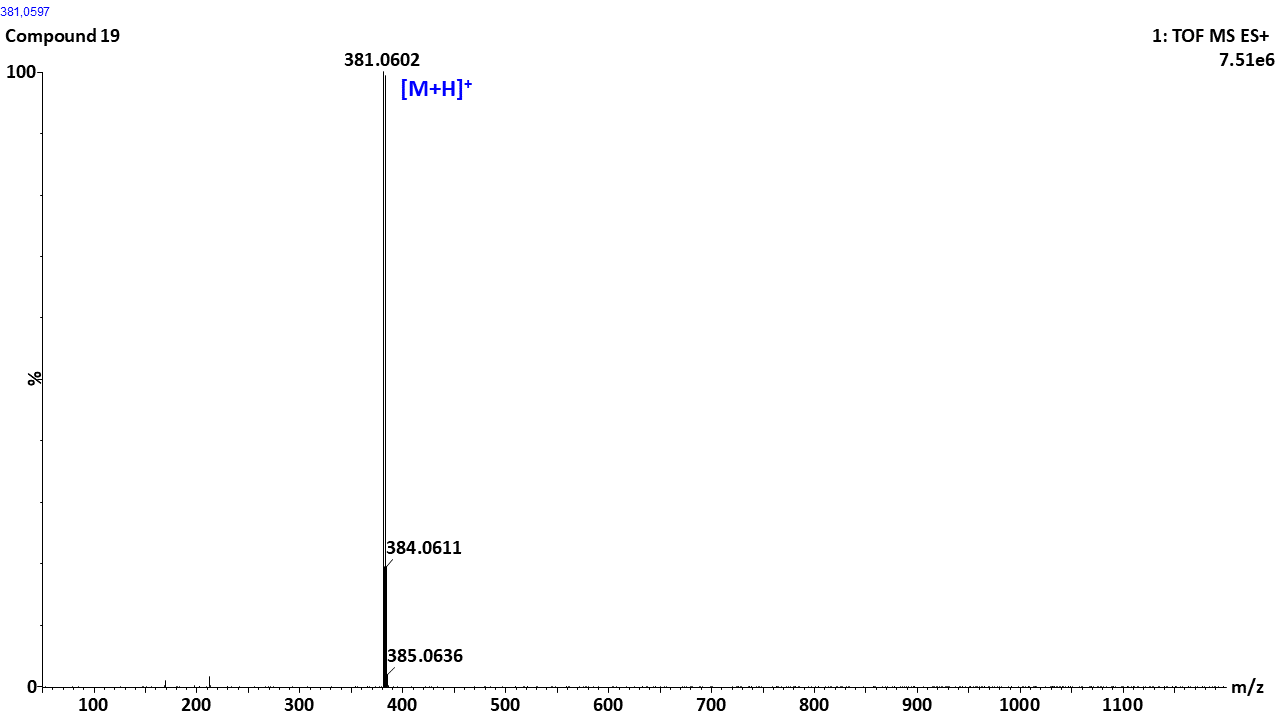


# HPLC-UV chromatogram of 9‐[(3‐bromophenyl)methyl]‐7‐methoxy‐1‐methyl‐9*H*‐pyrido[3,4‐*b*]indole (19):

# ^1^H NMR spectrum of 9‐[(4‐bromophenyl)methyl]‐7‐methoxy‐1‐methyl‐9*H*‐pyrido[3,4‐*b*]indole (20):

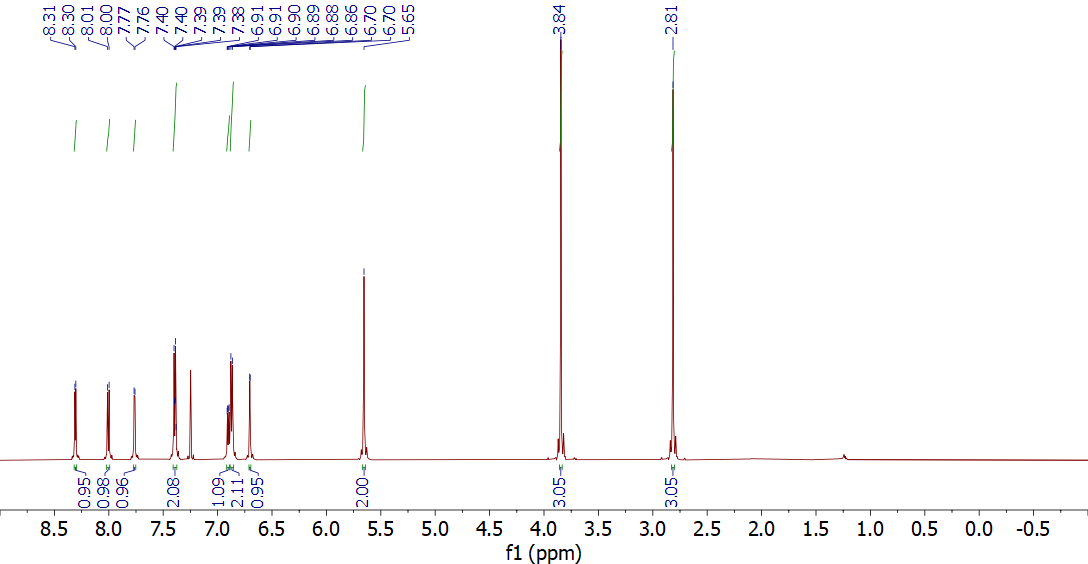


# ^13^C NMR spectrum of 9‐[(4‐bromophenyl)methyl]‐7‐methoxy‐1‐methyl‐9*H*‐pyrido[3,4‐*b*]indole (20):


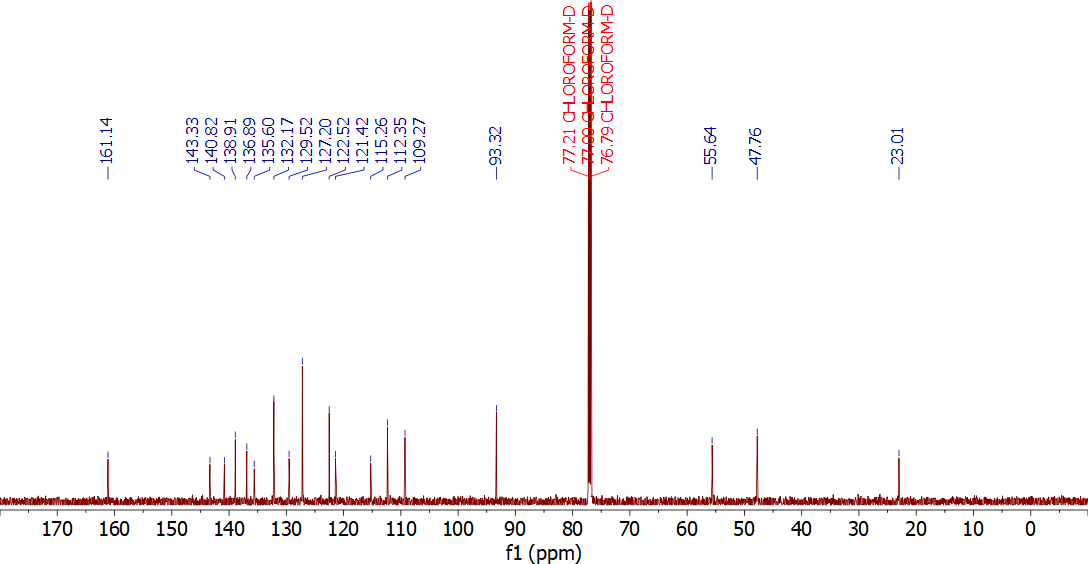


# ESI-HRMS spectrum of 9‐[(4‐bromophenyl)methyl]‐7‐methoxy‐1‐methyl‐9*H*‐pyrido[3,4‐*b*]indole (20):


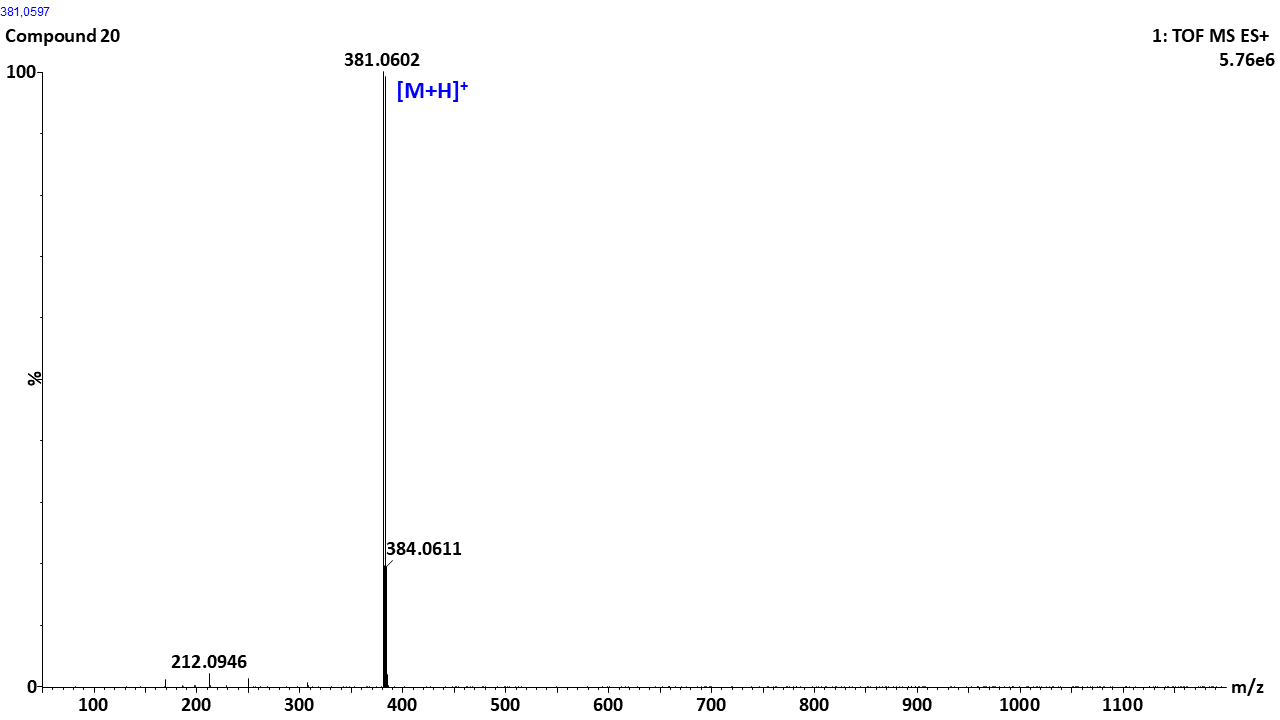


# HPLC-UV chromatogram of 9‐[(4‐bromophenyl)methyl]‐7‐methoxy‐1‐methyl‐9*H*‐pyrido[3,4‐*b*]indole (20):

# ^1^H NMR spectrum of 7‐methoxy‐1‐methyl‐9‐[(2‐nitrophenyl)methyl]‐9*H*‐pyrido[3,4‐*b*]indole (21):

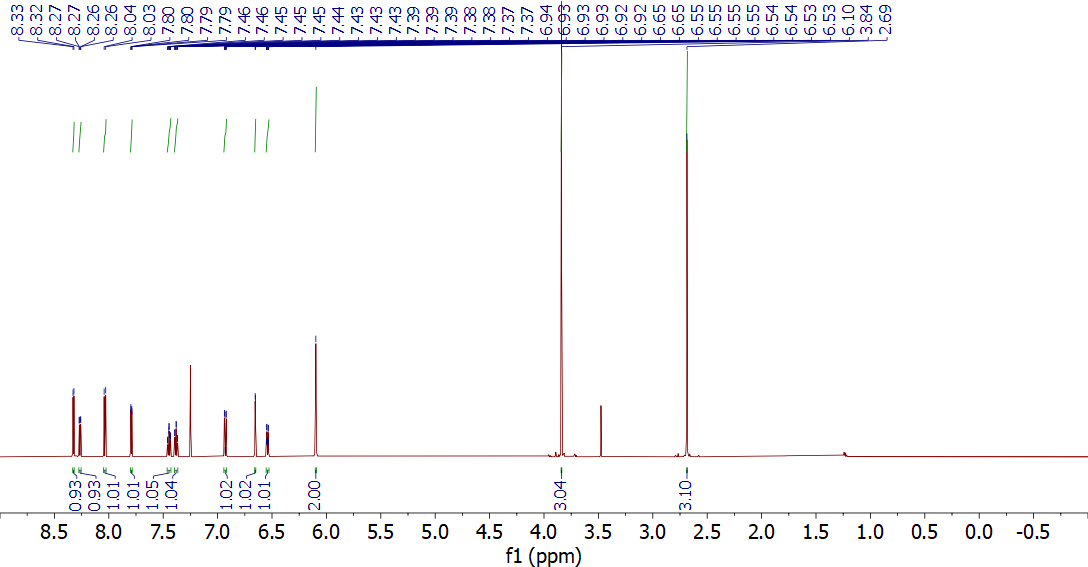


# ^13^C NMR spectrum of 7‐methoxy‐1‐methyl‐9‐[(2‐nitrophenyl)methyl]‐9*H*‐pyrido[3,4‐*b*]indole (21):


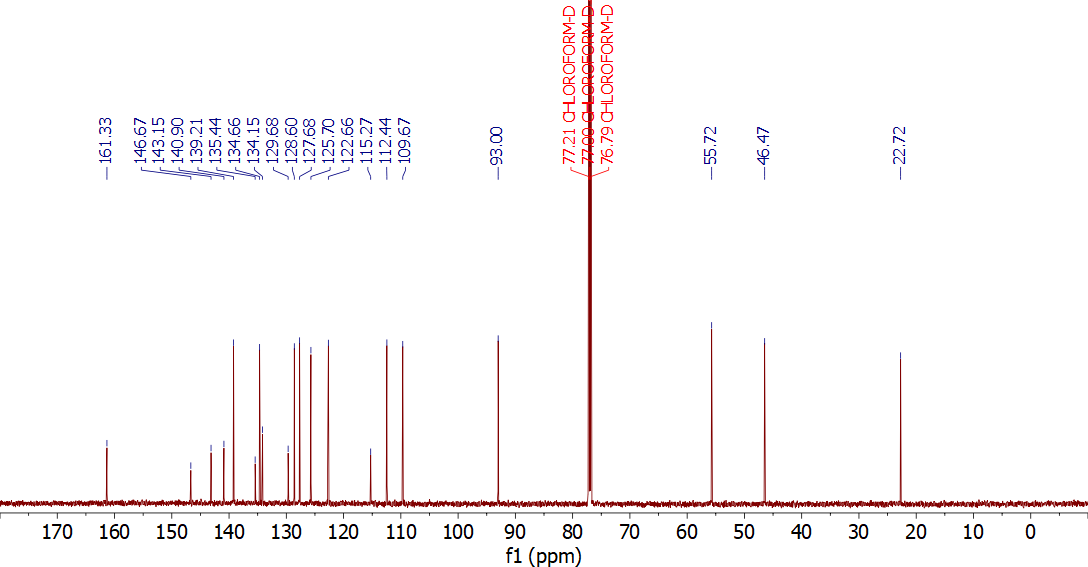


# ESI-HRMS spectrum of 7‐methoxy‐1‐methyl‐9‐[(2‐nitrophenyl)methyl]‐9*H*‐pyrido[3,4‐*b*]indole (21):


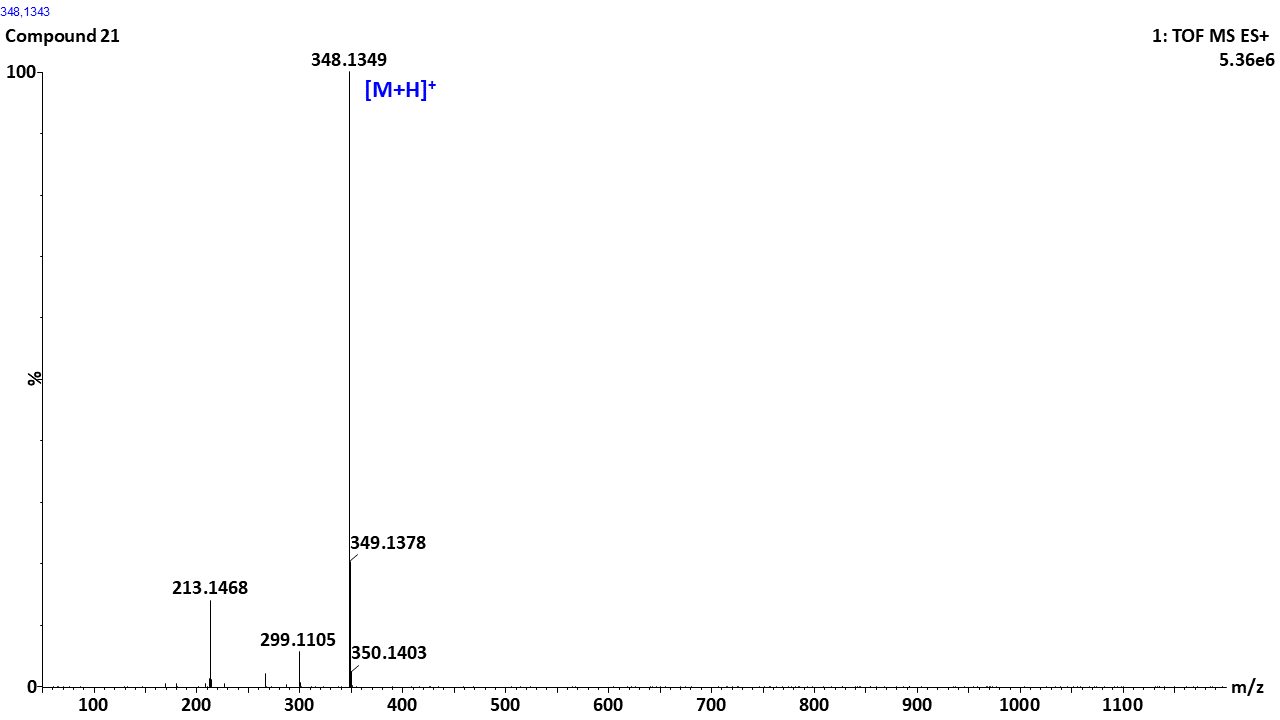


# HPLC-UV chromatogram of 7‐methoxy‐1‐methyl‐9‐[(2‐nitrophenyl)methyl]‐9*H*‐pyrido[3,4‐*b*]indole (21):

# ^1^H NMR spectrum of 7‐methoxy‐1‐methyl‐9‐[(3‐nitrophenyl)methyl]‐9*H*‐pyrido[3,4‐*b*]indole (22):

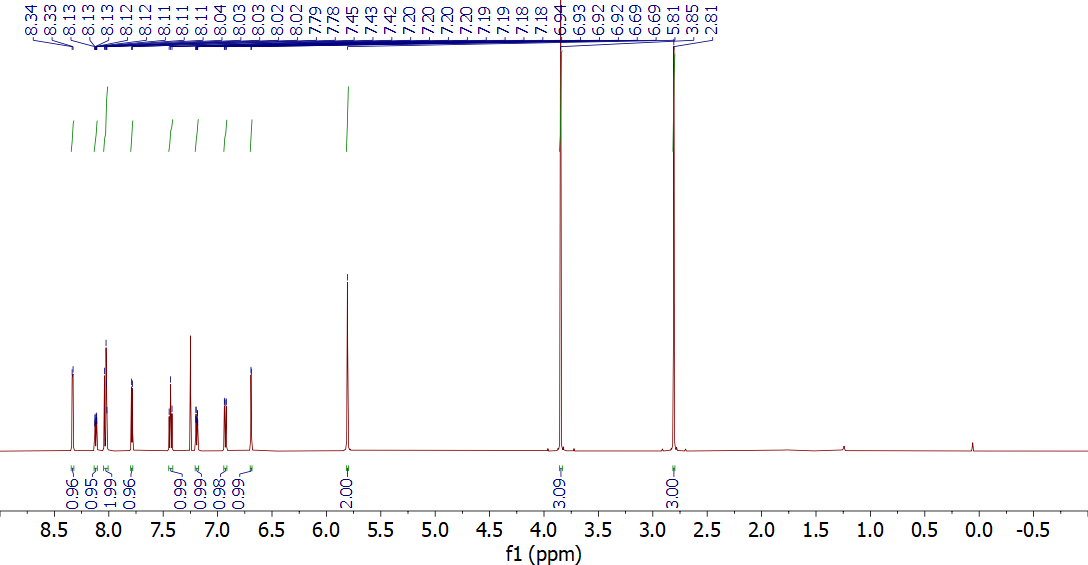


# ^13^C NMR spectrum of 7‐methoxy‐1‐methyl‐9‐[(3‐nitrophenyl)methyl]‐9*H*‐pyrido[3,4‐*b*]indole (22):


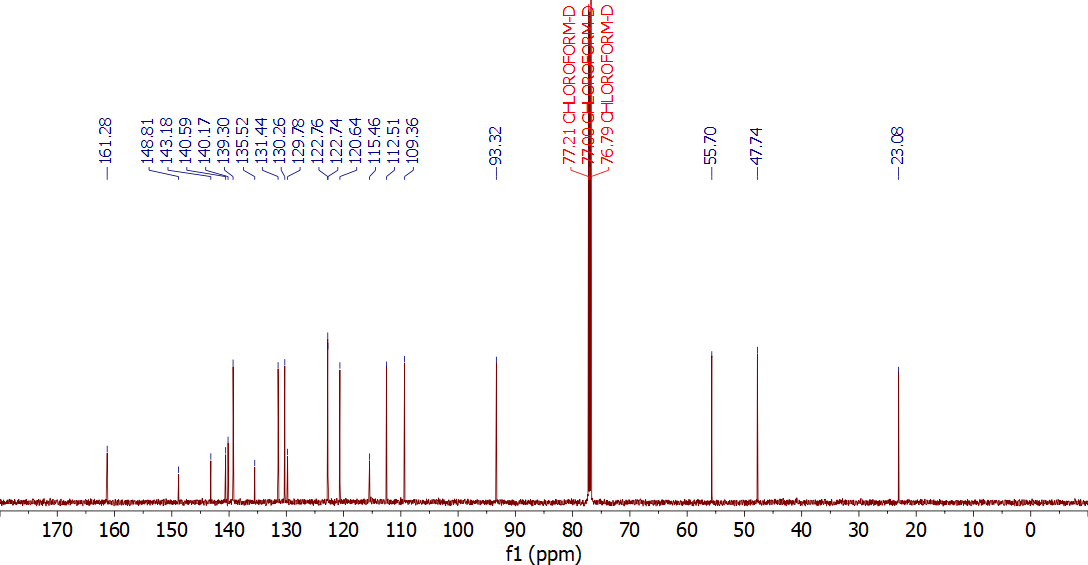


# ESI-HRMS spectrum of 7‐methoxy‐1‐methyl‐9‐[(3‐nitrophenyl)methyl]‐9*H*‐pyrido[3,4‐*b*]indole (22):


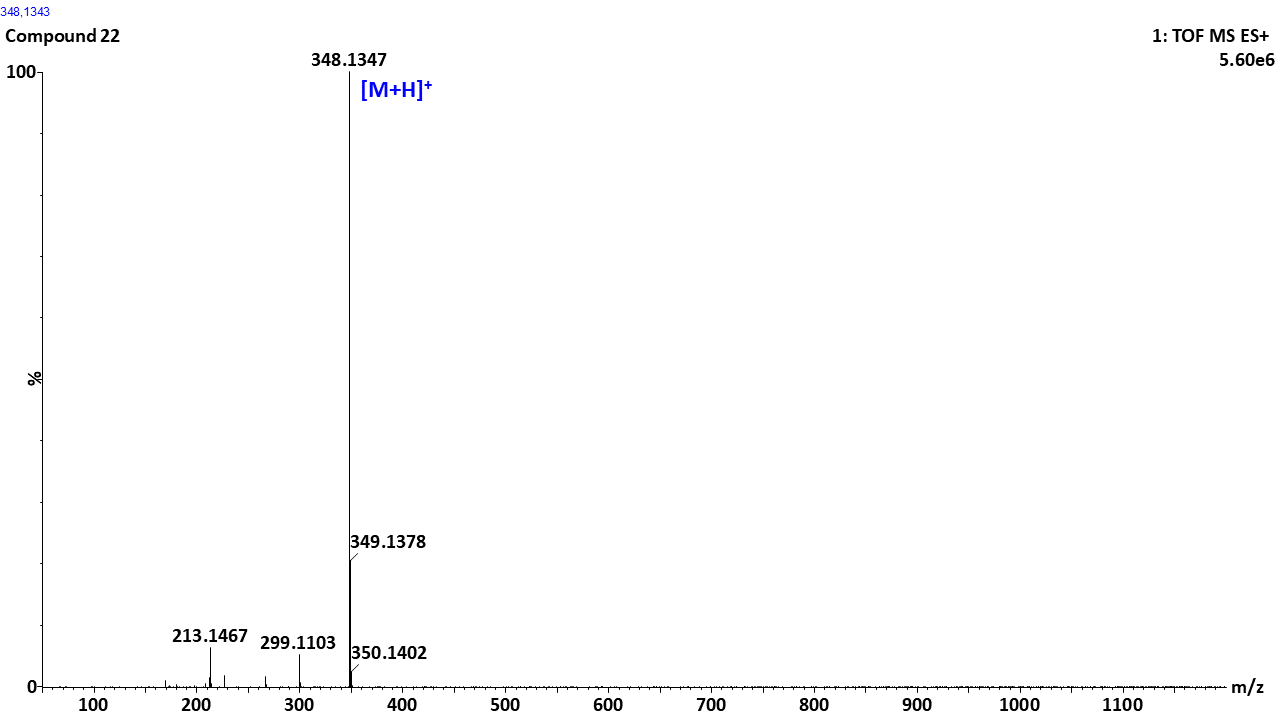


# HPLC-UV chromatogram of 7‐methoxy‐1‐methyl‐9‐[(3‐nitrophenyl)methyl]‐9*H*‐pyrido[3,4‐*b*]indole (22):

# ^1^H NMR spectrum of 7‐methoxy‐1‐methyl‐9‐[(4‐nitrophenyl)methyl]‐9H‐pyrido[3,4‐*b*]indole (23):

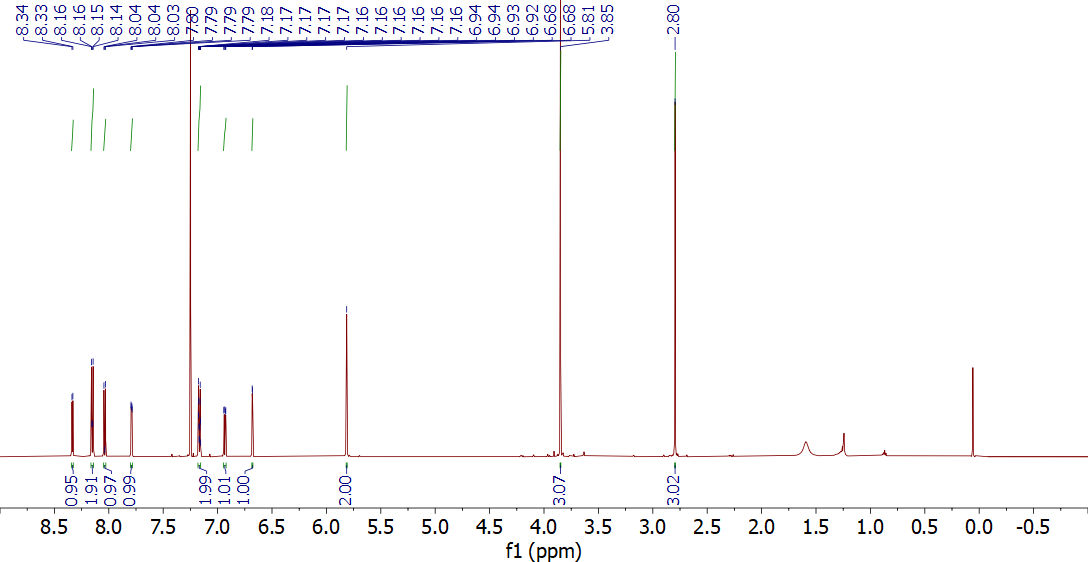


# ^13^C NMR spectrum of 7‐methoxy‐1‐methyl‐9‐[(4‐nitrophenyl)methyl]‐9H‐pyrido[3,4‐*b*]indole (23):


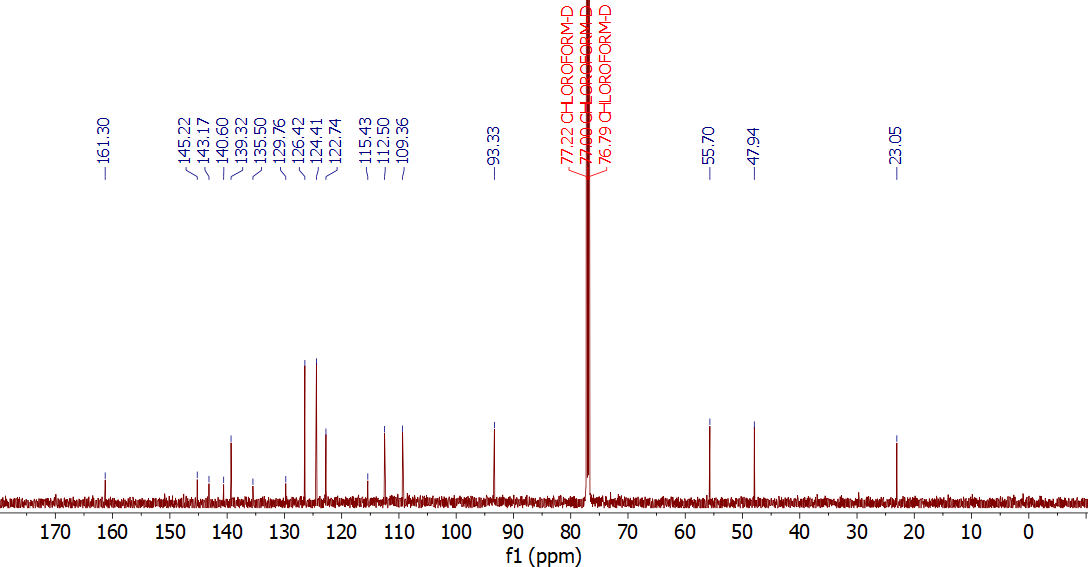


# ESI-HRMS spectrum of 7‐methoxy‐1‐methyl‐9‐[(4‐nitrophenyl)methyl]‐9H‐pyrido[3,4‐*b*]indole (23):

**
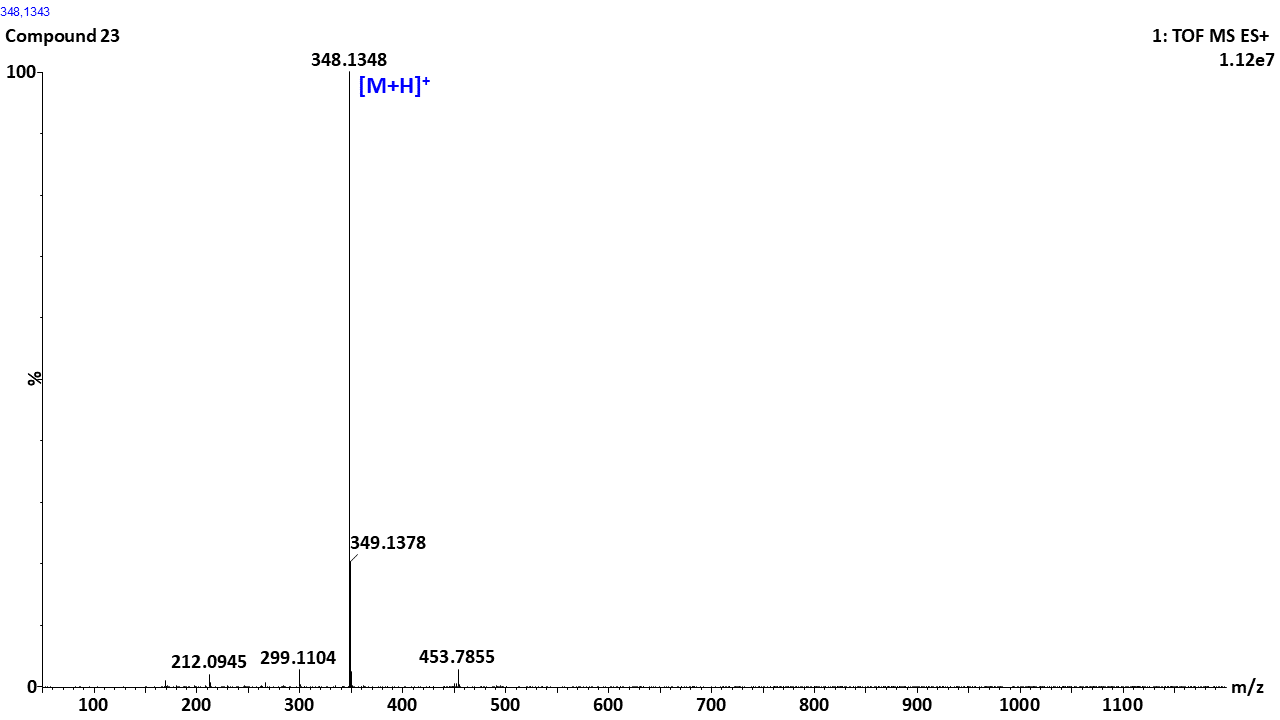
**

# HPLC-UV chromatogram of 7‐methoxy‐1‐methyl‐9‐[(4‐nitrophenyl)methyl]‐9H‐pyrido[3,4‐*b*]indole (23):

# ^1^H NMR spectrum of 7‐methoxy‐1‐methyl‐9‐[(naphthalen‐2‐yl)methyl]‐9*H*‐pyrido[3,4‐*b*]indole (24):


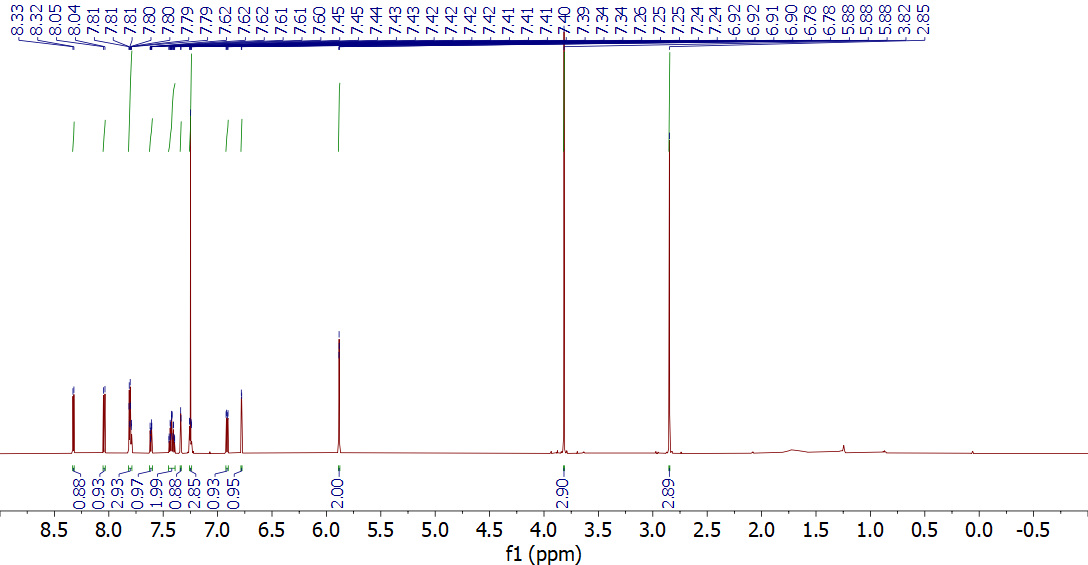


# ^13^C NMR spectrum of 7‐methoxy‐1‐methyl‐9‐[(naphthalen‐2‐yl)methyl]‐9*H*‐pyrido[3,4‐*b*]indole (24):


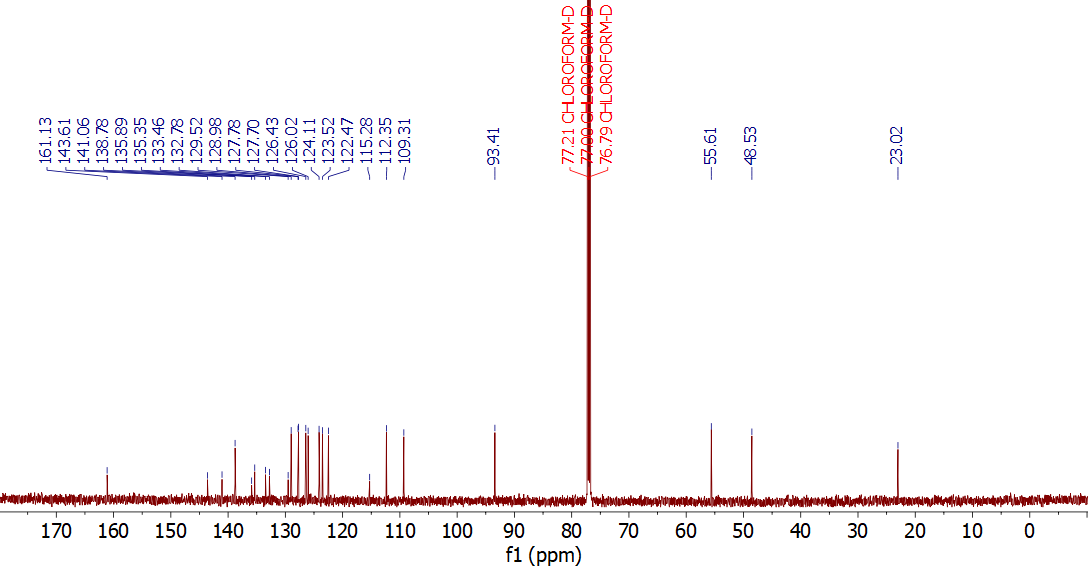


# ESI-HRMS spectrum of 7‐methoxy‐1‐methyl‐9‐[(naphthalen‐2‐yl)methyl]‐9*H*‐pyrido[3,4‐*b*]indole (24):


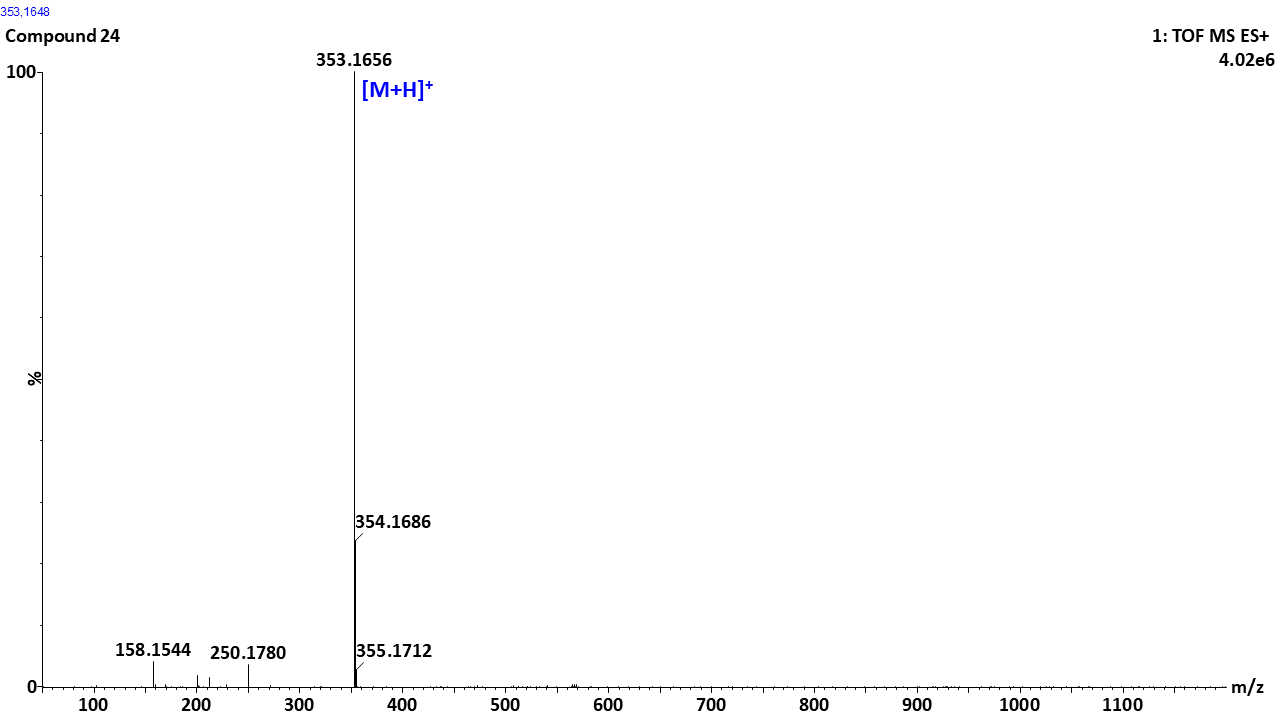


# HPLC-UV chromatogram of 7‐methoxy‐1‐methyl‐9‐[(naphthalen‐2‐yl)methyl]‐9*H*‐pyrido[3,4‐*b*]indole (24):

# ^1^H NMR spectrum of 7‐methoxy‐1‐methyl‐9‐propyl‐9*H*‐pyrido[3,4‐*b*]indole (25):


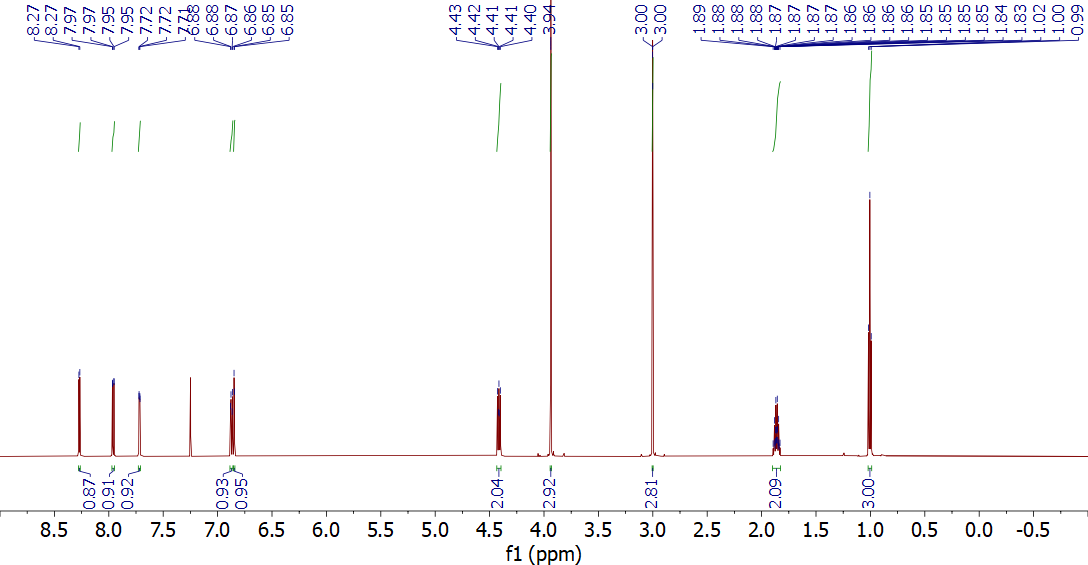


# ^13^C NMR spectrum of 7‐methoxy‐1‐methyl‐9‐propyl‐9*H*‐pyrido[3,4‐*b*]indole (25):


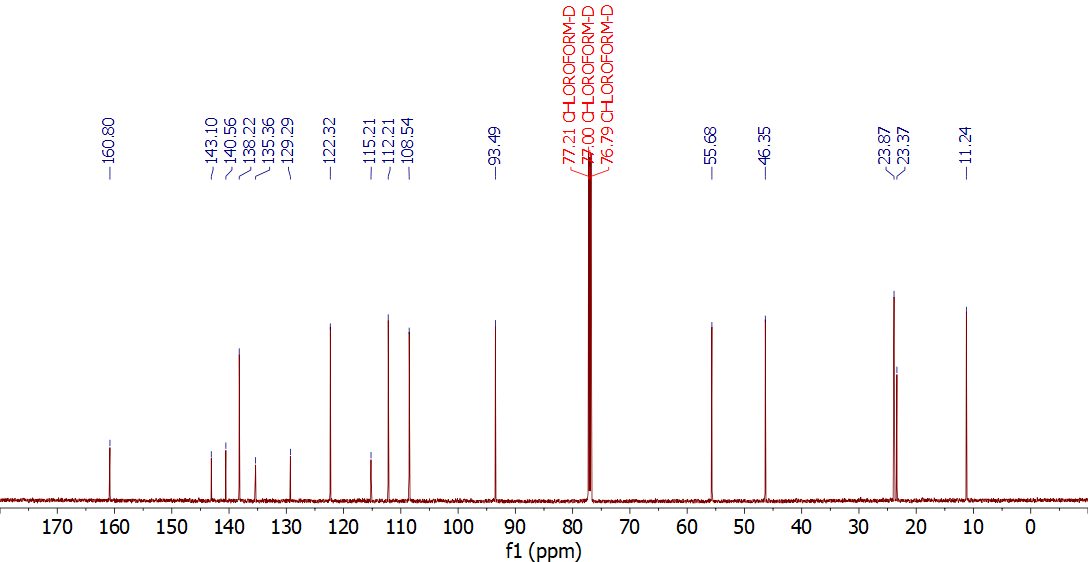


# ESI-HRMS spectrum of 7‐methoxy‐1‐methyl‐9‐propyl‐9*H*‐pyrido[3,4‐*b*]indole (25):


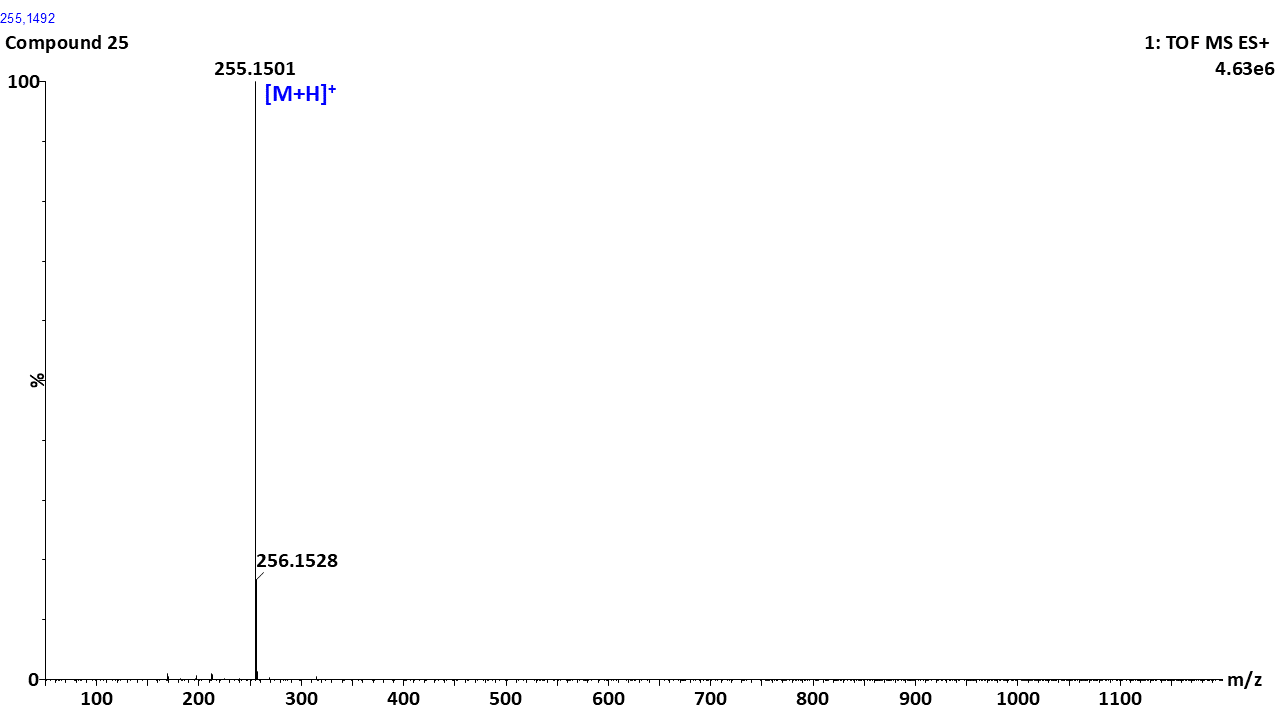


# HPLC-UV chromatogram of 7‐methoxy‐1‐methyl‐9‐propyl‐9*H*‐pyrido[3,4‐*b*]indole (25):

# ^1^H NMR spectrum of 7‐methoxy‐1‐methyl‐9‐(propan‐2‐yl)‐9*H*‐pyrido[3,4‐*b*]indole (26):

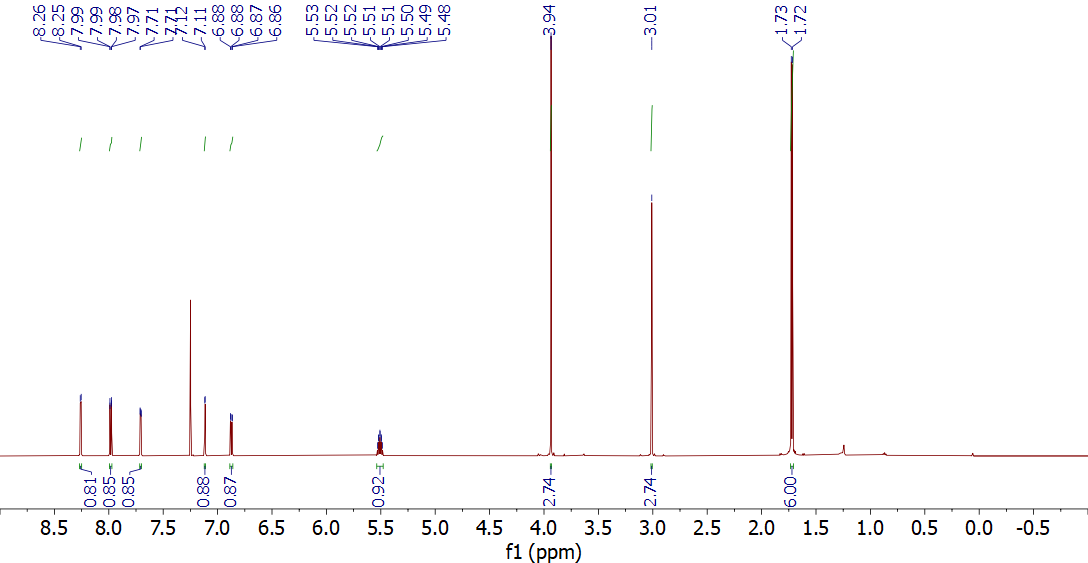


# ^13^C NMR spectrum of 7‐methoxy‐1‐methyl‐9‐(propan‐2‐yl)‐9*H*‐pyrido[3,4‐*b*]indole (26):


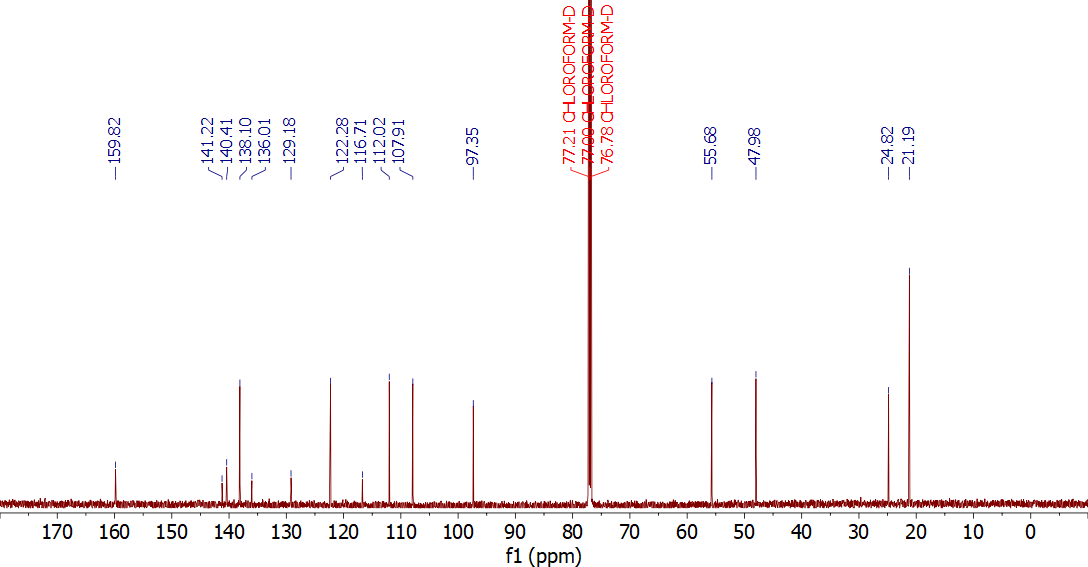


# ESI-HRMS spectrum of 7‐methoxy‐1‐methyl‐9‐(propan‐2‐yl)‐9*H*‐pyrido[3,4‐*b*]indole (26):


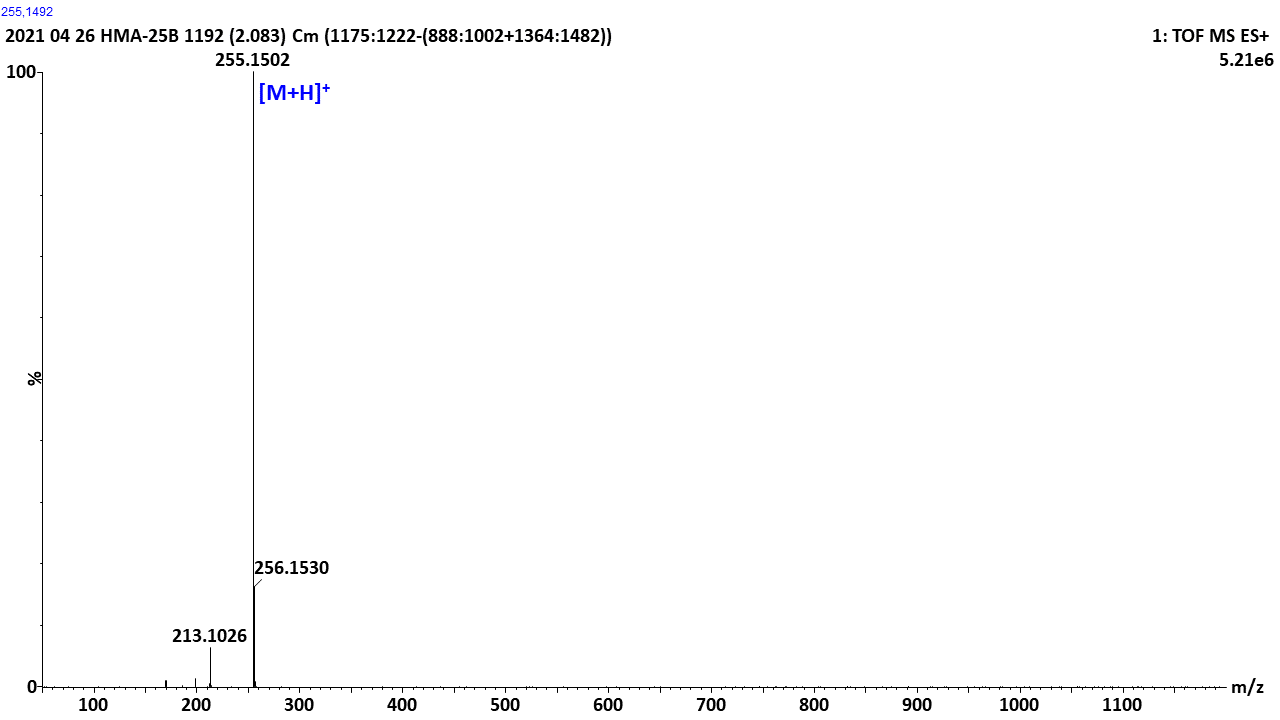


# HPLC-UV chromatogram of 7‐methoxy‐1‐methyl‐9‐(propan‐2‐yl)‐9*H*‐pyrido[3,4‐*b*]indole (26):

# ^1^H NMR spectrum of 7‐methoxy‐1‐methyl‐9‐(prop‐2‐en‐1‐yl)‐9*H*‐pyrido[3,4‐*b*]indole (27):


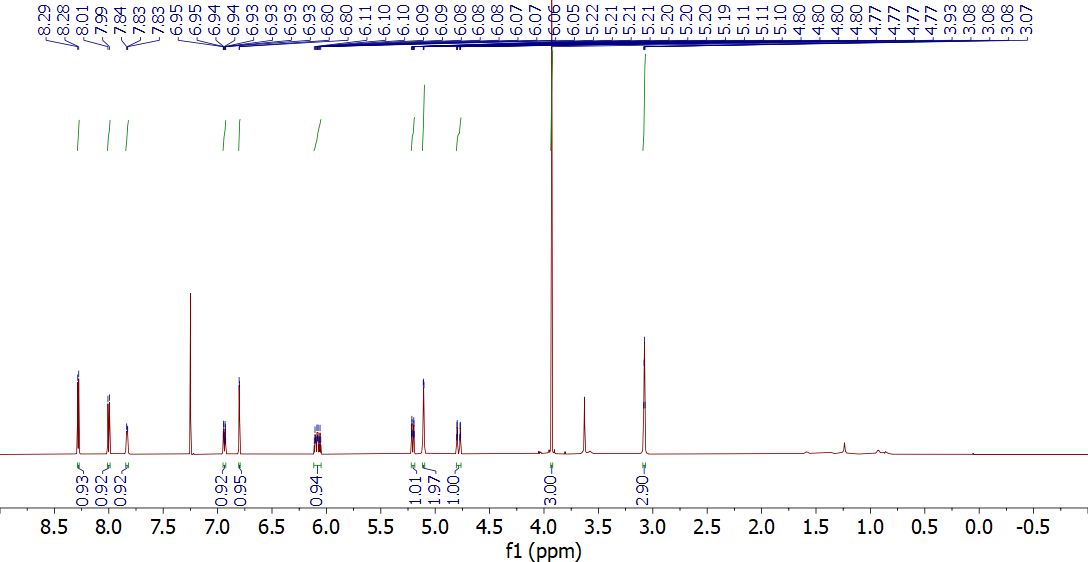


# ^13^C NMR spectrum of 7‐methoxy‐1‐methyl‐9‐(prop‐2‐en‐1‐yl)‐9*H*‐pyrido[3,4‐*b*]indole (27):


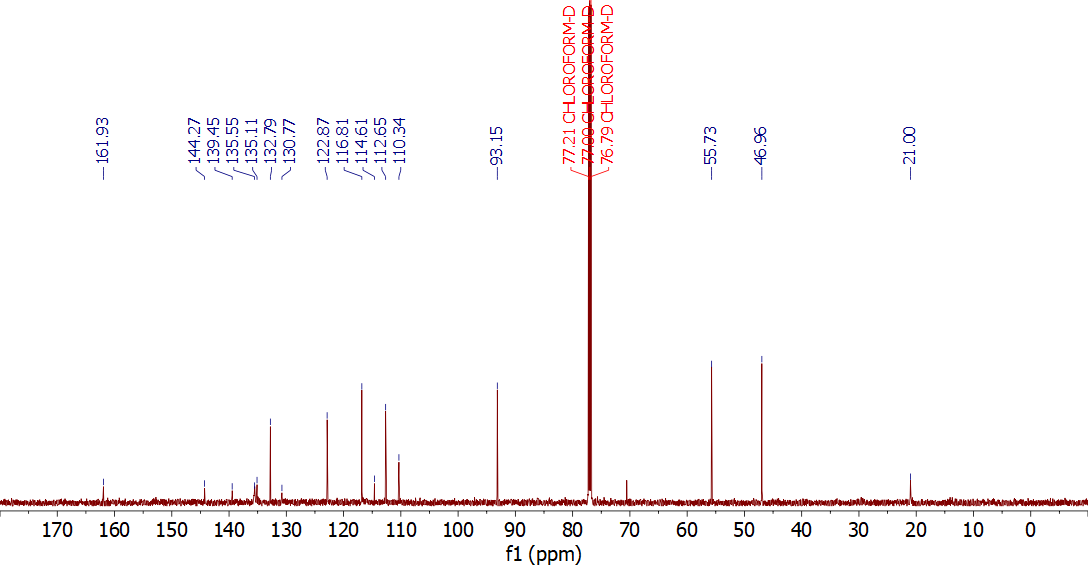


# ESI-HRMS spectrum of 7‐methoxy‐1‐methyl‐9‐(prop‐2‐en‐1‐yl)‐9*H*‐pyrido[3,4‐*b*]indole (27):


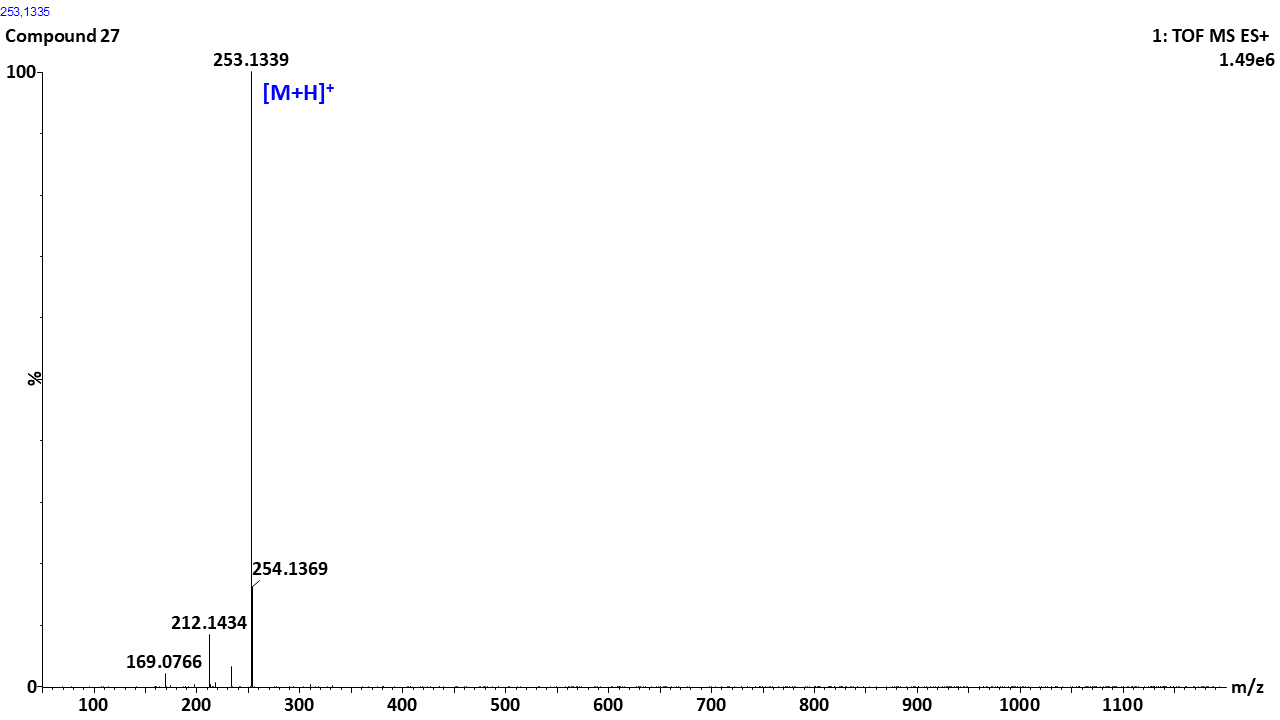


# HPLC-UV chromatogram of 7‐methoxy‐1‐methyl‐9‐(prop‐2‐en‐1‐yl)‐9*H*‐pyrido[3,4‐*b*]indole (27):

# ^1^H NMR spectrum of 7‐methoxy‐1‐methyl‐9‐(prop‐2‐yn‐1‐yl)‐9*H*‐pyrido[3,4‐*b*]indole (28):

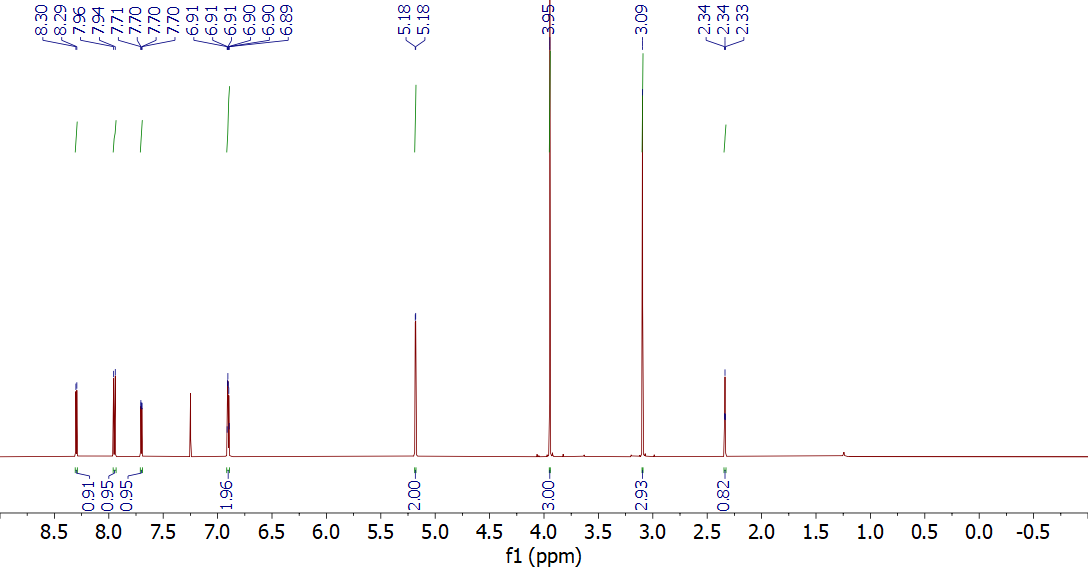


# ^13^C NMR spectrum of 7‐methoxy‐1‐methyl‐9‐(prop‐2‐yn‐1‐yl)‐9*H*‐pyrido[3,4‐*b*]indole (28):


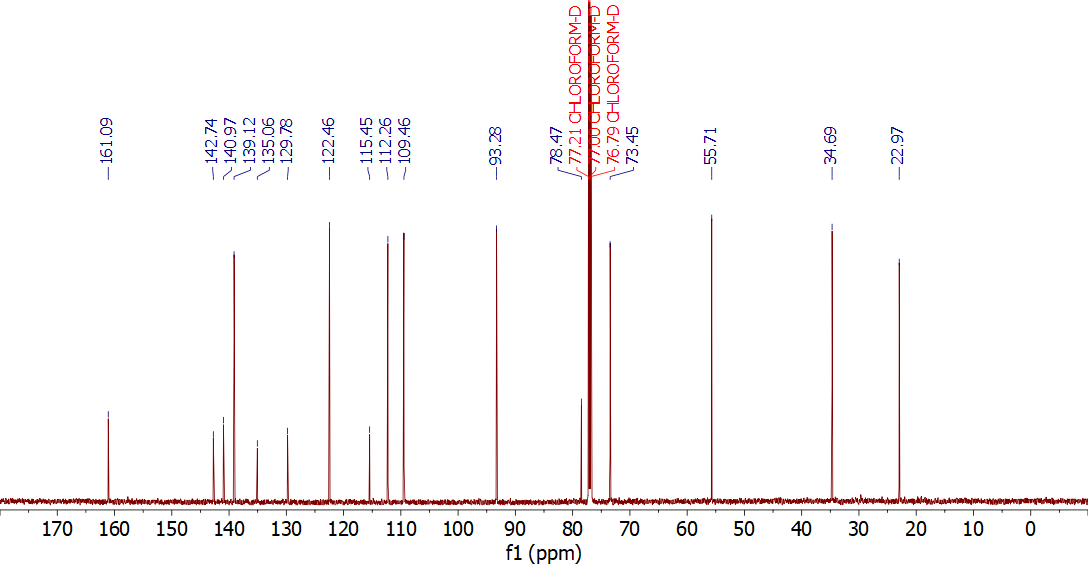


# ESI-HRMS spectrum of 7‐methoxy‐1‐methyl‐9‐(prop‐2‐yn‐1‐yl)‐9*H*‐pyrido[3,4‐*b*]indole (28):


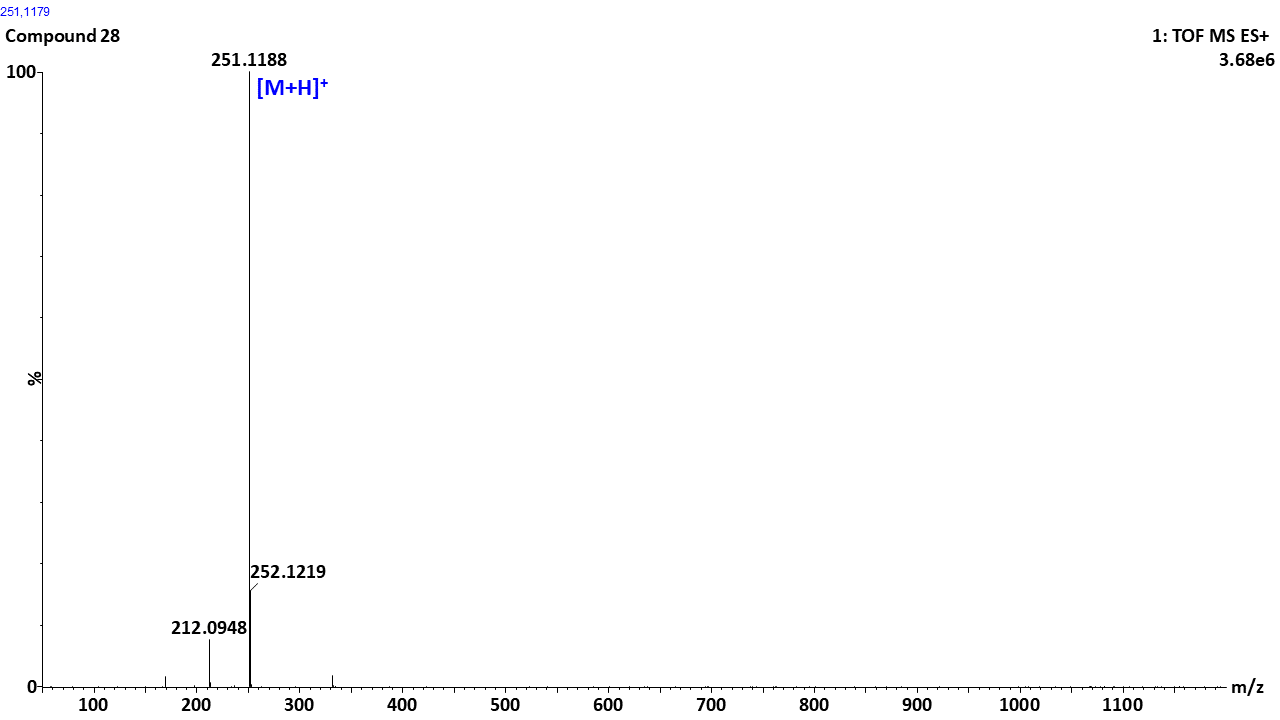


# HPLC-UV chromatogram of 7‐methoxy‐1‐methyl‐9‐(prop‐2‐yn‐1‐yl)‐9*H*‐pyrido[3,4‐*b*]indole (28):

# ^1^H NMR spectrum of 9‐butyl‐7‐methoxy‐1‐methyl‐9*H*‐pyrido[3,4‐*b*]indole (29):


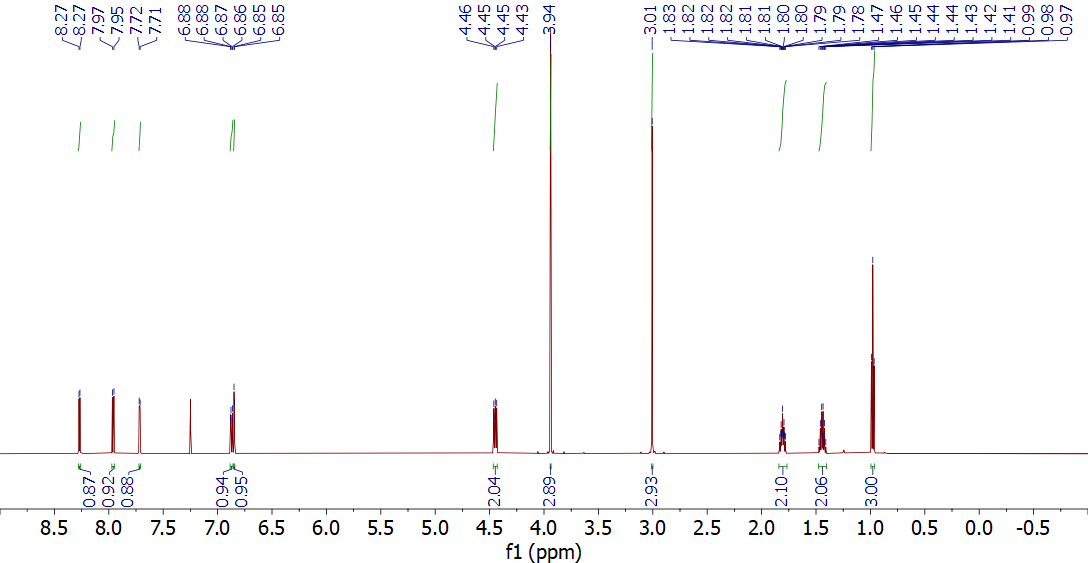


# ^13^C NMR spectrum of 9‐butyl‐7‐methoxy‐1‐methyl‐9*H*‐pyrido[3,4‐*b*]indole (29):


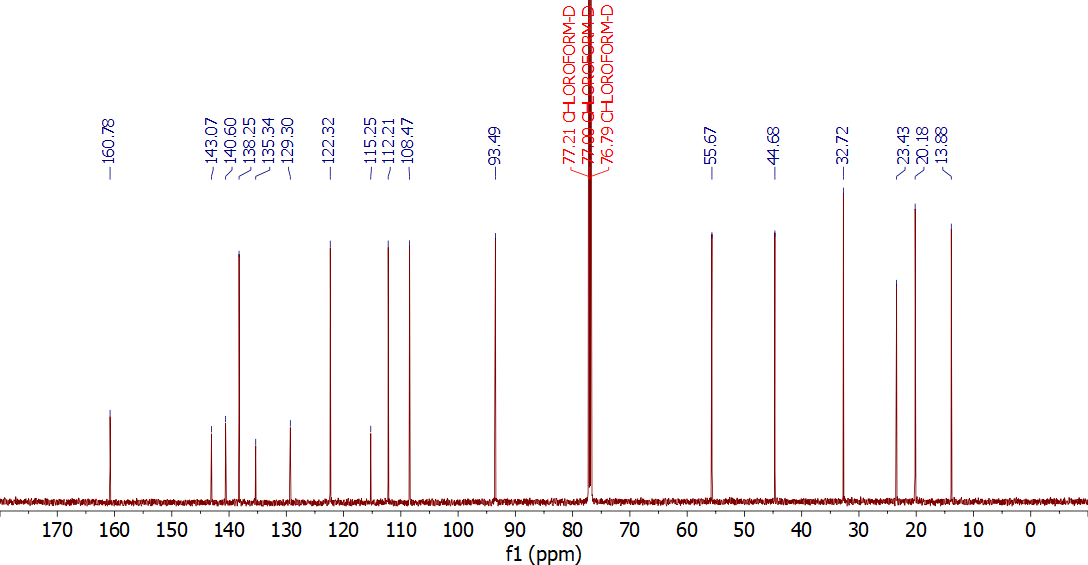


# ESI-HRMS spectrum of 9‐butyl‐7‐methoxy‐1‐methyl‐9*H*‐pyrido[3,4‐*b*]indole (29):


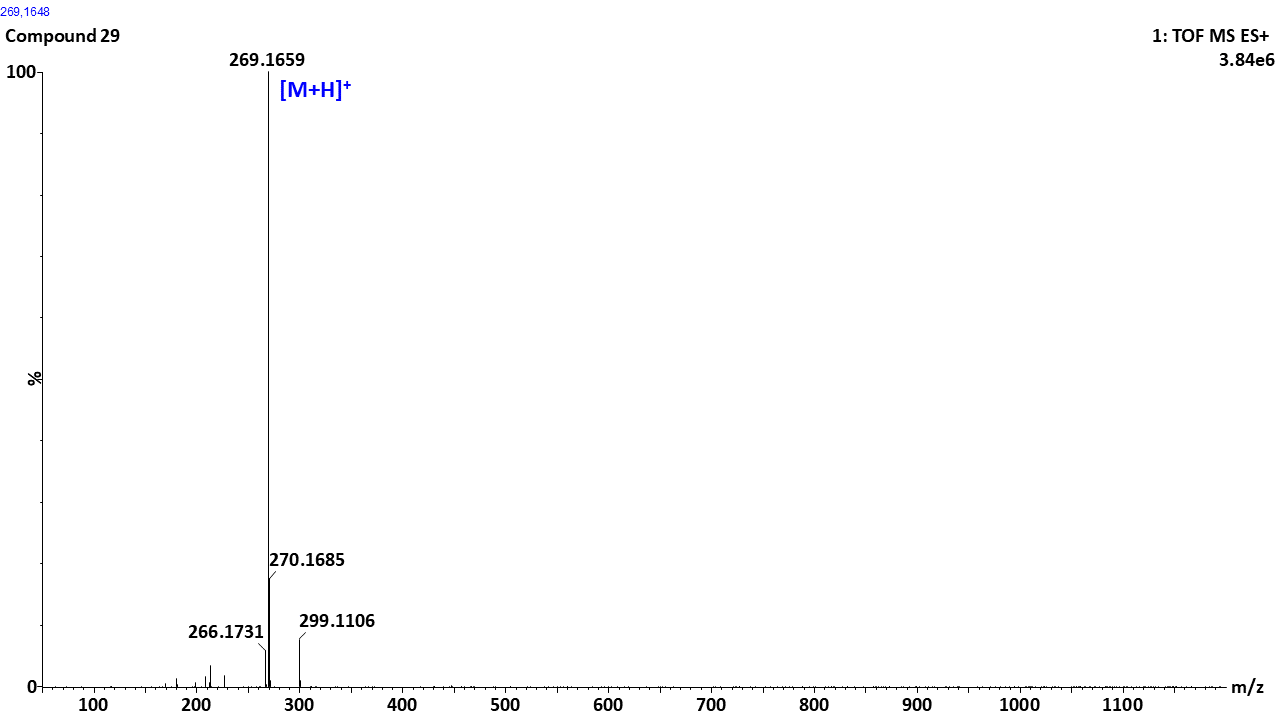


# HPLC-UV chromatogram of 9‐butyl‐7‐methoxy‐1‐methyl‐9*H*‐pyrido[3,4‐*b*]indole (29):

# ^1^H NMR spectrum of 7‐methoxy‐1‐methyl‐9‐pentyl‐9*H*‐pyrido[3,4‐*b*]indole (30):


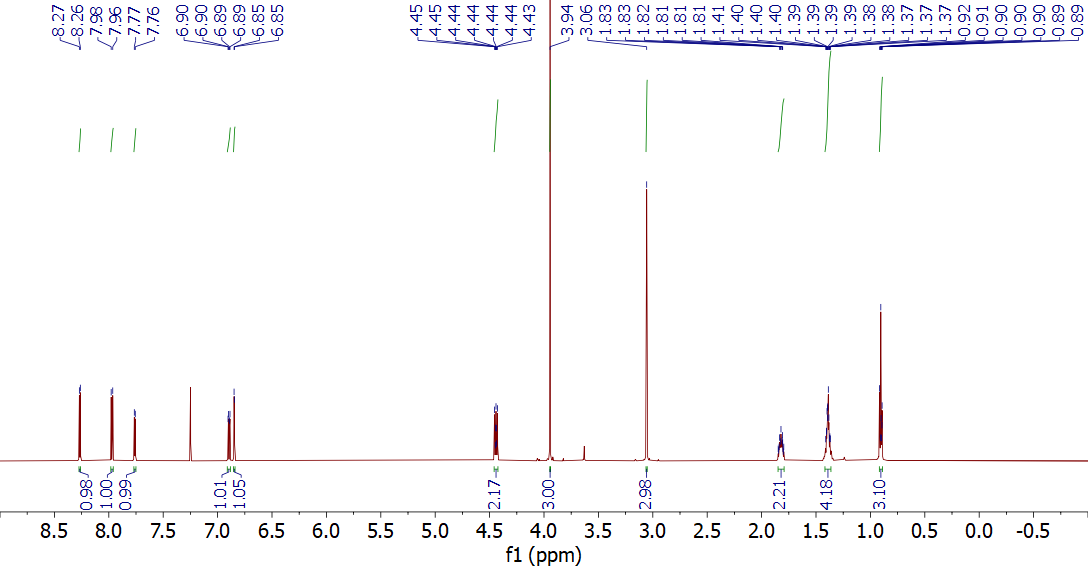


# ^13^C NMR spectrum of 7‐methoxy‐1‐methyl‐9‐pentyl‐9*H*‐pyrido[3,4‐*b*]indole (30):


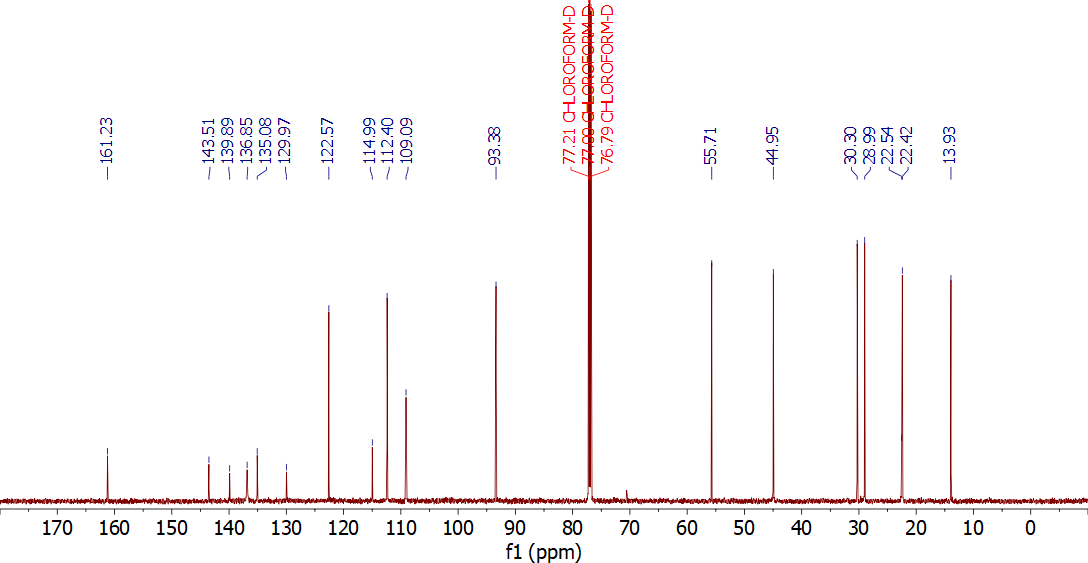


# ESI-HRMS spectrum of 7‐methoxy‐1‐methyl‐9‐pentyl‐9*H*‐pyrido[3,4‐*b*]indole (30):


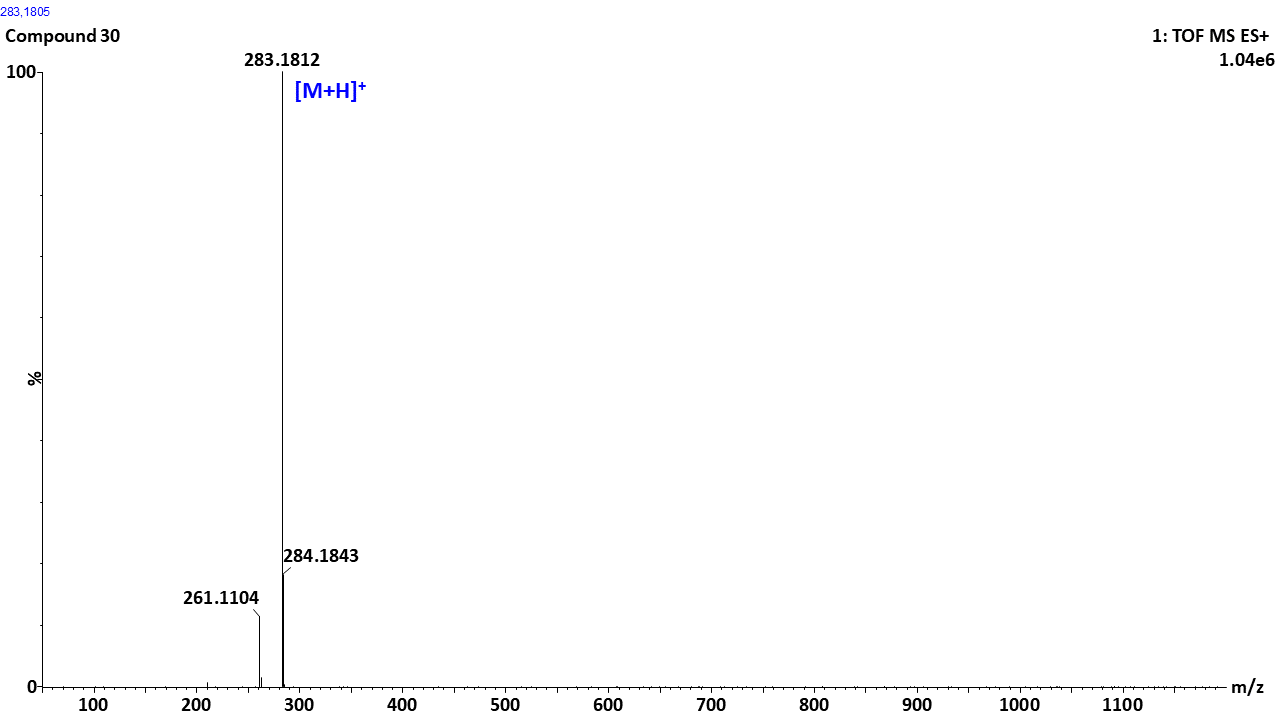


# HPLC-UV chromatogram of 7‐methoxy‐1‐methyl‐9‐pentyl‐9*H*‐pyrido[3,4‐*b*]indole (30):

# ^1^H NMR spectrum of 9‐hexyl‐7‐methoxy‐1‐methyl‐9*H*‐pyrido[3,4‐*b*]indole (31):


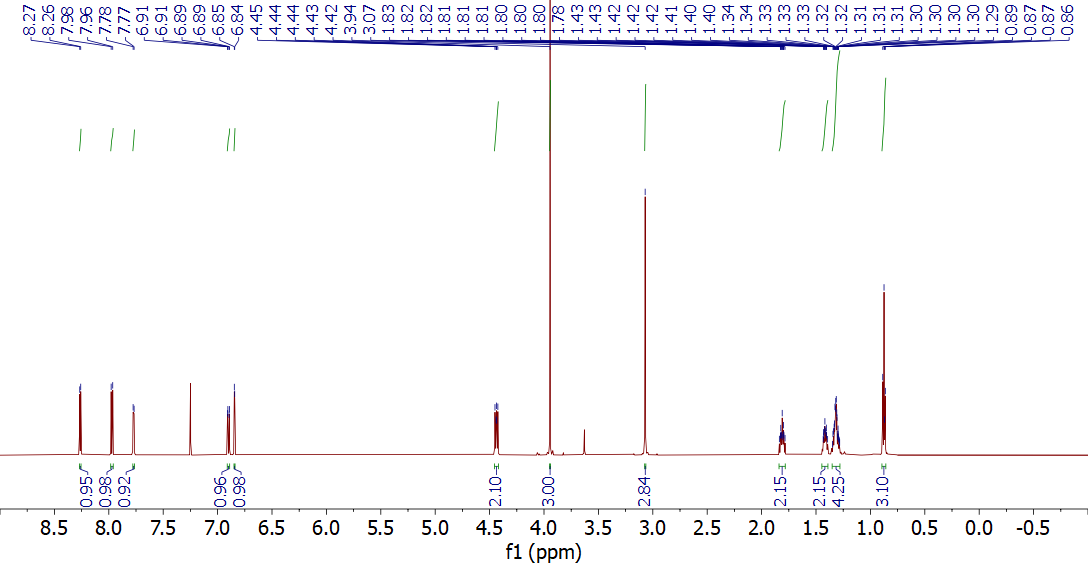


# ^13^C NMR spectrum of 9‐hexyl‐7‐methoxy‐1‐methyl‐9*H*‐pyrido[3,4‐*b*]indole (31):


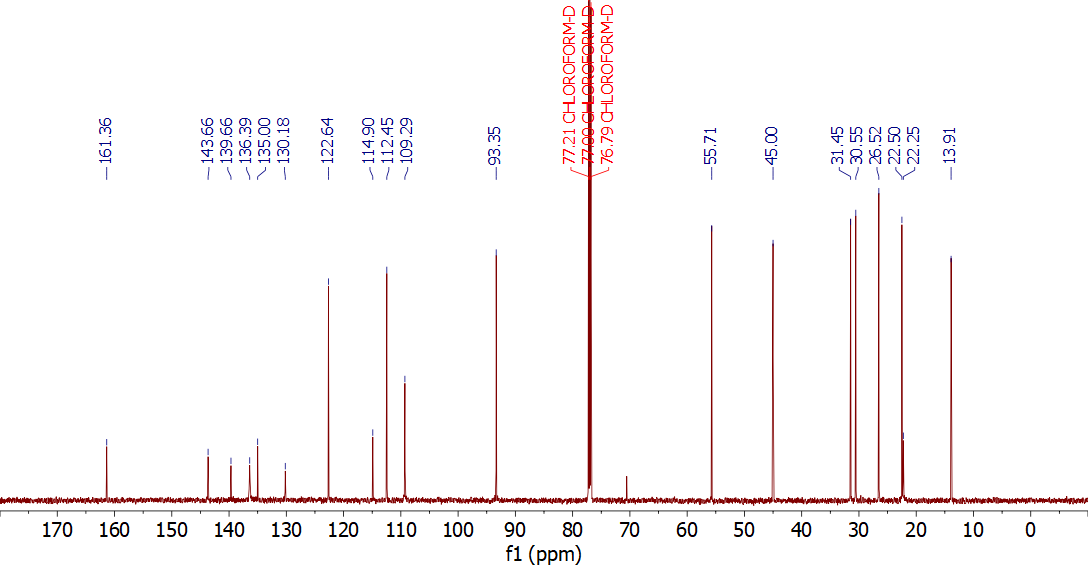


# ESI-HRMS spectrum of 9‐hexyl‐7‐methoxy‐1‐methyl‐9*H*‐pyrido[3,4‐*b*]indole (31):


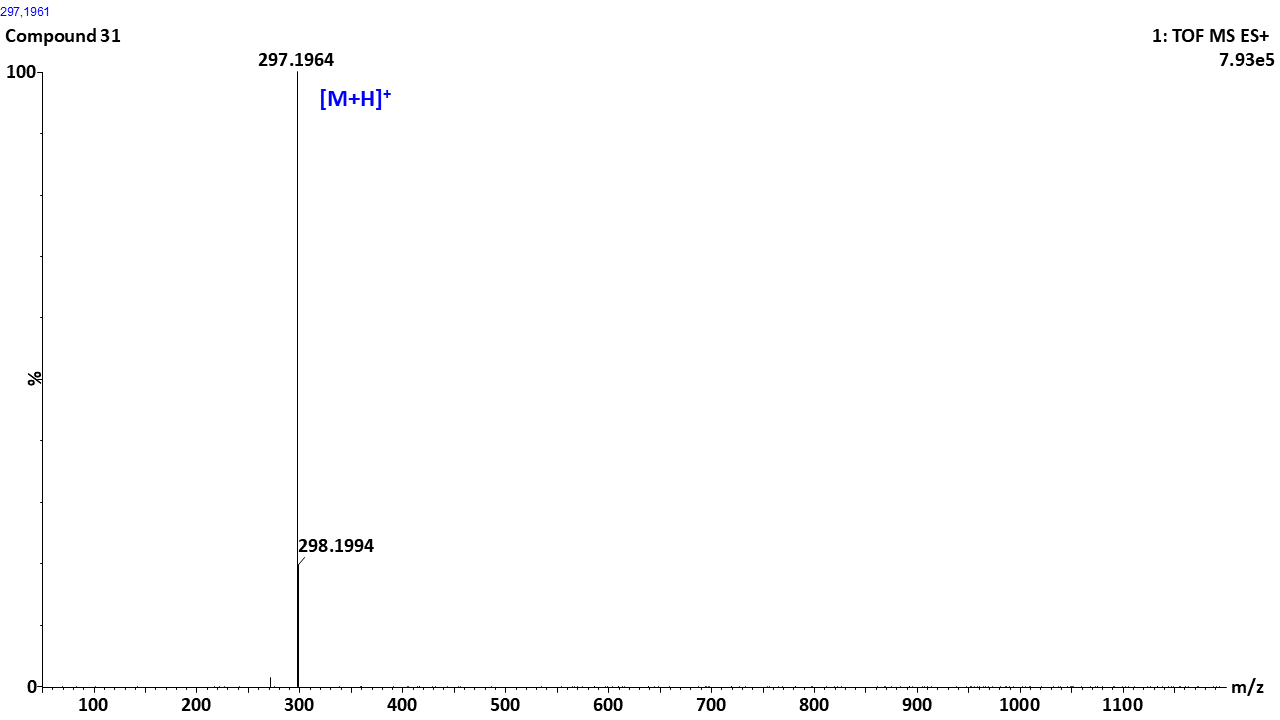


# HPLC-UV chromatogram of 9‐hexyl‐7‐methoxy‐1‐methyl‐9*H*‐pyrido[3,4‐*b*]indole (31):

# ^1^H NMR spectrum of 9‐(4‐bromobutyl)‐7‐methoxy‐1‐methyl‐9H‐pyrido[3,4‐*b*]indole (32):


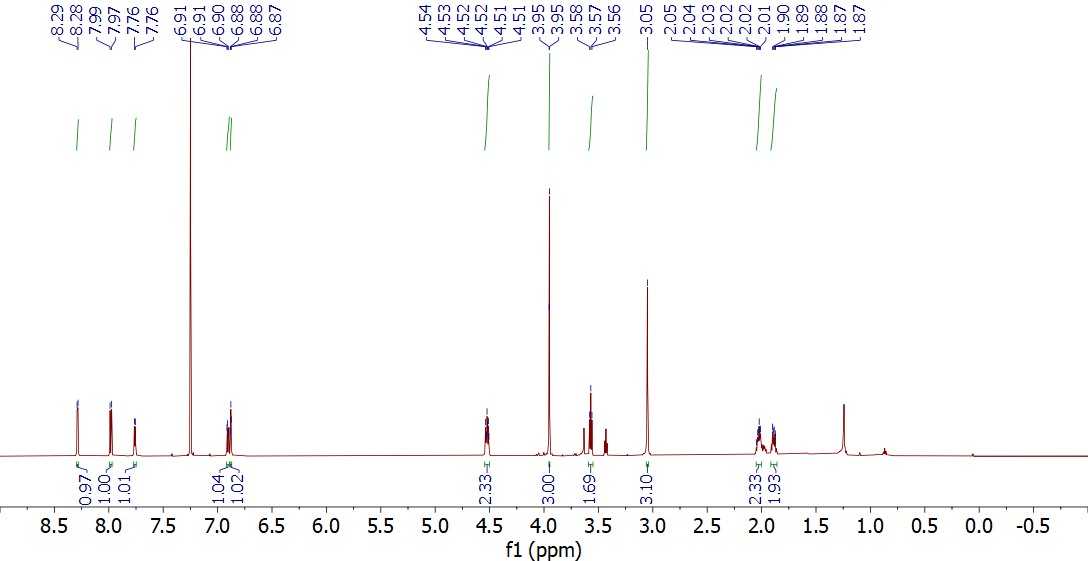


# ^13^C NMR spectrum of 9‐(4‐bromobutyl)‐7‐methoxy‐1‐methyl‐9H‐pyrido[3,4‐*b*]indole (32):


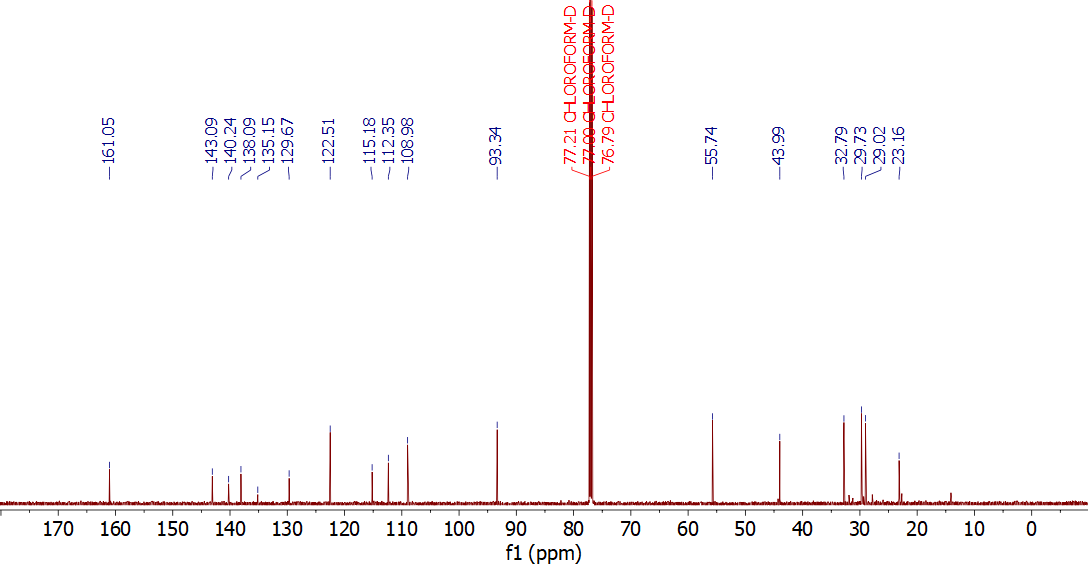


# ESI-HRMS spectrum of 9‐(4‐bromobutyl)‐7‐methoxy‐1‐methyl‐9H‐pyrido[3,4‐*b*]indole (32):


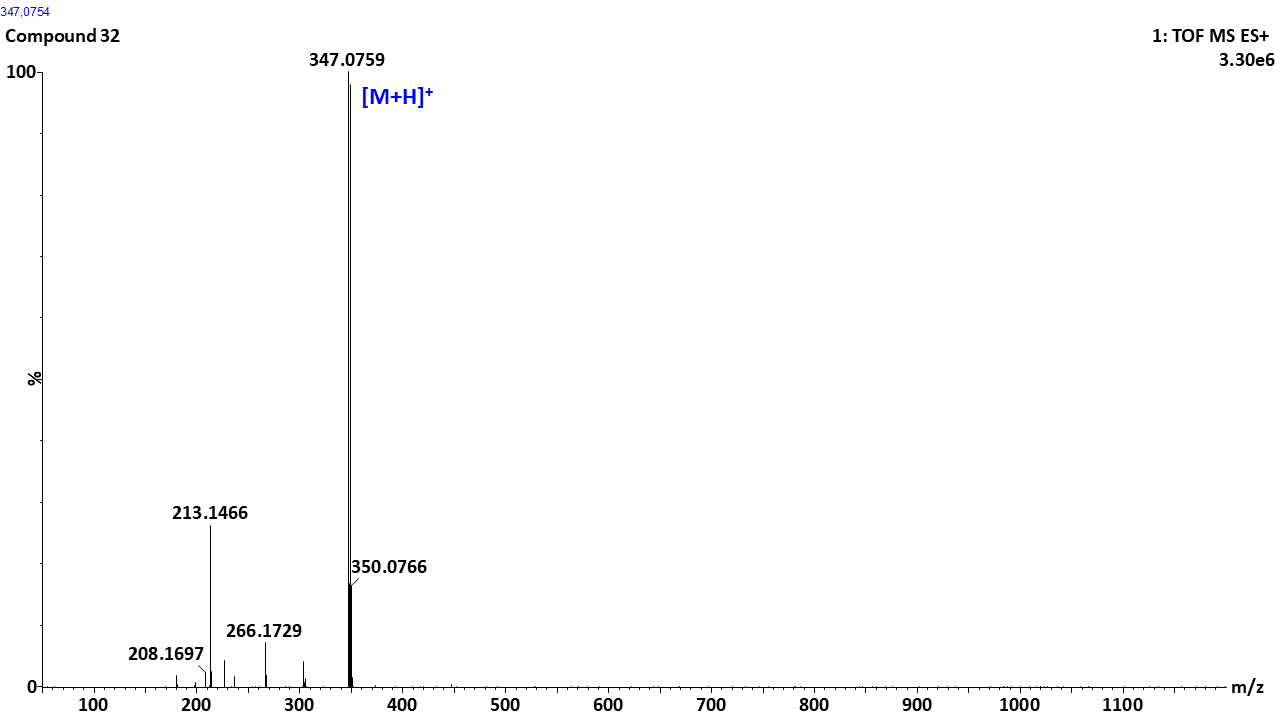


# HPLC-UV chromatogram of 9‐(4‐bromobutyl)‐7‐methoxy‐1‐methyl‐9H‐pyrido[3,4‐*b*]indole (32):

# ^1^H NMR spectrum of 9‐(5‐bromopentyl)‐7‐methoxy‐1‐methyl‐9*H*‐pyrido[3,4‐*b*]indole (33):


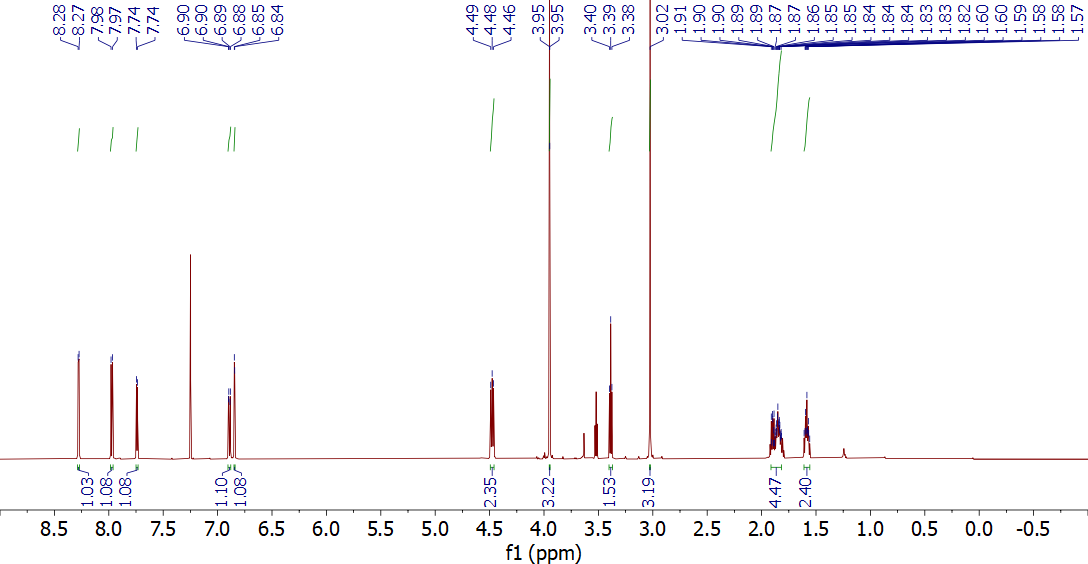


# ^13^C NMR spectrum of 9‐(5‐bromopentyl)‐7‐methoxy‐1‐methyl‐9*H*‐pyrido[3,4‐*b*]indole (33):


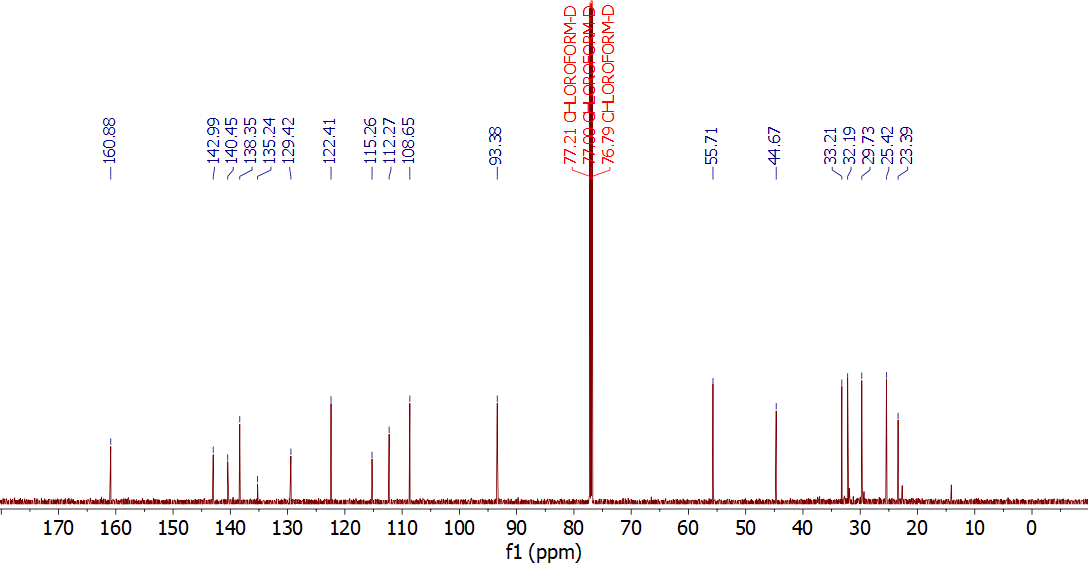


# ESI-HRMS spectrum of 9‐(5‐bromopentyl)‐7‐methoxy‐1‐methyl‐9*H*‐pyrido[3,4‐*b*]indole (33):


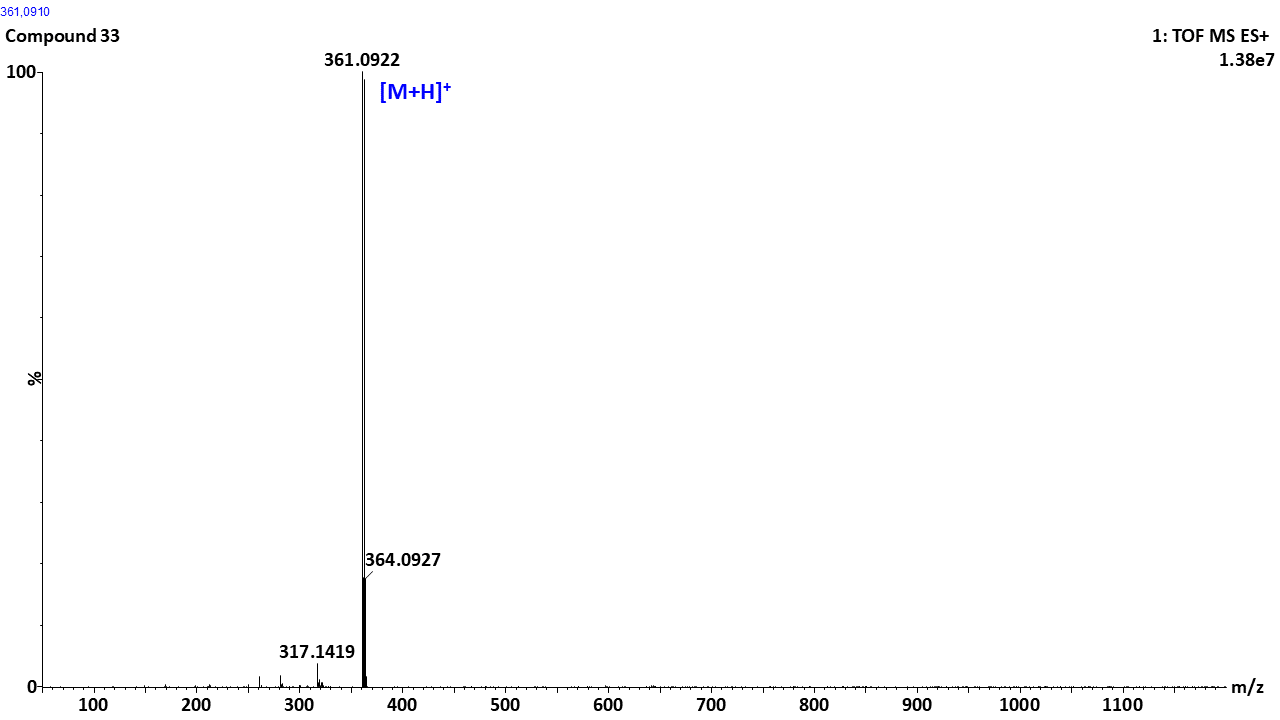


# HPLC-UV chromatogram of 9‐(5‐bromopentyl)‐7‐methoxy‐1‐methyl‐9*H*‐pyrido[3,4‐*b*]indole (33):

# ^1^H NMR spectrum of 9‐(6‐bromohexyl)‐7‐methoxy‐1‐methyl‐9*H*‐pyrido[3,4‐*b*]indole (34):


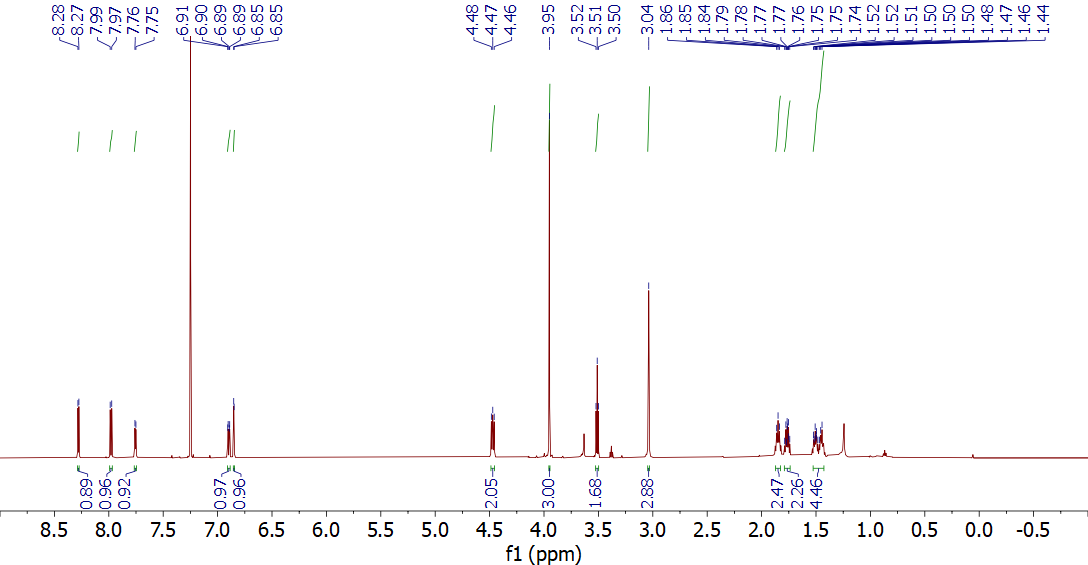


# ^13^C NMR spectrum of 9‐(6‐bromohexyl)‐7‐methoxy‐1‐methyl‐9*H*‐pyrido[3,4‐*b*]indole (34):


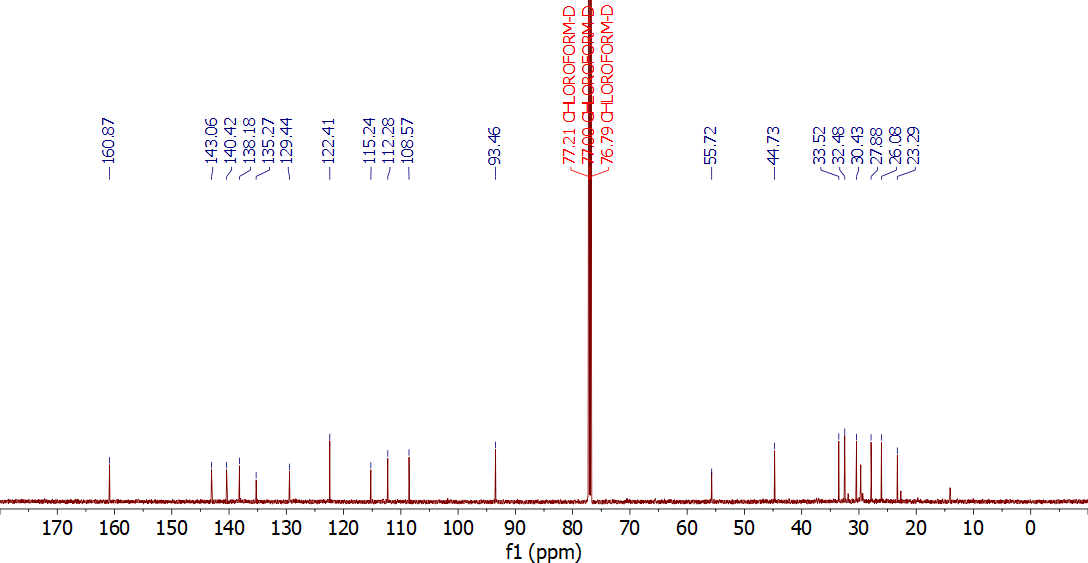


# ESI-HRMS spectrum of 9‐(6‐bromohexyl)‐7‐methoxy‐1‐methyl‐9*H*‐pyrido[3,4‐*b*]indole (34):


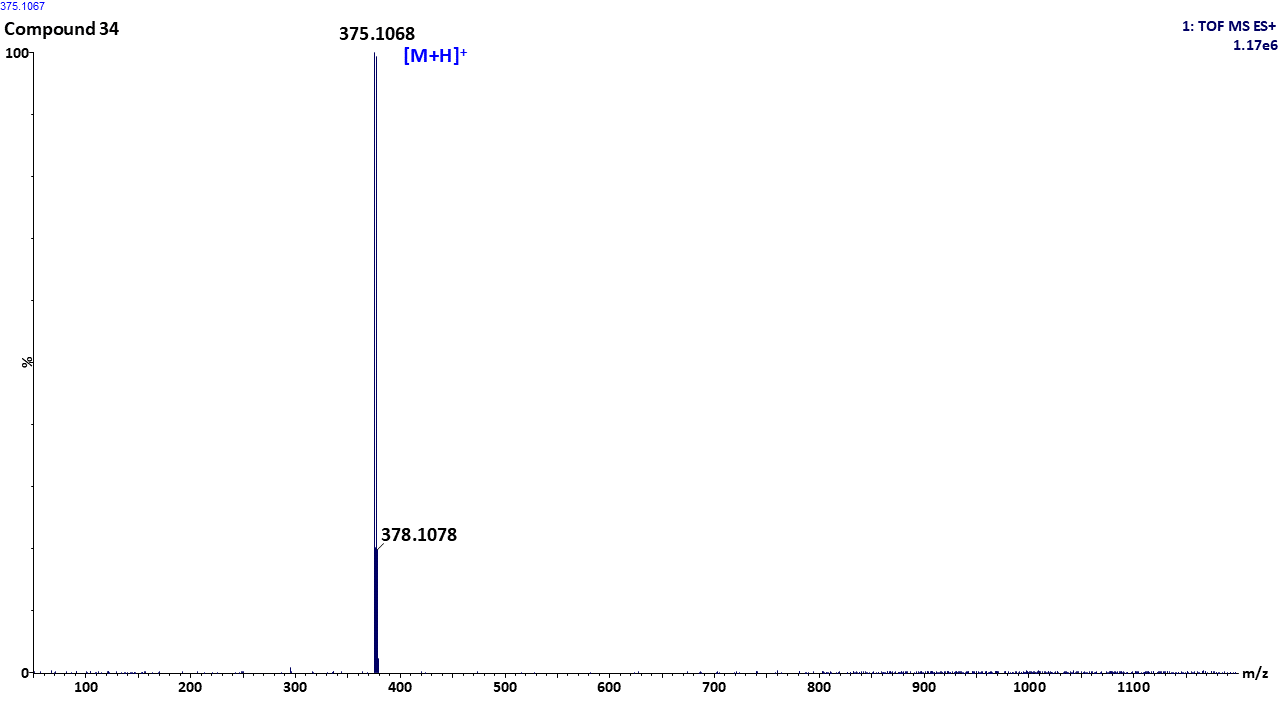


# HPLC-UV chromatogram of 9‐(6‐bromohexyl)‐7‐methoxy‐1‐methyl‐9*H*‐pyrido[3,4‐*b*]indole (34):

Figure S2. Inhibition of proliferation using all tested compounds treatment (**1-34,** positive control 1 µM doxorubicin). Each compound was tested on 9 cell lines and this effect is presented in a separate graph. The antiproliferative activity after the treatment at 10 µM for 48 h was measured using WST-1 assay and expressed as a percentage of control cells (0.1% DMSO treated, proliferation 100%). Each value represents the mean ± standard deviation of three independent experiments. The horizontal line highlights the 50% value.


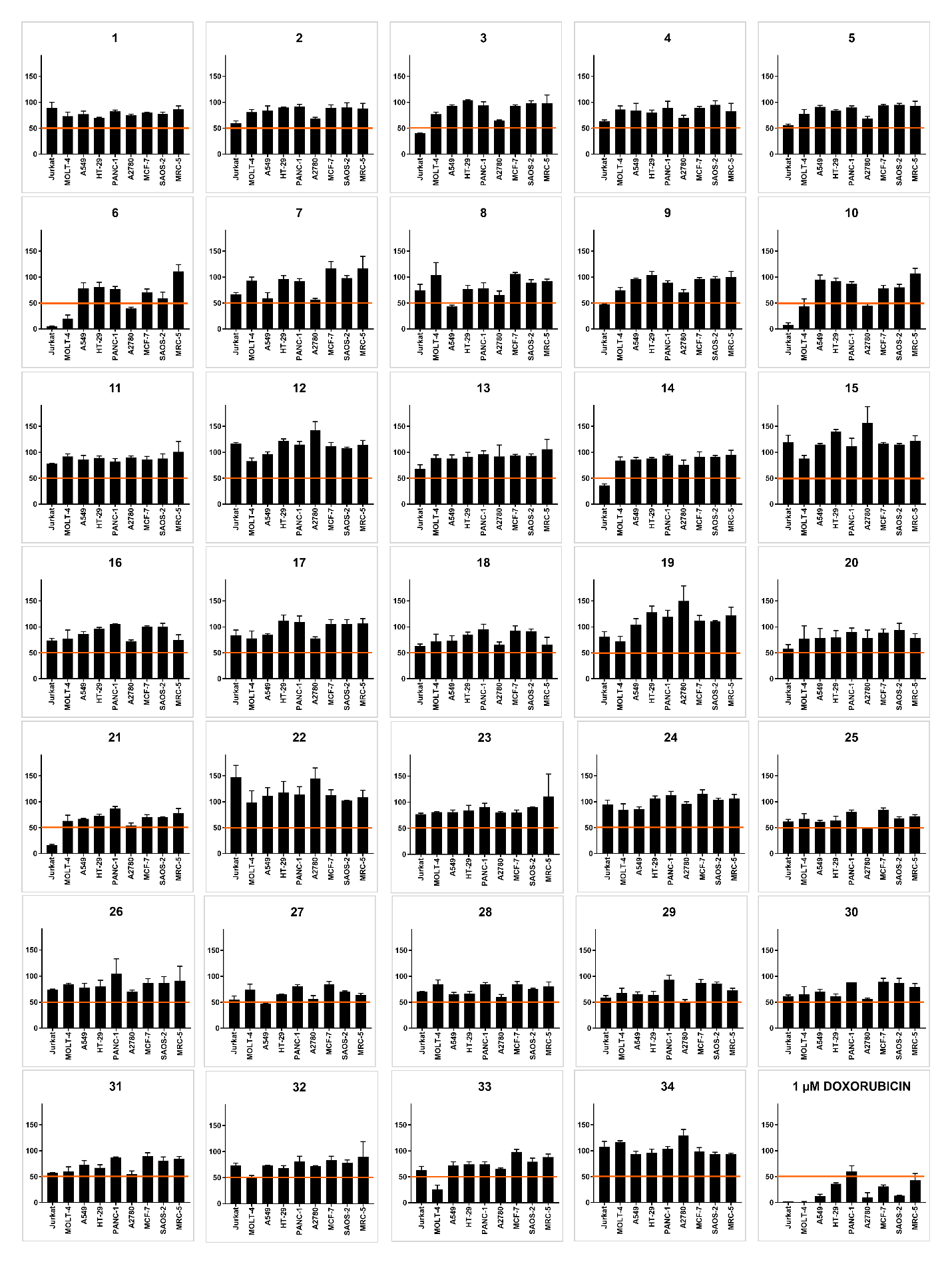


Figure S3. The overall antiproliferative effect of harmine (**1)** and its derivatives across all cell lines. The GP value of each compound represents the average proliferation after the application of a 10 µM dose within a 48-h treatment time interval on nine cell lines. Data were obtained from three independent experiments and are expressed as the percentage of cell proliferation relative to the control group treated with 0.1% DMSO, which was defined as 100%.

Figure S4. Effect of compound **6** on cell cycle progression in MRC-5 cells. Cells were treated with compound **6** (5–25 µM) or 0.1% DMSO (vehicle control) and incubated for (A) 24 h and (B) 48 h. Cell cycle distribution was subsequently analyzed by flow cytometry. Representative histograms from one of the independent experiments illustrate the mean percentage of cells in the G1, S, and G2 phases. Corresponding bar graphs show the cumulative phase distribution based on data from at least three independent experiments. Results are presented as mean ± SD. Statistical significance versus the negative control was evaluated using a t-test and is indicated as follows: *p* < 0.1 (*), *p* < 0.01 (**), *p* < 0.001 (***).
