# Supplementary material for: Harmine Derivatives as Anticancer Agents Endowed With Potent and Selective Antileukemia Activity: Synthesis, Biological Evaluation, Proapoptotic and Genotoxic Activity
Source: Arch Pharm (Weinheim). 2026 Feb 10;359(2):e70197. doi: 10.1002/ardp.70197 (PMC12892019; doi:10.1002/ardp.70197)
Supplement: Supplementary file 2 — ArchPharm SupplMat InChI Timbilla et al man. [file ARDP-359-e70197-s001.doc]

**Supplemental Material: Novel Compounds and Biological Screening Results**

Harmine derivatives as anticancer agents endowed with potent and selective antileukemia activity: Synthesis, biological evaluation, proapoptotic and genotoxic activity

Abdul Aziz Timbilla^1^, Filip Pidany^2^, Eliska Kohelova^2^, Jana Kroustkova^2^, Karel Kralovec^3^, Jan Rataj^4^, Martina Ceckova^4^, ​Negar Maafi^2^, Víctor Lopez^5^, ​Cristina Moliner Langa^5^, Stefan Kosturko^2^, Jaroslav Jenco^2^, Darina Muthna^1^, Darja Koutova^1^, Martina Rezacova^1^, Lucie Cahlikova^2^, Jakub Chlebek^2^, *, Radim Havelek^1^,*

^1^Department of Medical Biochemistry, Faculty of Medicine in Hradec Kralove, Charles University, Simkova 870, Hradec Kralove 500 03, Czech Republic

^2^Department of Pharmacognosy and Pharmaceutical Botany, Faculty of Pharmacy, Charles University, Akademika Heyrovskeho 1203, Hradec Kralove 500 05, Czech Republic

^3^Department of Biological and Biochemical Sciences, Faculty of Chemical Technology, University of Pardubice, Studentska 573, Pardubice 532 10, Czech Republic

^4^Department of Pharmacology and Toxicology, Faculty of Pharmacy in Hradec Kralove, Charles University, Akademika Heyrovskeho 1203, Hradec Kralove 500 05, Czech Republic

^5^Faculty of Health Sciences, Universidad San Jorge, 50830 Villanueva de Gállego (Zaragoza), Spain

*Corresponding authors

Assoc. Prof. Radim Havelek, Ph.D.

Department of Medical Biochemistry,

Faculty of Medicine in Hradec Kralove,

Charles University,

Simkova 870,

500 03 Hradec Kralove,

Czech Republic

Tel.: +420495816293

E-mail address: [havelekr@lfhk.cuni.cz](about:blank)

ORCID: 0000-0003-0528-1334

Assoc. Prof. Jakub Chlebek, Ph.D.

Department of Pharmacognosy and Pharmaceutical Botany,

Faculty of Pharmacy,

Charles University,

Akademika Heyrovskeho 1203,

500 05, Hradec Kralove,

Czech Republic

Tel.: +420495067232

E-mail address: chlej2aa@faf.cuni.cz

ORCID: 0000-0002-0697-5242

| **Compound No.** | **InChI** | **Biological Activity (*Growth Percentage after*** ***treatment with 10 µM concentration over 48-h)*^a^** | | | | | | | | | | | | | | | | |
| --- | --- | --- | --- | --- | --- | --- | --- | --- | --- | --- | --- | --- | --- | --- | --- | --- | --- | --- |
|  |  | **Jurkat** | | **MOLT-4** | | **A549** | | **HT-29** | | **PANC-1** | | **A2780** | | **MCF-7** | | **SAOS-2** | | **MRC-5** |
| **1** | 1S/C13H12N2O/c1-8-13-11(5-6-14-8)10-4-3-9(16-2)7-12(10)15-13/h3-7,15H,1-2H3 | 89 | | 73 | | 77 | | 70 | | 83 | | 75 | | 80 | | 78 | | 87 |
| **2** | 1S/C20H18N2O/c1-14-20-18(10-11-21-14)17-9-8-16(23-2)12-19(17)22(20)13-15-6-4-3-5-7-15/h3-12H,13H2,1-2H3 | 60 | | 81 | | 84 | | 90 | | 92 | | 69 | | 89 | | 90 | | 88 |
| **3** | 1S/C21H20N2O/c1-14-5-4-6-16(11-14)13-23-20-12-17(24-3)7-8-18(20)19-9-10-22-15(2)21(19)23/h4-12H,13H2,1-3H3 | 40 | | 77 | | 93 | | 104 | | 94 | | 65 | | 93 | | 98 | | 98 |
| **4** | 1S/C21H20N2O/c1-14-4-6-16(7-5-14)13-23-20-12-17(24-3)8-9-18(20)19-10-11-22-15(2)21(19)23/h4-12H,13H2,1-3H3 | 63 | | 86 | | 84 | | 80 | | 89 | | 70 | | 89 | | 95 | | 83 |
| **5** | 1S/C22H22N2O/c1-14-5-6-17(15(2)11-14)13-24-21-12-18(25-4)7-8-19(21)20-9-10-23-16(3)22(20)24/h5-12H,13H2,1-4H3 | 56 | | 78 | | 91 | | 84 | | 90 | | 69 | | 94 | | 95 | | 93 |
| **6** | 1S/C22H22N2O/c1-14-9-15(2)11-17(10-14)13-24-21-12-18(25-4)5-6-19(21)20-7-8-23-16(3)22(20)24/h5-12H,13H2,1-4H3 | 5 | | 20 | | 78 | | 81 | | 77 | | 40 | | 71 | | 59 | | 111 |
| **7** | 1S/C23H24N2O/c1-15(2)18-7-5-17(6-8-18)14-25-22-13-19(26-4)9-10-20(22)21-11-12-24-16(3)23(21)25/h5-13,15H,14H2,1-4H3 | 67 | | 93 | | 59 | | 96 | | 92 | | 56 | | 117 | | 98 | | 117 |
| **8** | 1S/C24H26N2O/c1-16-23-21(12-13-25-16)20-11-10-19(27-5)14-22(20)26(23)15-17-6-8-18(9-7-17)24(2,3)4/h6-14H,15H2,1-5H3 | 74 | | 104 | | 44 | | 77 | | 78 | | 65 | | 106 | | 89 | | 92 |
| **9** | 1S/C21H20N2O2/c1-14-21-19(9-10-22-14)18-8-7-17(25-3)12-20(18)23(21)13-15-5-4-6-16(11-15)24-2/h4-12H,13H2,1-3H3 | 47 | | 74 | | 96 | | 104 | | 89 | | 71 | | 96 | | 97 | | 100 |
| **10** | 1S/C22H22N2O3/c1-14-22-20(7-8-23-14)19-6-5-16(25-2)12-21(19)24(22)13-15-9-17(26-3)11-18(10-15)27-4/h5-12H,13H2,1-4H3 | 8 | | 44 | | 95 | | 92 | | 87 | | 45 | | 78 | | 80 | | 107 |
| **11** | 1S/C20H17FN2O/c1-13-20-17(9-10-22-13)16-8-7-15(24-2)11-19(16)23(20)12-14-5-3-4-6-18(14)21/h3-11H,12H2,1-2H3 | 78 | | 92 | | 86 | | 89 | | 82 | | 90 | | 86 | | 88 | | 101 |
| **12** | 1S/C20H17FN2O/c1-13-20-18(8-9-22-13)17-7-6-16(24-2)11-19(17)23(20)12-14-4-3-5-15(21)10-14/h3-11H,12H2,1-2H3 | 117 | | 83 | | 96 | | 122 | | 115 | | 143 | | 112 | | 108 | | 114 |
| **13** | 1S/C20H17FN2O/c1-13-20-18(9-10-22-13)17-8-7-16(24-2)11-19(17)23(20)12-14-3-5-15(21)6-4-14/h3-11H,12H2,1-2H3 | 68 | | 89 | | 88 | | 91 | | 96 | | 92 | | 94 | | 93 | | 106 |
| **14** | 1S/C20H17ClN2O/c1-13-20-17(9-10-22-13)16-8-7-15(24-2)11-19(16)23(20)12-14-5-3-4-6-18(14)21/h3-11H,12H2,1-2H3 | 36 | | 84 | | 86 | | 88 | | 94 | | 76 | | 91 | | 91 | | 95 |
| **15** | 1S/C20H17ClN2O/c1-13-20-18(8-9-22-13)17-7-6-16(24-2)11-19(17)23(20)12-14-4-3-5-15(21)10-14/h3-11H,12H2,1-2H3 | 119 | | 88 | | 115 | | 140 | | 112 | | 157 | | 117 | | 115 | | 122 |
| **16** | 1S/C20H17ClN2O/c1-13-20-18(9-10-22-13)17-8-7-16(24-2)11-19(17)23(20)12-14-3-5-15(21)6-4-14/h3-11H,12H2,1-2H3 | 74 | | 77 | | 86 | | 96 | | 105 | | 72 | | 100 | | 101 | | 75 |
| **17** | 1S/C20H16Cl2N2O/c1-12-20-16(7-8-23-12)15-5-4-14(25-2)10-19(15)24(20)11-13-3-6-17(21)18(22)9-13/h3-10H,11H2,1-2H3 | 84 | | 78 | | 85 | | 112 | | 109 | | 78 | | 106 | | 106 | | 107 |
| **18** | 1S/C20H17BrN2O/c1-13-20-17(9-10-22-13)16-8-7-15(24-2)11-19(16)23(20)12-14-5-3-4-6-18(14)21/h3-11H,12H2,1-2H3 | 63 | | 72 | | 73 | | 85 | | 95 | | 66 | | 93 | | 92 | | 66 |
| **19** | 1S/C20H17BrN2O/c1-13-20-18(8-9-22-13)17-7-6-16(24-2)11-19(17)23(20)12-14-4-3-5-15(21)10-14/h3-11H,12H2,1-2H3 | 81 | | 72 | | 104 | | 128 | | 119 | | 150 | | 112 | | 111 | | 122 |
| **20** | 1S/C20H17BrN2O/c1-13-20-18(9-10-22-13)17-8-7-16(24-2)11-19(17)23(20)12-14-3-5-15(21)6-4-14/h3-11H,12H2,1-2H3 | 58 | 77 | | 79 | | 80 | | 90 | | 79 | | 89 | | 94 | | 79 | |
| **21** | 1S/C20H17N3O3/c1-13-20-17(9-10-21-13)16-8-7-15(26-2)11-19(16)22(20)12-14-5-3-4-6-18(14)23(24)25/h3-11H,12H2,1-2H3 | 16 | 63 | | 67 | | 73 | | 87 | | 54 | | 70 | | 70 | | 78 | |
| **22** | 1S/C20H17N3O3/c1-13-20-18(8-9-21-13)17-7-6-16(26-2)11-19(17)22(20)12-14-4-3-5-15(10-14)23(24)25/h3-11H,12H2,1-2H3 | 147 | 98 | | 111 | | 118 | | 114 | | 145 | | 112 | | 102 | | 108 | |
| **23** | 1S/C20H17N3O3/c1-13-20-18(9-10-21-13)17-8-7-16(26-2)11-19(17)22(20)12-14-3-5-15(6-4-14)23(24)25/h3-11H,12H2,1-2H3 | 77 | 81 | | 81 | | 84 | | 91 | | 80 | | 80 | | 90 | | 111 | |
| **24** | 1S/C24H20N2O/c1-16-24-22(11-12-25-16)21-10-9-20(27-2)14-23(21)26(24)15-17-7-8-18-5-3-4-6-19(18)13-17/h3-14H,15H2,1-2H3 | 95 | 84 | | 86 | | 106 | | 112 | | 96 | | 115 | | 104 | | 106 | |
| **25** | 1S/C16H18N2O/c1-4-9-18-15-10-12(19-3)5-6-13(15)14-7-8-17-11(2)16(14)18/h5-8,10H,4,9H2,1-3H3 | 62 | 67 | | 61 | | 64 | | 80 | | 49 | | 84 | | 68 | | 72 | |
| **26** | 1S/C16H18N2O/c1-10(2)18-15-9-12(19-4)5-6-13(15)14-7-8-17-11(3)16(14)18/h5-10H,1-4H3 | 74 | 84 | | 78 | | 80 | | 105 | | 70 | | 87 | | 87 | | 91 | |
| **27** | 1S/C16H16N2O/c1-4-9-18-15-10-12(19-3)5-6-13(15)14-7-8-17-11(2)16(14)18/h4-8,10H,1,9H2,2-3H3 | 55 | 74 | | 47 | | 65 | | 81 | | 56 | | 84 | | 70 | | 64 | |
| **28** | 1S/C16H14N2O/c1-4-9-18-15-10-12(19-3)5-6-13(15)14-7-8-17-11(2)16(14)18/h1,5-8,10H,9H2,2-3H3 | 70 | 84 | | 66 | | 67 | | 84 | | 60 | | 85 | | 76 | | 80 | |
| **29** | 1S/C17H20N2O/c1-4-5-10-19-16-11-13(20-3)6-7-14(16)15-8-9-18-12(2)17(15)19/h6-9,11H,4-5,10H2,1-3H3 | 59 | 68 | | 66 | | 64 | | 93 | | 51 | | 87 | | 86 | | 73 | |
| **30** | 1S/C18H22N2O/c1-4-5-6-11-20-17-12-14(21-3)7-8-15(17)16-9-10-19-13(2)18(16)20/h7-10,12H,4-6,11H2,1-3H3 | 62 | 65 | | 70 | | 62 | | 88 | | 56 | | 89 | | 87 | | 79 | |
| **31** | 1S/C19H24N2O/c1-4-5-6-7-12-21-18-13-15(22-3)8-9-16(18)17-10-11-20-14(2)19(17)21/h8-11,13H,4-7,12H2,1-3H3 | 57 | 60 | | 73 | | 67 | | 87 | | 55 | | 90 | | 81 | | 84 | |
| **32** | 1S/C17H19BrN2O/c1-12-17-15(7-9-19-12)14-6-5-13(21-2)11-16(14)20(17)10-4-3-8-18/h5-7,9,11H,3-4,8,10H2,1-2H3 | 73 | 50 | | 73 | | 68 | | 81 | | 72 | | 84 | | 78 | | 90 | |
| **33** | 1S/C18H21BrN2O/c1-13-18-16(8-10-20-13)15-7-6-14(22-2)12-17(15)21(18)11-5-3-4-9-19/h6-8,10,12H,3-5,9,11H2,1-2H3 | 63 | 26 | | 72 | | 74 | | 74 | | 65 | | 98 | | 79 | | 88 | |
| **34** | 1S/C19H23BrN2O/c1-14-19-17(9-11-21-14)16-8-7-15(23-2)13-18(16)22(19)12-6-4-3-5-10-20/h7-9,11,13H,3-6,10,12H2,1-2H3 | 107 | 117 | | 94 | | 96 | | 103 | | 129 | | 98 | | 93 | | 93 | |

The antiproliferative effect of each compound across all cell lines was calculated following treatment with a 10 µM concentration, using a 48-h incubation period, and assessed via the WST-1 method relative to negative control cells (0.1% DMSO-treated, set at 100% proliferation). Each value is a mean of three independent experiments.
